# From Insect to Man: *Photorhabdus* Sheds Light on the Emergence of Human Pathogenicity

**DOI:** 10.1371/journal.pone.0144937

**Published:** 2015-12-17

**Authors:** Geraldine Mulley, Michael L. Beeton, Paul Wilkinson, Isabella Vlisidou, Nina Ockendon-Powell, Alexia Hapeshi, Nick J. Tobias, Friederike I. Nollmann, Helge B. Bode, Jean van den Elsen, Richard H. ffrench-Constant, Nicholas R. Waterfield

**Affiliations:** 1 School of Biological Sciences, University of Reading, Whiteknights, Reading, RG6 6AJ, United Kingdom; 2 Cardiff School of Health Sciences, Cardiff Metropolitan University, Llandaff Campus, Western Avenue, Cardiff, CF5 2YB, United Kingdom; 3 Life Sciences Building, Bristol University, 24 Tyndall Avenue, Bristol, BS8 1TQ, United Kingdom; 4 Primary Care Unit, Microbiology Department, Public Health England, Gloucester Royal Hospital, Great Western Road, Gloucester, GL1 3NN, United Kingdom; 5 Division of Biomedical Sciences, Warwick Medical School, Medical School Building, The University of Warwick, Gibbet Hill Road, Coventry, CV4 7AL, United Kingdom; 6 Buchmann Center for Life Sciences (BMLS), Fachbereich Biowissenschaften, Goethe Universität Frankfurt, 60438, Frankfurt, Germany; 7 Department of Biology & Biochemistry, University of Bath, Claverton Down, Bath, BA2 7AY, United Kingdom; 8 Biosciences, Cornwall Campus, University of Exeter, Penryn, TR10 9EZ, United Kingdom; University of Helsinki, FINLAND

## Abstract

*Photorhabdus* are highly effective insect pathogenic bacteria that exist in a mutualistic relationship with Heterorhabditid nematodes. Unlike other members of the genus, *Photorhabdus asymbiotica* can also infect humans. Most *Photorhabdus* cannot replicate above 34°C, limiting their host-range to poikilothermic invertebrates. In contrast, *P*. *asymbiotica* must necessarily be able to replicate at 37°C or above. Many well-studied mammalian pathogens use the elevated temperature of their host as a signal to regulate the necessary changes in gene expression required for infection. Here we use RNA-seq, proteomics and phenotype microarrays to examine temperature dependent differences in transcription, translation and phenotype of *P*. *asymbiotica* at 28°C versus 37°C, relevant to the insect or human hosts respectively. Our findings reveal relatively few temperature dependant differences in gene expression. There is however a striking difference in metabolism at 37°C, with a significant reduction in the range of carbon and nitrogen sources that otherwise support respiration at 28°C. We propose that the key adaptation that enables *P*. *asymbiotica* to infect humans is to aggressively acquire amino acids, peptides and other nutrients from the human host, employing a so called “nutritional virulence” strategy. This would simultaneously cripple the host immune response while providing nutrients sufficient for reproduction. This might explain the severity of ulcerated lesions observed in clinical cases of Photorhabdosis. Furthermore, while *P*. *asymbiotica* can invade mammalian cells they must also resist immediate killing by humoral immunity components in serum. We observed an increase in the production of the insect Phenol-oxidase inhibitor Rhabduscin normally deployed to inhibit the melanisation immune cascade. Crucially we demonstrated this molecule also facilitates protection against killing by the alternative human complement pathway.

## Introduction

When attempting to understand the evolution of human pathogenicity, microbiologists have traditionally compared the genomes of human pathogenic strains to ones that are less virulent or even avirulent. Examples include *Yersina pestis* / *Y*. *pseudotuberculosis* and *Bacillus anthracis* / *B*. *cereus*. Whilst this strategy can lead to the discovery of novel pathogenicity determinants or virulence factors such as toxins and secretion systems, it is usually difficult to identify more subtle changes that enable a pathogen to exploit specific host resources. Different lineages will have been exposed to different selective pressures leading to the accumulation of ancillary adaptations to diverse environmental conditions that may confound the identification of the key pathogenicity and virulence adaptations. In the case of *Photorhabdus asymbiotica*, a recently emerged human pathogen, the genes responsible for both insect and human pathogenicity are encoded in the same genome. We argue it is likely that many of the *Photorhabdus* virulence factors may be equally appropriate for insect and human infections. In addition *Photorhabdus* has a clearly defined life history in that it never leaves an animal host, either quiescent in its nematode symbiont or pathogenic in the prey insect [1]. This provides a relatively consistent and predictable, if punctuated, nutrient supply, and reduces the number of adaptations required for dealing more variable environmental conditions. These bacteria therefore represent a good model to investigate the molecular constraints that prevent entomopathogenic bacteria from infecting mammalian hosts and conversely, the molecular adaptations required to emerge as a human pathogen.

**Fig 1:**
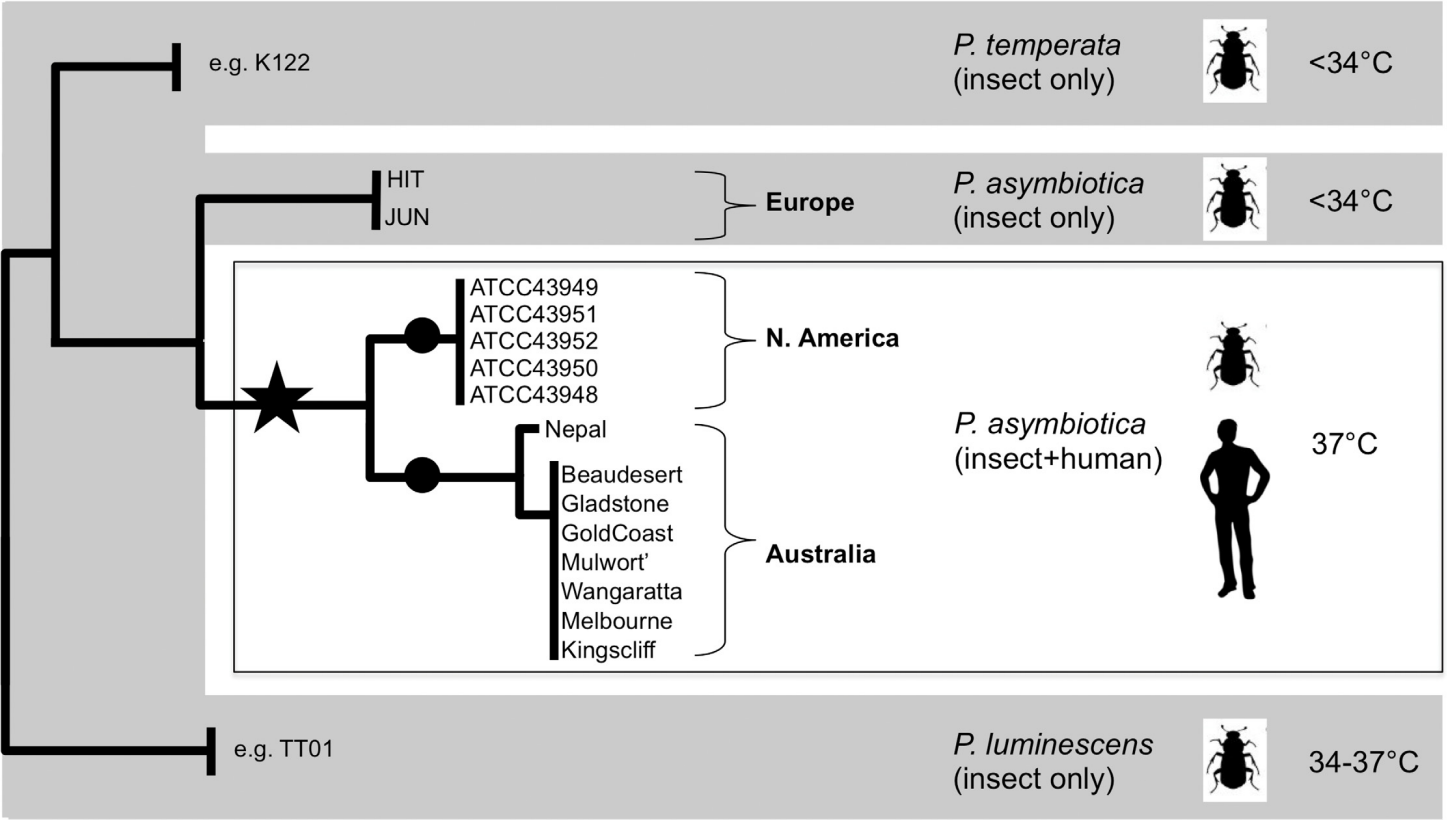
The genus *Photorhabdus* contains three predominant species. A stylized representation of a previous six gene MLST phylogeny (*adk*, *ghd*, *mdk*, *ndh*, *pgm* and *recA*) of *Photorhabdus* (adapted from [5]) is shown. The grey areas indicate species that consist of multiple strains, the majority of which are unable to grow above 34°C, with only a few *P*. *luminescens* strains capable of growth at temperatures up to 37°C. Example strains are *P*. *luminescens* ^TT01^ and *P*. *temperata* ^K122^. The clinical strains adapted to 37°C are boxed. The stars and circles indicate the potential historical timing of temperature adaptation, which could have occurred ancestrally (star) or independently (circles) in different geographical isolates.

All members of the genus *Photorhabdus* are insect pathogenic and form a species-specific mutualistic association with nematodes belonging to the genus *Heterorhabditis*. Many different entomopathogenic nematode complexes (EPN) have been identified from soils across the world, and many are produced commercially for the bio-control of insect pests. In the well-characterised life cycle, *Photorhabdus* cells are delivered into the open blood system (hemocoel) of an insect host; they resist the immune response and rapidly kill the insect. They subsequently bio-convert the insect tissues into more bacteria that provide a food source for the replicating nematodes. When resources are depleted the bacteria re-associate with new infective juvenile nematodes, which leave the cadaver in search of new prey [1]. While the precise phylogeny of the genus is subject to regular review [2–4], we can recognise three distinct species, *P*. *luminescens*, *P*. *temperata and P*. *asymbiotica* [5] (Fig 1). In addition to the normal insect life cycle, *P*. *asymbiotica* is also the etiological agent of a serious human infection termed Photorhabdosis [6]. It shows a range of clinical symptoms, including severe ulcerated skin lesions both at the initial infection foci and later at disseminated distal sites. Reported cases have required extensive antibiotic intervention, which in many cases also relapsed [7]. It is likely that many cases of Photorhabdosis are misdiagnosed as *Photorhabdus* is not currently in the databases of automated diagnosis machines and its accurate identification requires specialist knowledge [8].

To the best of our knowledge, human Photorhabdosis is non-transmissible. Our unpublished work confirmed that the symbiont nematode of an Australian clinical isolate, *Heterorhabditis geradii*, is unable to survive prolonged exposure to temperatures in excess of 32°C. The nematodes are therefore unlikely to penetrate into deep tissues. We suggest that *P*. *asymbiotica* have alternative, as yet unidentified, mammalian or bird hosts. In support of this we have demonstrated that Heterorhabditid nematodes are capable of penetrating through dead *ex vivo* rat dermal tissue (unpublished). We argue it is likely that pre-existing adaptations required for insect pathogenicity contribute significantly toward allowing *P*. *asymbiotica* to also infect human hosts. This would include factors that enable *Photorhabdus* to resist destruction by components of the insect innate immune system such as phagocytic haemocytes, antimicrobial peptides and the complement-like Phenol-Oxidase cascade. Conservation of components of the insect and mammalian innate immune systems would support this argument [9] [10] [11] [12]. In addition, previous studies revealed that many of the cytotoxins and virulence factors produced by *Photorhabdus* are equally effective against both insect and mammalian professional phagocytes [13]. It has been suggested that invertebrates represent a “training-ground” for emerging mammalian pathogens [14] [15].

We previously performed detailed comparative genome analyses of *P*. *luminescens* strain TT01 (*Pl* ^TT01^) and two *P*. *asymbiotica* strains, ATCC43949 from the USA (*Pa* ^ATCC43949^) and Kingscliff from Australia (*Pa* ^kingscliff^) [16, 17]. These studies confirmed that the clinical and insect restricted strains were very similar in gene content and short-range synteny. Differences were mainly restricted to a lower diversity of virulence factor duplications in the clinical strains compared to *Pl* ^TT01^. The clinical strains also possess a second predicted type III secretion system and alternative effectors, consistent with their additional facultative intracellular invasion abilities [16]. Nevertheless it was not possible to pinpoint specific genetic differences that have enabled the *P*. *asymbiotica* to infect humans.

In the normal lifecycle of *Photorhabdus*, they replicate at the ambient temperature of their insect host. Furthermore their nematode partners cannot survive prolonged exposure to temperatures in excess of 32–34°C (unpublished data). Accordingly, the majority of *Photorhabdus* spp. are also unable to grow at temperatures in excess of 32–34°C [18], which restricts them to poikilothermic hosts. The clinical *P*. *asymbiotica* isolates clearly survive within human hosts and are routinely cultured at 37°C in the laboratory. However, at least some non-clinical *Photorhabdus* strains are also able to grow at temperatures in excess of 37°C [18]. Therefore it seems that temperature tolerance is not the sole barrier for these insect pathogens to establish an infection in humans, suggesting further adaptations are required.

Many mammalian pathogens use the temperature of the host body as a signal to induce changes in gene expression relevant to infection [19]. In pathogens such as *Yersinia*, which move either from the environment or an invertebrate host into a mammal, temperature is a key signal that activates production of host-specific factors [20]. It is interesting to note that genomic studies have revealed that the *Photorhabdus* are genetically closely related to the *Yersinia*. Furthermore the *P*. *asymbiotica* specific pPAU1-like plasmids [16, 17] also share limited homology to the *Yersinia pestis* pMT1 plasmid, essential for colonisation of the flea vector. In this study we used a combination of RNA-seq, proteomics and phenotype microarrays to examine differences in transcription, translation and phenotype of *P*. *asymbiotica* at 28°C versus 37°C, representative of ambient insect and human host temperatures respectively. Our findings suggest that while *P*. *asymbiotica* does use elevated temperature as a signal to induce production of certain virulence factors, the majority of virulence factors remain unchanged, at least in the absence of other host-specific signals. Most strikingly, growth at 37°C leads to significant changes in metabolism which restricts utilization of the majority of carbon and nitrogen sources that can otherwise support respiration at 28°C. This suggests that the bacteria have evolved to adopt a temperature dependent “nutritional virulence” strategy, required for human infection, but inappropriate for an insect host.

## Materials and Methods

### Bacterial culture

*Pa* ^ATCC43949^ was routinely grown in Lysogeny Broth (LB) with shaking (250 rpm) or on LB agar plates for 48 h at 28°C, unless otherwise stated. Where relevant, microaerobic conditions were produced by incubating agar plates in a 2.5L anaerobic jar with an AnaeroGen Sachet (Oxoid AN0025) at the indicated temperatures for 3–4 days, which lowers O_2_ levels to less than 1%. Full anaerobic conditions were achieved using a Modular Atmospheric Controlled System anaerobic cabinet (DW Scientific). Haemolysis was determined on Tryptic Soy Agar base supplemented with 5% Sheep blood *v/v* (Oxoid). Carbon utilisation was determined using a modified version of Oxidation/Fermentation media (0.2% *w/v* Casein, 85 mM NaCl, 1.7 mM K_2_HPO_4_, 0.008% *w/v* Bromothymol blue, 0.8% *w/v* agar).

### Thermal tolerance assays

Cultures were initially grown overnight at 28°C in LB media with aeration. Overnight cultures were then diluted to 10^5^ cfu/ml into fresh LB media and added to the wells of a sterile 96 well PCR plate. Using the gradient function on a thermocycler cultures were exposed to temperatures of 30–45°C for either 4 or 18 h. To assess viability from the large number of samples following heat exposure a kinetics-based method utilizing a bacterial growth curve was used. To do this heat-exposed cultures were diluted 1:100 into fresh LB within a sterile flat bottom 96-well plate (Costar, Corning). Cultures were then incubated at 28°C with orbital shaking for 24 h with optical density (OD_600_) readings taken every 10 minutes using a BMG labtech microtitre plate reader.

### Bioluminescence

*Photorhabdus* cultures were grown in LB broth at 28°C, 250 rpm for 16–18 h, diluted 1:100 in LB and 200 μl aliquoted in a 96-well “white” microtitre plate. Plates were incubated at the specified temperature in a Fluostar plate reader (BMG) with luminescence optic installed and regular measurements taken with continuous orbital shaking in between measurements for 24 h.

### RNA purification

*Pa* ^ATCC43949^ was grown overnight in 10 ml LB at 28°C, 250 rpm, and sub-cultured (1:100 dilution) in 50 ml media at either 28°C or 37°C in 250 ml flasks for 4 h to mid-log phase (OD_600_ ~0.6). To extract total RNA, a 10 ml aliquot of each culture was added to 25 ml RNA*later* (Ambion) and centrifuged at 10,000 rpm, 4°C in a JA-25.50 rotor centrifuge (Beckman). RNA was isolated from bacterial pellets using the miRNeasy kit (Qiagen), with an on-column DNase treatment (Qiagen), and RNA eluted in 70 μl RNAse-free H_2_0. To ensure complete removal of DNA, a subsequent DNase treatment was performed using the Turbo^™^ DNase-free kit (Ambion). The concentration and integrity of RNA samples was determined with an Experion RNA StdSens analysis kit (Bio-Rad Laboratories).

### RNA-seq library preparation

Ribosomal RNA was depleted from RNA samples using the Ribozero kit for Gram-negative bacteria (Epicentre). rRNA depletion was verified and samples quantified using a 2100 Bioanalyzer with a RNA 6000 Pico kit (Agilent). Strand-specific RNA-seq libraries were constructed using the Illumina compatible ScriptSeq mRNA-seq library preparation kit (Epicentre). cDNA libraries were quantified using a 2100 Bioanalyzer with a DNA 1000 kit (Agilent). To provide experimental robustness we constructed three RNA-seq libraries for each condition using RNA extracted from independent biological replicates and sequenced each using a different sequencing strategy. The first replicate of each library was sequenced in two separate lanes on the Genome Analyzer IIx (Illumina), with 36 bp paired-end reads at the University of Exeter Sequencing Service (Accession numbers SRR1555173 and SRR1555174). The second replicate of each library was sequenced on the HiSeq 2000 (Illumina), in two separate lanes with 70 bp paired-end reads at the University of Exeter Sequencing Service (Accession numbers SRR1555126 and SRR1555127). The third replicate of each library was sequenced on the HiSeq 2000 (Illumina), multiplexed with 10 libraries/lane with 100 bp paired-end reads (Accession numbers SRR1555150 and SRR1555151) by Source Bioscience (UK).

### RNA-seq analysis

The analysis of the RNA-seq data was performed on linux servers running debian OS, with 96GB RAM. A comparative analysis was performed between treatments. For each treatment, the raw data in fastq format was converted to bfq format. The MAQ alignment software (version 0.7.1) [21] was used to align the Illumina data to the *Photorhabdus asymbiotica* ATCC43949 genome and plasmid gene models obtained from Genbank. Custom PERL scripts were then used to count the number of reads aligned to each gene and convert the data into a format suitable for statistical analysis. The data was normalised by reads per kilobase of exon per million mapped reads (RPKM). The RPKM measure was chosen as read density reflects the molar concentration of a transcript in the starting sample by normalizing for RNA length and for the total number of reads in the measurement. RPKM normalization enables comparison of transcript levels both within and between samples [22]. Treatments were then compared using DESeq, an R package that estimates variance-mean dependence in count data derived from RNA-seq experiments and tests for differential expression based on a model using the negative binomial distribution, to identify differentially expressed genes from different samples [23]. Using DESeq it was possible to generate text files containing the expression values for the samples, and a P-value for each gene to denote its expression difference between libraries. In addition the RNA-seq data was visualised using the methods described by Croucher *et al* [24]. Reads were aligned to the genome and plasmid of *P*. *asymbiotica* ATCC43949 using SSAHA2 (version1.0.9) [25]. The cigar2Coverage PERL script was used to convert the SSAHA2 output into a format compatible with the Artemis genome browser [26]. This allowed the mapped transcriptome data to be viewed, in a strand-specific manner, as a graph relative to the genome annotation.

### Quantitative Real-Time PCR

Quantitative real time-PCR (qRT-PCR) was performed using the OneStep RT-PCR kit (Qiagen) as recommended by the manufacturer, with 100 ng of the appropriate RNA sample. PCR reactions were performed using the StepOnePlus Real Time PCR System (Agilent Biosystems) with reaction conditions as follows: 50°C x 30 min, 95°C x 15 min and 30 cycles of 95°C x 30 s, 58°C x 30 s, 72°C x 45 s. Cycle threshold (C_t_) values were calculated using StepOne software v2.2 (Agilent Biosystems) and comparative analysis (ΔΔC_t_) performed using the Relative Expression Software Tool (REST-2009, Pfaffl 2002) with *rpoA* and *csrB* as reference genes. qRT-PCR primers are listed in S13 Table.

### 2-Dimensional—Difference Gel Electrophoresis (2D-DIGE)

A 10 ml aliquot of each exponential phase culture used for the RNA purification for the RNA-seq experiment (see above) was centrifuged at 4500 rpm for 10 min. The cell pellets were sent on dry ice to Cambridge Centre for Proteomics. Cells were lysed in CHAPS/Thiourea buffer (6 M Urea, 2M Thiourea, 4% Chaps, 5mM Mg Acetate, 10mM Tris pH 8.5) by standard sonication. Samples were quantified using Quick Start^™^ Bradford (ref^2^) and 50 μg of each sample was labeled with 250pmol Cy3 dye (GE Healthcare) for 30 min at room temperature, protected from light. A 50μg pool of all samples was labeled with 250 pmol Cy5 in order to normalize results from replicate gels. 10 mM lysine was added to quench the reaction and incubated for 10 min at room temperature. Equivalent concentrations of Cy3-labeled sample and Cy5-labeled pool were mixed and diluted in 2x Sample buffer (8M Urea, 4% Chaps, 2% DTT, 2% IPG buffer 3-10NL (GE Healthcare). Samples were incubated in the dark for 15 min and then diluted in De-streak rehydration solution (GE Healthcare). 2D-DIGE was performed as previously described [27].

### Phenotype Microarrays

*Pa* ^ATCC43949^ and *Pl* ^TT01^ were grown from frozen glycerol stock on LB agar plates for 48 h at 28°C, a swab of culture was re-suspended in the appropriate media for each plate and adjusted to 85% transmittance measured using a Biolog turbidimeter. These cultures were diluted 1:100 in the appropriate media and PM plates were inoculated with 100 μl per well (three repeats per plate). We found that the recommended inoculating fluid (IF0a) supplied by Biolog interferes with the growth and respiration of *Photorhabdus* spp. Therefore, we used an adapted version of M9 media (12.5 mM Na_2_HPO_4_, 22 mM KH2PO4, 8.5 mM NaCl, 2 mM MgSO4, 100 uM CaCl2, pH 6.8) supplemented with 1x Dye A (Biolog). In addition, plates PM1 and PM2 were also supplemented with 0.5% (*w/v*) casein and plates PM3B, PM6 and PM7 were supplemented with 20 mM mannose. Plates PM1, PM2, PM3B, PM6, PM7 and PM8 were supplemented with 1x RPMI vitamin mix when incubated at 37°C. Plates PM9 and PM10 utilised the manufacturer’s medium (2.0 g of tryptone, 1.0 g of yeast extract, and 1.0 g of NaCl per liter). Plates were incubated at the indicated temperature in an OmniLog incubator, formazan formation was monitored every 15 min for 48 h and kinetic data was analysed using OmniLog-PM software (Biolog).

### Secondary metabolite analysis

*Pa* ^ATCC43949^ was grown at 28°C and 37°C in LB medium upon addition of 10% (w/v) amberlite XAD-16 adsorber resin. Supernatant and XAD-16 were separated by decantation. After washing the adsorber resin the bound compounds were eluted upon incubating twice with 2 ml methanol at room temperature for 20 min and the solvent was removed under reduced pressure. Then the samples were re-dissolved in 0.5 ml methanol, centrifuged (10 min, 13500 rpm, room temperature), diluted 1:10 with methanol and analysed with a Dionex Ultimate 3000 system (Thermo Scientific, Dreieich, Germany) using an Acquity UPLC BEH C18 1.7μm RP column (Waters GmbH, Eschborn, Germany) coupled to an AmaZon X mass spectrometer (Bruker Daltonik GmbH, Bremen, Germany). Metabolites were gradually eluted from the column in 22 min (gradient from 5% to 95% acetonitrile in H_2_O with 0.1% formic acid; flowrate of 0.6 mL/min).

### Insect virulence assays

Caterpillar virulence assays were conducted essentially as previously reported [28]. Briefly, first day fifth instar *Manduca sexta* larvae raised on an antibiotic free artificial wheat-germ diet were injected into an ethanol (70% *v/v*) swabbed region (using a 27-gauge insulin needle (VWR) just above the first proleg with a 50 μl of a dilution (in 1xPBS) of mid-exponentially grown *Pa* ^ATCC43949^ bacteria (taken at OD_600_ = 0.4 with aeration at 28°C). Caterpillars were placed on ice for 10 minutes prior to injection to reduce mobility. Two cohorts of 30 insects were injected with estimated 1000 cells each. One cohort was then placed at 28°C and the other at 37°C. The larvae were monitored over 7 days using physical stimulus to assess their status.

### Complement activity assay

The Wielisa Total Complement System Screen (Wieslab), described by Seelen and co-workers [29], was used to detect inhibition of the classical (CP), mannose-binding lectin (MBLP) and alternative (AP) complement pathways by rhabduscin-containing supernatants from strains *Pl* ^TT01^, *Pa* ^ATCC43949^ and *Pa* ^Kc^. After 24 hours, the induced cultures were centrifuged at 3900 g for 20min and the supernatants were used in Wielisa complement activity assay. 6μl of rhabduscin-containing supernatant was added to 14 μl of concentrated human serum (positive control serum, supplied with the kit) and assayed for complement activity after pre-incubation at 30°C or 37°C for 30 min. The assay was completed in duplicate, according to the manufacturers instructions, and included a blank, a positive control (human serum from healthy individuals) and a negative control (heat inactivated serum). Complement activity inhibition was quantified from the absorbance at 405 _nm_ using the equation: (sample-negative control)/(positive control-negative control) x 100%. Rhabduscin analog was produced from heterologous expression of *isnAB* from *P*. *luminescens* ^TTO1^ in *E*. *coli* as described previously [30] and its structure was confirmed by detailed NMR analysis.

### Serum resistance

Human serum (male) from type AB plasma was obtained from Sigma-Aldrich (Catalog number H4522). Overnight cultures of *Pl* ^TT01^, *Pa* ^ATCC43949^ and *E*. *coli* ^DH5α^ were diluted in LB or LB supplemented with 20% (*v/v*) human serum type AB, Heat Inactivated human serum type AB and 0.9% saline at a starting optical density of 0.05 at 600 nm. A total of 150 μl of each diluted cell suspension was added in triplicate into wells of a 96-well plate (Corning^®^ Costar). The assay was conducted at 28°C, shaking, and growth was monitored using an automated plate reader FluoStar Omega (BMG Labtech) with measurements taken every 30 min for 24 h.

### Murine macrophage challenge

J774.2 (ECACC reference 85011428) murine macrophage-like cells (2x10^6^ cells per well on poly L-lysine-coated plastic coverslips) were washed twice with pre-warmed medium, and triplicate wells were infected with GFP labeled *Pa* ^ATCC43949^ (using the *gfp*-expressing plasmid pHC60) at a multiplicity of infection of ten bacteria per cell in a 5% CO_2_ atmosphere. Bacteria and mammalian cells were centrifuged for 5 min at 800g to enhance bacteria—host interactions. After incubation at 37°C for 1 h, cells were washed five times with pre-warmed medium without serum and prepared for microscopic observation as follows. Cells were fixed with 4% (w/v) PBS-paraformaldehyde at room temperature for 1 h, washed three times with PBS and incubated with 50 mM ammonium chloride to minimize auto-fluorescence. Coverslips were further washed with PBS and the cells were permeabilized with 0.5% (v/v) Triton X-100 in PBS for 10 min. Non-specific binding sites were blocked with 0.5% (w/v) bovine serum albumin (BSA) in PBS. F-actin was stained with TRITC-phalloidin (Sigma). Stained cells were washed extensively with PBS, mounted overnight at 4°C with Mowiol and analyzed with a Zeiss confocal microscopy.

### Manduca hemocyte in vivo challenge

*Manduca sexta* were individually reared as described [31]. Briefly, larvae were maintained individually at 25°C under a photoperiod of 17 hours light: 7 hours dark and fed on an artificial diet based on wheat germ. *Manduca* larvae (taken 1 day after ecdysis to the 5^th^ instar) were injected with GFP labeled *Pa* ^ATCC43949^ (using the *gfp*-expressing plasmid pHC60). After 4 hours the insects were bled (~100 μl) from the cut dorsal horn. Monolayers were formed on a coverslip from a suspension of ~5 x 10^6^ hemocytes ml^-1^ in Grace’s insect medium (GIM) (Sigma), and observed in a Zeiss laser-scanning confocal microscope.

### Biofilm visualisation

Visualisation of surface-associated biofilms was done by inoculating a chambered microscopy slide (μ-slide 8 well, Ibidi) with 300μl of mid log *Pa* ^ATCC43949^ containing the *gfp*-expressing plasmid pHC60, grown with aeration at 28°C in LB. Static cultures were then incubated for up to 24h in either 28°C in normal air, at 37°C in 5% C0_2_ or at 37°C in normal air. Fluorescent images from washed slides were obtained using an inverted confocal microscope (Zeiss LSM510, 40x oil immersion objective).

### Accession Numbers

NCBI (SRP045656) accession contains the experimental metadata and the individual fastq files for the triplicated data from the RNA-seq experiments.

## Results

### Temperature tolerance of *P*. *asymbiotica* strains

**Fig 2:**
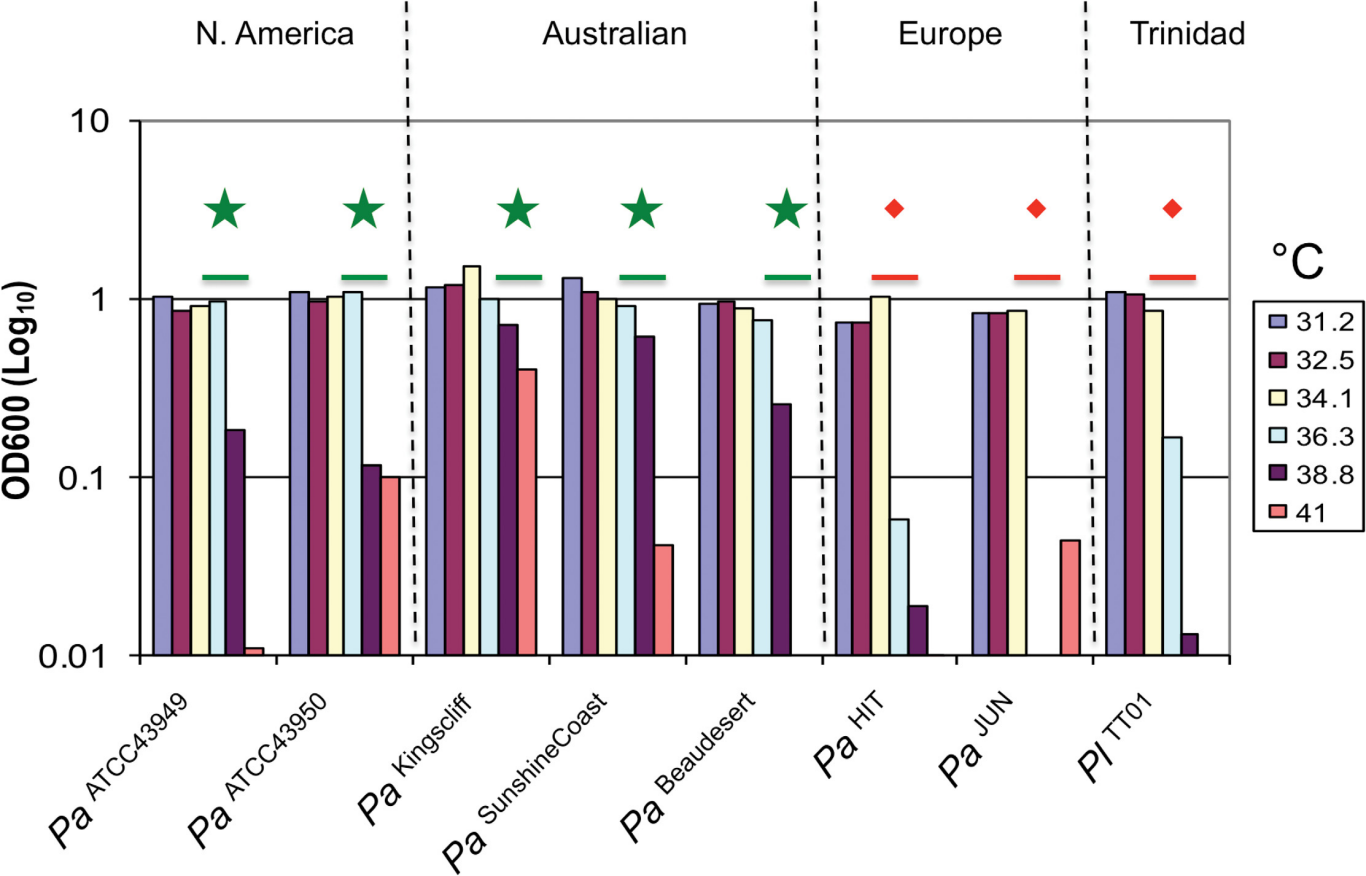
Clinical *Photorhabdus* isolates are able to survive exposure to higher temperatures than most non-clinical isolates. The optical density achieved by representative strains after overnight growth in static conditions (at 28°C in LB medium) after prior 18 h exposure to a range of temperatures. A range of clinical (N. American and Australian) and non-clinical (European) strains of *P*. *asymbiotica (Pa)* were tested, and the well-studied *P*. *luminescens* strain (*Pl* ^TT01^) was included for comparison. Green stars and red diamonds indicate thermal tolerance and intolerance respectively. *Pa* strain designations are indicated as superscripts.

We examined the ability of various *Photorhabdus* strains to survive an 18h exposure to various temperatures and their subsequent recovery and growth under static growth conditions at 28°C in LB medium (Fig 2). We also compared their growth dynamics after a shorter pre-exposure period of 4h (S1 Fig). We note that increasing the challenge temperature increased the lag times for recovery. The *P*. *asymbiotica* strains showed relatively short recovery times after exposure to 38.8°C compared to the temperature “intolerant” strains. Nevertheless there was great variation in the tolerance of strains. For example while *Pa* ^ATCC43948^ was not seen to recover at all after exposure to 38.8°C (within the time frame tested), the *Pa* ^Goldcoast^ strain was not affected even by 45°C exposure for 4h. The reason for this temperature induced lag is the beyond the scope of this paper and the subject of a subsequent publication. Nevertheless it should be noted that while all clinical *P*. *asymbiotica* strains grow readily in rich LB medium during continued exposure to 37°C (from a low inoculum), none of the other strains tested here could sustain growth in this temperature regime.

### Temperature dependent differences in RNA abundance

If *P*. *asymbiotica* is specifically adapted to infect a mammalian host it is likely that the endothermic host temperature is a signal for the production of mammalian relevant pathogenicity determinants. To investigate this we performed strand-specific RNA-seq using triplicate biological samples of *Pa* ^ATCC43949^ RNA isolated from exponential phase cultures grown in LB medium with aeration. To facilitate mapping of the RNA-seq reads we chose to focus our studies on *Pa* ^ATCC43949^, as the genome has been fully sequenced and closed [16]. The annotated genome contains 4401 predicted ORFs on the chromosome and 29 annotated ORFs on the pPAU1 plasmid. Differential gene expression was analysed using DESeq and significant changes determined using cut-off scores of +/- 1.95 log_2_-fold change, P-value ≤ 0.1 and minimum base mean read depth of 10 in the reference condition (S1 Data). This analysis found only 100 transcripts (with 45 of unknown function) significantly up-regulated at 37°C compared to 28°C (S1 Table), and 83 down regulated (with 31 of unknown function) (S2 Table).

**Fig 3:**
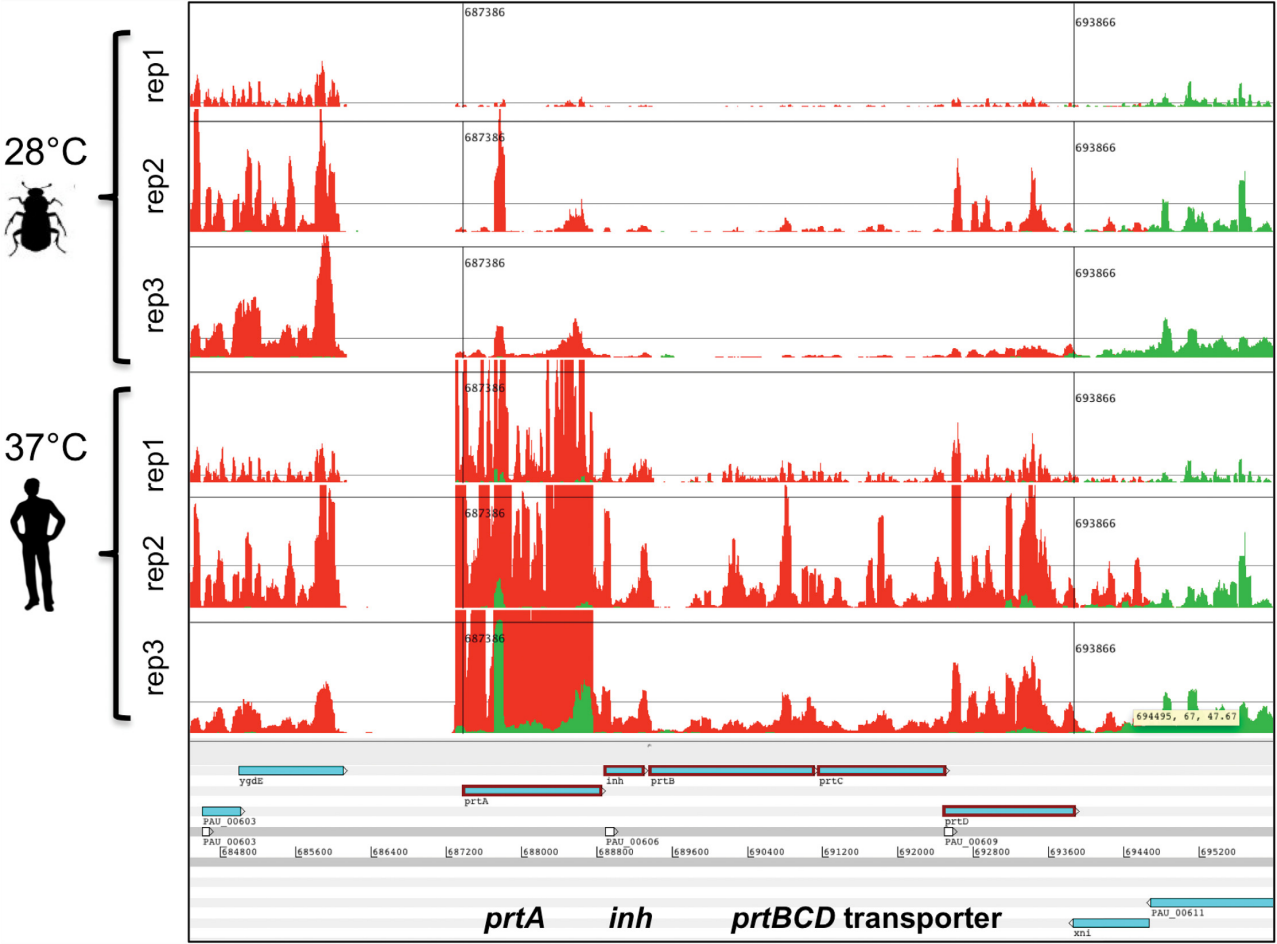
The secreted metalloprotease PrtA is one of the most highly up regulated genes at 37°C. An Artemis view of mapped RNA-seq data showing higher transcription of the *prtA* gene at 37°C compared to 28°C. A slight increase is also seen in the associated ABC transporter genes, *prtBCD*, and the predicted inhibitor gene *inh*.

Of the 37°C up-regulated chromosomal genes, the most strongly induced encode products involved in the acquisition and metabolism of amino acids and peptides. These include genes for a secreted metalloprotease *prtA* (a putative virulence factor, see discussion) and its exporter complex *prtBCD* (Fig 3), four solute-binding proteins of OppA-like oligopeptide ABC transporters, a predicted amino acid ABC transporter (PAU_03993) and several proteases/peptidases (D-aminopeptidase, enhancin and a putative thermostable carboxypeptidase (PAU_00753). We also note the up regulation of several genes involved in linking amino acids to central metabolism (*asnA*, AdoMet-synthetase and *aroG*) and vitamin biosynthesis (*cbi* genes). Several genes encoding virulence factors are significantly up regulated at 37°C including the potent *mcf1* toxin [28], several genes of PVC-unit1 [32] and its putative effectors (PAU_02805/6), the insect toxin *pirB* [33], a putative “invasin” containing operon (PAU_02531–37) and a *sepC* toxin-like gene (PAU_0214). Secondary metabolism genes showing significantly higher expression at 37°C include; an operon containing genes similar to those used for the production of the antibiotic Fortimicin (PAU_01180–4), the *luxCD* substrate recycling genes, two NRPS-related genes (PAU_02219–20) and the *isnAB*-like genes (PAU_01720–21) used for the synthesis of the aglycon precursor of rhabducsin [30]. Several genes encoding chaperone and heat shock proteins (*clpB*, *htpG*/*hsp90*) were also significantly up-regulated at 37°C. Whilst DESeq did not indicate a significant increase in transcription (given the cut off criteria) of the chaperonin genes *groL* and *groS*, these genes do show some up regulation, although they are already amongst the most highly expressed transcripts at both temperatures suggesting their importance (S2 Fig). Interestingly the most abundant transcripts overall are from the tmRNA *ssrA*-PAU_01268 nucleoprotein complex. While these do not vary significantly with temperature the very high levels of expression suggest a high demand for re-cycling stalled ribosomes. We also note increased abundance of PAU_01563 mRNA encoding a predicted stress-response protein.

The majority of chromosomal genes down-regulated at 37°C encode products involved in the uptake and metabolism of carbohydrates (e.g. *tctABC*, *lamB*, *mal*-genes, *pfkF*) and nucleotides (e.g. *purD*, *purH purL*, *purE*, *purK*, *guaA*, *guaB* and xanthine/uracil permease PAU_00190). Several genes predicted to encode regulators of carbohydrate metabolism were also strongly down at 37°C, including the carbon starvation protein (*cstA*), a putative *ner*-like sugar fermentation stimulation protein regulator (PAU_03919) and the tricarboxylate transport two-component system (*tctDE*). Only a few putative virulence genes are down regulated at 37°C; those that are include those whose products show some similarity to type VI secretion system proteins (PAU_02313–02321), the *xaxA* insect toxin gene and a *papH* fimbrial regulator homologue. Finally an uncharacterised operon (PAU_03768–03772) that contains a homologue of the *Streptomyces afs* gene (involved in *Streptomyces* quorum sensing) is also strongly down regulated.

All *P*. *asymbiotica* strains so far analysed carry a plasmid with homology to pPAU1 [16], which shows limited homology to the pMT1 plasmid of *Y*. *pestis* essential for flea vectoring. This plasmid has not yet been seen in any strains of *P*. *luminescens* and *P*. *temperata*, and it is therefore tempting to speculate that it encodes determinants for human pathogenicity. A previous proteomic analysis comparing the secretome of *Pa* ^ATCC43949^ at 28°C and 37°C revealed a significant decrease in the abundance of the plasmid-encoded protein pPAU_0028 at 37°C. This protein has an unknown function however it does contain a bacterial Ig-like domain also found in invasins [34]. Nevertheless we could detect no significant difference in the transcription of any of the plasmid genes between the two temperatures using DESeq (S1 Data). Decreased secretion of the pPAU_0028-encoded protein is therefore a consequence of post-transcriptional changes.

Visualisation of the mapped RNA-seq reads in Artemis (S2 and S3 Data) revealed one of the most highly expressed transcripts is a putative sRNA (nt 721652–721861) which is in the same genomic location and has 85% nucleotide identity to *csrB* in *P*. *luminescens*. We also note expression of several other novel sRNA with homology to known sRNA in the rfam database. A transcript that shows homology to the spot42 sRNA, which regulates carbohydrate metabolism and uptake in *E*. *coli* is expressed at both temperatures in LB during exponential growth from the forward strand (nt 336528–336582) upstream of PAU_00301. A putative *gcvB* sRNA is also expressed at both temperatures from the reverse strand (nt c683067-683243) upstream of *gcvA*. The sRNA *gcvB* negatively regulates the translation of mRNA encoding solute-binding proteins of amino acid and peptide ABC transport systems in enteric bacteria [35]. Putative sRNA with homology to *glmY* (nt c1505935-1506092) and *glmZ* (nt c4794954-4795177) are also expressed at both temperatures. In *E*. *coli*, these sRNA act in a regulatory cascade to control the level of intracellular glucosamine-6-phosphate [36]. These novel transcripts were not included in the genome annotation used to perform the DESeq analyses, but visualisation of the plots in Artemis suggests no obvious deviation in any putative sRNA expression levels at the two temperatures.

We obtained further validation of the RNA-seq dataset using qRT-PCR to confirm the changes of several distinct transcripts as exemplars, using RNA extracted from additional independent biological replicates to add confidence to our interpretation of the data (S3 Fig).

### Temperature dependent differences in protein abundance

Changes in relative mRNA abundance do not always correlate with protein abundance due to post-transcriptional regulation and differential protein stability/turnover. Therefore we also conducted triplicate 2D-DIGE proteome analyses [37] of protein extracted from cell lysates derived from the same exponential phase cultures used for RNA extractions. Six 2D-DIGE gels (three biological replicates for each temperature against pooled standard) were run and protein spots were identified and quantified using DeCyder. Graphical images of the individual 2D-proteomics gels, the DIGE-comparison gel and the DeCyder statistical analysis for each relevant protein spot are included in supplementary data (S5 Data). This analysis found 30 protein spots significantly more abundant at 37°C and 25 spots significantly less abundant at 37°C compared to 28°C (> = -1.8-fold difference with a T-test P value <0.01). Using LC-MS/MS we were able to unambiguously identify 15 proteins more abundant at 37°C than 28°C and 15 less abundant at 37°C than at 28°C (S3 Table). Proteins more abundant at 37°C were seen to be involved in chaperone activities (GroEL, GroES, DnaK ClpB), oxidative stress resistance (Bcp, Gst), autoinducer AI-2 catabolism (LsrF), lipopolysaccharide (LPS) biosynthesis (GmhA), iron acquisition (PAU_03286) and a secreted Asparaginase (AnsB). The majority of proteins less abundant at 37°C are involved in metabolic processes. These include proteins for maltose transport (MalE); purine metabolism (PurH, PurL, GuaAB); gluconeogenesis/anapluerosis (PckA, AspC, SfcA) and glycine detoxification/one carbon pool metabolism (GcvT). A protein potentially involved in immune evasion, a homologue of the lipid-A modification/ AMP resistance gene ArnA, also showed lower abundance at 37°C.

### Temperature dependent differences in metabolism and stress tolerance

Our results from the RNA-seq and proteomics experiments suggest that *P*. *asymbiotica* alters its metabolism in response to host temperature. Whilst *P*. *asymbiotica* may be cultured easily at 28°C and 37°C in LB with aeration, our initial attempts to cultivate this strain in minimal media were problematic at 37°C. We therefore tested the ability of *Pa* ^ATCC43949^ to use different carbon and nitrogen sources and to tolerate a range of pH and osmolytes at the two host temperatures using the Biolog Phenotype Microarray system. We included the insect host restricted *Pl* ^TT01^ as a comparator. In order to use the Phenotype Microarray system it was necessary to make certain adaptations to the standard protocols. We replaced the standard IF0A media (Biolog) with a modified M9 salts medium (see methods) in plates PM01, PM02, PM3B, PM06, PM07 and PM08 as we found IF0A to be inhibitory to *Photorhabdus*. Furthermore it was necessary to supplement the carbon plates (PM01 and PM02) with a low concentration of Casamino Acids (0.05% w/v), as a mixed nitrogen source, as in these studies *Photorhabdus* could not utilise inorganic nitrogen. In the nitrogen (PM3B) and peptide plates (PM06, PM07, PM08) we supplemented 20 mM D-mannose as a carbon source. In addition, it was necessary to supplement the carbon, nitrogen and peptide plates with a 1x RPMI vitamin mix to support respiration at 37°C. Individual and combined (mean) respiration data replicates (per well of each plate) were separately visualised in a series of plots using the *lattice* package in R. From these, it was possible to obtain an overall, visual assessment of general trends of compounds that support respiration. For simplicity we have summarised the data as a qualitative analysis in S4 to S9 Tables, highlighting substrates that supported respiration above the level of the negative control wells.

This analysis showed that at 28°C both *Pl* ^TT01^ and *Pa* ^ATCC43949^ could utilise a diverse range of compounds as a sole carbon source for respiration at 28°C. However at 37°C *Pa* ^ATCC43949^ was seen to only utilise D-mannose, uridine, adenosine, inosine, L-serine or glycyl-L-proline as a sole carbon source. At 28°C, *Pa* ^ATCC43949^ could not utilise the majority of amino acids as a sole nitrogen source. Exceptions include L-glutamate, L-glutamine, L-tyrosine, L-serine and L-tryptophan. However when *Pa* ^ATCC43949^ was incubated at 37°C this list became further restricted to L-glutamate, L-glutamine and L-tyrosine. Interestingly we noted that *Pa* ^ATCC43949^ could use L-aspartate and L-asparagine as a sole carbon source at 28°C but not at 37°C while conversely it could use these amino acids as a sole nitrogen source at 37°C but not at 28°C. Other organic nitrogen sources that could be used as a sole nitrogen source by *Pa* ^ATCC43949^ at 28°C included N-acetyl-D-glucosamine, adenosine, cytidine and xanthine, with only xanthine being used at 37°C. The Phenotype Microarray analyses suggested *Pa* ^ATCC43949^ is only able to utilise a restricted subset of di- and tri-peptides as a sole nitrogen source at either temperature.

The RNA-seq and proteomics analyses show an increase in transcription and/or abundance of chaperones and stress response proteins at 37°C. To determine if the higher temperature affects the stress tolerance of *P*. *asymbiotica* we performed triplicate biological replicates of the Biolog osmolyte and pH plates (PM09 and PM10). The standard Biolog media recommended for these plates (IF10) is a similar composition to LB and therefore we could use this without additional supplements. *Pa* ^ATCC43949^ became more sensitive to osmotic stress and tolerated a narrower range of pH at 37°C (S10 and S11 Tables).

### Temperature dependent differences in oxygen requirement and bioluminescence

Our RNA-seq analysis showed a significant increase in the expression of *luxCD*, which encode the enzymes that recycle the aldehyde substrate for luciferase. We tested the light emission from *Pa* ^ATCC43949^, *Pa* ^Kingscliff^ and *Pl* ^TT01^ at 28°C and 37°C in LB medium with aeration. Light emission was shown to increase at 37°C for both clinical strains, which correlates with the increase in *luxCD* mRNA abundance. A surprising observation is that the temperature intolerant *Pl* ^TT01^ also emitted high levels of light up to around 10h when cultured at 37°C before apparently succumbing to temperature stress (S4 Fig). While the biological significance of bioluminescence in *Photorhabdus* remains unclear, conservation of the *lux* operon in all strains so far isolated argues for a selective advantage in nature. It is possible that bioluminescence protects *Photorhabdus* against ROS because the luciferase reaction consumes high levels of molecular oxygen. We therefore tested the ability of *P*. *asymbiotica* to grow in different oxygen environments at the two temperatures on various types of solid media. This showed that *P*. *asymbiotica* is unable to grow under full anaerobic conditions at either temperature on any media tested. Growth under oxygen-limited conditions (microaerobic) is poor compared to aerobic (normal air) at 28°C, and only full aerobic conditions support growth at 37°C (S5 Fig). We suggest that the increased consumption of oxygen by the luciferase reduces the availability of oxygen at 37°C thereby preventing microaerobic growth.

### Temperature dependent differences in secondary metabolite production

**Fig 4:**
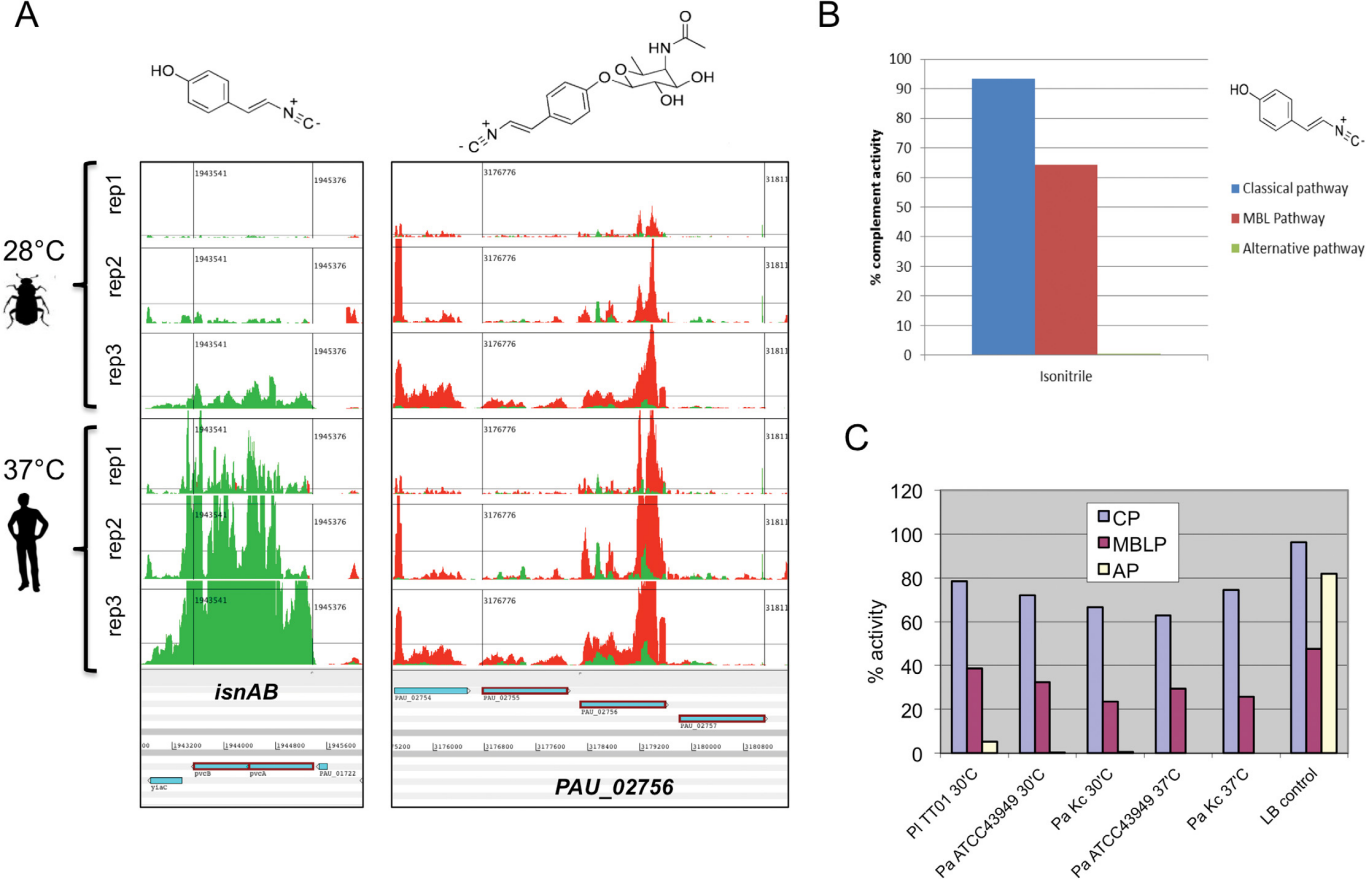
The expression and function of the *Photorhabdus* natural product rhabduscin. (A) Artemis views of the RNA-seq reads of the three replicates mapped onto the *Pa* ^ATCC43949^ operons responsible for rhabduscin synthesis. The *isnAB* genes are responsible for synthesis of the aglycon precursor shown above the left panel. The PAU_02755–7 genes encode glycosidase enzymes that add the sugar groups to produce the final rhabduscin molecule. Note PAU_02756 is unique to the *P*. *asymbiotica* (replaced by a transposase in *Pl* ^TT01^) and so the final *Pa* ^ATCC43949^ rhabduscin structure from *Pa* ^ATCC43949^ may not be the same as that shown from *Pl* ^TT01^ (above the right panel). (B) The purified aglycon precursor of rhabduscin (shown above the key) is able to completely inhibit the human alternative complement pathway. (C) Cell free supernatants from *Pa* ^ATCC43949^ (PaATCC43949), *Pa* ^Kingscliff^ (Pa Kc) and *Pl* ^TT01^ (Pl TT01) can all inhibit the human alternative complement pathway (AP). Note the classical (CP) is only partially inhibited, while LB alone also inhibits the Maltose binding lectin (MBLP) pathway to some extent.

All *Photorhabdus* genomes so far examined encode a large number of genes for enzymes dedicated to the production of secondary metabolites, suggesting natural products (NPs) are important to the EPN life cycle [38]. For example *Pl* ^TT01^ encodes over 24 clusters comprising approximately 8% of the genome. Secreted NPs produced by *Pa* ^ATCC43949^ were compared from the supernatants of stationary phase cultures grown in LB with aeration for 48 h at 28°C and 37°C, using LC-MS/MS. This revealed very few differences in the overall profile of NPs produced at the two temperatures. Of the known NPs that have been characterised to date, we detected a 2.6-fold increase in the production of GameXPeptide [39] and a 1.5-fold increase in iso-propyl-stilbene (IPS) [40, 41] at 37°C compared to 28°C. While the DESeq analysis did confirm up regulation of transcription of the GameXPeptide synthesis gene (*gxpS*) at 37°C (S6 Fig and S1 Table) the slight increase in the transcription of the IPS synthetic genes fell below the 1.95 log2fold cut off (S7 Fig). The natural product rhabduscin, produced by several *Photorhabdus* and *Xenorhabdus* strains, was recently shown to inhibit the insect Phenol-Oxidase (PO) cascade [30]. Furthermore homologues of key rhabduscin synthetic genes, *isnAB*, can also be identified in many diverse bacteria (S4 Data). While RNA-seq showed a significant increase in the expression of the *isnAB*-like rhabduscin synthesis genes at 37°C (Fig 4A), we did not detect increased secretion of rhabduscin itself. Crawford et al [30] demonstrated that the majority of rhabduscin localised to the cell surface, which may explain why we did not detect it directly in the clarified supernatants tested. We hypothesised that the decoration of the cell surface with this PO inhibitor might also afford protection against components of the human innate immune system analogous to the insect PO cascade. We therefore investigated whether heterologously produced and purified aglycon precursor of rhabduscin could offer protection against human complement, which like PO also constitutes a serine protease cascade. Our results confirmed that the aglycon precursor alone (synthesised by IsnAB) does act as a potent inhibitor of the mammalian alternative complement pathway (Fig 4B). Furthermore cell-free supernatants from all *Photorhabdus* strains we tested were also able to specifically inhibit the alternative pathway of complement (Fig 4C) suggesting rhabduscin is released in sufficient quantities from the cell surface.

As complement is not the only means of killing bacteria by human serum we decided to also test the ability of *Photorhabdus* to resist killing by commercially available serum. This is an important prerequisite for the survival of *P*. *asymbiotica* during human infection. Unlike the *E*. *coli* control both *Pa* ^ATCC43949^ and *Pl* ^TT01^ could resist killing by 20% (v/v) commercially available human serum (S8 Fig). In addition we also tested the ability of *Pa* ^ATCC43949^ and *Pl* ^TT01^ to resist killing by pig and rabbit serum (S9 Fig). Interestingly while *Pa* ^ATCC43949^ was resistant to both, the growth of *Pl* ^TT01^ was strongly inhibited by pig serum suggesting *Pa* ^ATCC43949^ is tolerant to serum from a greater range of mammals.

### Temperature dependent differences in insect virulence

Our results show that growth of *Pa* ^ATCC43949^ at 37°C restricts its ability to utilise a range of nutrients and increases sensitivity to pH and osmolyte stress. It also leads to an increase in the production of specific virulence factors and secondary metabolites. To determine how these changes affect the ability of *P*. *asymbiotica* to infect insects, we performed standard infection assays of *Manduca sexta* larvae at 28°C and 37°C [28]. As expected, 100% of the cohort of 5^th^ instar larvae (n = 30) were killed within 48 h by an injected low dose of approximately 1000 cells at 28°C. Importantly however, when the infected insects were incubated at 37°C the bacteria became avirulent and the whole cohort survived for up to 7 days.

## Discussion

The overall goal of this research is to investigate the adaptations that have enabled specific members of the ubiquitous insect pathogenic genus *Photorhabdus* to increase their host range to include humans. Photorhabdosis is characterised by the development of an initial skin lesion on an extremity, presumably at the site of infection, with subsequent bacteraemia and the appearance of secondary skin lesions around the body. Bacterial load can become very high in patients who will then become seriously ill, requiring extensive antibiotic intervention. *P*. *asymbiotica* must necessarily be able to replicate at the core body temperature of 37°C to sustain a systemic infection and must also produce virulence factors sufficient to overcome mammalian immune responses. However, no less important is the need to acquire sufficient nutrients from the relevant human host cells/tissues to satisfy growth and reproductive requirements. It should be noted that we have discounted any influence of the nematode vector *Heterorhabditis gerrardi* [42], as we previously confirmed that it cannot survive above 32°C (data not shown), suggesting it is unlikely to penetrate into deep tissues of human hosts. It should be noted that the temperature of human skin is very variable but normally significantly less than 37°C. Therefore the temperature dependent phenotypic switching documented here may not be immediately relevant during the first stages of invasion.

### Temperature tolerance

The ability of the clinical strains to tolerate growth at 37°C or above is unusual in the genus as, with the exception of certain *P*. *luminescens* strains, the majority of *Photorhabdus* cannot replicate above 34°C. This is clearly a prerequisite for bacteraemia and the establishment of a sustained human infection. Previous MLST and a recent whole genome phylogeny analysis of eight strains of *P*. *asymbiotica* and three strains of *P*. *luminescens* (using predicted proteomes) suggest a monophyletic origin of the clinical strains and their pPAU1-like plasmids (data not shown). Nevertheless it cannot be ascertained from this study whether 37°C tolerance was inherent in the last common ancestor of all the clinical strains, or whether it evolved independently on the different continents. The identification of clinical isolates from USA, Australia and Nepal (*Pa* ^Nep^) and a clinical isolate-*like* strain from Thailand (*Pa* ^Thai^), in addition to the two closely related temperature intolerant strains from northern Europe (*Pa* ^Hit^ and *Pa* ^Jun^), does suggest the *P*. *asymbiotica* phylogenomic group are globally widespread. The higher abundance of *Pa* ^ATCC423949^ heat shock chaperone at 37°C is not surprising, although at this point it is unclear if these levels indicate a stress-response *per se* or an appropriate temperature specific induction. However the decreased tolerance to both salt stress and a wider pH range does suggest a lower integrity of the cell membrane and/or an impairment of osmotic homeostasis at the higher temperature. Importantly the observation that *Pa* ^ATCC423949^ becomes avirulent to *M*. *sexta* at 37°C suggests that it is using temperature as a cue to orchestrate changes in transcription and translation appropriate for human infection but inappropriate for insect virulence. The alternative hypothesis that the higher temperature causes dysfunction of key proteins or pathways is unlikely given their ability to cause human infection.

### Immune evasion

It is generally accepted that insect immune systems share a great deal in common, both mechanistically and genetically, with the innate immune system of mammals. Upon entry into the human host *P*. *asymbiotica* must be able to resist immediate killing by both humoral and cellular arms of the innate immune system.

#### Humoral immunity

We have shown that both *Pa* ^ATCC43949^ and the insect host restricted *Pl* ^TT01^ can resist killing by humoral components of human serum. This indicates that whatever virulence factors *Photorhabdus* evolved to resist killing in insect blood serum effectors are also sufficient to protect against the human equivalents. We have shown for the first time that the Phenol-Oxidase inhibitor rhabduscin [30] is also able to inhibit the alternative pathway of human complement activation. The up regulation of the rhabduscin synthesis genes (*isnAB*) at 37°C suggests that they need increased levels to ensure protection against the complement system. That Rhabduscin is active against both insect (33) and mammalian (this work) immune cascades implies a similarity of action, and perhaps a common evolutionary ancestral link between the analogous PO and complement systems. We also note that transcription of the genes responsible for the synthesis of the iso-propyl-stilbene (IPS) is slightly increased, accompanied by an increase in the levels of IPS itself. The IPS can act as a Gram-positive antibiotic [43, 44], is necessary for symbiosis [45] and can inhibit soluble epoxide hydrolase [46], functions which would be relevant in the insect host life cycle. Interestingly it also shows good activity against psoriasis via suppression of the immune system [47]. Like rhabduscin, this is suggestive of a natural product, evolved to act against insects, being re-deployed against the mammalian immune system. The observed reduction in the levels of the ArnA bifunctional polymyxin resistance protein at 37°C appears counter intuitive. In *Salmonella* ArnA changes the charge on the LPS Lipid-A antigen, which protects against killing by host cationic antimicrobial peptides (AMPs) and is up regulated when the bacterium moves from the environment into a human host. However, given that our findings represent relative levels between insect and human relevant temperatures, the apparent reduction in ArnA abundance at 37°C may be a reflection of very high levels required in an insect infection. This may be the consequence of the need to defend against the strong reliance by insects on high levels of AMPs [48, 49] and might also be the reason why insect infection fails at 37°C. However it should be noted that in *Salmonella* the *arn* operon is required when strains are administered orally to mice but not when they are given via the intraperitoneal route [50]. Furthermore in *Yersinia pseudotuberculosis*, the *arn* operon has not been shown to give any advantage in mice infections [51]. *P*. *asymbiotica* also encodes Ail and PagC homologues (PAU_02601 and PAU_02047) which afford some protection against AMP activity [52] although their expression is not significantly affected by temperature.

#### Evasion of cellular immunity

The facultative intracellular invasion phenotype of macrophages likely represents a mechanism to evade mammalian specific immune mechanisms, which is not relevant in the ancestral insect host infection cycle (S10 Fig). We speculate that the up regulation of an uncharacterised operon containing an “invasin” gene homologue (PAU_02531) might be involved in this process. We also see transcriptional up regulation of a carbonic anhydrase gene (PAU_00820) at 37°C. These enzymes have previously been shown to be important in the intracellular survival of other bacterial pathogens although their precise role is unclear [53]. We do not see any up regulation of the second type III secretion operon [16] which we have previously speculated may be involved in intracellular invasion, suggesting exposure to host cells may be required. Interestingly, the transcription of several flagella biosynthesis genes is up regulated at 37°C, which is seen in other human pathogens such as *Salmonella*. As we see little up regulation of the majority of known toxin gene homologues it suggests that the expression levels relevant to an insect infection are also sufficient for a human infection. A notable exception is the increase in transcription of the potent *mcf1* toxin (PAU_03379) [28]. Previous studies have shown that this pro-apoptotic toxin is active against both insect and mammalian cells in tissue culture [54], suggesting the bacteria are deploying it in a similar way to combat either insect or human cells. We also observed the transcriptional up regulation of several other genes encoding known toxins at 37°C which might be used in either defence against the mammalian cellular immune system or tissue invasion. These include the *pirB* toxin (PAU_03717) [55] and the *Photorhabdus* Virulence Cassette genes (specifically PVCunit1) [32]. The up regulation of these cytotoxins is consistent with previously published findings that despite intracellular invasion, *P*. *asymbiotica* will ultimately cause apoptosis of the invaded cells in tissue culture [56]. It should be noted that many virulence factor genes can be observed to have a reasonable level of transcription during exponential growth (compared to the genome average) at both 28°C and 37°C and here we only highlight those with increased expression at 37°C. The failure to detect any mammalian-specific toxins in a previous screen [13] suggests that from the point of view of immune evasion, certain insect evolved virulence factors might also be deployed against humans. Furthermore, several of the changes in expression and protein abundance discussed below in relation to nutrition, such as the strong induction of *prtA* encoding a secreted metalloprotease, could also play a vital role in overcoming both humoral and cellular immunity.

### Other secondary metabolites

*Pa* ^ATCC43949^ shares only 15 of its 25 known operons for natural product synthesis with *Pl* ^TT01^ suggesting niche or host specific roles for many of these. In addition to IPS and rhabduscin we also see significant increase in transcription of the non-ribosomal-peptide-synthesis gene *gxpS* (PAU_03067), which produces the natural product GameXPeptide. While the exact function of this is unknown preliminary work suggests a role in which immune suppression (unpublished).

### Changes in metabolism as a nutritional virulence strategy

Previous studies have indicated that *Photorhabdus* employs a metabolic switch to regulate its transition from insect pathogen to nematode mutualist [45]. The significant reduction in the range of metabolic capabilities of *Pa* ^ATCC43949^ at 37°C could be the result of an adapted and regulated response, a consequence of unintended pathway failures, or a combination of both. Nevertheless, when taken together the RNA-seq, proteomics and phenotype microarray data is suggestive of a regulated response.

**Fig 5:**
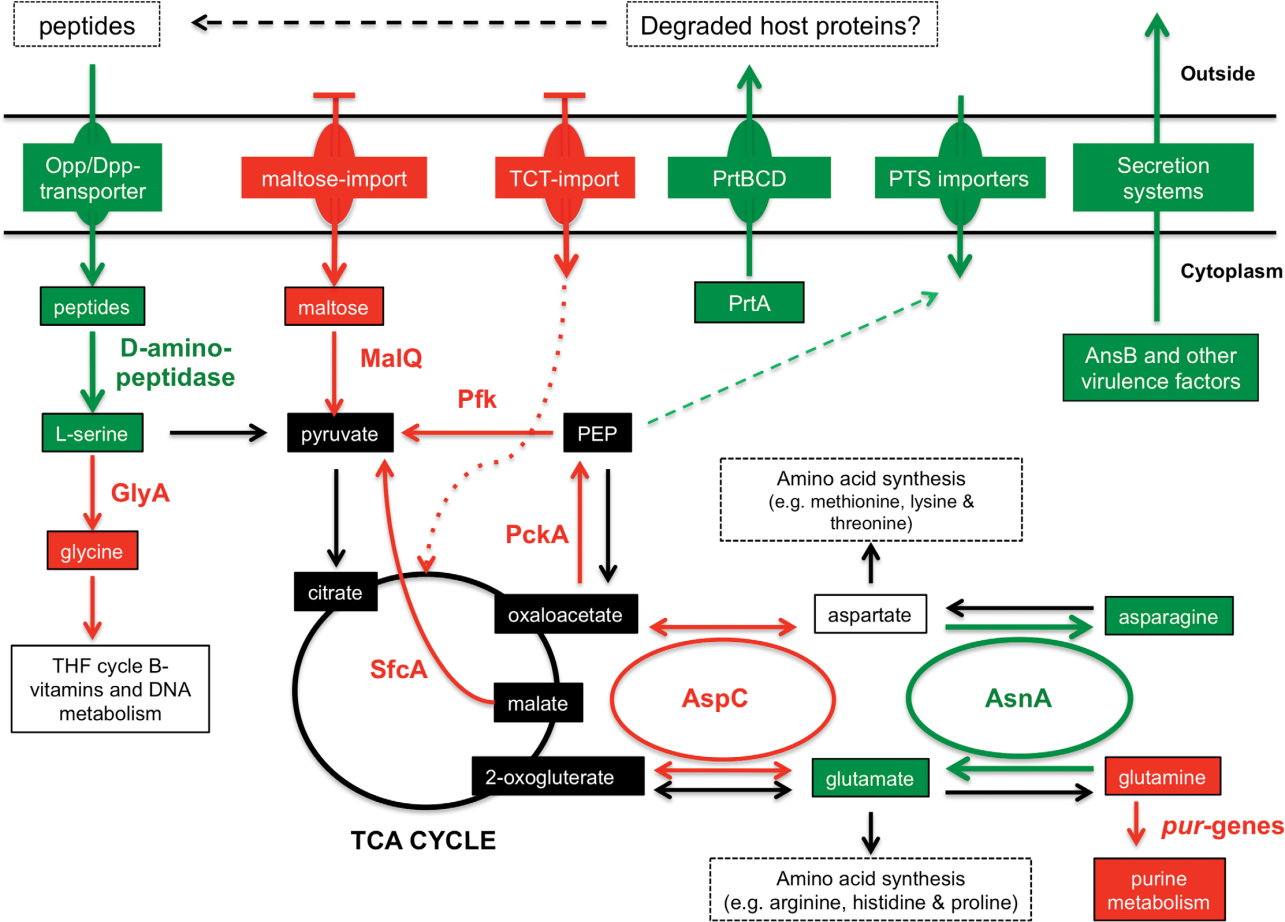
A schematic summarising some key differences in metabolism at 37°C compared to 28°C, centred on glutamate/asparagine metabolism and the TCA cycle. This model is predicted by integrating data from the RNA-seq, proteomics and phenotype microarray studies. Intermediates (boxes) and pathways (arrows) predicted to be down regulated at 37°C are in red while those up regulated are in green. Data suggests TCA cycle intermediates (back boxes) would be relatively isolated from glutamate/asparagine metabolism and could be maintained via the conversion of L-serine into citrate via pyruvate. Black arrows indicate certain potential enzyme pathways that are present and predicted to be unchanged at 37°C. The data suggests a central role for imported peptides and amino acids in metabolism at 37°C. Opp/Dpp represent oligo- and di-peptide importers, TCT represents tricarboxylic acid and PEP is Phosphoenolpyruvate.

One possible reason for the observed changes is that by reducing the levels of enzymes involved in the metabolism of pyruvate, PEP and acetyl-CoA, the cells could deliberately slow down metabolite flux feeding into the TCA cycle at 37°C. When taken together with the reduction in the one-carbon pool NADPH production implied by the data, it may indicate that the bacteria are attempting to counter unavoidable and undesirably high metabolism arising from increased enzyme rates at the higher temperature. Such an explanation would imply that *Pa* ^ATCC43949^ is not well adapted to the mammalian temperature regime. Nevertheless, we argue that a more likely interpretation of the data is that the bacteria are using 37°C as a cue to deploy a “nutritional virulence” strategy, as seen in several other human pathogens. This is a strategy by which pathogenic bacteria selectively use certain metabolites in order to deprive the host of molecules necessary for resisting infection [57, 58]. We will expand on this hypothesis below. Fig 5 illustrates some of the relevant metabolic pathway changes we discuss.

#### Carbon metabolism and energy production

*Pa* ^ATCC43949^ is able to use relatively few substrates as a carbon source at 37°C, limited to N-acetyl-D-glucosamine (GlucNac), D-mannose, uridine, adenosine, inosine, L-serine and glycyl-L-proline. As the monomeric unit of chitin and a component of hyaluronan, major connective tissue components of insect and man, GlucNac would be a relevant carbon source in both hosts. Consistent with this, the GlucNac PTS system and metabolism genes are expressed at similar levels at both temperatures. Interestingly glycyl-L-proline is a degradation product of collagen and so therefore likely represents an abundant resource during a mammalian infection. The ability to utilise mannose as a carbon source at 37°C indicates adequate transport rates via the mannose PTS and efficient conversion to glycolytic intermediates. Mannose is found at around 50 μM in human plasma and in the cell cytoplasm where it is kept at a steady concentration for use primarily in N-glycosylation of proteins suggesting it may be a reliable carbon source [59]. Preferential depletion of mannose could also therefore interfere with N-glycosylation of host proteins. Conversely trehalose is readily used a carbon source at 28°C but does not support respiration at 37°C. As the primary storage sugar in insects, trehalose is therefore likely not relevant for mammalian infection. The observed inability to use maltose, maltotriose / maltodextrin as carbon sources at the elevated temperature correlates with the down-regulation of maltose transport and metabolism genes (PAU_00364–7, 00376–7). Similarly, the inability to use TCA intermediates at 37°C correlates with the strong down-regulation of the *tctDE* two component system and the corresponding *tctABC* importer genes at 37°C. This would likely lead to a reduction of the flow of extracellular tricarboxylates directly into the TCA cycle. In addition, utilisation of TCA intermediates as carbon sources requires synthesis of pyruvate from oxaloacetate via the concerted activity of phosphoenolpyruvate carboxykinase (PckA) and pyruvate kinase (PykF) and/or the conversion of malate to pyruvate by malic enzymes (SfcA and MaeB). We note a reduction in the levels of PckA (PAU_00083) and the NAD^+^-dependent malic enzyme, SfcA (PAU_02896) in addition to the down-regulation of *pykF* transcription at 37°C. The expression and abundance of *maeB* (PAU_01820), encoding the NADP^+^-dependent malic-enzyme, is not altered but alone this isoform might not provide sufficient pyruvate to sustain TCA function when TCA intermediates are the sole carbon source [60].

It is also possible that the down-regulation of *pykF* enables high rates of transport via the PTS systems. In previous temperature adaptation experiments in which *E*. *coli* mutants were selected for increased growth rate at 42°C in glucose supplemented minimal medium, a common mutation involved the down-regulation of PykF [61]. The authors hypothesized that PykF down-regulation enables faster glucose uptake by virtue of an increased intracellular pool of PEP, which is then available for use by the PTS system to drive glucose uptake. In *Pa* ^ATCC43949^ it is possible that such a strategy increases the pool of PEP for use by PTS systems for the import of preferred substrates such as GlucNac and mannose. Alternatively sufficient pyruvate may be provided by the deamination of L-serine (see below)

Interestingly L-serine is readily used as a carbon source for respiration at both temperatures; however this is unlikely to be via serine-driven one-carbon metabolism based NADPH generation at 37°C as *glyA* (PAU_01354) transcription is reduced. A drop in GlyA activity would reduce conversion of L-serine to glycine with a concomitant lowering of the conversion of THF to 5,10-methylene-THF. L-serine would therefore be available for shunting into pyruvate metabolism pathways (and ultimately into the TCA cycle via Acetyl-Co into citrate). Consistent with this we note that transcription of the relevant enzymes SdaA (PAU_01839) and IlvA (PAU_04161) are unaltered at 37°C. Furthermore, the conversion of 5,10-methylene-THF to 5-formyl-THF would be depressed by a drop in the production of GcvT (PAU_01159). Unaltered levels of YgfA (PAU_01153) could regenerate the pool of 5,10-methylene-THF from any 5-formyl-THF formed. This could then be converted directly into 10-formyl-THF for the regeneration of NADPH from NADP+, until the 5, 10 methyl-THF became limiting. Unlike L-serine, these bacteria cannot use D-serine as a carbon source at either temperature. Consistently, at 37°C we see a strong down regulation of transcription of the D-serine importer *dsdX* (PAU_02599) and *dsdA* (PAU_2598) genes. DsdA is an enzyme used for the conversion of toxic D-serine into pyruvate and ammonia.

Our model suggests there would be an increase in the pool of glutamate at 37°C (Fig 5), implied by the up-regulation of *asnA* (PAU_00048) transcription. AsnA catalyses the ATP-dependent conversion of aspartate to asparagine with the concurrent conversion of glutamine to glutamate. Furthermore, the observed down-regulation of the *pur*-operon genes would reduce the entry of glutamine into purine biosynthesis. The phenotype microarray data shows that both glutamine and glutamate can be used as both carbon and nitrogen sources at 28°C, although only as a nitrogen source at 37°C. Furthermore, aspartate and asparagine can be used as carbon but not nitrogen sources at 28°C, and vice versa at 37°C, which correlates with the decrease in AspC (PAU_02766) at 37°C. This would reduce the potential for entry of aspartate and glutamate into the TCA cycle. The combination of the down-regulation of AspC and up-regulation of AsnA would essentially isolate the TCA cycle intermediates from glutamate/aspartate metabolism. This would preserve TCA intermediate flux for energy production and free up glutamate/aspartate for nitrogen metabolism and the synthesis of other amino acids (Fig 5).

#### Nitrogen metabolism

Nitrogen sources that allow respiration at 37°C are restricted to glutamine, glutamate, asparagine, aspartate, tyrosine and xanthine and a range of di- and tri-peptides. Consistent with this strong dependence on relatively few amino acids and peptides, we observed the up-regulation of several key genes involved in amino acid / peptide acquisition and metabolism. These include a putative amino acid transporter (PAU_03993), a leucyl-aminopeptidase-T homologue (PAU_01923), a putative thermostable carboxypeptidase (PAU_00753), D-aminopeptidase DmpA (PAU_02203) and three solute-binding proteins of OppA-like oligopeptide ABC transporters (PAU_02032, 02337 and 02342). Interestingly we also see up regulation of a gene encoding a protein with an enhancin-peptidase like domain (PAU_02334). However the only identified substrate for enhancin is insect intestinal mucin, tempting speculation of an alternative mammalian target for this *P*. *asymbiotica* homologue. Importantly the gene for the secreted metalloprotease PrtA (PAU_00605) has one of the highest increases in differential expression level at 37°C (Fig 3). PrtA is used by *P*. *luminescens* during insect infection to degrade extracellular matrix components and destroy the midgut epithelium [62]. However, it has been suggested that PrtA is used to generate the amino acids required for nutrition during bioconversion of the insect cadaver, as it is only detectable 24 h after infection [63]. In a recent study, the heterologous expression of this PrtA in *Bacillus thuringinesis* increases both the virulence and final bacterial yield in insect blood [64] suggesting a potential additional role in immune evasion. This suggestion is also supported by the finding that PrtA can cleave six *M*. *sexta* immunity proteins [65]. Unfortunately our attempts to create a deletion of this operon have so far been unsuccessful.

#### Nucleic acid metabolism

Down regulation of *purDHKELNM* (PAU_00405–6, 00996–7, 01324, 01779–80) and *guaAB* (PAU_01826–7) transcription suggests that they cease *de novo* purine biosynthesis at 37°C. As we see no reduction in replication it suggests that they rely upon scavenging purines from the surroundings. This is consistent with the facultative intracellular invasion phenotype seen in mammalian tissue culture (S10 Fig), whereupon they would have access to the host cytoplasmic pool of purines. Furthermore, the ability to use the purines: adenosine, uridine and inosine as a carbon sources at both temperatures demonstrates they have efficient uptake systems for these nucleosides. Adenosine and xanthine can also act as nitrogen sources, although only xanthine is used as such at 37°C. Interestingly the activity of xanithine oxidase has been associated with T and B-cell responses so we may speculate that depletion of this purine may also constitute a mechanism to interfere with the immune response [66]. An increase in expression of *cys*/*cbi* and *cob*-genes suggests a greater requirement for Vitamin B_12_ synthesis at 37°C. Vitamin B_12_ is important as a co-enzyme factor in several metabolic proteins including class II ribonucleotide reductases, which catalyse the formation of deoxyribonucleotides from ribonucleotides (ADP, GDP, CDP and UDP) for DNA synthesis. Consistent with this is our observation that *Pa* ^ATCC43949^ could not respire at 37°C in a base of M9 minimal media unless we supplemented it with a vitamin mix, suggesting one or more vitamins become limiting at 37°C. Studies in *Salmonella* have shown that Vitamin B_12_ is synthesized *de novo* under anaerobic conditions to support otherwise poor growth [67]. In addition previous work in *Pseudomonas* has demonstrated that DNA replication is impaired during anaerobic growth in biofilms, due to a suppression of Vitamin B_12_ production, resulting in cell-elongation. When Vitamin B_12_ was provided exogenously DNA replication and growth dynamics were restored to normal [68]. These findings correlate with our observations of cellular elongation by *Pa* ^ATCC43949^ when grown under macrophage tissue culture conditions (S10 Fig). The increased expression of vitamin B_12_ *de novo* synthesis genes is consistent with an adaptation to reduced oxygen availability at 37°C (see below).

#### Amino acid metabolism

It has been argued that serine depletion from the host has the effect of impairing cellular activity, as animal cells significantly rely upon serine-driven one-carbon metabolism for NADPH generation [69]. Therefore a switch at 37°C to using L-serine for pyruvate production and TCA maintenance, and de-coupling glycolysis from TCA through the repression of PfkF (PAU_01920) and PckA (PAU_00083) might have the effect of diverting energy production through biochemistry that would simultaneously cripple the host cell energetics. It should be noted that we do not see any significant increase in the expression of the putative L-serine importer (PAU_03626) at 37°C suggesting import is not limiting at least in LB medium. As LB broth contains high levels of peptides and amino acids it is likely the observed increase in the expression of Opp and Dpp importers is sufficient to satisfy an increased requirement for L-serine.

Glutamine plays an essential role in lymphocyte cell survival and proliferation in low oxygen conditions (as found during inflammation). Furthermore, it is known that glutamine/glutamate depletion is related to an increase in susceptibility to infection [70]. *Helicobacter* has been shown to deplete glutamine in the gastric mucosa and in lymphocytes by using it as a primary energy source [71]. In addition glutamate uptake is known to promote meningococcal survival through a reduction of the neutrophil oxidative burst and by increasing its own glutathione production, as a defence against reactive oxygen species (ROS) [72]. The metabolic changes at 37°C in *Pa* ^ATCC43949^ would likely result in glutamine depletion from invaded host cells, potentially supressing both iNOS production and maintenance of the host cell TCA cycle, damaging their bio-energetic status. Furthermore as glutamine is used as a precursor for glutathione, its depletion would also leave lymphocytes themselves vulnerable to ROS damage.

At 37°C we see an increase in L-asparaginase II (AsnB) production. In *Salmonella*, this secreted enzyme converts host Asparagine into Aspartate and ammonia. This has the effect of supressing T-cell blastogenesis, cytokine production, proliferation and down-regulation of the expression of the host T-cell receptor [70]. Previous work has also confirmed the role of L-asparaginase in *Helicobacter* as a cell cycle inhibitor in non-immune tissues [73]. We note that the *Pa* ^ATCC43949^ AsnB protein also has a predicted type II signal peptide suggesting it is serving a similar role. Inhibiting T-cell responses in addition to macrophages and neutrophils is also likely to be important to *P*. *asymbiotica* survival. It is perhaps no coincidence that while the rhabduscin molecule can inhibit the alternative pathway of complement, that it has no effect on the antibody mediated classical pathway. Therefore prevention of T-cell stimulated antibody production may be vital for sustained infection.

#### Iron metabolism

Consistent with many other bacterial pathogens we see an up regulation of certain iron-acquisition related genes at 37°C. Previous studies have confirmed the importance of iron for *Photorhabdus* [74]. The Iron compound ABC transporter PAU_03286, CjrC outer membrane siderophore receptor (PAU_01824) and Bcp (bacterioferritin co-migratory protein—PAU_01793) are induced. Paradoxically we also see a down regulation of both the *fecI* iron stimulated ECF sigma factor and the *fecABCDE* iron (iii) di-citrate transporter genes however the relative expression levels of these genes are very low so the importance of this questionable. It is possible that decreasing the import of Fe^3+^ would lower cytoplasmic levels of Fe^2+^, alleviating the potential for spontaneous ROS generation, which would increase with temperature.

#### Oxygen metabolism

It should be noted that members of the *Photorhabdus* are particularly sensitive to ROS. They are routinely cultured either in the dark or with the inclusion of pyruvate into LB agar plates to prevent photo-activated ROS molecules from inhibiting their growth. Consistent with this, we see an increase in production of the Gst enzyme (PAU_01940) at 37°C, affording them additional protection against ROS. Furthermore the observed increase in the luciferase reaction would lead to a greater depletion of molecular oxygen, again lowering the potential threat of ROS damage. The increase in bioluminescence is likely the result of increased reaction kinetics of the LuxAB holoenzyme at the higher temperature although we also see a limited up-regulation of *luxAB* transcription, which falls below our significance criteria. However we do see a significant increase in the transcription of the *luxCD* genes, which produce enzymes required for cofactor recycling, presumably to compensate for the higher levels of bioluminescence. This raises the intriguing possibility that Photorhabdosis skin lesions might actually glow in the dark if bacterial numbers were sufficient, in a similar manner to the “Angels Glow” phenomena experienced by wounded soldiers at the Battle of Shiloh during the American Civil War [75]. Local oxygen levels may be further reduced by the up regulation of a putative secreted intradiol dioxygenase (PAU_03890), as this class of enzyme utilise molecular oxygen. The increase in transcription of a homologue of a vitamin K_2_ synthesis enzyme, PAU_01543, which is used in bacterial anaerobic respiration also implies cytoplasmic molecular oxygen may become limiting at 37°C. Nevertheless, *Pa* ^ATCC43949^ grows poorly under microareobic conditions and not at all in a fully anaerobic environment (on LB rich media plates) suggesting that anaerobic respiration alone is insufficient for growth. The terminal electron acceptor for *Pa* ^ATCC43949^ during microareobic growth is not known although candidates are fumarate (from the TCA cycle) or sulphate. The up regulation of various *cys*-genes at 37°C could implicate sulphate. Extracellular sulfate may be imported by the CysPUWA proteins (and Sbp) and then converted to adenosine 5’-phosphosulfate (APS), using the up-regulated CysND genes although this would consume ATP. Finally studies with *E*. *coli* have suggested that low oxygen inhibits the expression of many TCA cycle enzymes, which affects pyruvate-acetate-acetyl-CoA flow, consistent with our observations [76].

### Quorum sensing?

It has recently been discovered that *P*. *asymbiotica* can use dialkylresorcinol molecules for QS [77]. These molecules are synthesised by the products of the *darABC* operon (PAU_02400–02402) [78], which are also involved in synthesis of the IPS molecule (S7 Fig). In QS, the dialkylresorcinol molecules are sensed the PauR receptor (PAU_4062), which is a LuxR like protein, that once activated induces transcription of the *pcfABCDEF* operon (PAU_04063–68). Transcription of this operon induces a cell-clumping phenotype which has been shown to contribute to high pathogenicity of *P*. *asymbiotica* toward insect larvae [77]. Interestingly in *Pl* ^TT01^ a close homologue of PauR, named PluR, senses α-pyrones as QS signals which again induce the same cell clumping response from the *Pl* ^TT01^ *pcfABCDEF* operon [79]. It has been suggested that the use of dialkylresorcinol molecules instead of α-pyrones, which are not produced by *P*. *asymbiotica* strains, as quorum sensing molecules might be an important evolutionary step from invertebrate to human pathogenicity [77]. Nevertheless neither the *Pa* ^ATCC43949^ *pcfABCDEF* nor *pluR* genes show any significant difference in expression between the two temperatures. However we do see changes in the expression of other genes that might be suggestive of alternations in QS activity. For example, the AI-2 pathway genes *lsrFG* are induced at 37°C which would act to degrade the AI-2 signal if present. Interestingly, in *Pl* ^TT01^, knocking out the AI-2 synthesis gene *luxS* increases the oxidative stress response [80], which may indicate a role for this QS system in *Pa* ^ATCC43949^ as it attempts to adapt to a stringent mammalian host environment. More speculatively, an operon encoding genes homologous to those for the synthesis of a known *Streptomyces* QS is also strongly repressed (PAU_03767–03778) again suggesting that QS systems relevant to insect infection may be inappropriate to the human host environment. There is an interesting parallel here to the necessary loss of the *plcR* QS system of *Bacillus anthracis* in its adaptation to the mammalian host from the invertebrate associated *B*. *cereus* ancestor [81].

### Temperature sensing and regulators

While we have described in detail the changes in gene expression at 37°C, we cannot yet attribute this to any specific regulators. It is interesting that many of the down-regulated regulators are *luxR* gene homologues. However, without further experimentation we cannot determine if changes in the expression of these plays any role in human virulence. Nevertheless this remains a formal possibility [82]. For example any one could act as a repressor, the down-regulation of which could lead to the activation of mammalian specific genes at 37°C. Alternatively down regulation or dysfunction of certain response regulators could “blind” the cell to the presence of otherwise abundant nutrients. This would have the effect of forcing them into becoming nutritional specialists and therefore facilitating a nutritional virulence strategy.

Many diverse mechanisms for bacterial temperature sensing have been described. These range from RNA-thermometers [83] to temperature dependent conformation changes in specific regulator proteins. A good example of the latter is the intrinsic thermal sensing by the *Yersinia* virulence regulator RovA [84], which becomes proteolytically cleaved upon thermally induced conformation change. We actually see a slight *increase* in the expression of the *Pa* ^ATCC43949^ *slyA* gene (a *rovA* homologue) at 37°C although this is not significant by our criteria. A recent study in *Y*. *pseudotuberculosis* revealed that, like *Pa* ^ATCC43949^, they also reprogram a large set of catabolic/energy production genes in response to temperature increase. In the *Yersinia* the effects are mediated through a massive remodelling of the cAMP Regulatory Protein CRP-controlled network [85]. How this is accomplished has not yet been fully determined, however a previous study revealed many of virulence-associated functions are co-regulated with carbohydrate metabolism. This is mediated through the carbon storage regulator system Csr, involving the *csrB* and *csrC* regulatory sRNAs and CRP [86]. In the *Yersinia* the Csr system consists of an RNA binding protein CsrA that binds the Shine-Dalgarno regions of target mRNAs to repress and destabilises them. CsrA itself can be bound and sequestered by either *csrB* or *csrC*. Normally CsrA inhibits the production of the RovA virulence regulator, so increasing the expression of *csrB* leads to the de-repression of RovA and activation of several virulence factors. It is a CRP-mediated repression of the response regulator UvrY that leads to the activation of *csrB* transcription. Interestingly UvrY has been shown to be essential for the adaptation of *Photorhabdus* to the insect host [87]. Knock out the *uvrY* gene in *Pl* ^TT01^, resulted in changes in gene expression and phenotype that showed much in common with *Pa* ^ATCC43949^, grown at 37°C. For example the UvrY deficient *Pl* ^TT01^ strain showed increased transcription of *groESL* and *luxCD*, together with an increase in motility and iron transport and a decrease in carbohydrate transport. Conversely, the *uvrY* mutant also showed expression changes not consistent with *Pa* ^ATCC43949^ growth at 37°C. For example *Pl* ^TT01^ *uvrY* induces transcription of components of the AI-2 QS system, which would be theoretically repressed in *Pa* ^ATCC43949^ due to the increase in the AI-2 degrading enzymes, LsrFG. *Pl* ^TT01^ *uvrY* also increases *csrB* and represses *tldD* protease expression, which together would lead to the repression of CsrA activity. In our experiments we see no change in either *csrB* or *tldD* transcription. Nevertheless *Pl* ^TT01^ *uvrY* also becomes avirulent to insects consistent with *Pa* ^ATCC43949^ at 37°C.

Our challenge now is to determine the key regulators that allow *Pa* ^ATCC43949^ to exhibit such large differences in temperature dependent phenotype despite relatively limited changes in gene expression.

## Supporting Information

S1 Data DESeq analysis of RNA-seq data (as an excel spread-sheet). (XLSX)

S2 Data Artemis plots of Chromosomal RNA-seq data (as a zip archive). (ZIP)

S3 Data Artemis plots of pPAU1 RNA-seq data (as a zip archive). (ZIP)

S4 Data A list of *isnAB* rhabduscin synthesis gene homologues. (XLSX)

S5 Data Triplicate 2D-proteomic analysis gels, DIGE-comparison gel and DeCyder analysis of each relevant protein spot. (PDF)

S1 Fig Temperature tolerance of different strains of *Photorhabdus*. The growth dynamics of strains resuming aerated growth at 28°C in LB medium after a 4 h static exposure to a range of temperatures. Strains tested include clinical *P*. *asymbiotica* isolates from (A) USA, (B) Australia and Nepal, (C) non-clinical European *P*. *asymbiotica* isolates and (D) representative *P*. *luminescens* strains. Note there is a large variation in times taken for strains to exit lag-phase into exponential growth, but growth rates are comparable once exponential growth begins. The longer lag-phase observed at the threshold between permissive and non-permissive temperatures is therefore likely due to a proportion of the population dying due to temperature stress.
(PDF)

S2 Fig A comparison of the most abundant transcripts at 28°C and 37°C as a percentage of total reads. The graph shows the mapped base mean values as a percentage of total reads for various gene transcripts at the two temperatures. It should be noted that the majority of sRNAs are not annotated on the *Pa* ^ATCC43949^ genome so would have been excluded from the DESEQ analysis of the RNA-seq data.
(PDF)

S3 Fig qRT-PCR validation of selected transcripts. RNA-seq log_2_fold changes (y-axis) were compared to those measured by qRT-PCR. The symbols: [*], [**], [***] refers to P-value of <0.05, <0.01, <0.001 calculated using REST 2009 and DESeq for RT-PCR and RNA-seq respectively. RNA was extracted from cultures growing at exponential phase for 4 h in LB broth. Target genes were chosen to represent a range of classes and transcription levels. Primer sequences may be seen in S13 Table.
(PDF)

S4 Fig Temperature dependent bioluminescence activity. (A) Bioluminescence of *Pa* ^ATCC43949^ (purple), *Pa* ^Kingscliff^ (blue), *Pl* ^TT01^ (green) and *E*. *coli* DH5α negative control (red) growing at 28°C (top) and 37°C (bottom) in LB broth with aeration. Graphs show the relative mean light emission per cell (n = 3). All strains showed normal growth curves at 28°C and 37°C except *Pl* ^TT01^ which only reached OD_600_ of 0.2 by 10 h at 37°C before declining. Red dotted line shows the equivalent emission level between the two graphs for comparison.
(PDF)

S5 Fig Classical microbiological testing of *Photorhabdus* phenotypes. Panels (A-C) show *P*. *asymbiotica* ^ATCC43949^, (D-F) show *P*. *asymbiotica* ^Kingscliff^ and (G-H) show *P*. *luminescens* ^TT01^. The strains were cultured on solid agar media under aerobic or microaerobic conditions at 28°C and 37°C. Various supplements were added to the plates as indicated. In the Oxidation/Fermentation (O/F) plates the bromothymol blue indicator change from blue to yellow indicates acid production from the supplemented carbon source.
(PDF)

S6 Fig Transcription of the non-ribosomal peptide synthase gene *gxpS* for the GameXPeptide natural product at 28°C and 37°C. Increased transcription of this NRPS gene correlates with detection of increased levels of the final peptide in the supernatant.
(PDF)

S7 Fig Production of the antibiotic and anti-inflammatory molecule iso-propyl-stilbene at 28°C and 37°C. (A) Artemis views of the RNA-seq reads of the three replicates mapped onto the *Pa* ^ATCC43949^ operons responsible for IPS synthesis. Note *stlA* shows very little transcription during exponential growth although it is induced more highly at stationary phase (data not shown). Conversely the *darABC* operon transcription is inducted at 37°C during exponential growth. (B) *Staphylococcus aureus* MRSA (strain cdc16) overlaid onto *Pa* ^ATCC43949^ grown on LB agar plates at 28°C and 37°C showing increased antibiotic production at 37°C. Arrows indicate zones of growth inhibition of the MRSA which is almost complete on the 37°C plate.
(PDF)

S8 Fig Resistance to human serum killing by *Photorhabdus*. Growth of (A) *Pa* ^ATCC43949^ and (B) *Pl* ^TT01^ and (C) *E*. *coli*, *Ec* ^DH5α^, at 28°C with aeration in LB and LB supplemented with 20% (*v/v*) human serum (type AB), Heat Inactivated human AB serum (HIS) and 0.9% saline. Results shown are the average of three independent experiments +/- standard error bars.
(PDF)

S9 Fig *Photorhabdus* can also resist killing by serum from other mammals. Growth of (A) *Pa* ^ATCC43949^ and (B) *Pl* ^TT01^ at 28°C with aeration in LB supplemented with 10% (*v/v*) serum from pigs and rabbits. Heat inactivated (HI) serum was also included. Note *Pa* ^ATCC43949^ is resistant to both types while *Pl* ^TT01^ shows complete resistance to rabbit but is strongly inhibited by the pig serum.
(PDF)

S10 Fig Bacterial morphology during immune cell interaction and biofilm formation at different host temperatures. (A-B) Bacterial morphology and behaviour of GFP labelled *Pa* ^ATCC43949^ when exposed to (A) *Manduca sexta* haemocytes (bled from a whole animal infection) at 28°C in normal air and (B) cultured J774.2 murine macrophage-like cells at 37°C in a 5% CO_2_ incubator. The host cell actin cytoskeleton is labelled with Phalloidin:TRITC stain in both cases and slides visualised on a confocal microscope. White bar represents 10μM. (C-E) Time courses showing representative *Pa* ^ATCC43949^ cell morphology and early biofilm development in LB medium in static conditions on glass slides under different conditions, (C) at 28°C in normal air, (D) at 37°C in 5% C0_2_ and (E) at 37°C in normal air. White bar represents 500μM. Note a combination of 37°C and 5% C0_2_ induces filamentation as also seen in panel (B).
(PDF)

S1 Table Transcripts more abundant at 37°C than at 28°C. (DOCX)

S2 Table Transcripts more abundant at 28°C than at 37°C. (DOCX)

S3 Table Proteomic differences of *P*. *asymbiotica* ^ATCC43949^ cellular proteins at 28°C and 37°C. (DOCX)

S4 Table Carbon source utilization enabling respiration for *P*. *asymbiotica* ^ATCC43949^ (Pa) and *P*. *luminescens* ^TT01^ (Pl) at 28°C and 37°C (part 1). (DOCX)

S5 Table Carbon source utilization enabling respiration for *P*. *asymbiotica* ^ATCC43949^ (Pa) and *P*. *luminescens* ^TT01^ (Pl) at 28°C and 37°C (part 2). (DOCX)

S6 Table Nitrogen source utilization enabling respiration for *P*. *asymbiotica* ^ATCC43949^ (Pa) and *P*. *luminescens* ^TT01^ (Pl) at 28°C and 37°C. (DOCX)

S7 Table Utilization of peptides as nitrogen sources for respiration for *P*. *asymbiotica* ^ATCC43949^ (Pa) and *P*. *luminescens* ^TT01^ (Pl) at 28°C and 37°C (part 1). (DOCX)

S8 Table Utilization of peptides as nitrogen sources for respiration for *P*. *asymbiotica* ^ATCC43949^ (Pa) and *P*. *luminescens* ^TT01^ (Pl) at 28°C and 37°C (part 2). (DOCX)

S9 Table Utilization of peptides as nitrogen sources for respiration for *P*. *asymbiotica* ^ATCC43949^ (Pa) and *P*. *luminescens* ^TT01^ (Pl) at 28°C and 37°C (part 3). (DOCX)

S10 Table Osmolyte effect on respiration for *P*. *asymbiotica* ^ATCC43949^ (Pa) and *P*. *luminescens* ^TT01^ (Pl) at 28°C and 37°C. (DOCX)

S11 Table pH effect on respiration for *P*. *asymbiotica* ^ATCC43949^ (Pa) and *P*. *luminescens* ^TT01^ (Pl) at 28°C and 37°C. (DOCX)

S12 Table Changes in the production of known secondary metabolites found in *P*. *asymbiotica* at 28°C and 37°C. (DOCX)

S13 Table qRT-PCR primers used for data presented in S5 Fig. (DOCX)

## References

[1] Waterfield NR, Ciche T, Clarke D. *Photorhabdus* and a host of hosts. Annual review of microbiology. 2009;63:557–74. Epub 2009/07/07. 10.1146/annurev.micro.091208.073507 19575559

[2] Ferreira T, van Reenen CA, Endo A, Tailliez P, Pages S, Sproer C, et al *Photorhabdus heterorhabditis* sp. nov., a symbiont of the entomopathogenic nematode *Heterorhabditis zealandica* . International journal of systematic and evolutionary microbiology. 2014;64(Pt 5):1540–5. 10.1099/ijs.0.059840-0 24478206

[3] Tailliez P, Laroui C, Ginibre N, Paule A, Pages S, Boemare N. Phylogeny of *Photorhabdus* and *Xenorhabdus* based on universally conserved protein-coding sequences and implications for the taxonomy of these two genera. Proposal of new taxa: *X*. *vietnamensis* sp. nov., *P*. *luminescens* subsp. caribbeanensis subsp. nov., *P*. *luminescens* subsp. *hainanensis* subsp. nov., *P*. *temperata* subsp. *khanii* subsp. nov., *P*. *temperata* subsp. *tasmaniensis* subsp. nov., and the reclassification of *P*. *luminescens* subsp. *thracensis* as *P*. *temperata* subsp. *thracensis* comb. nov. International journal of systematic and evolutionary microbiology. 2010;60(Pt 8):1921–37. 10.1099/ijs.0.014308-0 19783607

[4] Orozco RA, Hill T, Stock SP. Characterization and phylogenetic relationships of Photorhabdus luminescens subsp. sonorensis (gamma-Proteobacteria: Enterobacteriaceae), the bacterial symbiont of the entomopathogenic nematode Heterorhabditis sonorensis (Nematoda: Heterorhabditidae). Current microbiology. 2013;66(1):30–9. 10.1007/s00284-012-0220-6 23053483

[5] Gerrard JG, Joyce SA, Clarke DJ, ffrench-Constant RH, Nimmo GR, Looke DFM, et al Nematode symbiont for *Photorhabdus asymbiotica* . Emerging Infectious Diseases. 2006;12(10):1562–4. 17176572 10.3201/eid1210.060464 PMC3290952

[6] Gerrard JG, McNevin S, Alfredson D, Forgan-Smith R, Fraser N. *Photorhabdus* species: bioluminescent bacteria as emerging human pathogens? Emerg Infect Dis. 2003;9(2):251–4. 12603999 10.3201/eid0902.020220 PMC2902266

[7] Gerrard J, Waterfield N, Vohra R, ffrench-Constant R. Human infection with *Photorhabdus asymbiotica*: an emerging bacterial pathogen. Microbes and infection / Institut Pasteur. 2004;6(2):229–37. 15049334 10.1016/j.micinf.2003.10.018

[8] Weissfeld AS, Halliday RJ, Simmons DE, Trevino EA, Vance PH, O'Hara CM, et al *Photorhabdus asymbiotica*, a pathogen emerging on two continents that proves that there is no substitute for a well-trained clinical microbiologist. Journal of clinical microbiology. 2005;43(8):4152–5. 16081963 10.1128/JCM.43.8.4152-4155.2005 PMC1234010

[9] Theopold U, Krautz R, Dushay MS. The *Drosophila* clotting system and its messages for mammals. Developmental and comparative immunology. 2014;42(1):42–6. 10.1016/j.dci.2013.03.014 23545286

[10] Kimbrell DA, Beutler B. The evolution and genetics of innate immunity. Nat Rev Genet. 2001;2(4):256–67. 11283698 10.1038/35066006

[11] Hultmark D. Immune reactions in *Drosophila* and other insects: a model for innate immunity. Trends in Genetics. 1993;9:178–84. 8337755 10.1016/0168-9525(93)90165-e

[12] Medzhitov R, Janeway CA Jr. Decoding the patterns of self and nonself by the innate immune system. Science. 2002;296(5566):298–300. 11951031 10.1126/science.1068883

[13] Waterfield NR, Sanchez-Contreras M, Eleftherianos I, Dowling A, Wilkinson P, Parkhilll J, et al Rapid Virulence Annotation (RVA): Identification of virulence factors using a bacterial genome library and multiple invertebrate hosts. Proceedings of the National Academy of Sciences of the United States of America. 2008;105(41):15967–72. 10.1073/pnas.0711114105 18838673 PMC2572985

[14] Read TD, Peterson SN, Tourasse N, Baillie LW, Paulsen IT, Nelson KE, et al The genome sequence of *Bacillus anthracis* Ames and comparison to closely related bacteria. Nature. 2003;423(6935):81–6. 12721629 10.1038/nature01586

[15] Meibom KL, Blokesch M, Dolganov NA, Wu CY, Schoolnik GK. Chitin induces natural competence in *Vibrio cholerae* . Science. 2005;310(5755):1824–7. Epub 2005/12/17. 310/5755/1824 10.1126/science.1120096 16357262

[16] Wilkinson P, Waterfield NR, Crossman L, Corton C, Sanchez-Contreras M, Vlisidou I, et al Comparative genomics of the emerging human pathogen *Photorhabdus asymbiotica* with the insect pathogen *Photorhabdus luminescens* . BMC Genomics. 2009;10:302 Epub 2009/07/09. 1471-2164-10-302. 10.1186/1471-2164-10-302 19583835 PMC2717986

[17] Wilkinson P, Paszkiewicz K, Moorhouse A, Szubert JM, Beatson S, Gerrard J, et al New plasmids and putative virulence factors from the draft genome of an Australian clinical isolate of *Photorhabdus asymbiotica* . FEMS microbiology letters. 2010;309(2):136–43. Epub 2010/06/30. FML2030 10.1111/j.1574-6968.2010.02030.x 20584081

[18] Fischer-Le Saux M, Viallard V, Brunel B, Normand P, Boemare NE. Polyphasic classification of the genus *Photorhabdus* and proposal of new taxa: *P*. *luminescens* subsp. *luminescens* subsp. nov., *P*. *luminescens* subsp. *akhurstii* subsp. nov., *P*. *luminescens* subsp. *laumondii* subsp. nov., *P*. *temperata* sp. nov., *P*. *temperata* subsp. *temperata* subsp. nov. and *P*. *asymbiotica* sp. nov. Int J Syst Bacteriol. 1999;49:1645–56. 10555346 10.1099/00207713-49-4-1645

[19] Konkel ME, Tilly K. Temperature-regulated expression of bacterial virulence genes. Microbes and infection / Institut Pasteur. 2000;2(2):157–66. 10742688 10.1016/s1286-4579(00)00272-0

[20] Marceau M. Transcriptional regulation in *Yersinia*: an update. Current issues in molecular biology. 2005;7(2):151–77. 16053248

[21] Manual M. Available: http://maq.sourceforge.net/maq-man.shtml.

[22] Mortazavi A, Williams BA, McCue K, Schaeffer L, Wold B. Mapping and quantifying mammalian transcriptomes by RNA-Seq. Nature methods. 2008;5(7):621–8. 10.1038/nmeth.1226 18516045 PMC13303166

[23] Anders S, Huber W. Differential expression analysis for sequence count data. Genome biology. 2010;11(10):R106 10.1186/gb-2010-11-10-r106 . 20979621 PMC3218662

[24] Croucher NJ, Fookes MC, Perkins TT, Turner DJ, Marguerat SB, Keane T, et al A simple method for directional transcriptome sequencing using Illumina technology. Nucleic acids research. 2009;37(22):e148 10.1093/nar/gkp811 . 19815668 PMC2794173

[25] Ning Z, Cox AJ, Mullikin JC. SSAHA: a fast search method for large DNA databases. Genome research. 2001;11(10):1725–9. 10.1101/gr.194201 11591649 PMC311141

[26] cigar2Coverage PERL script URL. Available: ftp://ftp.sanger.ac.uk/pub/project/pathogens/st/solexa/Typhi_transcriptome/cigar2CoverageStranded.pl

[27] Feret R L K. Protein profiling using two-dimensional difference gel electrophoresis (2-D DIGE). Curr Protoc Protein Sci. 2014;75(Feb 3). 10.1002/0471140864.ps2202s75 24510675

[28] Daborn PJ, Waterfield N, Silva CP, Au CPY, Sharma S, Ffrench-Constant RH. A single *Photorhabdus* gene, makes caterpillars floppy (*mcf*), allows *Escherichia coli* to persist within and kill insects. Proceedings of the National Academy of Sciences of the United States of America. 2002;99(16):10742–7. 10.1073/pnas.102068099 12136122 PMC125031

[29] Seelen MA, Roos A, Wieslander J, Mollnes TE, Sjoholm AG, Wurzner R, et al Functional analysis of the classical, alternative, and MBL pathways of the complement system: standardization and validation of a simple ELISA. Journal of immunological methods. 2005;296(1–2):187–98. 10.1016/j.jim.2004.11.016 15680163

[30] Crawford JM, Portmann C, Zhang X, Roeffaers MB, Clardy J. Small molecule perimeter defense in entomopathogenic bacteria. Proc Natl Acad Sci U S A. 2012;109(27):10821–6. Epub 2012/06/20. 1201160109 [pii] 10.1073/pnas.1201160109 22711807 PMC3390839

[31] Reynolds SE, Nottingham SF. Food and water economy and its relation to growth in the 5th-instar larvae of the Tobacco Hornworm, *Manduca sexta* . Journal of insect physiology. 1985;31:119–27.

[32] Yang G, Dowling AJ, Gerike U, ffrench-Constant RH, Waterfield NR. *Photorhabdus* virulence cassettes confer injectable insecticidal activity against the wax moth. Journal of bacteriology. 2006;188(6):2254–61. 16513755 10.1128/JB.188.6.2254-2261.2006 PMC1428146

[33] Waterfield N, Kamita SG, Hammock BD, ffrench-Constant R. The *Photorhabdus* Pir toxins are similar to a developmentally regulated insect protein but show no juvenile hormone esterase activity. FEMS microbiology letters. 2005;245(1):47–52. 15796978 10.1016/j.femsle.2005.02.018

[34] Jones RT, Sanchez-Contreras M, Vlisidou I, Amos MR, Yang G, Munoz-Berbel X, et al *Photorhabdus* adhesion modification protein (Pam) binds extracellular polysaccharide and alters bacterial attachment. BMC microbiology. 2010;10. 10.1186/1471-2180-10-141 PMC2878306 20462430

[35] Urbanowski ML, Stauffer LT, Stauffer GV. The *gcvB* gene encodes a small untranslated RNA involved in expression of the dipeptide and oligopeptide transport systems in *Escherichia coli* . Molecular microbiology. 2000;37(4):856–68. 10972807 10.1046/j.1365-2958.2000.02051.x

[36] Reichenbach B, Maes A, Kalamorz F, Hajnsdorf E, Gorke B. The small RNA GlmY acts upstream of the sRNA GlmZ in the activation of glmS expression and is subject to regulation by polyadenylation in *Escherichia coli* . Nucleic acids research. 2008;36(8):2570–80. 10.1093/nar/gkn091 18334534 PMC2377431

[37] Lilley KS, Friedman DB. All about DIGE: quantification technology for differential-display 2D-gel proteomics. Expert review of proteomics. 2004;1(4):401–9. 10.1586/14789450.1.4.401 15966837

[38] Bode HB. Entomopathogenic bacteria as a source of secondary metabolites. Curr Opin Chem Biol. 2009;13(2):224–30. Epub 2009/04/07. S1367-5931(09)00024-6.0. 19345136 10.1016/j.cbpa.2009.02.037

[39] Nollmann FI, Dauth C, Mulley G, Kegler C, Kaiser M, Waterfield NR, et al Insect-Specific Production of New GameXPeptides in *Photorhabdus luminescens* TTO1, Widespread Natural Products in Entomopathogenic Bacteria. Chembiochem: a European journal of chemical biology. 2015;16(2):205–8. 10.1002/cbic.201402603 25425189

[40] Richardson WH, Schmidt TM, Nealson KH. Identification of an anthraquinone pigment and a hydroxystilbene antibiotic from *Xenorhabdus luminescens* . Applied and environmental microbiology. 1988;54(6):1602–5. 3415225 10.1128/aem.54.6.1602-1605.1988 PMC202703

[41] Kronenwerth M, Brachmann AO, Kaiser M, Bode HB. Bioactive derivatives of isopropylstilbene from mutasynthesis and chemical synthesis. Chembiochem: a European journal of chemical biology. 2014;15(18):2689–91. 10.1002/cbic.201402447 25346446

[42] Plichta Kathryn. L. J SA, Clarke David, Waterfield Nick, and Stock S. Patricia. Heterorhabditis gerrardi n. sp. (Nematoda: Heterorhabditidae): the hidden host of Photorhabdus asymbiotica (Enterobacteriaceae: gamma-Proteobacteria). Journal of Helminthology. 2009 12;83(4):309–20. 10.1017/S0022149X09222942 19216823

[43] Hu K, Webster JM. Antibiotic production in relation to bacterial growth and nematode development in *Photorhabdus*-*Heterorhabditis* infected *Galleria mellonella* larvae. FEMS microbiology letters. 2000;189(2):219–23. 10930742 10.1111/j.1574-6968.2000.tb09234.x

[44] Li J, Chen G, Wu H, Webster JM. Identification of two pigments and a hydroxystilbene antibiotic from *Photorhabdus luminescens* . Applied and environmental microbiology. 1995;61(12):4329–33. 8534100 10.1128/aem.61.12.4329-4333.1995 PMC167744

[45] Joyce SA, Lango L, Clarke DJ. The Regulation of Secondary Metabolism and Mutualism in the Insect Pathogenic Bacterium *Photorhabdus luminescens* . Advances in applied microbiology. 2011;76:1–25. 10.1016/B978-0-12-387048-3.00001-5 21924970

[46] Buscato E, Buttner D, Bruggerhoff A, Klingler FM, Weber J, Scholz B, et al From a multipotent stilbene to soluble epoxide hydrolase inhibitors with antiproliferative properties. ChemMedChem. 2013;8(6):919–23. 10.1002/cmdc.201300057 23596124

[47] Zhao L, Zhu B, Chen X, Chen G, Chen H, Li Y, et al Development and validation of a rapid and sensitive liquid chromatography-tandem mass spectrometry method for benvitimod quantification in human plasma. Journal of chromatography B, Analytical technologies in the biomedical and life sciences. 2012;885–886:160–5. 10.1016/j.jchromb.2011.12.026 22281235

[48] Meylaers K, Cerstiaens A, Vierstraete E, Baggerman G, Michiels CW, De Loof A, et al Antimicrobial compounds of low molecular mass are constitutively present in insects: characterisation of beta-alanyl-tyrosine. Current pharmaceutical design. 2003;9(2):159–74. 12570666 10.2174/1381612033392279

[49] Vilcinskas A. Evolutionary plasticity of insect immunity. Journal of insect physiology. 2013;59(2):123–9. 10.1016/j.jinsphys.2012.08.018 22985862

[50] Gunn JS, Ryan SS, Van Velkinburgh JC, Ernst RK, Miller SI. Genetic and functional analysis of a PmrA-PmrB-regulated locus necessary for lipopolysaccharide modification, antimicrobial peptide resistance, and oral virulence of *Salmonella enterica* serovar *typhimurium* . Infection and immunity. 2000;68(11):6139–46. 11035717 10.1128/iai.68.11.6139-6146.2000 PMC97691

[51] Marceau M, Sebbane F, Ewann F, Collyn F, Lindner B, Campos MA, et al The pmrF polymyxin-resistance operon of *Yersinia pseudotuberculosis* is upregulated by the PhoP-PhoQ two-component system but not by PmrA-PmrB, and is not required for virulence. Microbiology. 2004;150(Pt 12):3947–57. 10.1099/mic.0.27426-0 15583148

[52] Mouammine A, Lanois A, Pages S, Lafay B, Molle V, Canova M, et al Ail and PagC-Related Proteins in the Entomopathogenic Bacteria of *Photorhabdus* Genus. PloS one. 2014;9(10):e110060 10.1371/journal.pone.0110060 25333642 PMC4198210

[53] Smith KS, Ferry JG. Prokaryotic carbonic anhydrases. FEMS microbiology reviews. 2000;24(4):335–66. 10978542 10.1111/j.1574-6976.2000.tb00546.x

[54] Dowling AJ, Waterfield NR, Hares MC, Le Goff G, Streuli CH, Ffrench-Constant RH. The Mcf1 toxin induces apoptosis via the mitochondrial pathway and apoptosis is attenuated by mutation of the BH3-like domain. Cellular microbiology. 2007;9(10):2470–84. 10.1111/j.1462-5822.2007.00974.x 17848168

[55] Waterfield N, Kamita S. G., Hammock B. D. and ffrench-Constant R. H. The *Photorhabdus* Pir toxins are similar to a developmentally regulated insect protein but show no juvenile hormone esterase activity. FEMS microbiology letters. 2005 4 1;245(1):47–52. 15796978 10.1016/j.femsle.2005.02.018

[56] Costa SC, Girard PA, Brehelin M, Zumbihl R. The emerging human pathogen *Photorhabdus asymbiotica* is a facultative intracellular bacterium and induces apoptosis of macrophage-like cells. Infection and immunity. 2008 Epub 2008/12/17. IAI.01064-08 10.1128/IAI.01064-08 PMC2643617 19075024

[57] Abu Kwaik Y, Bumann D. Microbial quest for food in vivo: 'nutritional virulence' as an emerging paradigm. Cellular microbiology. 2013;15(6):882–90. 10.1111/cmi.12138 23490329

[58] Zhang YJ, Rubin EJ. Feast or famine: the host-pathogen battle over amino acids. Cellular microbiology. 2013;15(7):1079–87. 10.1111/cmi.12140 23521858 PMC6434321

[59] Sharma V, Ichikawa M, Freeze HH. Mannose metabolism: More than meets the eye. Biochemical and biophysical research communications. 2014 10.1016/j.bbrc.2014.06.021 PMC4252654 24931670

[60] Mulley G, Lopez-Gomez M, Zhang Y, Terpolilli J, Prell J, Finan T, et al Pyruvate is synthesized by two pathways in pea bacteroids with different efficiencies for nitrogen fixation. Journal of bacteriology. 2010;192(19):4944–53. 10.1128/JB.00294-10 20675477 PMC2944551

[61] Sandberg TE, Pedersen M, LaCroix RA, Ebrahim A, Bonde M, Herrgard MJ, et al Evolution of *Escherichia coli* to 42 degrees C and Subsequent Genetic Engineering Reveals Adaptive Mechanisms and Novel Mutations. Molecular biology and evolution. 2014 10.1093/molbev/msu209 PMC4166923 25015645

[62] Silva CP, Waterfield NR, Daborn PJ, Dean P, Chilver T, Au CPY, et al Bacterial infection of a model insect: *Photorhabdus luminescens* and *Manduca sexta* . Cellular microbiology. 2002;6(4):329–39. 10.1046/j.1462-5822.2002.00194.x 12067318

[63] Daborn PJ, Waterfield N, Blight MA, Ffrench-Constant RH. Measuring virulence factor expression by the pathogenic bacterium *Photorhabdus luminescens* in culture and during insect infection. Journal of bacteriology. 2001;183(20):5834–9. 10.1128/JB.183.20.5834-5839.2001 11566980 PMC99659

[64] Bishop AH. Expression of prtA from *Photorhabdus luminescens* in *Bacillus thuringiensis* enhances mortality in lepidopteran larvae by sub-cutaneous but not oral infection. Journal of invertebrate pathology. 2014;121C:85–8. 10.1016/j.jip.2014.07.001 25036004

[65] Felfoldi G, Marokhazi J, Kepiro M, Venekei I. Identification of natural target proteins indicates functions of a serralysin-type metalloprotease, PrtA, in anti-immune mechanisms. Applied and environmental microbiology. 2009;75(10):3120–6. 10.1128/AEM.02271-08 19304826 PMC2681659

[66] Kurashige S, Akuzawa Y, Mitsuhashi S. Purine metabolic enzymes in lymphocytes. IV. Effects of enzyme inhibitors and enzyme substrates on the blastogenic responses of human lymphocytes. Scandinavian journal of immunology. 1985;22(1):1–7. 3927475 10.1111/j.1365-3083.1985.tb01853.x

[67] Roth JR, Lawrence JG, Rubenfield M, Kieffer-Higgins S, Church GM. Characterization of the cobalamin (vitamin B12) biosynthetic genes of *Salmonella typhimurium* . Journal of bacteriology. 1993;175(11):3303–16. 8501034 10.1128/jb.175.11.3303-3316.1993 PMC204727

[68] Lee KM, Go J, Yoon MY, Park Y, Kim SC, Yong DE, et al Vitamin B12-mediated restoration of defective anaerobic growth leads to reduced biofilm formation in *Pseudomonas aeruginosa* . Infection and immunity. 2012;80(5):1639–49. 10.1128/IAI.06161-11 22371376 PMC3347431

[69] Fan J, Ye J, Kamphorst JJ, Shlomi T, Thompson CB, Rabinowitz JD. Quantitative flux analysis reveals folate-dependent NADPH production. Nature. 2014;510(7504):298–302. 10.1038/nature13236 24805240 PMC4104482

[70] Kullas AL, McClelland M, Yang HJ, Tam JW, Torres A, Porwollik S, et al L-asparaginase II produced by *Salmonella typhimurium* inhibits T cell responses and mediates virulence. Cell host & microbe. 2012;12(6):791–8. 10.1016/j.chom.2012.10.018 23245323 PMC4361029

[71] Stark RM, Suleiman MS, Hassan IJ, Greenman J, Millar MR. Amino acid utilisation and deamination of glutamine and asparagine by *Helicobacter pylori* . Journal of medical microbiology. 1997;46(9):793–800. 9291892 10.1099/00222615-46-9-793

[72] Tala A, Monaco C, Nagorska K, Exley RM, Corbett A, Zychlinsky A, et al Glutamate utilization promotes meningococcal survival in vivo through avoidance of the neutrophil oxidative burst. Molecular microbiology. 2011;81(5):1330–42. 10.1111/j.1365-2958.2011.07766.x 21777301 PMC3755445

[73] Scotti C, Sommi P, Pasquetto MV, Cappelletti D, Stivala S, Mignosi P, et al Cell-cycle inhibition by *Helicobacter pylori* L-asparaginase. PloS one. 2010;5(11):e13892 10.1371/journal.pone.0013892 21085483 PMC2976697

[74] Watson RJ, Millichap P, Joyce SA, Reynolds S, Clarke DJ. The role of iron uptake in pathogenicity and symbiosis in *Photorhabdus luminescens* TT01. BMC microbiology. 2010;10:177 10.1186/1471-2180-10-177 20569430 PMC2905363

[75] Martin B. "Angel's Glow at The Battle of Shiloh". Available: http://www.americancivilwarstory.com/angels-glow-shiloh.html.

[76] Wolfe AJ. The acetate switch. Microbiology and molecular biology reviews: MMBR. 2005;69(1):12–50. 10.1128/MMBR.69.1.12-50.2005 15755952 PMC1082793

[77] Fuchs SW, Bozhuyuk KA, Kresovic D, Grundmann F, Dill V, Brachmann AO, et al Formation of 1,3-cyclohexanediones and resorcinols catalyzed by a widely occurring ketosynthase. Angewandte Chemie. 2013;52(15):4108–12. 10.1002/anie.201210116 23423827

[78] Brameyer S K D, Bode HB, Heermann R. Dialkylresorcinols as bacterial signaling molecules. Proc Natl Acad Sci U S A. 2015;1 13;(112(2)):572–7577. 10.1073/pnas.1417685112 25550519 PMC4299209

[79] Brachmann AO, Brameyer S, Kresovic D, Hitkova I, Kopp Y, Manske C, et al Pyrones as bacterial signaling molecules. Nat Chem Biol. 2013;9(9):573–8. 10.1038/nchembio.1295 23851573

[80] Krin E, Chakroun N, Turlin E, Givaudan A, Gaboriau F, Bonne I, et al Pleiotropic role of quorum-sensing autoinducer 2 in *Photorhabdus luminescens* . Applied and environmental microbiology. 2006;72(10):6439–51. 10.1128/AEM.00398-06 17021191 PMC1610301

[81] Mignot T, Mock M, Robichon D, Landier A, Lereclus D, Fouet A. The incompatibility between the PlcR- and AtxA-controlled regulons may have selected a nonsense mutation in *Bacillus anthracis* . Molecular microbiology. 2001;42(5):1189–98. 11886551 10.1046/j.1365-2958.2001.02692.x

[82] Brameyer S, Kresovic D, Bode HB, Heermann R. LuxR solos in *Photorhabdus* species. Frontiers in cellular and infection microbiology. 2014;4:166 10.3389/fcimb.2014.00166 25478328 PMC4235431

[83] Grosso-Becera MV, Servin-Gonzalez L, Soberon-Chavez G. RNA structures are involved in the thermoregulation of bacterial virulence-associated traits. Trends in microbiology. 2015 10.1016/j.tim.2015.04.004 25999019

[84] Herbst K, Bujara M, Heroven AK, Opitz W, Weichert M, Zimmermann A, et al Intrinsic thermal sensing controls proteolysis of *Yersinia* virulence regulator RovA. PLoS pathogens. 2009;5(5):e1000435 10.1371/journal.ppat.1000435 19468295 PMC2676509

[85] Nuss AM, Heroven AK, Waldmann B, Reinkensmeier J, Jarek M, Beckstette M, et al Transcriptomic profiling of *Yersinia pseudotuberculosis* reveals reprogramming of the Crp regulon by temperature and uncovers Crp as a master regulator of small RNAs. PLoS Genet. 2015 3 27;11(3):e1005087 10.1371/journal.pgen.1005087 25816203 PMC4376681

[86] Heroven AK, Sest M, Pisano F, Scheb-Wetzel M, Steinmann R, Bohme K, et al Crp induces switching of the CsrB and CsrC RNAs in *Yersinia pseudotuberculosis* and links nutritional status to virulence. Frontiers in cellular and infection microbiology. 2012;2:158 10.3389/fcimb.2012.00158 23251905 PMC3523269

[87] Krin E, Derzelle S, Bedard K, Adib-Conquy M, Turlin E, Lenormand P, et al Regulatory role of UvrY in adaptation of *Photorhabdus luminescens* growth inside the insect. Environmental microbiology. 2008;10(5):1118–34. 10.1111/j.1462-2920.2007.01528.x 18248456

