# Supplementary material for: From Insect to Man: Photorhabdus Sheds Light on the Emergence of Human Pathogenicity
Source: PLoS One. 2015 Dec 17;10(12):e0144937. doi: 10.1371/journal.pone.0144937 (PMC4683029; doi:10.1371/journal.pone.0144937)

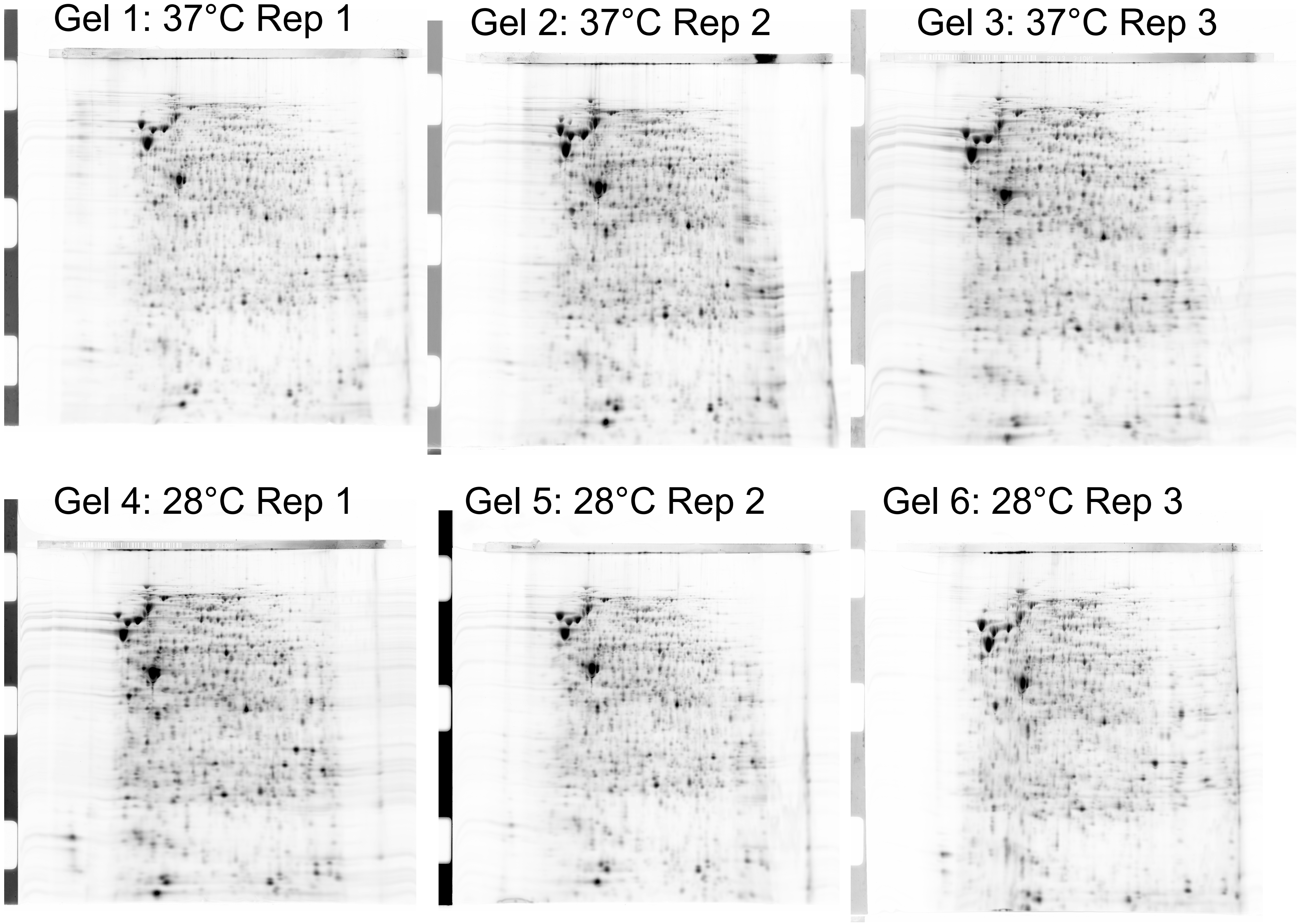
Gel 1: 37°C Rep 1
Gel 2: 37°C Rep 2
Gel 3: 37°C Rep 3
Gel 4: 28°C Rep 1
Gel 5: 28°C Rep 2
Gel 6: 28°C Rep 3

Gel 1 master image: green – cy3 labelled sample 1, red – cy5 labelled standard consisting of all the 6 samples used in experiment, yellow - where two colours overlay

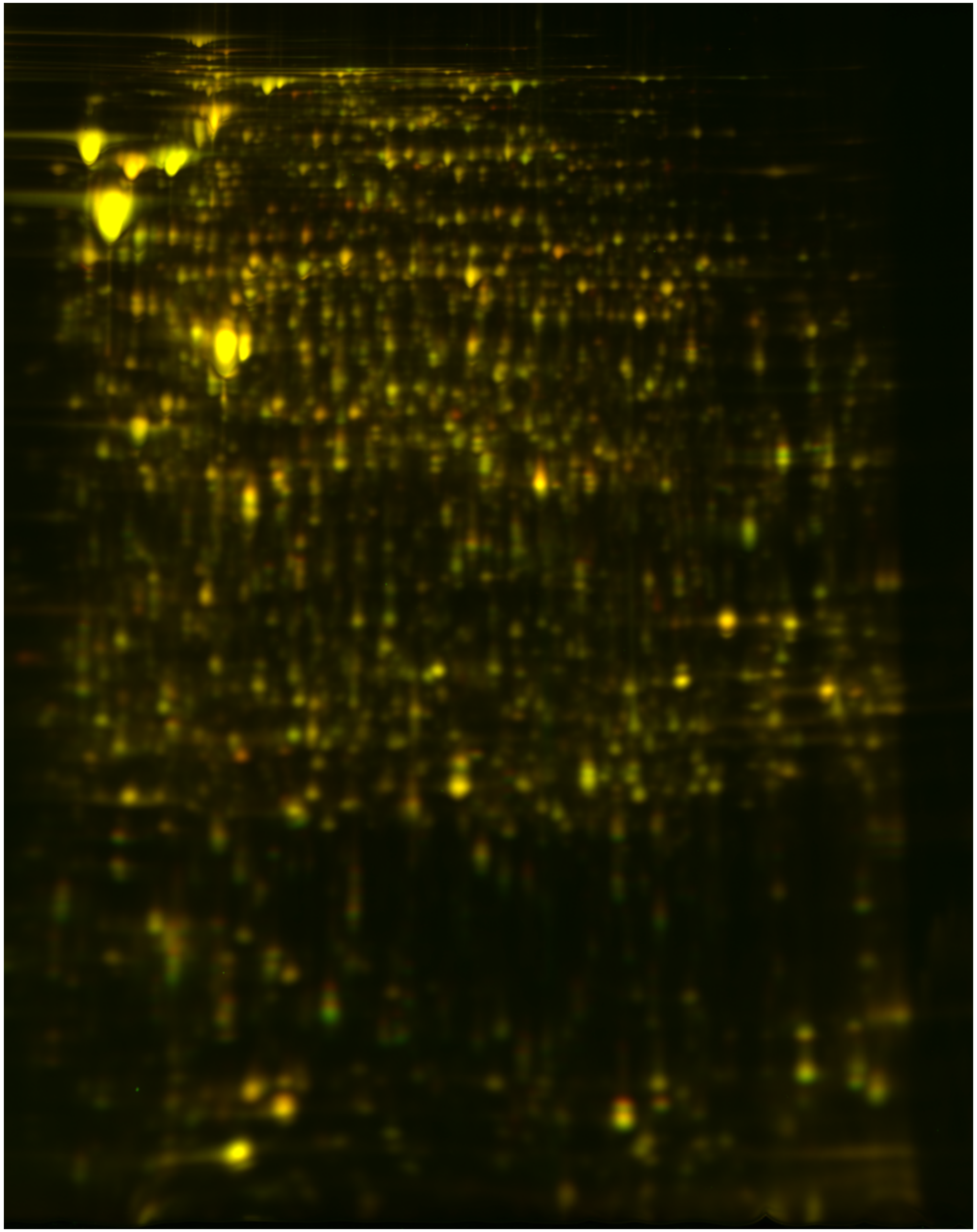

# ClpB (PAU_03190)

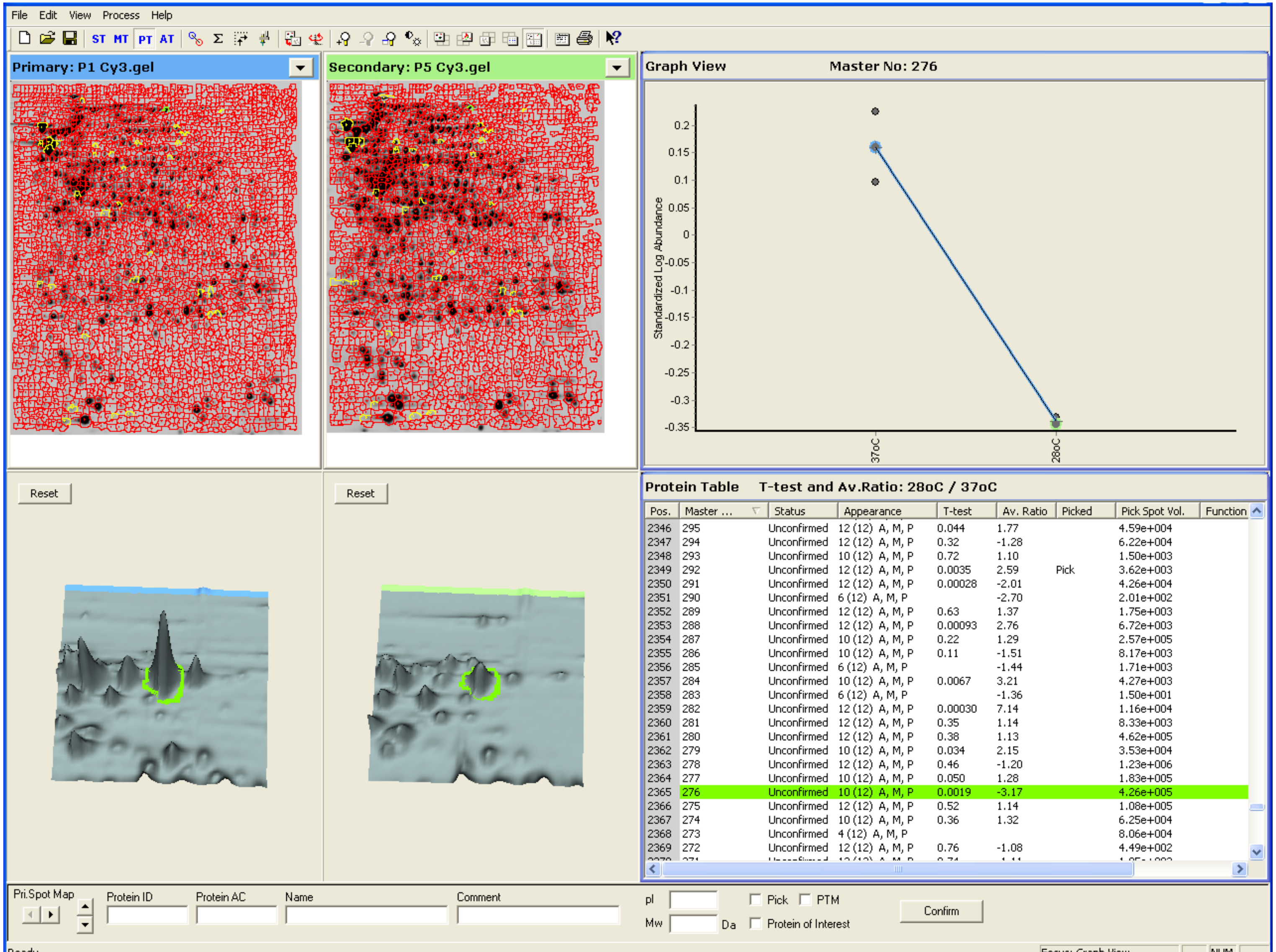

# ClpB (PAU_03190)

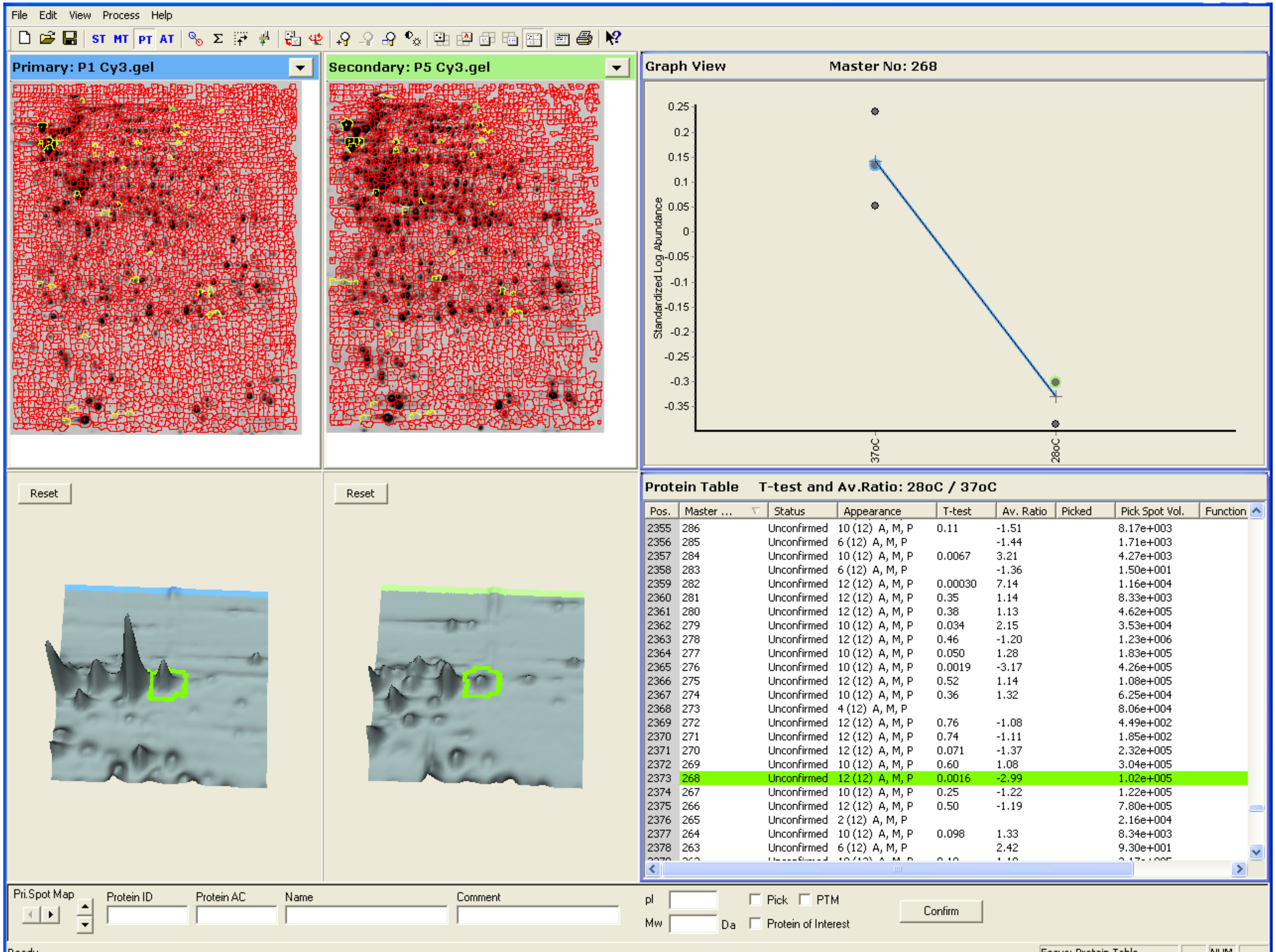


| Pos. | Master ... | Status | Appearance | T-test | Av. Ratio | Picked | Pick Spot Vol. | Function |
|---|---|---|---|---|---|---|---|---|
| 2355 | 286 | Unconfirmed | 10 (12) A, M, P | 0.11 | -1.51 | | 8.17e+003 | |
| 2356 | 285 | Unconfirmed | 6 (12) A, M, P | | -1.44 | | 1.71e+003 | |
| 2357 | 284 | Unconfirmed | 10 (12) A, M, P | 0.0067 | 3.21 | | 4.27e+003 | |
| 2358 | 283 | Unconfirmed | 6 (12) A, M, P | | -1.36 | | 1.50e+001 | |
| 2359 | 282 | Unconfirmed | 12 (12) A, M, P | 0.00030 | 7.14 | | 1.16e+004 | |
| 2360 | 281 | Unconfirmed | 12 (12) A, M, P | 0.35 | 1.14 | | 8.33e+003 | |
| 2361 | 280 | Unconfirmed | 12 (12) A, M, P | 0.38 | 1.13 | | 4.62e+005 | |
| 2362 | 279 | Unconfirmed | 10 (12) A, M, P | 0.034 | 2.15 | | 3.53e+004 | |
| 2363 | 278 | Unconfirmed | 12 (12) A, M, P | 0.46 | -1.20 | | 1.23e+006 | |
| 2364 | 277 | Unconfirmed | 10 (12) A, M, P | 0.050 | 1.28 | | 1.83e+005 | |
| 2365 | 276 | Unconfirmed | 10 (12) A, M, P | 0.0019 | -3.17 | | 4.26e+005 | |
| 2366 | 275 | Unconfirmed | 12 (12) A, M, P | 0.52 | 1.14 | | 1.08e+005 | |
| 2367 | 274 | Unconfirmed | 10 (12) A, M, P | 0.36 | 1.32 | | 6.25e+004 | |
| 2368 | 273 | Unconfirmed | 4 (12) A, M, P | | | | 8.06e+004 | |
| 2369 | 272 | Unconfirmed | 12 (12) A, M, P | 0.76 | -1.08 | | 4.49e+002 | |
| 2370 | 271 | Unconfirmed | 12 (12) A, M, P | 0.74 | -1.11 | | 1.85e+002 | |
| 2371 | 270 | Unconfirmed | 12 (12) A, M, P | 0.071 | -1.37 | | 2.32e+005 | |
| 2372 | 269 | Unconfirmed | 10 (12) A, M, P | 0.60 | 1.08 | | 3.04e+005 | |
| 2373 | 268 | Unconfirmed | 12 (12) A, M, P | 0.0016 | -2.99 | | 1.02e+005 | |
| 2374 | 267 | Unconfirmed | 10 (12) A, M, P | 0.25 | -1.22 | | 1.22e+005 | |
| 2375 | 266 | Unconfirmed | 12 (12) A, M, P | 0.50 | -1.19 | | 7.80e+005 | |
| 2376 | 265 | Unconfirmed | 2 (12) A, M, P | | | | 2.16e+004 | |
| 2377 | 264 | Unconfirmed | 10 (12) A, M, P | 0.098 | 1.33 | | 8.34e+003 | |
| 2378 | 263 | Unconfirmed | 6 (12) A, M, P | | 2.42 | | 9.30e+001 | |
| [illegible] | [illegible] | [illegible] | [illegible] | [illegible] | [illegible] | | [illegible] | |

# GroEL (PAU_03756)

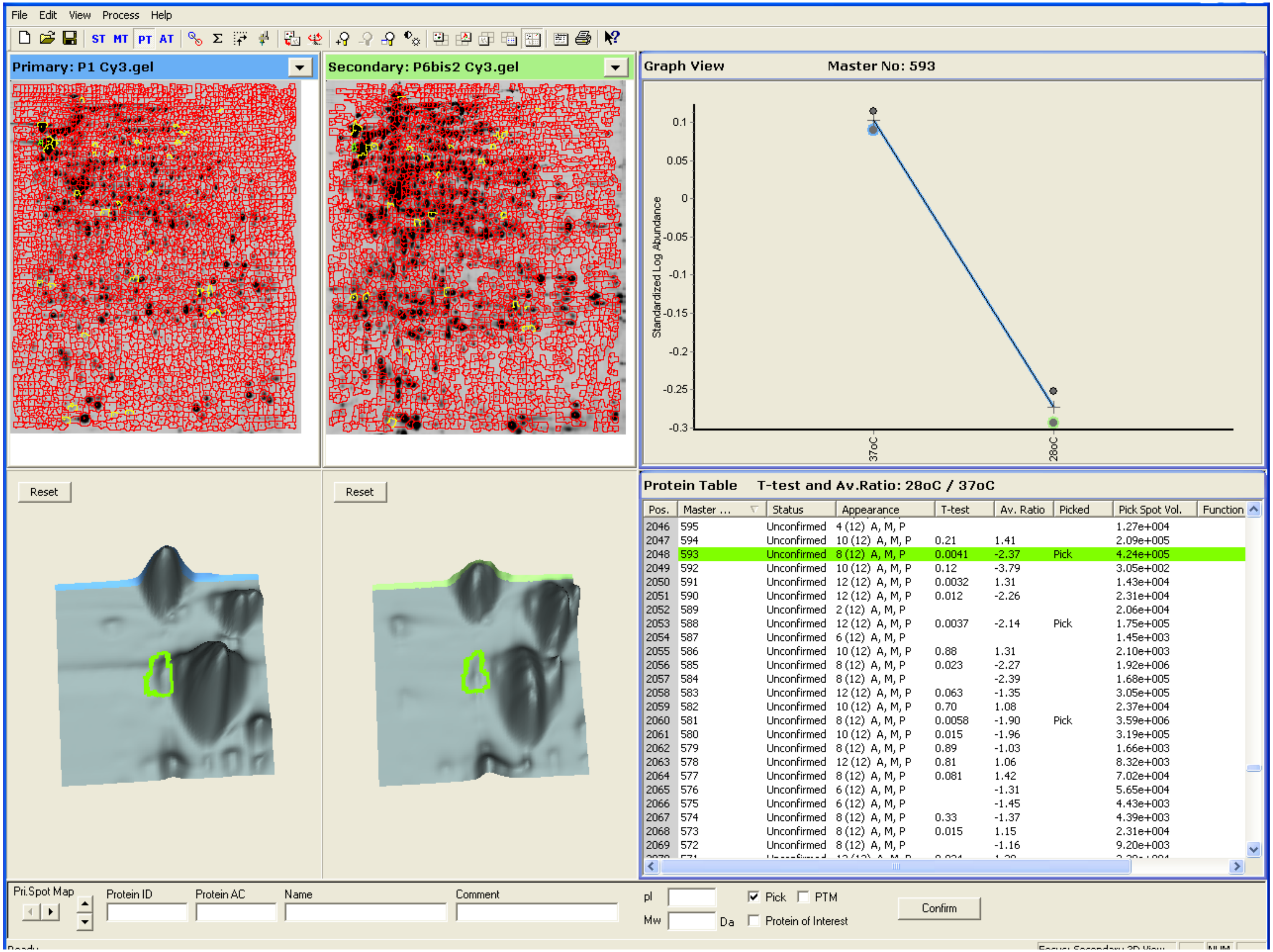

# GroES (PAU_03757)

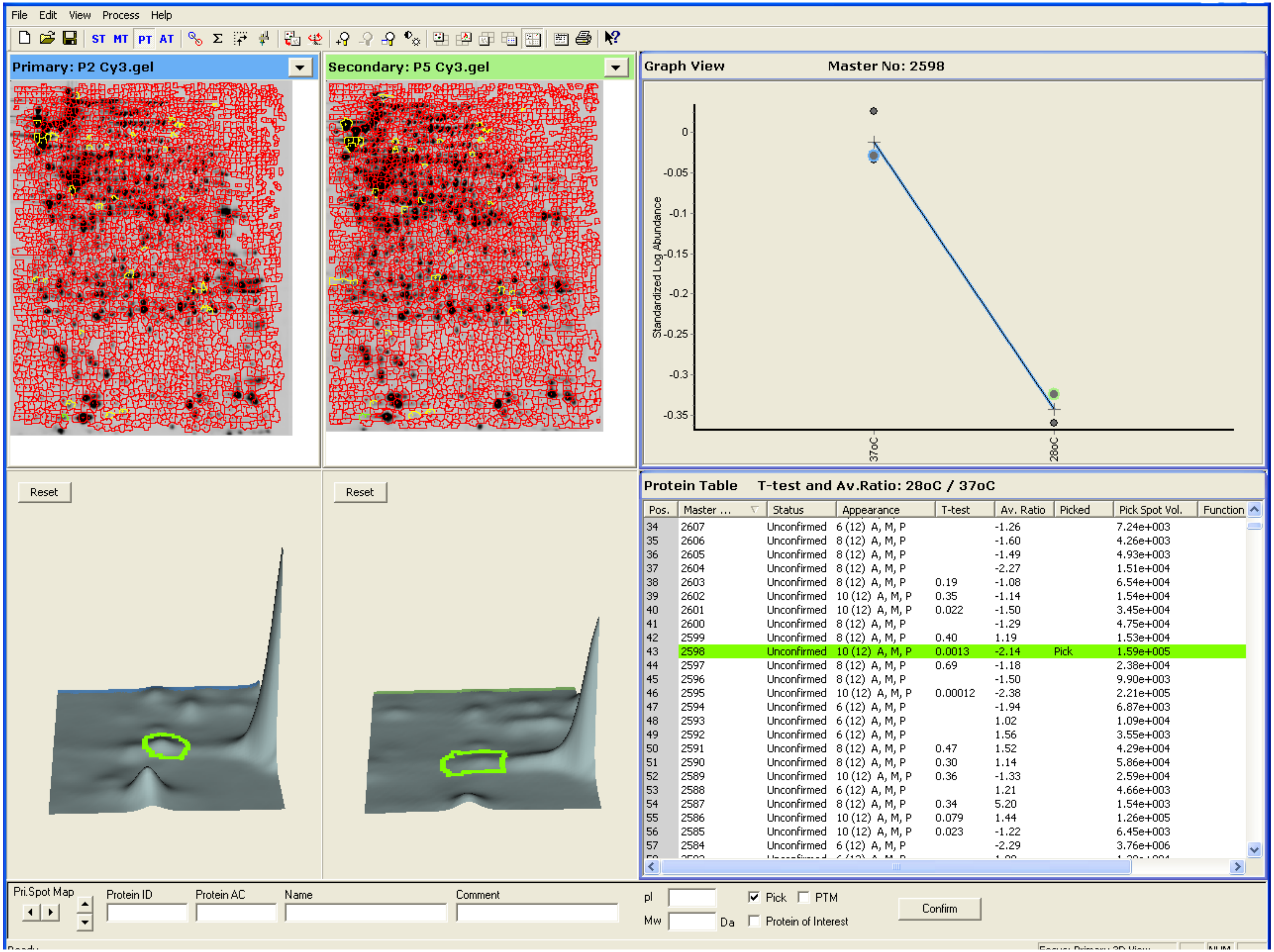

# RecA (PAU_03212)

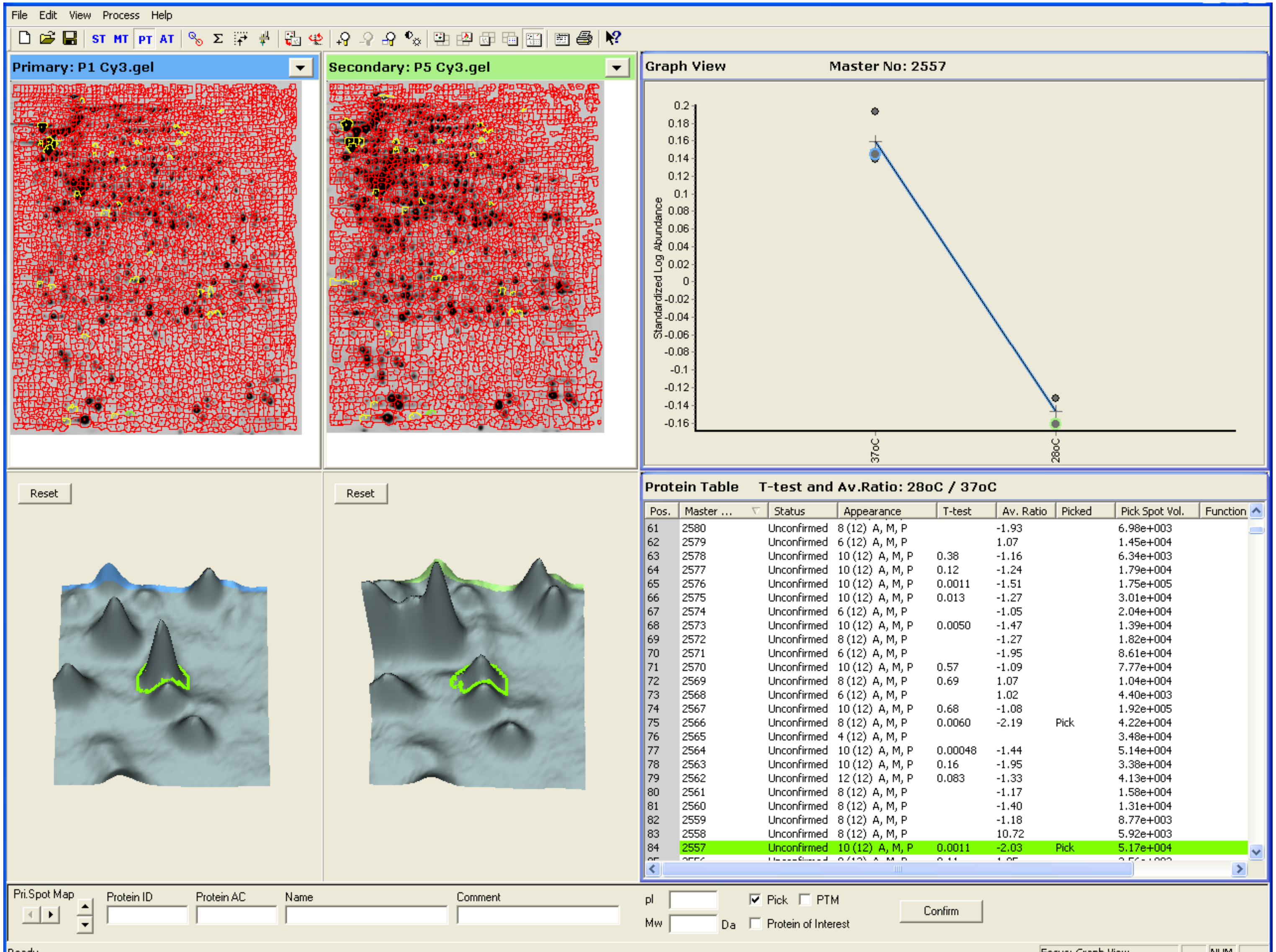

# RecA (PAU_03212)

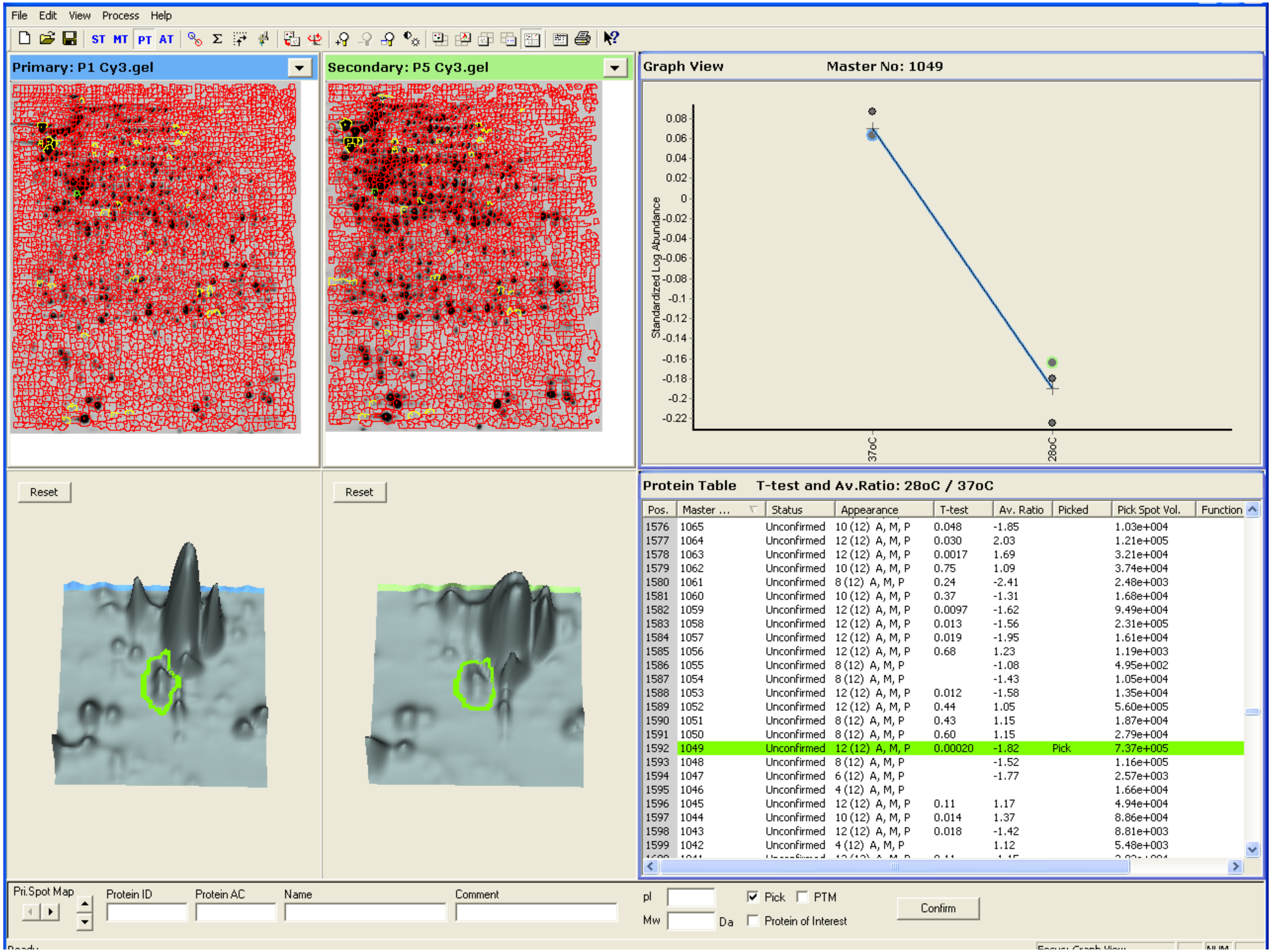

# DnaK (PAU_00543)

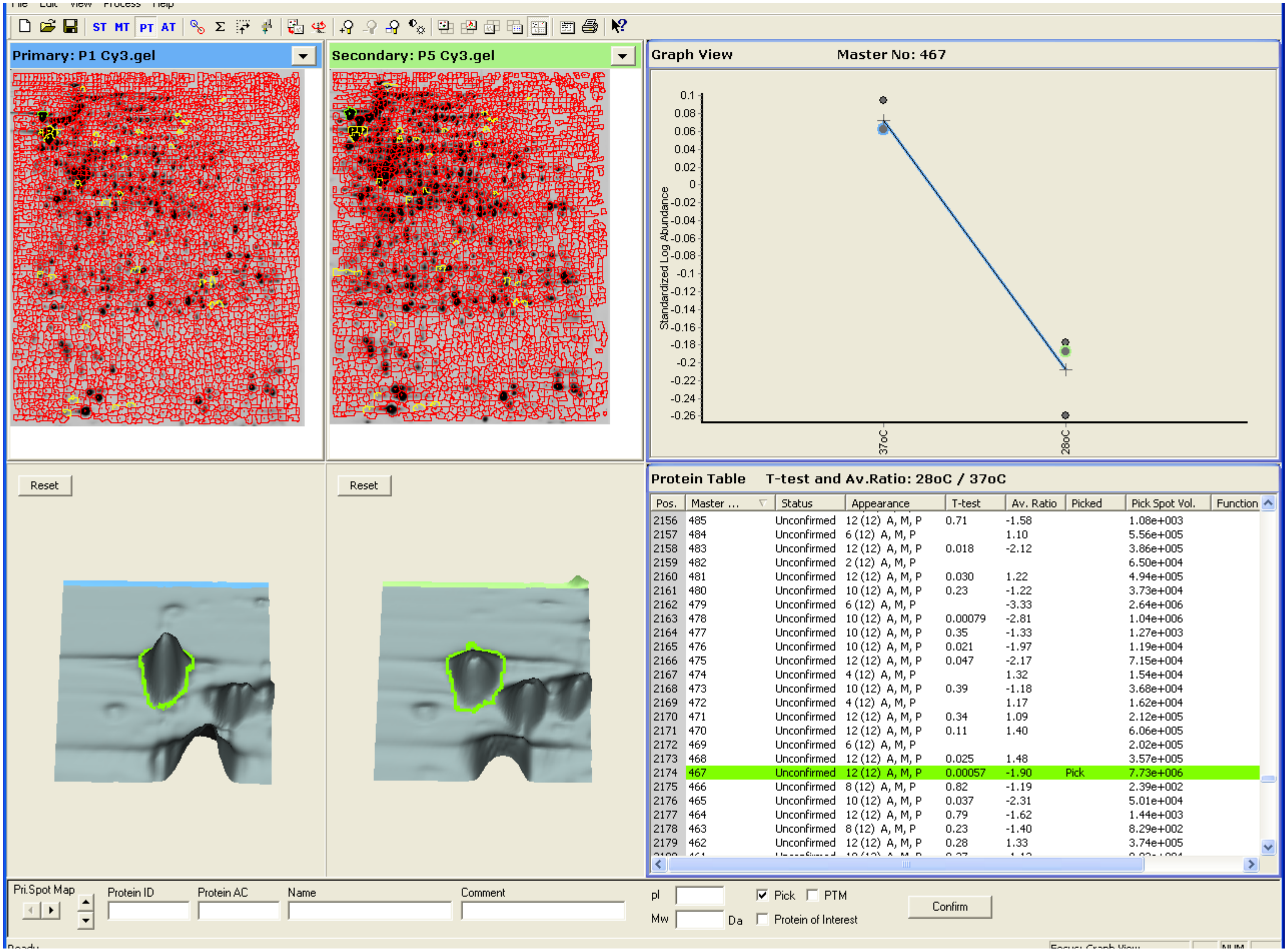

# PAU_02173

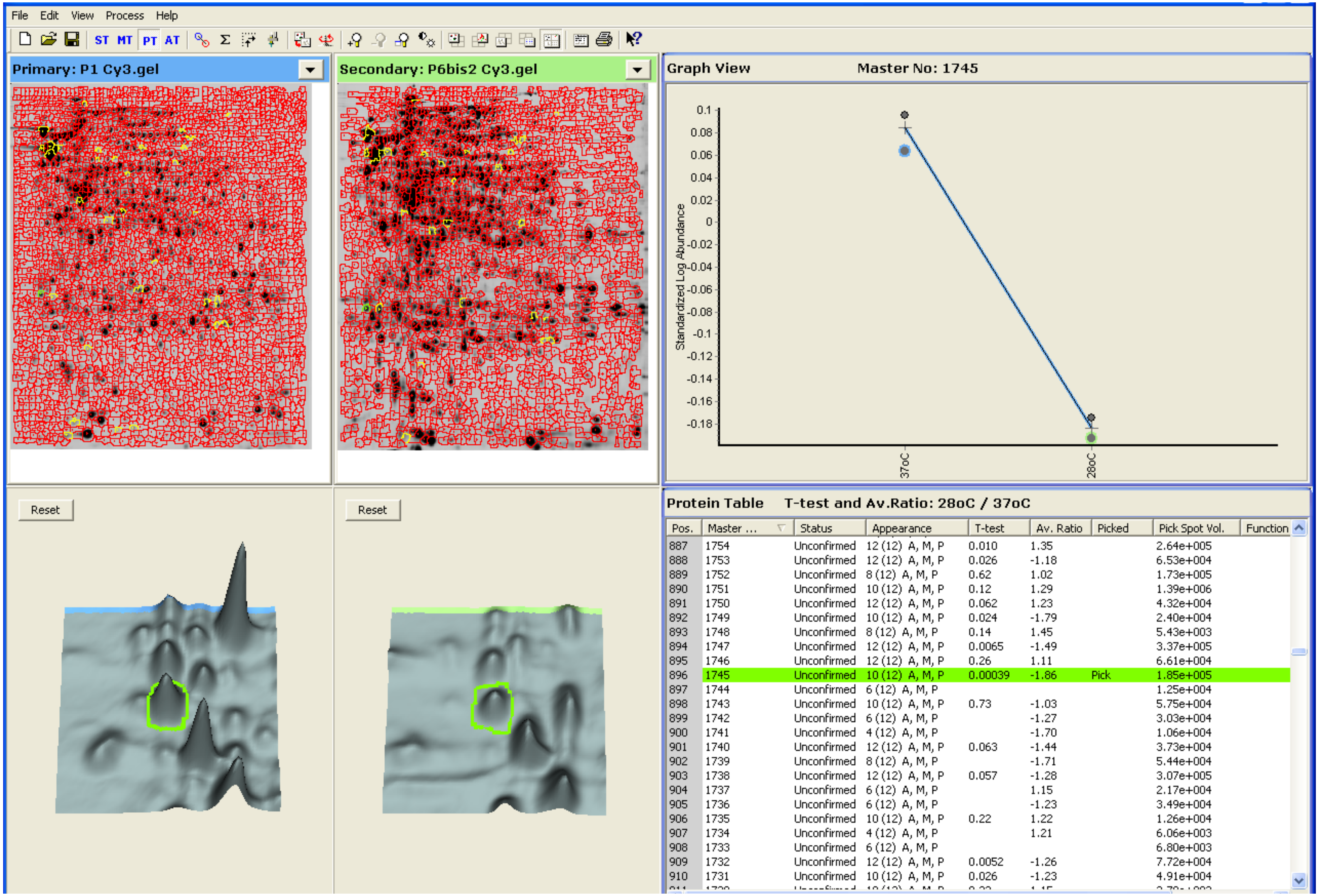

File Edit View Process Help
ST MT PT AT
Primary: P1 Cy3.gel
Secondary: P6bis2 Cy3.gel
Graph View
Master No: 1745
Standardized Log Abundance
0.1
0.08
0.06
0.04
0.02
0
-0.02
-0.04
-0.06
-0.08
-0.1
-0.12
-0.14
-0.16
-0.18
37oC
28oC
Reset
Reset
Protein Table
T-test and Av.Ratio: 28oC / 37oC
Pos. | Master ... | Status | Appearance | T-test | Av. Ratio | Picked | Pick Spot Vol. | Function
887 1754 Unconfirmed 12 (12) A, M, P 0.010 1.35 2.64e+005
888 1753 Unconfirmed 12 (12) A, M, P 0.026 -1.18 6.53e+004
889 1752 Unconfirmed 8 (12) A, M, P 0.62 1.02 1.73e+005
890 1751 Unconfirmed 10 (12) A, M, P 0.12 1.29 1.39e+006
891 1750 Unconfirmed 12 (12) A, M, P 0.062 1.23 4.32e+004
892 1749 Unconfirmed 10 (12) A, M, P 0.024 -1.79 2.40e+004
893 1748 Unconfirmed 8 (12) A, M, P 0.14 1.45 5.43e+003
894 1747 Unconfirmed 12 (12) A, M, P 0.0065 -1.49 3.37e+005
895 1746 Unconfirmed 12 (12) A, M, P 0.26 1.11 6.61e+004
896 1745 Unconfirmed 10 (12) A, M, P 0.00039 -1.86 Pick 1.85e+005
897 1744 Unconfirmed 6 (12) A, M, P 1.25e+004
898 1743 Unconfirmed 10 (12) A, M, P 0.73 -1.03 5.75e+004
899 1742 Unconfirmed 6 (12) A, M, P -1.27 3.03e+004
900 1741 Unconfirmed 4 (12) A, M, P -1.70 1.06e+004
901 1740 Unconfirmed 12 (12) A, M, P 0.063 -1.44 3.73e+004
902 1739 Unconfirmed 8 (12) A, M, P -1.71 5.44e+004
903 1738 Unconfirmed 12 (12) A, M, P 0.057 -1.28 3.07e+005
904 1737 Unconfirmed 6 (12) A, M, P 1.15 2.17e+004
905 1736 Unconfirmed 6 (12) A, M, P -1.23 3.49e+004
906 1735 Unconfirmed 10 (12) A, M, P 0.22 1.22 1.26e+004
907 1734 Unconfirmed 4 (12) A, M, P 1.21 6.06e+003
908 1733 Unconfirmed 6 (12) A, M, P 6.80e+003
909 1732 Unconfirmed 12 (12) A, M, P 0.0052 -1.26 7.72e+004
910 1731 Unconfirmed 10 (12) A, M, P 0.026 -1.23 4.91e+004

# PAU_03263

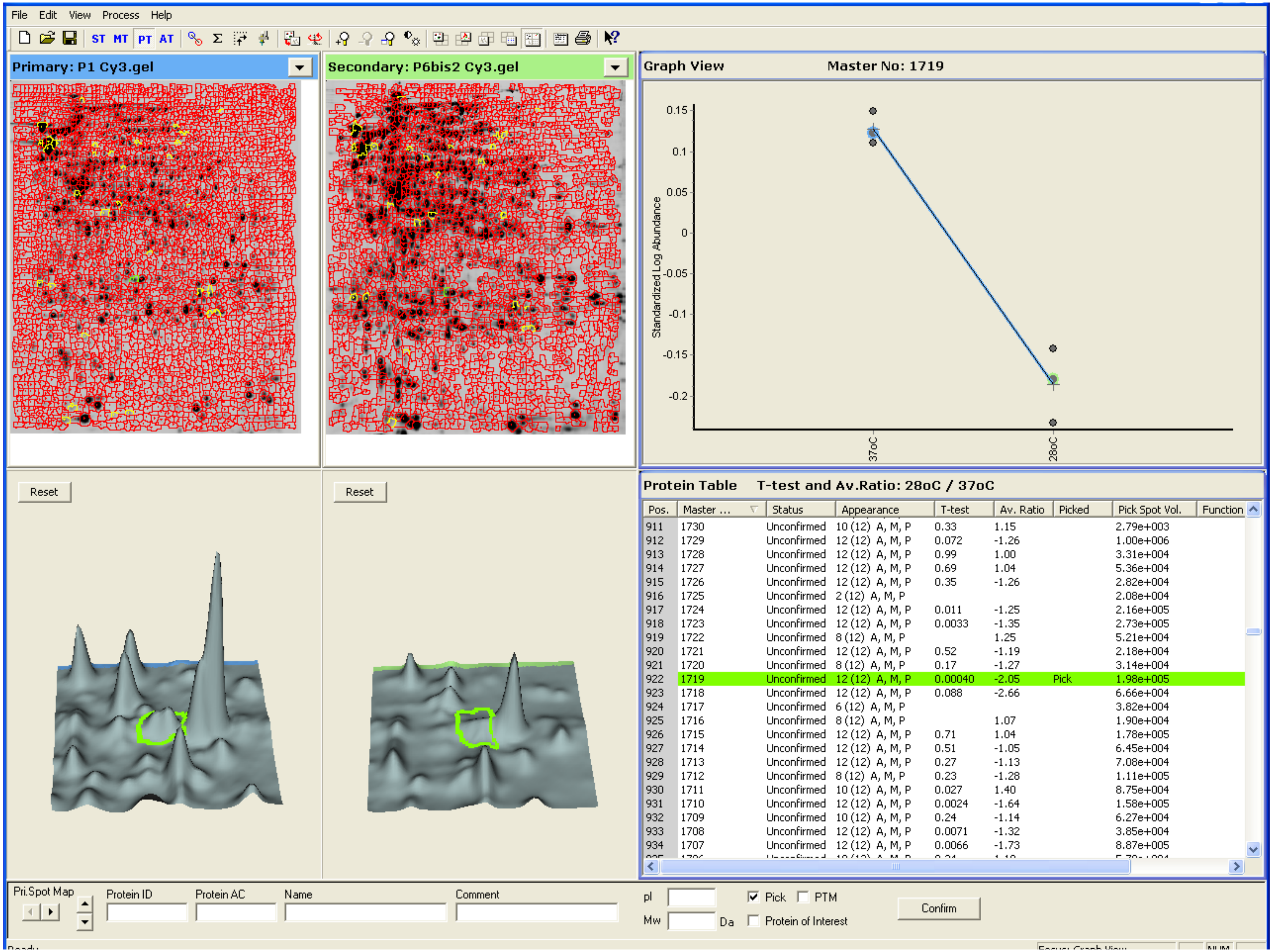
File Edit View Process Help
ST MT PT AT
Primary: P1 Cy3.gel
Secondary: P6bis2 Cy3.gel
Graph View
Master No: 1719
0.15
0.1
0.05
0
-0.05
-0.1
-0.15
-0.2
Standardized Log Abundance
37oC
28oC
Reset
Reset
Protein Table T-test and Av.Ratio: 28oC / 37oC
Pos. Master ... Status Appearance T-test Av. Ratio Picked Pick Spot Vol. Function
911 1730 Unconfirmed 10 (12) A, M, P 0.33 1.15 2.79e+003
912 1729 Unconfirmed 12 (12) A, M, P 0.072 -1.26 1.00e+006
913 1728 Unconfirmed 12 (12) A, M, P 0.99 1.00 3.31e+004
914 1727 Unconfirmed 12 (12) A, M, P 0.69 1.04 5.36e+004
915 1726 Unconfirmed 12 (12) A, M, P 0.35 -1.26 2.82e+004
916 1725 Unconfirmed 2 (12) A, M, P 2.08e+004
917 1724 Unconfirmed 12 (12) A, M, P 0.011 -1.25 2.16e+005
918 1723 Unconfirmed 12 (12) A, M, P 0.0033 -1.35 2.73e+005
919 1722 Unconfirmed 8 (12) A, M, P 1.25 5.21e+004
920 1721 Unconfirmed 12 (12) A, M, P 0.52 -1.19 2.18e+004
921 1720 Unconfirmed 8 (12) A, M, P 0.17 -1.27 3.14e+004
922 1719 Unconfirmed 12 (12) A, M, P 0.00040 -2.05 Pick 1.98e+005
923 1718 Unconfirmed 12 (12) A, M, P 0.088 -2.66 6.66e+004
924 1717 Unconfirmed 6 (12) A, M, P 3.82e+004
925 1716 Unconfirmed 8 (12) A, M, P 1.07 1.90e+004
926 1715 Unconfirmed 12 (12) A, M, P 0.71 1.04 1.78e+005
927 1714 Unconfirmed 12 (12) A, M, P 0.51 -1.05 6.45e+004
928 1713 Unconfirmed 12 (12) A, M, P 0.27 -1.13 7.08e+004
929 1712 Unconfirmed 8 (12) A, M, P 0.23 -1.28 1.11e+005
930 1711 Unconfirmed 10 (12) A, M, P 0.027 1.40 8.75e+004
931 1710 Unconfirmed 12 (12) A, M, P 0.0024 -1.64 1.58e+005
932 1709 Unconfirmed 10 (12) A, M, P 0.24 -1.14 6.27e+004
933 1708 Unconfirmed 12 (12) A, M, P 0.0071 -1.32 3.85e+004
934 1707 Unconfirmed 12 (12) A, M, P 0.0066 -1.73 8.87e+005
Pri.Spot Map
Protein ID
Protein AC
Name
Comment
pI
Mw
Da
Pick
PTM
Protein of Interest
Confirm

# PAU_02575

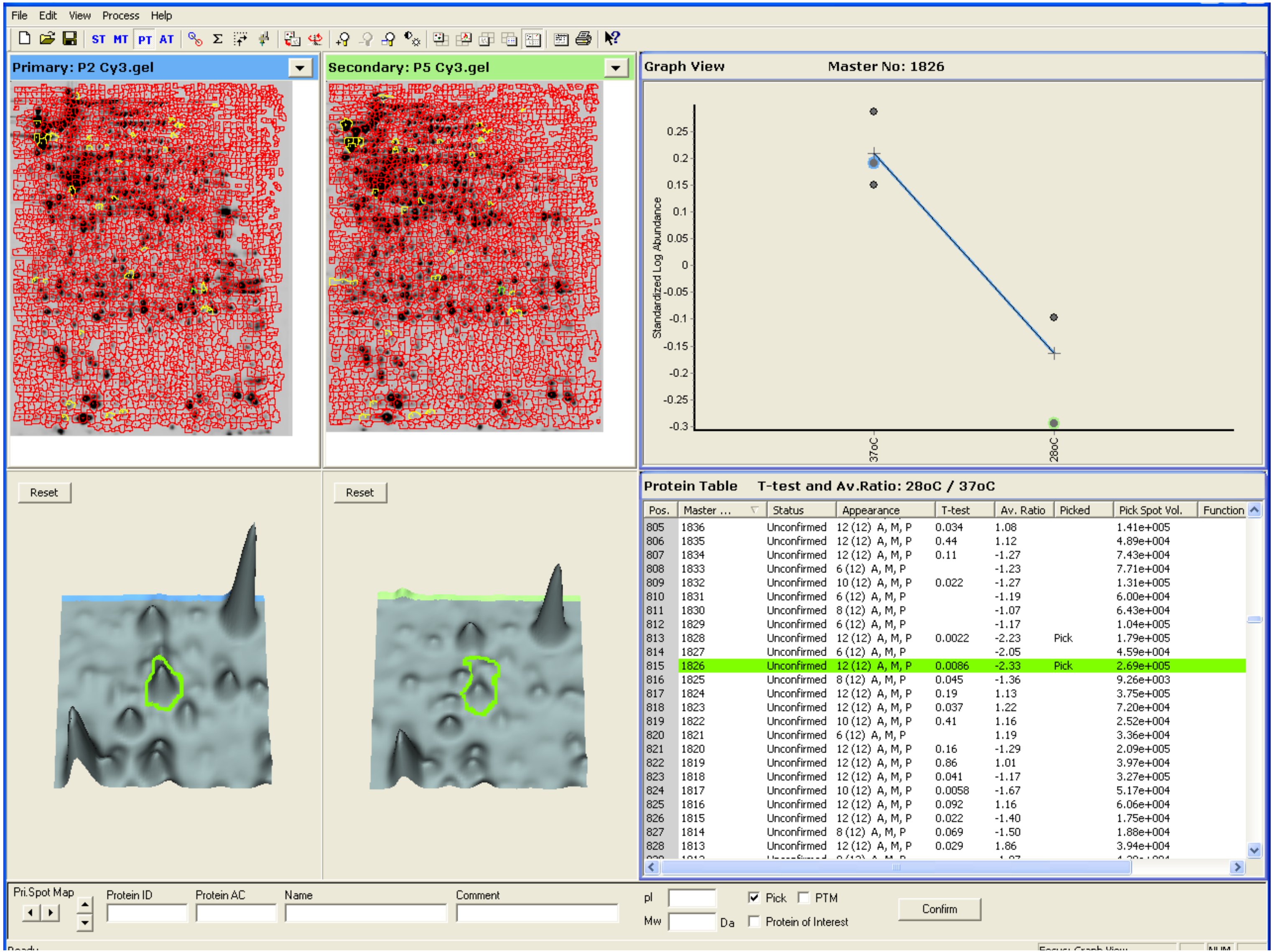
File Edit View Process Help
ST MT PT AT
Primary: P2 Cy3.gel
Secondary: P5 Cy3.gel
Graph View
Master No: 1826
Standardized Log Abundance
0.25
0.2
0.15
0.1
0.05
0
-0.05
-0.1
-0.15
-0.2
-0.25
-0.3
37oC
28oC
Reset
Reset
Protein Table T-test and Av.Ratio: 28oC / 37oC
Pos. Master ... Status Appearance T-test Av. Ratio Picked Pick Spot Vol. Function
805 1836 Unconfirmed 12 (12) A, M, P 0.034 1.08 1.41e+005
806 1835 Unconfirmed 12 (12) A, M, P 0.44 1.12 4.89e+004
807 1834 Unconfirmed 12 (12) A, M, P 0.11 -1.27 7.43e+004
808 1833 Unconfirmed 6 (12) A, M, P -1.23 7.71e+004
809 1832 Unconfirmed 10 (12) A, M, P 0.022 -1.27 1.31e+005
810 1831 Unconfirmed 6 (12) A, M, P -1.19 6.00e+004
811 1830 Unconfirmed 8 (12) A, M, P -1.07 6.43e+004
812 1829 Unconfirmed 6 (12) A, M, P -1.17 1.04e+005
813 1828 Unconfirmed 12 (12) A, M, P 0.0022 -2.23 Pick 1.79e+005
814 1827 Unconfirmed 6 (12) A, M, P -2.05 4.59e+004
815 1826 Unconfirmed 12 (12) A, M, P 0.0086 -2.33 Pick 2.69e+005
816 1825 Unconfirmed 8 (12) A, M, P 0.045 -1.36 9.26e+003
817 1824 Unconfirmed 12 (12) A, M, P 0.19 1.13 3.75e+005
818 1823 Unconfirmed 12 (12) A, M, P 0.037 1.22 7.20e+004
819 1822 Unconfirmed 10 (12) A, M, P 0.41 1.16 2.52e+004
820 1821 Unconfirmed 6 (12) A, M, P 1.19 3.36e+004
821 1820 Unconfirmed 12 (12) A, M, P 0.16 -1.29 2.09e+005
822 1819 Unconfirmed 12 (12) A, M, P 0.86 1.01 3.97e+004
823 1818 Unconfirmed 12 (12) A, M, P 0.041 -1.17 3.27e+005
824 1817 Unconfirmed 10 (12) A, M, P 0.0058 -1.67 5.17e+004
825 1816 Unconfirmed 12 (12) A, M, P 0.092 1.16 6.06e+004
826 1815 Unconfirmed 12 (12) A, M, P 0.022 -1.40 1.75e+004
827 1814 Unconfirmed 8 (12) A, M, P 0.069 -1.50 1.88e+004
828 1813 Unconfirmed 12 (12) A, M, P 0.029 1.86 3.94e+004
Pri.Spot Map
Protein ID
Protein AC
Name
Comment
pI
Mw
Da
Pick
PTM
Protein of Interest
Confirm

# PAU_03286

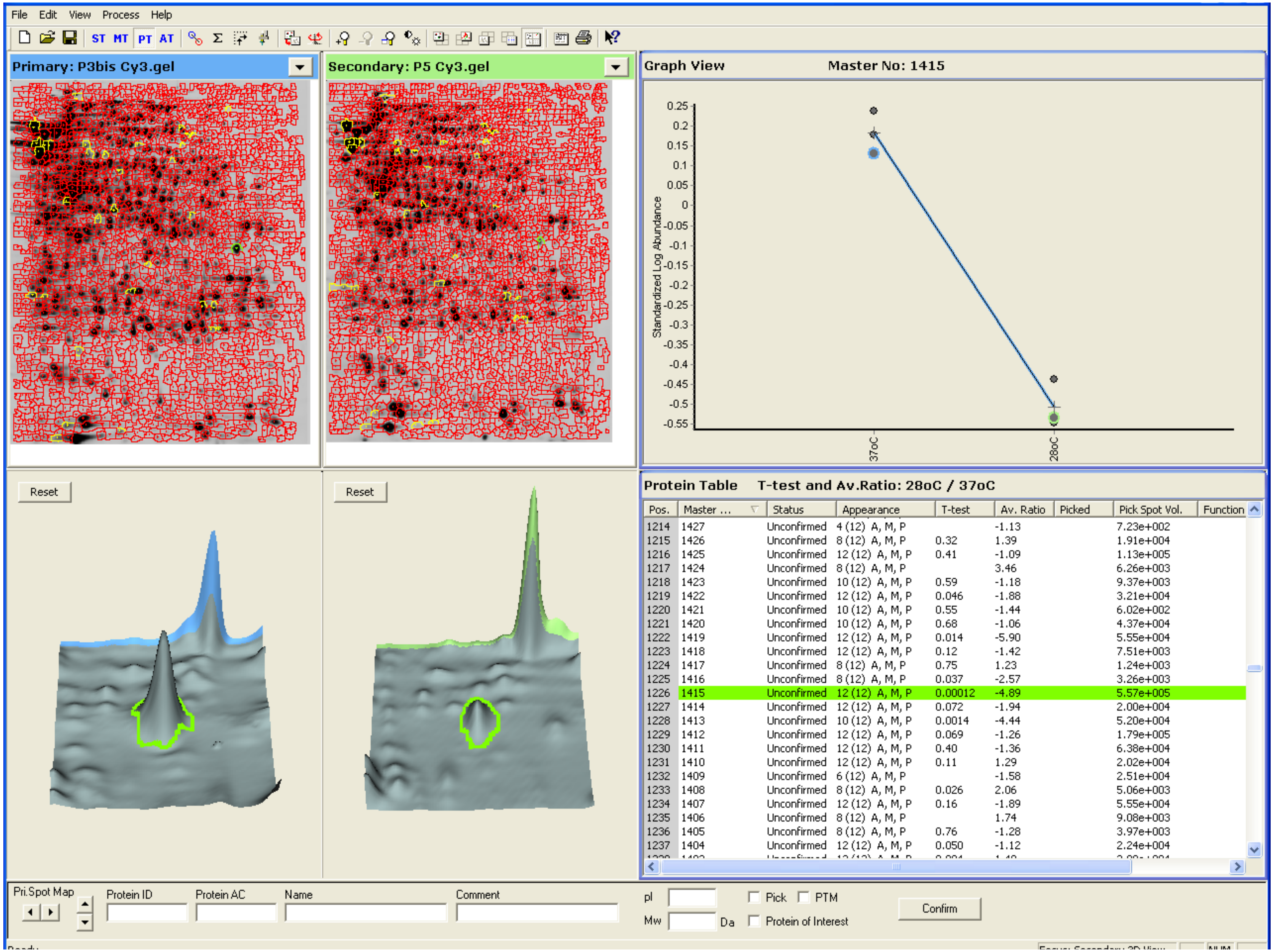

File Edit View Process Help
ST MT PT AT
Primary: P3bis Cy3.gel
Secondary: P5 Cy3.gel
Graph View
Master No: 1415
Standardized Log Abundance
0.25
0.2
0.15
0.1
0.05
0
-0.05
-0.1
-0.15
-0.2
-0.25
-0.3
-0.35
-0.4
-0.45
-0.5
-0.55
37oC
28oC
Reset
Reset
Protein Table
T-test and Av.Ratio: 28oC / 37oC
Pos. Master ... Status Appearance T-test Av. Ratio Picked Pick Spot Vol. Function
1214 1427 Unconfirmed 4 (12) A, M, P -1.13 7.23e+002
1215 1426 Unconfirmed 8 (12) A, M, P 0.32 1.39 1.91e+004
1216 1425 Unconfirmed 12 (12) A, M, P 0.41 -1.09 1.13e+005
1217 1424 Unconfirmed 8 (12) A, M, P 3.46 6.26e+003
1218 1423 Unconfirmed 10 (12) A, M, P 0.59 -1.18 9.37e+003
1219 1422 Unconfirmed 12 (12) A, M, P 0.046 -1.88 3.21e+004
1220 1421 Unconfirmed 10 (12) A, M, P 0.55 -1.44 6.02e+002
1221 1420 Unconfirmed 10 (12) A, M, P 0.68 -1.06 4.37e+004
1222 1419 Unconfirmed 12 (12) A, M, P 0.014 -5.90 5.55e+004
1223 1418 Unconfirmed 12 (12) A, M, P 0.12 -1.42 7.51e+003
1224 1417 Unconfirmed 8 (12) A, M, P 0.75 1.23 1.24e+003
1225 1416 Unconfirmed 8 (12) A, M, P 0.037 -2.57 3.26e+003
1226 1415 Unconfirmed 12 (12) A, M, P 0.00012 -4.89 5.57e+005
1227 1414 Unconfirmed 12 (12) A, M, P 0.072 -1.94 2.00e+004
1228 1413 Unconfirmed 10 (12) A, M, P 0.0014 -4.44 5.20e+004
1229 1412 Unconfirmed 12 (12) A, M, P 0.069 -1.26 1.79e+005
1230 1411 Unconfirmed 12 (12) A, M, P 0.40 -1.36 6.38e+004
1231 1410 Unconfirmed 12 (12) A, M, P 0.11 1.29 2.02e+004
1232 1409 Unconfirmed 6 (12) A, M, P -1.58 2.51e+004
1233 1408 Unconfirmed 8 (12) A, M, P 0.026 2.06 5.06e+003
1234 1407 Unconfirmed 12 (12) A, M, P 0.16 -1.89 5.55e+004
1235 1406 Unconfirmed 8 (12) A, M, P 1.74 9.08e+003
1236 1405 Unconfirmed 8 (12) A, M, P 0.76 -1.28 3.97e+003
1237 1404 Unconfirmed 12 (12) A, M, P 0.050 -1.12 2.24e+004
Pri.Spot Map
Protein ID
Protein AC
Name
Comment
pI
Mw
Da
Pick
PTM
Protein of Interest
Confirm

# PAU_03286

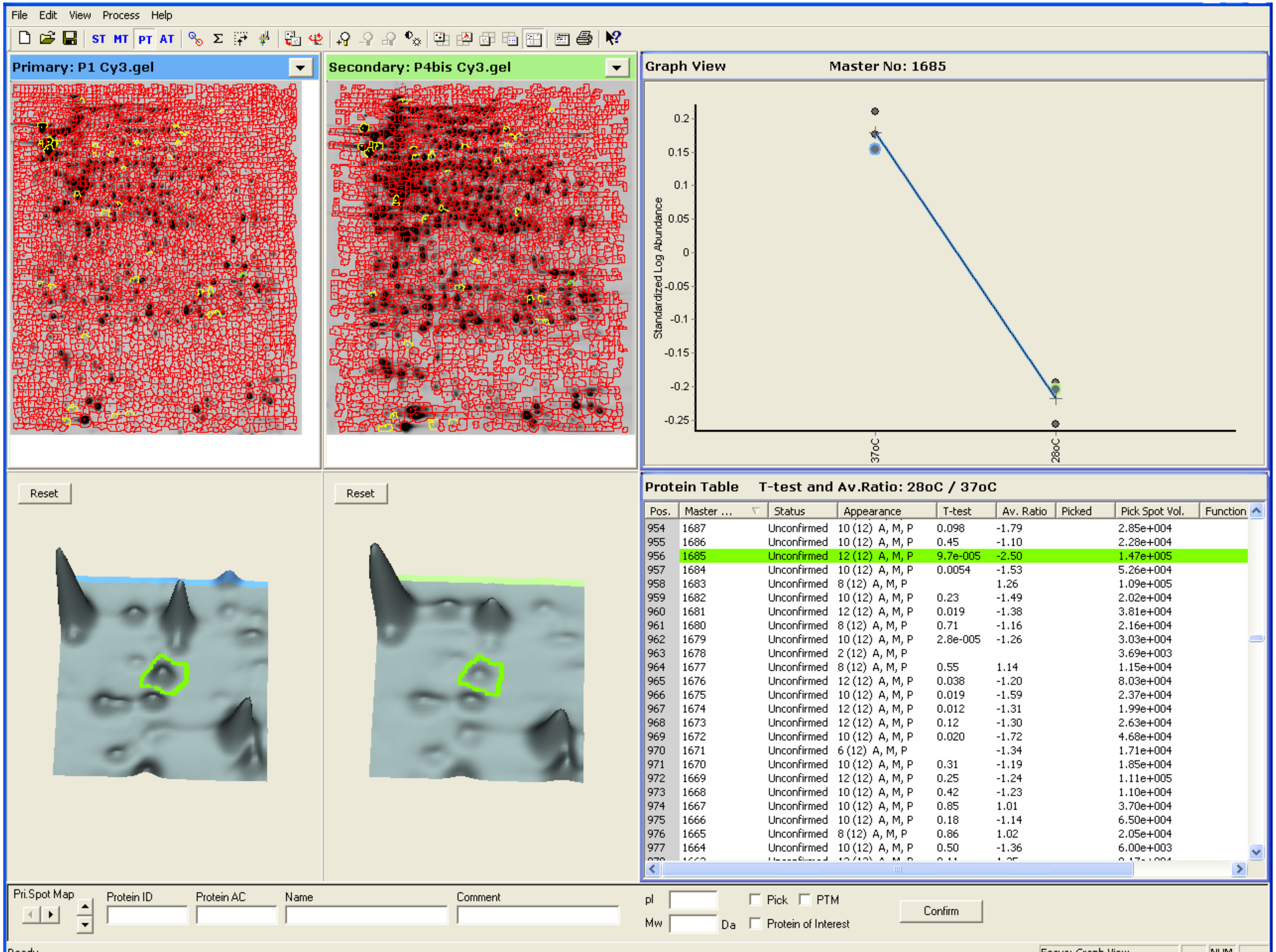

File Edit View Process Help
ST MT PT AT
Primary: P1 Cy3.gel
Secondary: P4bis Cy3.gel
Graph View
Master No: 1685
Standardized Log Abundance
0.2
0.15
0.1
0.05
0
-0.05
-0.1
-0.15
-0.2
-0.25
37oC
28oC
Reset
Reset
Protein Table
T-test and Av.Ratio: 28oC / 37oC
Pos. Master ... Status Appearance T-test Av. Ratio Picked Pick Spot Vol. Function
954 1687 Unconfirmed 10 (12) A, M, P 0.098 -1.79 2.85e+004
955 1686 Unconfirmed 10 (12) A, M, P 0.45 -1.10 2.28e+004
956 1685 Unconfirmed 12 (12) A, M, P 9.7e-005 -2.50 1.47e+005
957 1684 Unconfirmed 10 (12) A, M, P 0.0054 -1.53 5.26e+004
958 1683 Unconfirmed 8 (12) A, M, P 1.26 1.09e+005
959 1682 Unconfirmed 10 (12) A, M, P 0.23 -1.49 2.02e+004
960 1681 Unconfirmed 12 (12) A, M, P 0.019 -1.38 3.81e+004
961 1680 Unconfirmed 8 (12) A, M, P 0.71 -1.16 2.16e+004
962 1679 Unconfirmed 10 (12) A, M, P 2.8e-005 -1.26 3.03e+004
963 1678 Unconfirmed 2 (12) A, M, P 3.69e+003
964 1677 Unconfirmed 8 (12) A, M, P 0.55 1.14 1.15e+004
965 1676 Unconfirmed 12 (12) A, M, P 0.038 -1.20 8.03e+004
966 1675 Unconfirmed 10 (12) A, M, P 0.019 -1.59 2.37e+004
967 1674 Unconfirmed 12 (12) A, M, P 0.012 -1.31 1.99e+004
968 1673 Unconfirmed 12 (12) A, M, P 0.12 -1.30 2.63e+004
969 1672 Unconfirmed 10 (12) A, M, P 0.020 -1.72 4.68e+004
970 1671 Unconfirmed 6 (12) A, M, P -1.34 1.71e+004
971 1670 Unconfirmed 10 (12) A, M, P 0.31 -1.19 1.85e+004
972 1669 Unconfirmed 12 (12) A, M, P 0.25 -1.24 1.11e+005
973 1668 Unconfirmed 10 (12) A, M, P 0.42 -1.23 1.10e+004
974 1667 Unconfirmed 10 (12) A, M, P 0.85 1.01 3.70e+004
975 1666 Unconfirmed 10 (12) A, M, P 0.18 -1.14 6.50e+004
976 1665 Unconfirmed 8 (12) A, M, P 0.86 1.02 2.05e+004
977 1664 Unconfirmed 10 (12) A, M, P 0.50 -1.36 6.00e+003
Pri.Spot Map
Protein ID
Protein AC
Name
Comment
pI
Mw
Da
Pick
PTM
Protein of Interest
Confirm

# GmhA (PAU_03260)

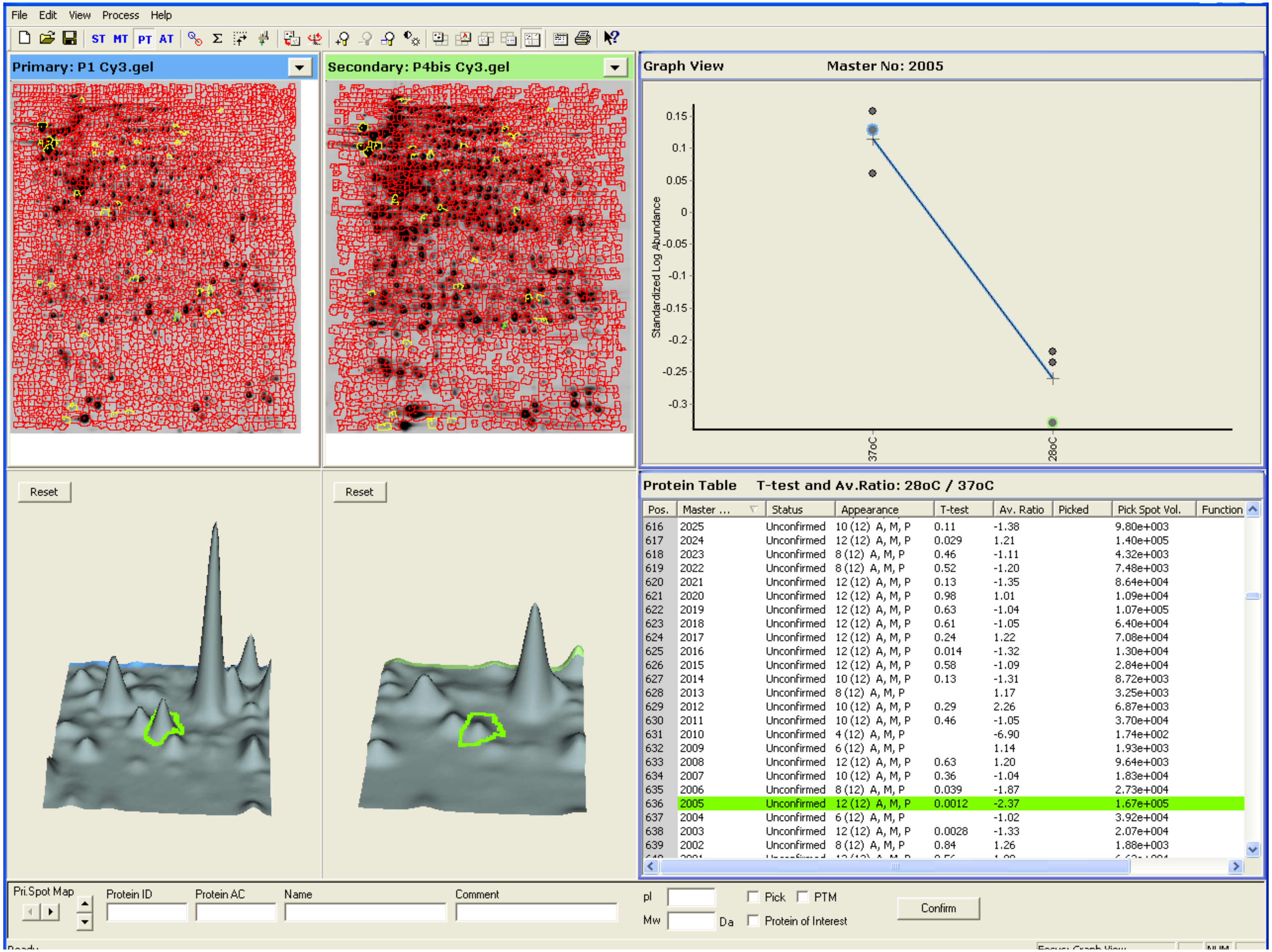

# Bcp (PAU_01793)

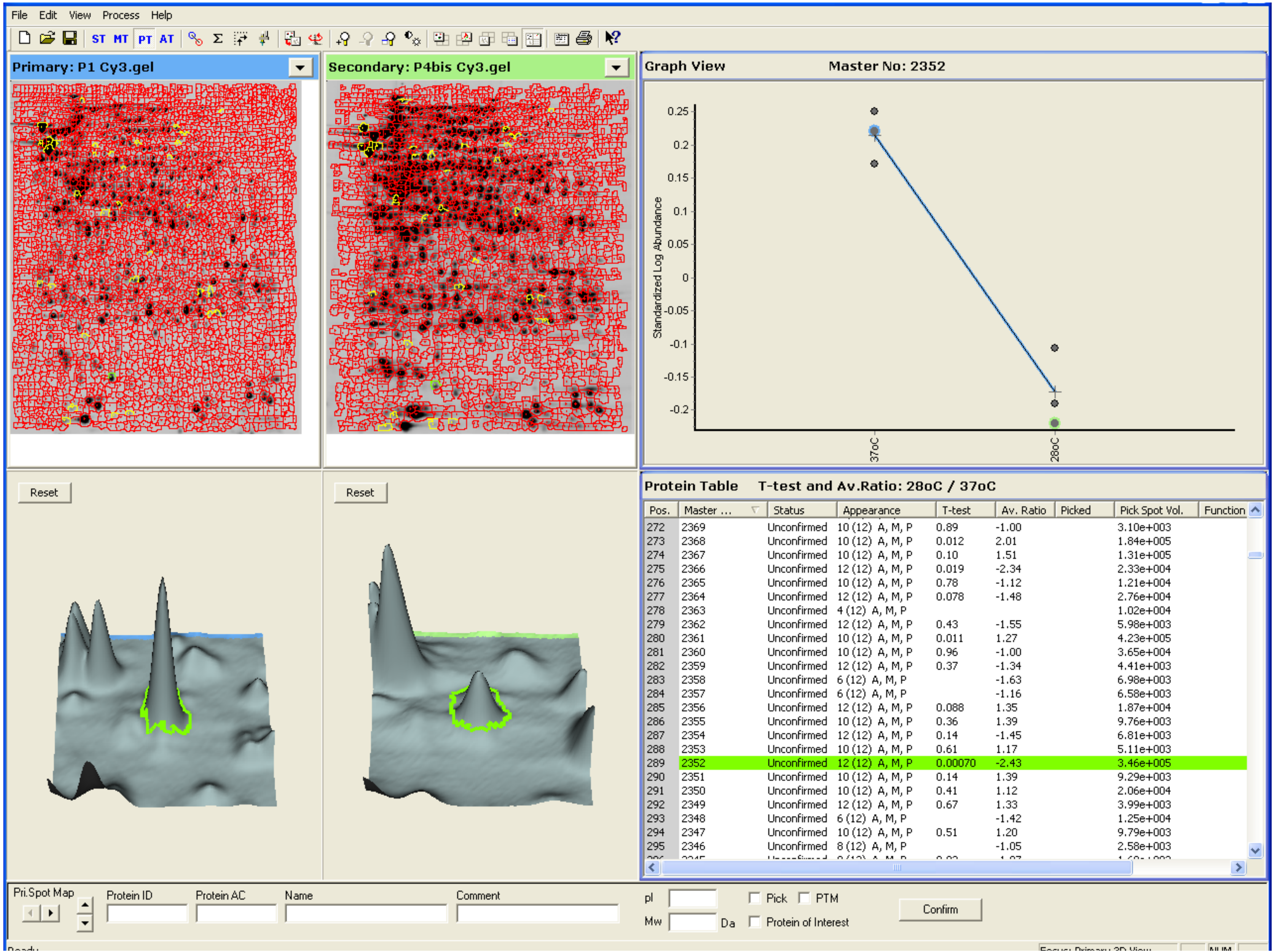

# YneB (PAU_01469)

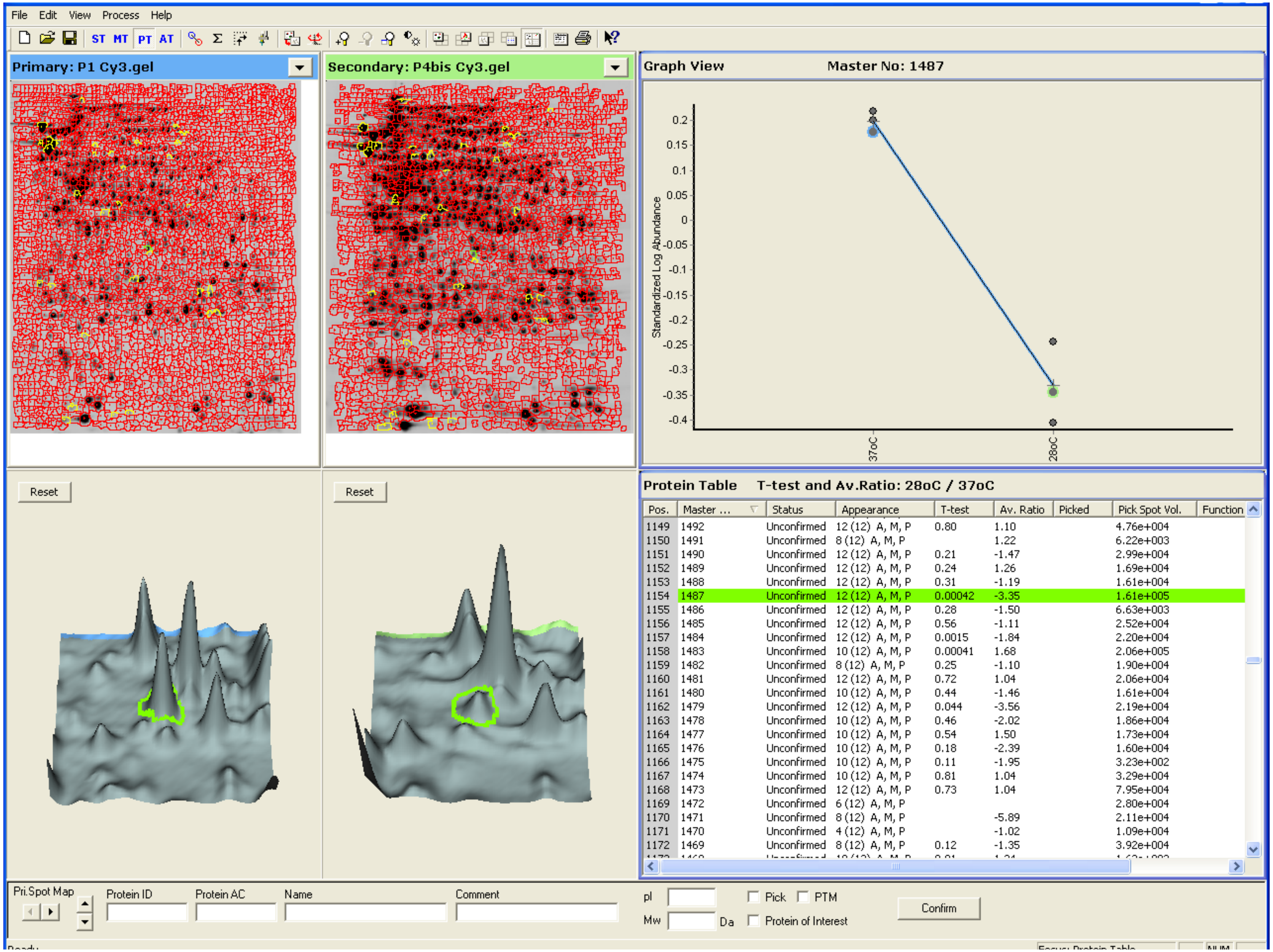

# Gst (PAU_01940)

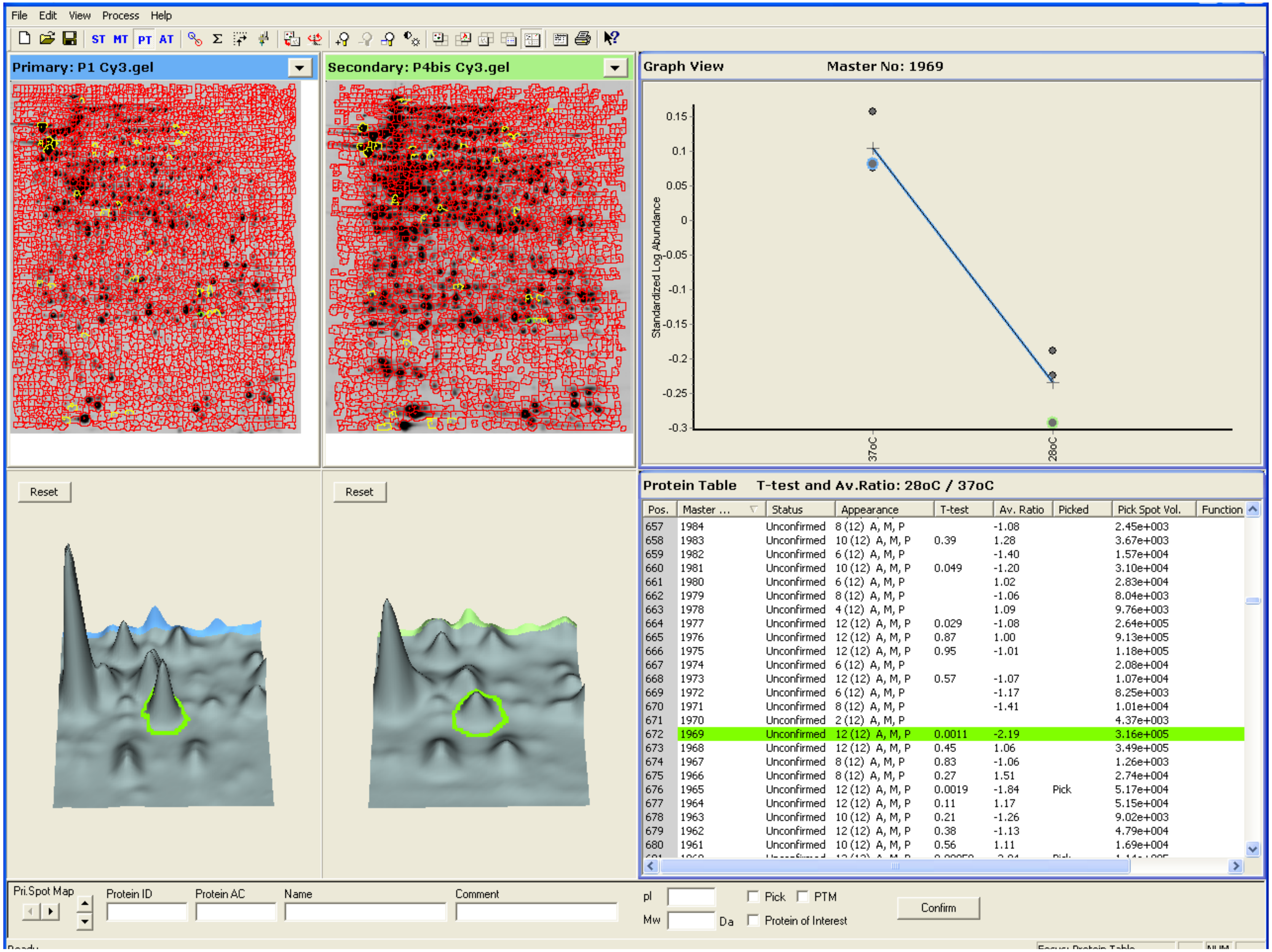

# AnsB (PAU_02834)

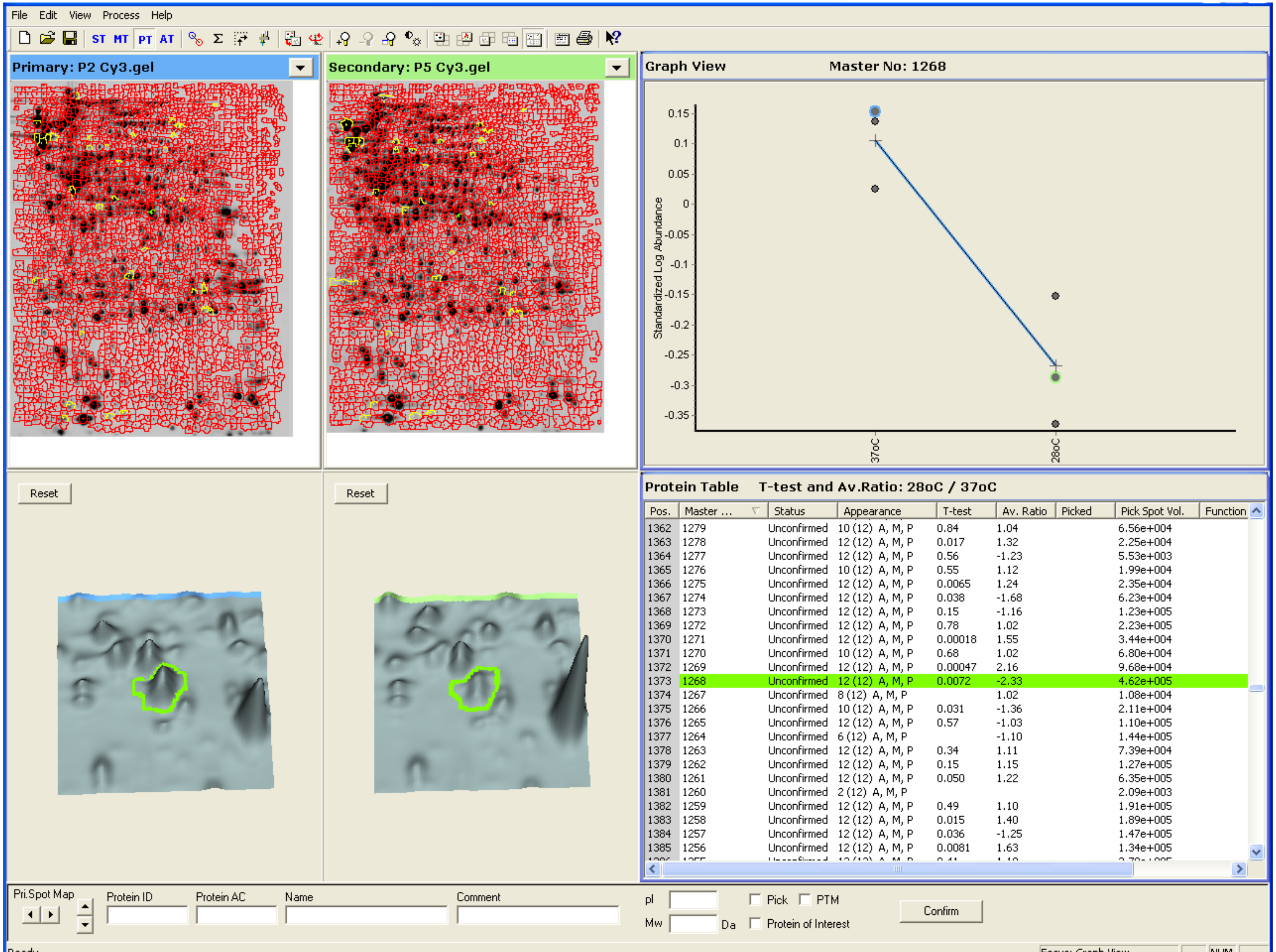

# GuaA (PAU_01827)

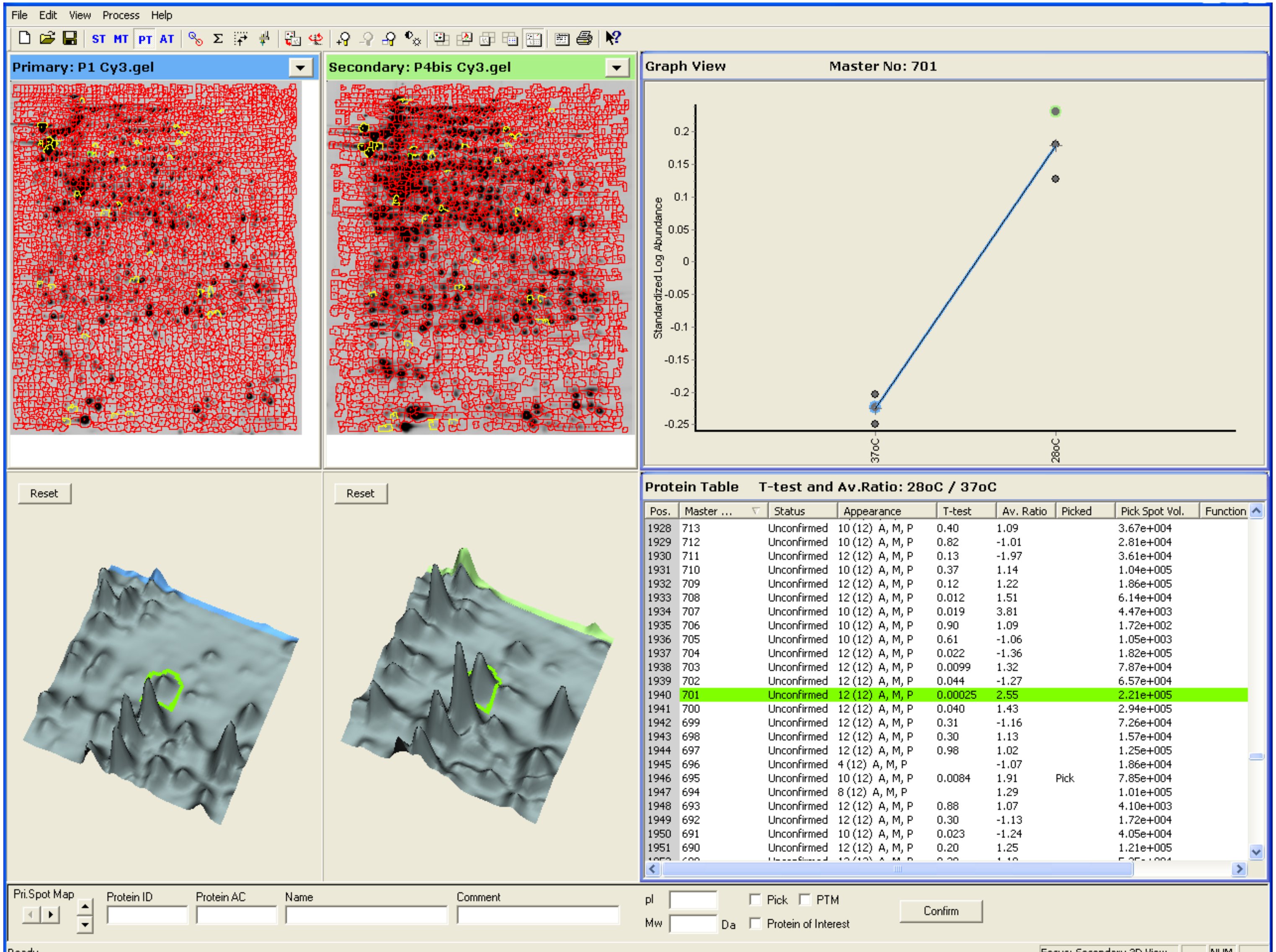

# GuaB (PAU_01826)

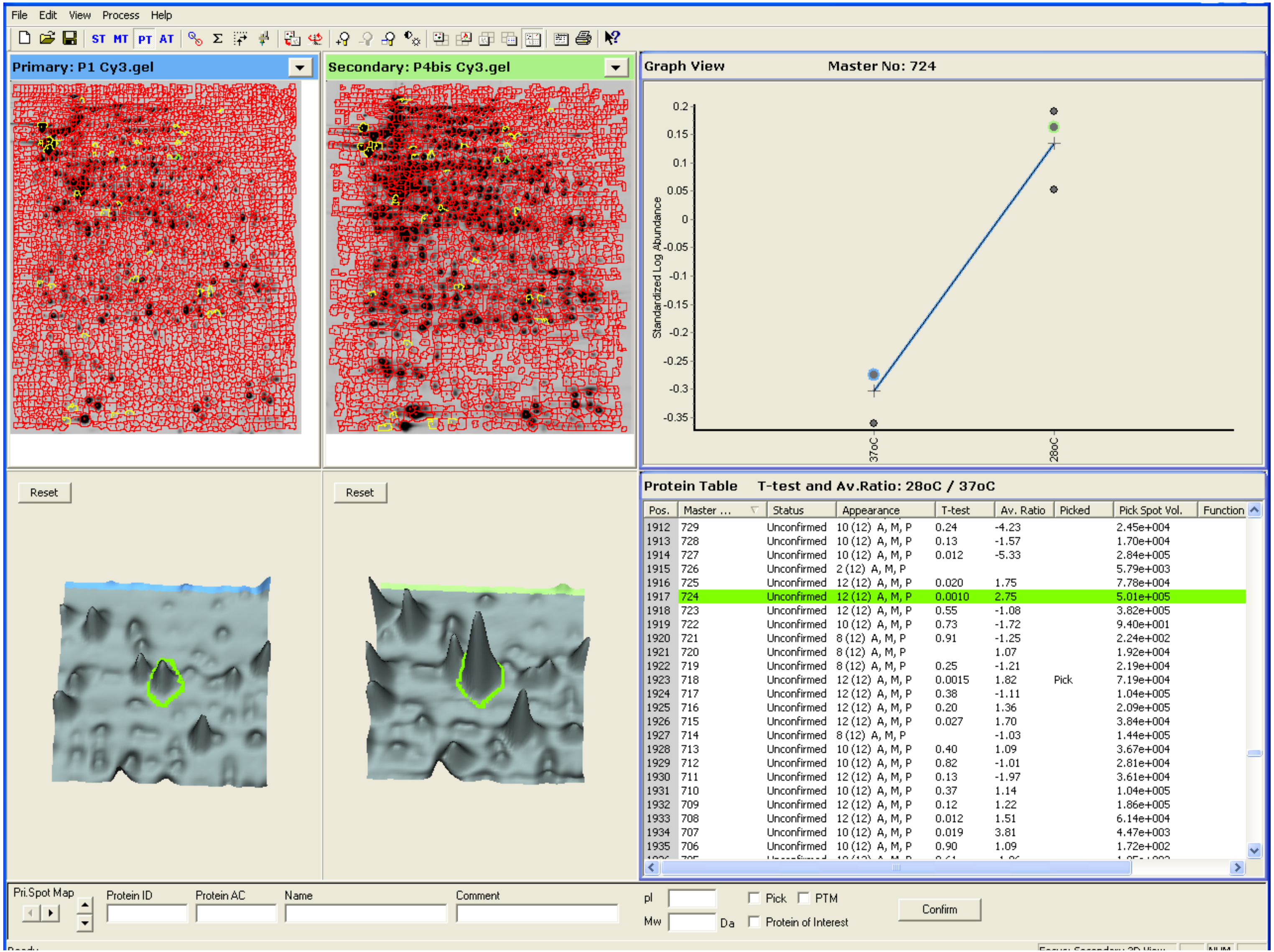

# MalE (PAU_00367)

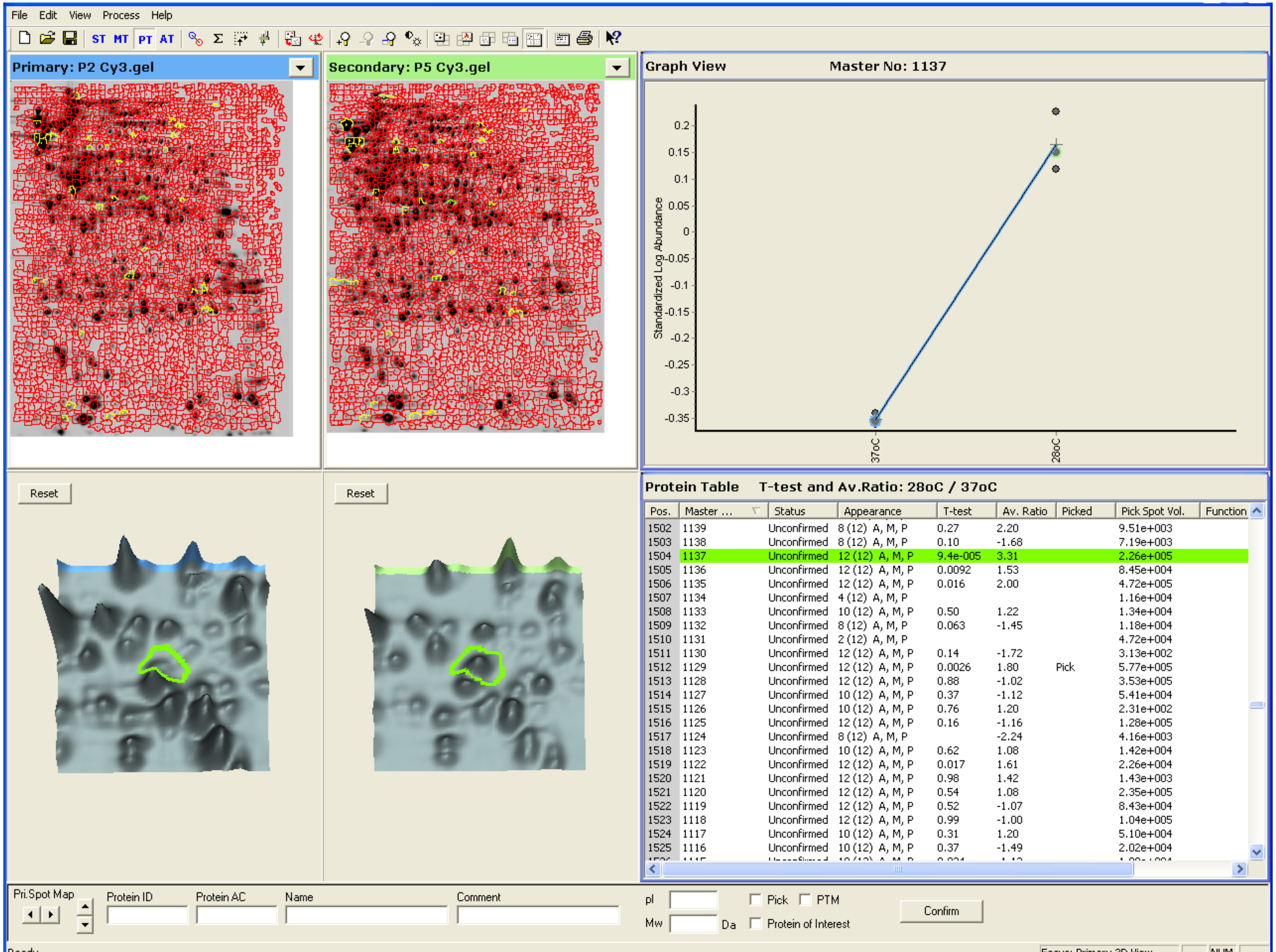

# PurH (PAU_00406)

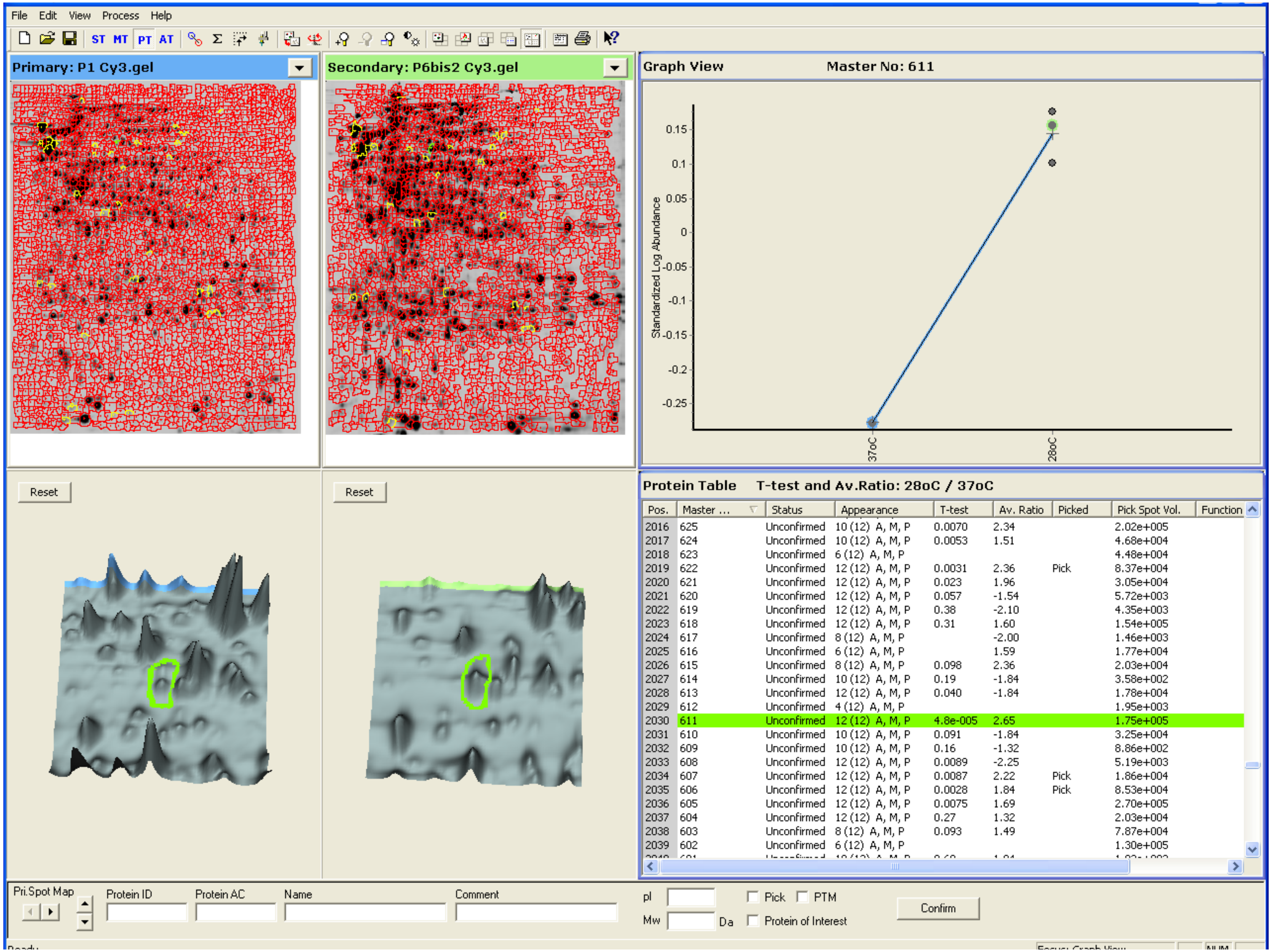

# PckA (PAU_00083)

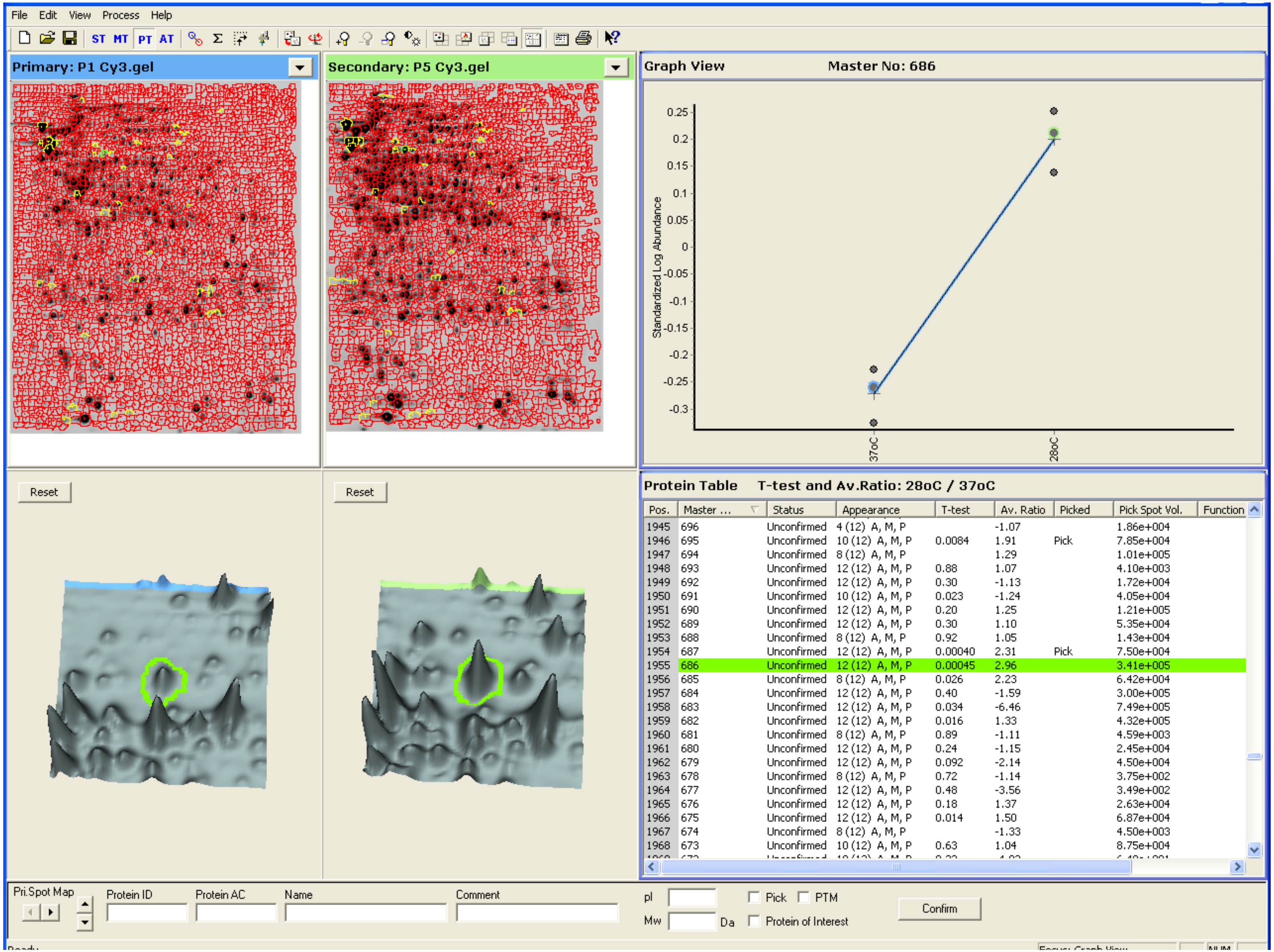

# GcvT (PAU_01159)

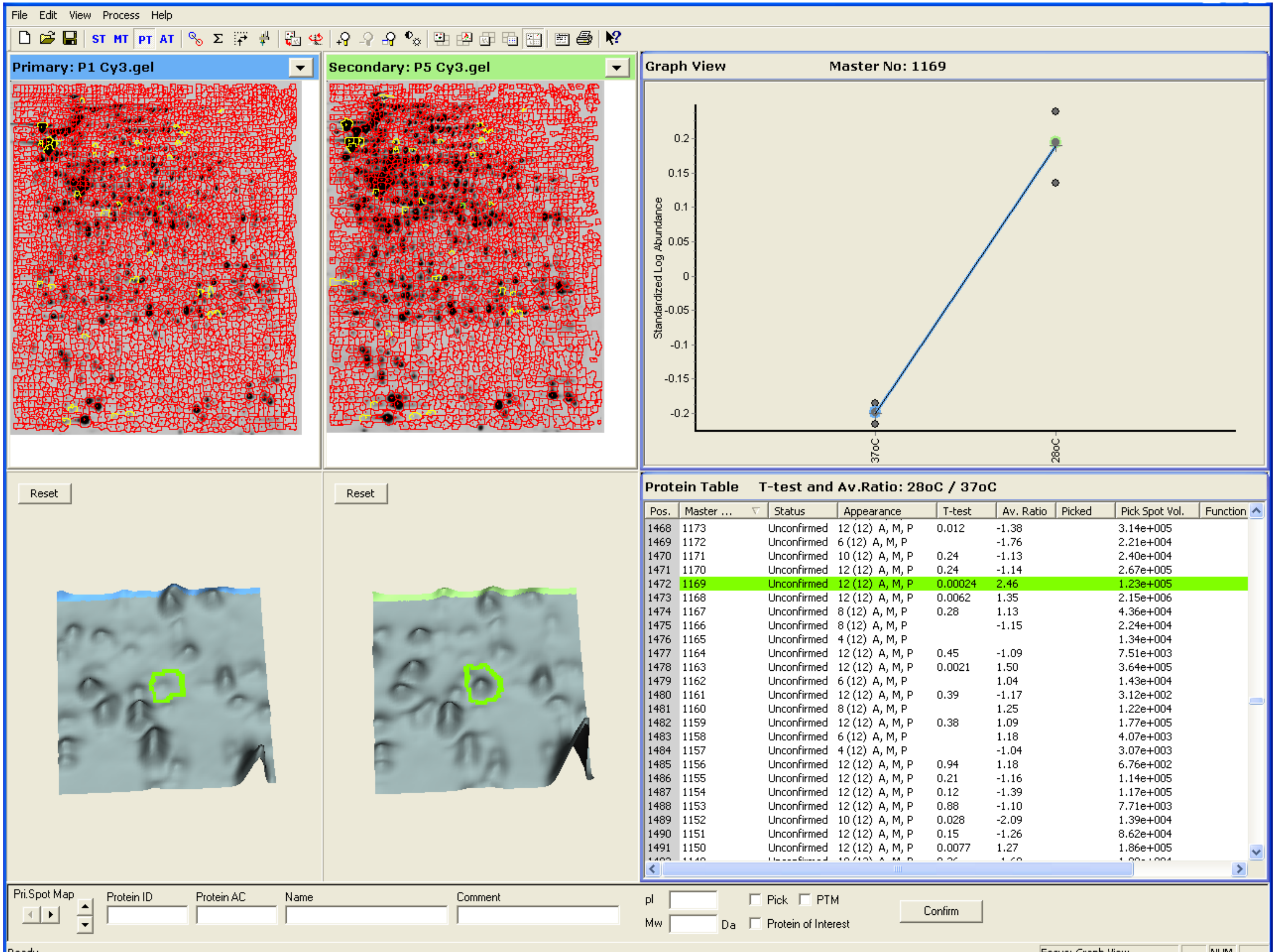

# MetY (PAU_01242)

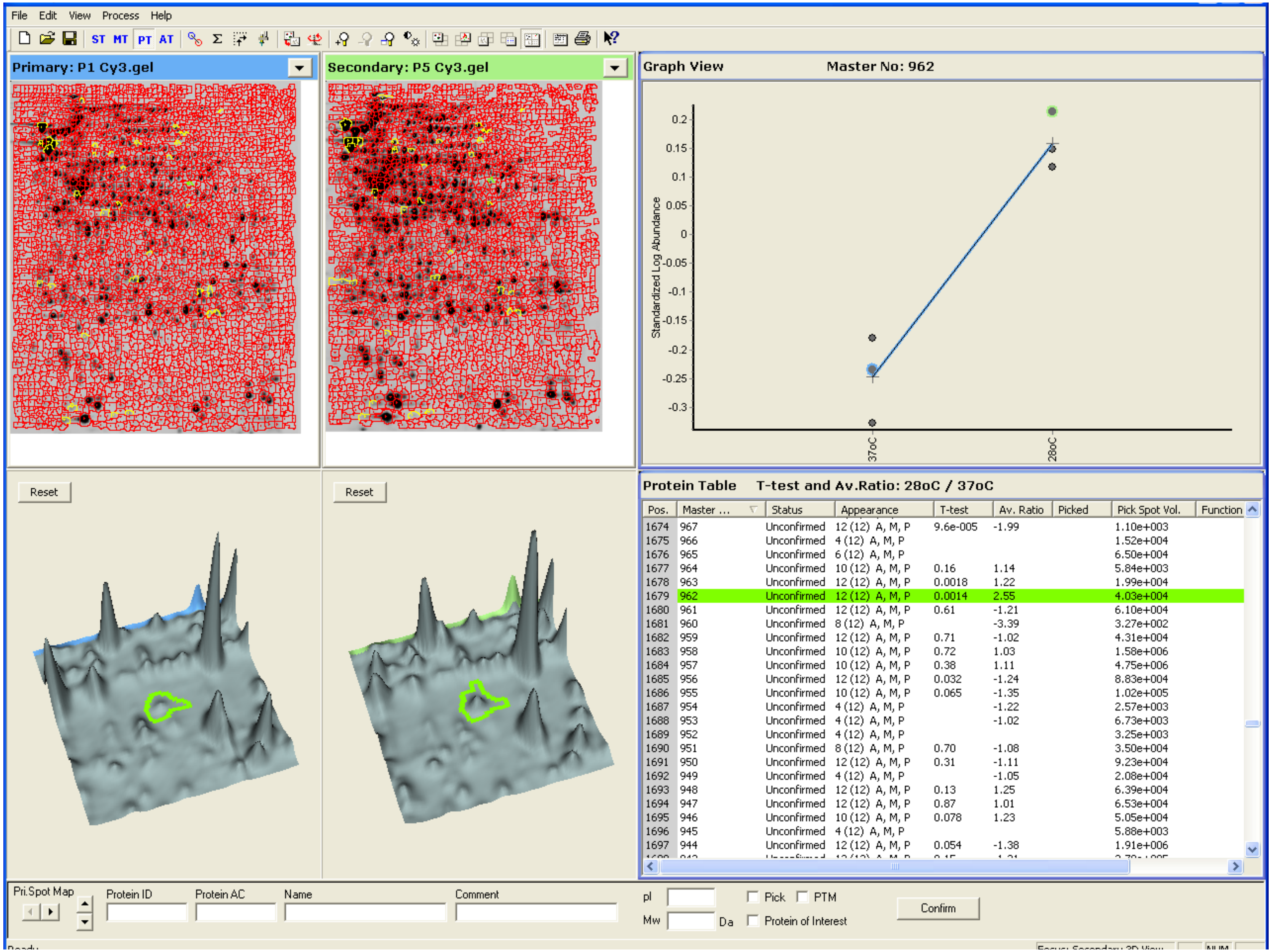

# ArnA (PAU_01877)

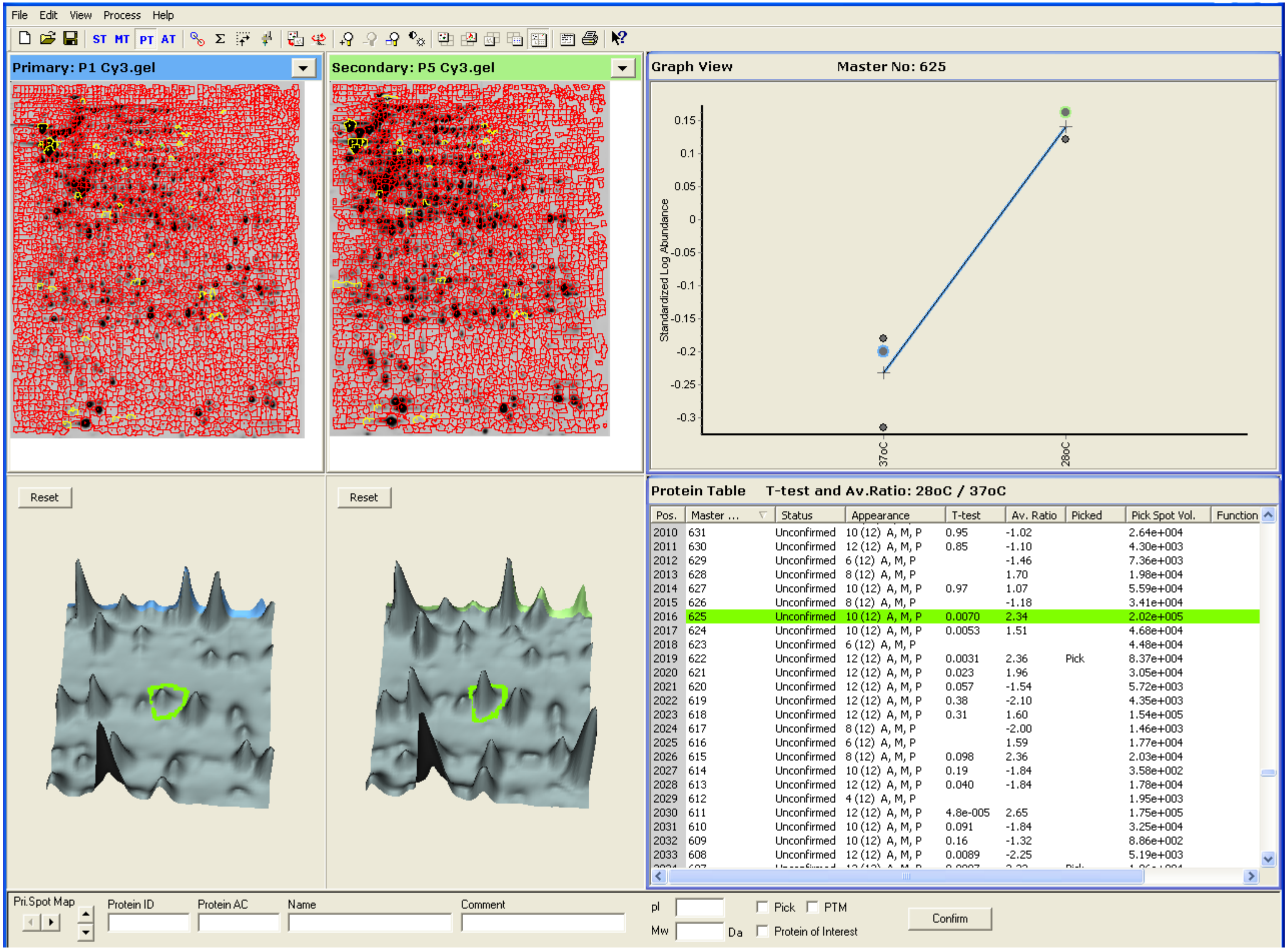

# Zwf (PAU_02443)

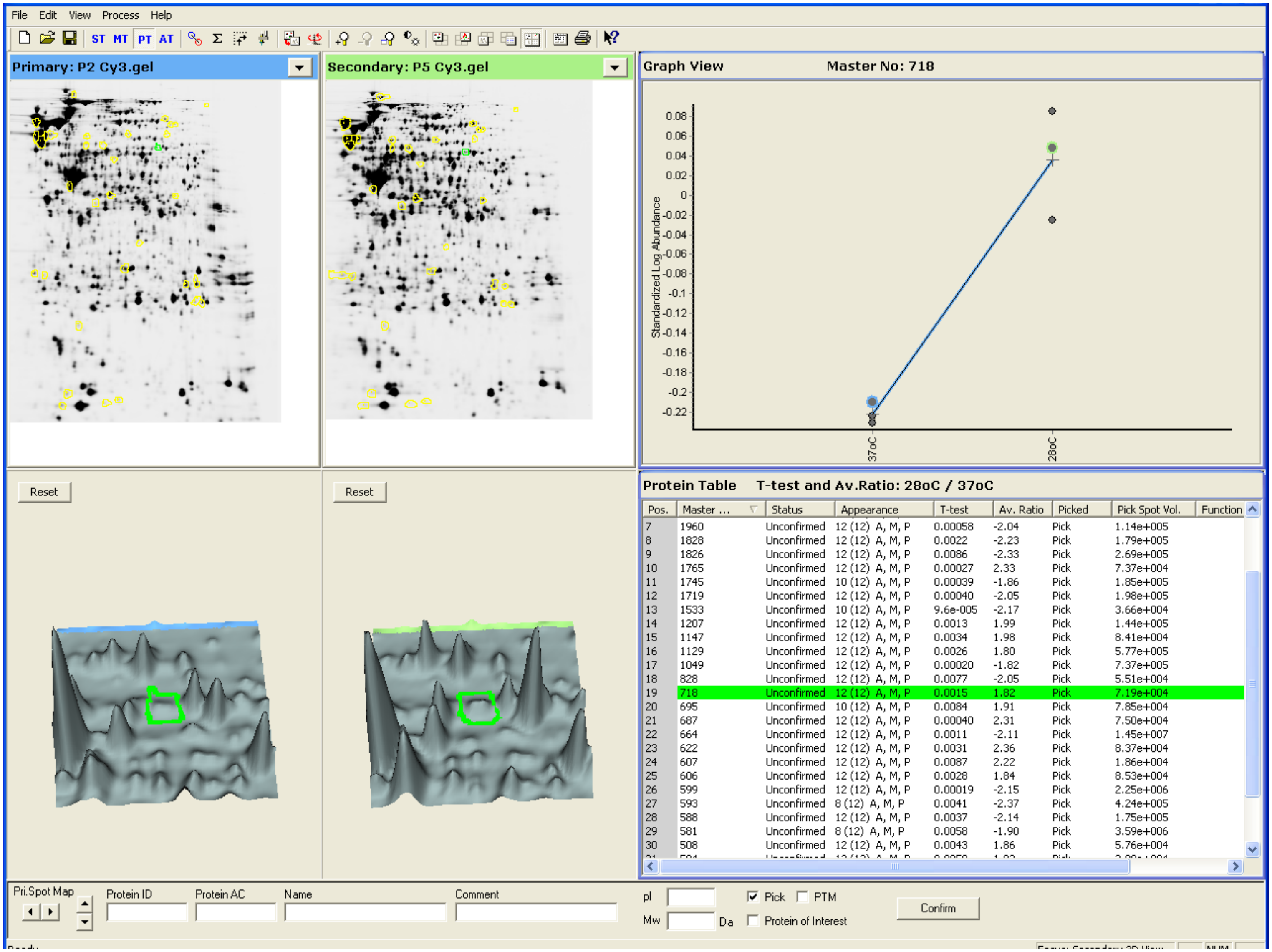

# PAU_01687

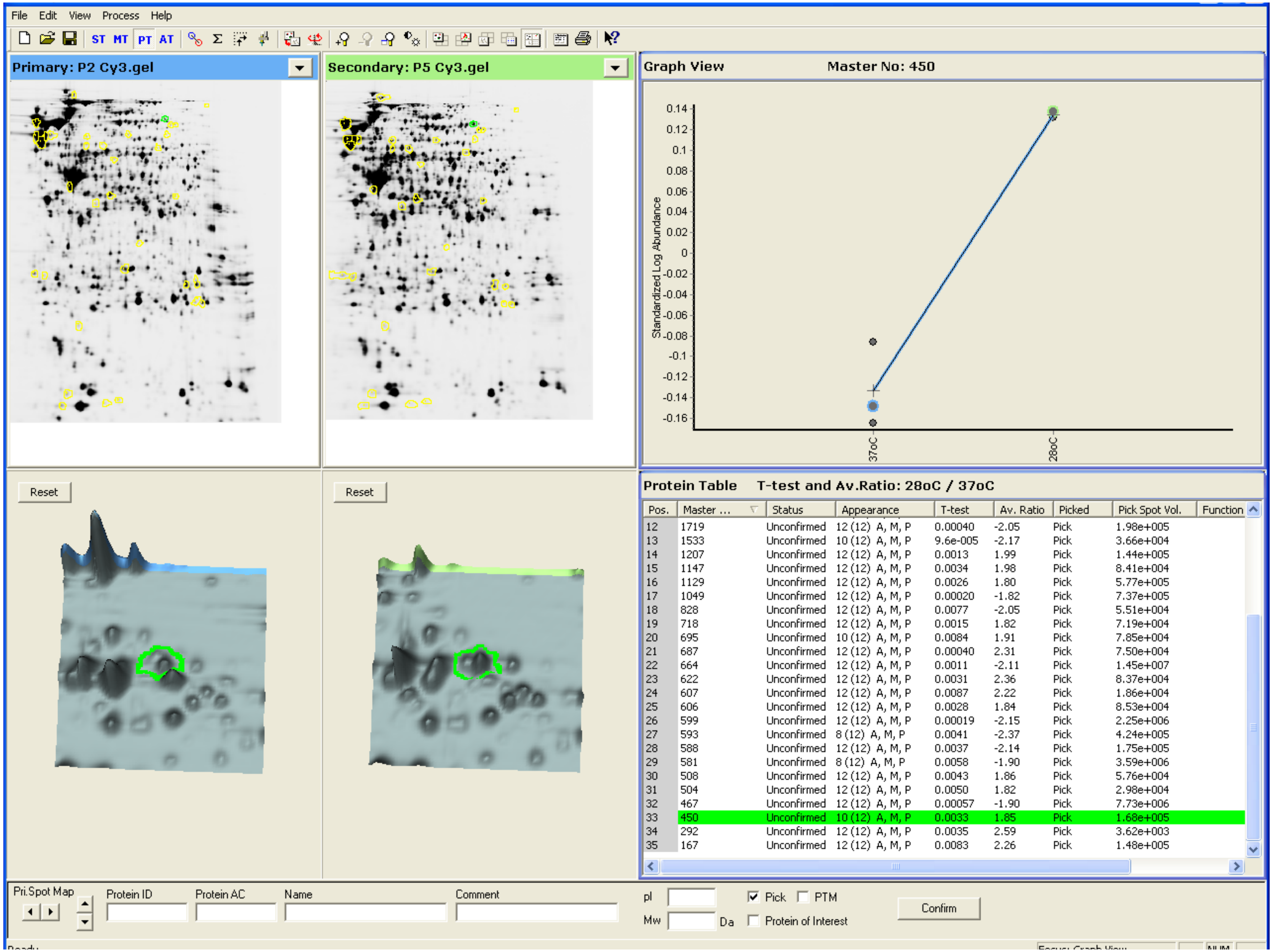

File Edit View Process Help
ST MT PT AT
Primary: P2 Cy3.gel
Secondary: P5 Cy3.gel
Graph View
Master No: 450
Standardized Log Abundance
0.14
0.12
0.1
0.08
0.06
0.04
0.02
0
-0.02
-0.04
-0.06
-0.08
-0.1
-0.12
-0.14
-0.16
37oC
28oC
Reset
Reset
Protein Table
T-test and Av.Ratio: 28oC / 37oC
Pos. Master ... Status Appearance T-test Av. Ratio Picked Pick Spot Vol. Function
12 1719 Unconfirmed 12 (12) A, M, P 0.00040 -2.05 Pick 1.98e+005
13 1533 Unconfirmed 10 (12) A, M, P 9.6e-005 -2.17 Pick 3.66e+004
14 1207 Unconfirmed 12 (12) A, M, P 0.0013 1.99 Pick 1.44e+005
15 1147 Unconfirmed 12 (12) A, M, P 0.0034 1.98 Pick 8.41e+004
16 1129 Unconfirmed 12 (12) A, M, P 0.0026 1.80 Pick 5.77e+005
17 1049 Unconfirmed 12 (12) A, M, P 0.00020 -1.82 Pick 7.37e+005
18 828 Unconfirmed 12 (12) A, M, P 0.0077 -2.05 Pick 5.51e+004
19 718 Unconfirmed 12 (12) A, M, P 0.0015 1.82 Pick 7.19e+004
20 695 Unconfirmed 10 (12) A, M, P 0.0084 1.91 Pick 7.85e+004
21 687 Unconfirmed 12 (12) A, M, P 0.00040 2.31 Pick 7.50e+004
22 664 Unconfirmed 12 (12) A, M, P 0.0011 -2.11 Pick 1.45e+007
23 622 Unconfirmed 12 (12) A, M, P 0.0031 2.36 Pick 8.37e+004
24 607 Unconfirmed 12 (12) A, M, P 0.0087 2.22 Pick 1.86e+004
25 606 Unconfirmed 12 (12) A, M, P 0.0028 1.84 Pick 8.53e+004
26 599 Unconfirmed 12 (12) A, M, P 0.00019 -2.15 Pick 2.25e+006
27 593 Unconfirmed 8 (12) A, M, P 0.0041 -2.37 Pick 4.24e+005
28 588 Unconfirmed 12 (12) A, M, P 0.0037 -2.14 Pick 1.75e+005
29 581 Unconfirmed 8 (12) A, M, P 0.0058 -1.90 Pick 3.59e+006
30 508 Unconfirmed 12 (12) A, M, P 0.0043 1.86 Pick 5.76e+004
31 504 Unconfirmed 12 (12) A, M, P 0.0050 1.82 Pick 2.98e+004
32 467 Unconfirmed 12 (12) A, M, P 0.00057 -1.90 Pick 7.73e+006
33 450 Unconfirmed 10 (12) A, M, P 0.0033 1.85 Pick 1.68e+005
34 292 Unconfirmed 12 (12) A, M, P 0.0035 2.59 Pick 3.62e+003
35 167 Unconfirmed 12 (12) A, M, P 0.0083 2.26 Pick 1.48e+005
Pri.Spot Map
Protein ID
Protein AC
Name
Comment
pI
Mw
Da
Pick
PTM
Protein of Interest
Confirm

# VgrG (PAU_00969)

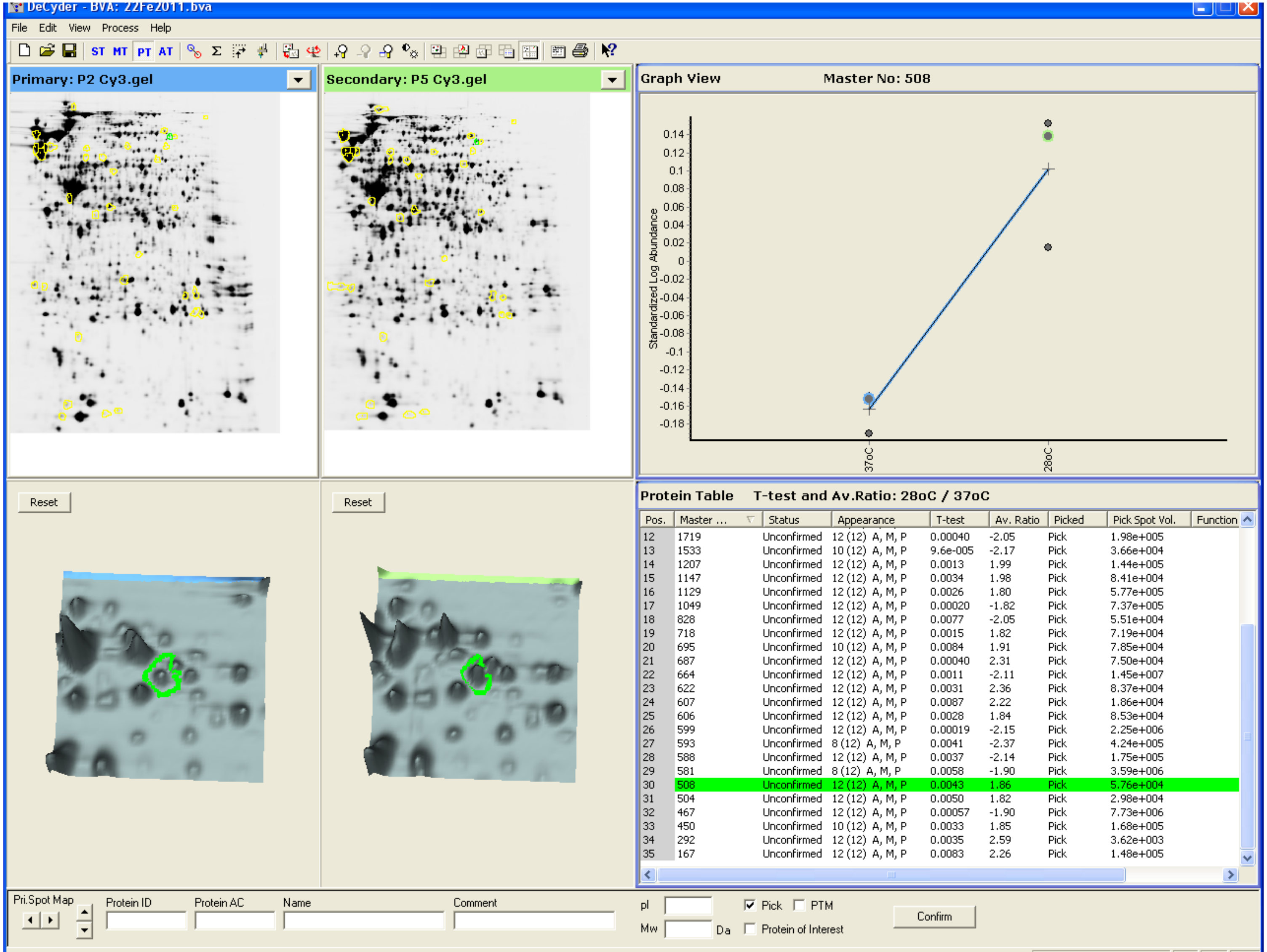

# GuaA (PAU_01827)

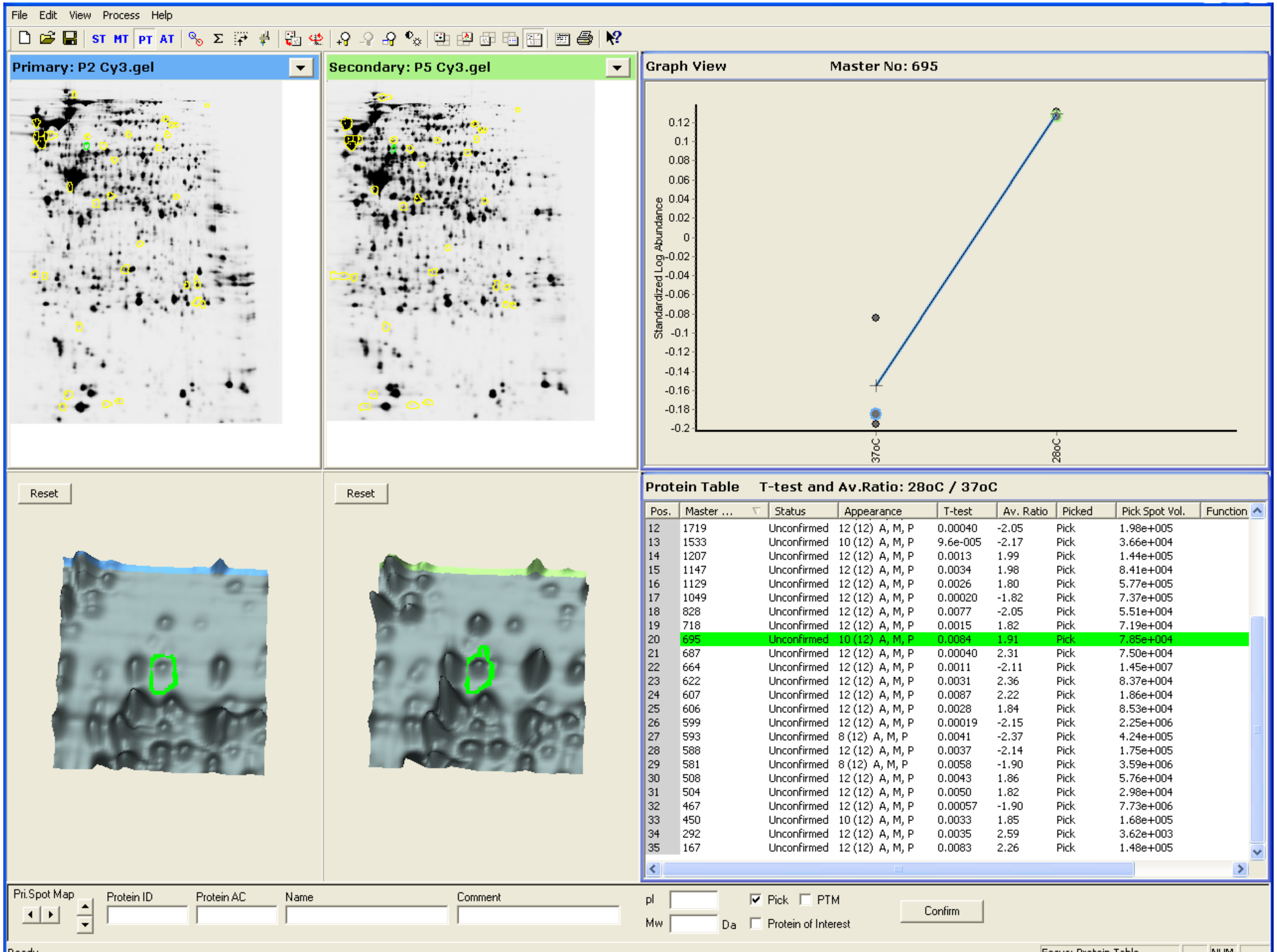

# Ggt (PAU_03499)

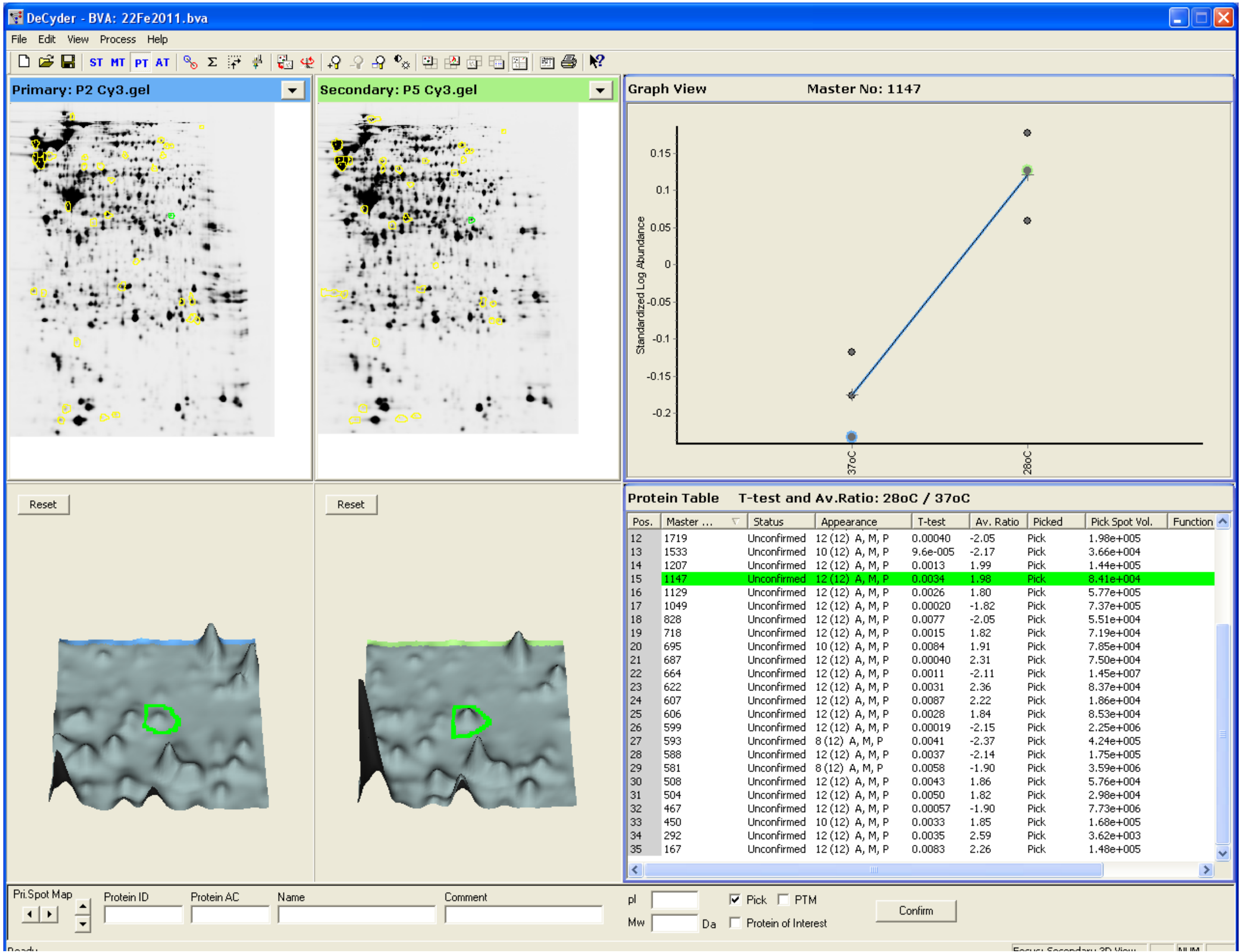


| Pos. | Master ... | Status | Appearance | T-test | Av. Ratio | Picked | Pick Spot Vol. | Function |
|---|---|---|---|---|---|---|---|---|
| 12 | 1719 | Unconfirmed | 12 (12) A, M, P | 0.00040 | -2.05 | Pick | 1.98e+005 | |
| 13 | 1533 | Unconfirmed | 10 (12) A, M, P | 9.6e-005 | -2.17 | Pick | 3.66e+004 | |
| 14 | 1207 | Unconfirmed | 12 (12) A, M, P | 0.0013 | 1.99 | Pick | 1.44e+005 | |
| 15 | 1147 | Unconfirmed | 12 (12) A, M, P | 0.0034 | 1.98 | Pick | 8.41e+004 | |
| 16 | 1129 | Unconfirmed | 12 (12) A, M, P | 0.0026 | 1.80 | Pick | 5.77e+005 | |
| 17 | 1049 | Unconfirmed | 12 (12) A, M, P | 0.00020 | -1.82 | Pick | 7.37e+005 | |
| 18 | 828 | Unconfirmed | 12 (12) A, M, P | 0.0077 | -2.05 | Pick | 5.51e+004 | |
| 19 | 718 | Unconfirmed | 12 (12) A, M, P | 0.0015 | 1.82 | Pick | 7.19e+004 | |
| 20 | 695 | Unconfirmed | 10 (12) A, M, P | 0.0084 | 1.91 | Pick | 7.85e+004 | |
| 21 | 687 | Unconfirmed | 12 (12) A, M, P | 0.00040 | 2.31 | Pick | 7.50e+004 | |
| 22 | 664 | Unconfirmed | 12 (12) A, M, P | 0.0011 | -2.11 | Pick | 1.45e+007 | |
| 23 | 622 | Unconfirmed | 12 (12) A, M, P | 0.0031 | 2.36 | Pick | 8.37e+004 | |
| 24 | 607 | Unconfirmed | 12 (12) A, M, P | 0.0087 | 2.22 | Pick | 1.86e+004 | |
| 25 | 606 | Unconfirmed | 12 (12) A, M, P | 0.0028 | 1.84 | Pick | 8.53e+004 | |
| 26 | 599 | Unconfirmed | 12 (12) A, M, P | 0.00019 | -2.15 | Pick | 2.25e+006 | |
| 27 | 593 | Unconfirmed | 8 (12) A, M, P | 0.0041 | -2.37 | Pick | 4.24e+005 | |
| 28 | 588 | Unconfirmed | 12 (12) A, M, P | 0.0037 | -2.14 | Pick | 1.75e+005 | |
| 29 | 581 | Unconfirmed | 8 (12) A, M, P | 0.0058 | -1.90 | Pick | 3.59e+006 | |
| 30 | 508 | Unconfirmed | 12 (12) A, M, P | 0.0043 | 1.86 | Pick | 5.76e+004 | |
| 31 | 504 | Unconfirmed | 12 (12) A, M, P | 0.0050 | 1.82 | Pick | 2.98e+004 | |
| 32 | 467 | Unconfirmed | 12 (12) A, M, P | 0.00057 | -1.90 | Pick | 7.73e+006 | |
| 33 | 450 | Unconfirmed | 10 (12) A, M, P | 0.0033 | 1.85 | Pick | 1.68e+005 | |
| 34 | 292 | Unconfirmed | 12 (12) A, M, P | 0.0035 | 2.59 | Pick | 3.62e+003 | |
| 35 | 167 | Unconfirmed | 12 (12) A, M, P | 0.0083 | 2.26 | Pick | 1.48e+005 | |

# PurL (PAU_01324)

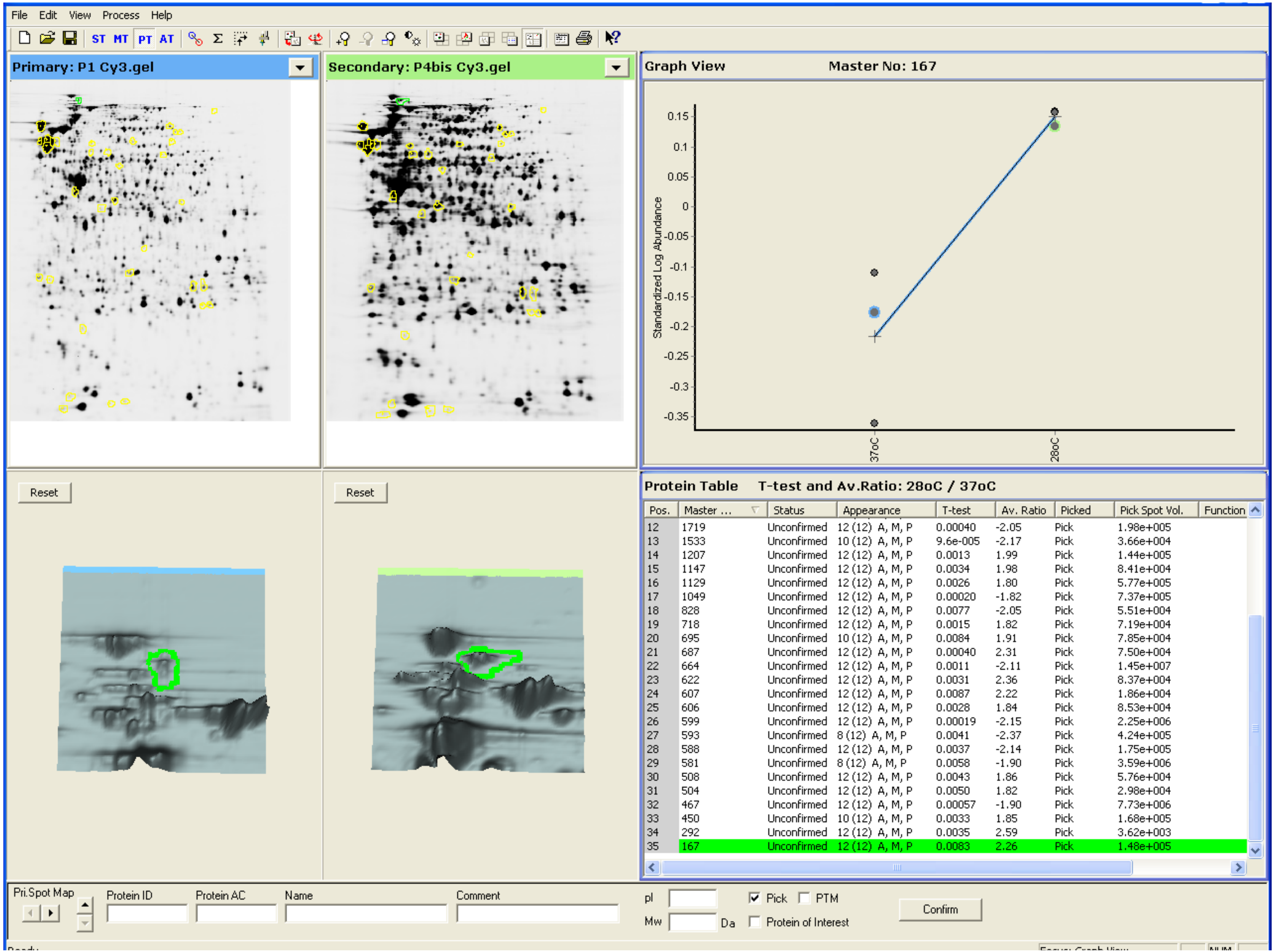

# SfcA (PAU_02896)

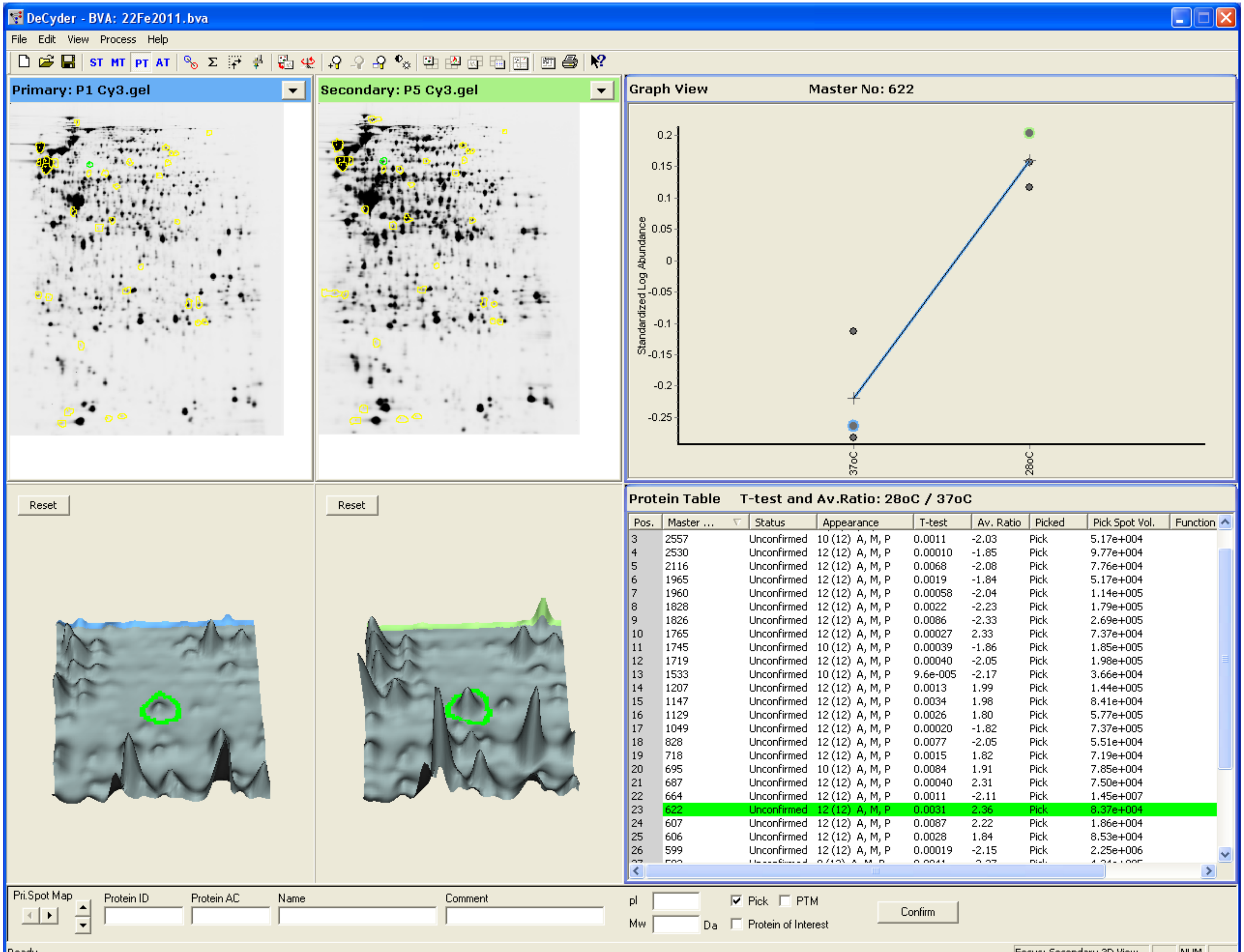

# GroEL (PAU_03756)

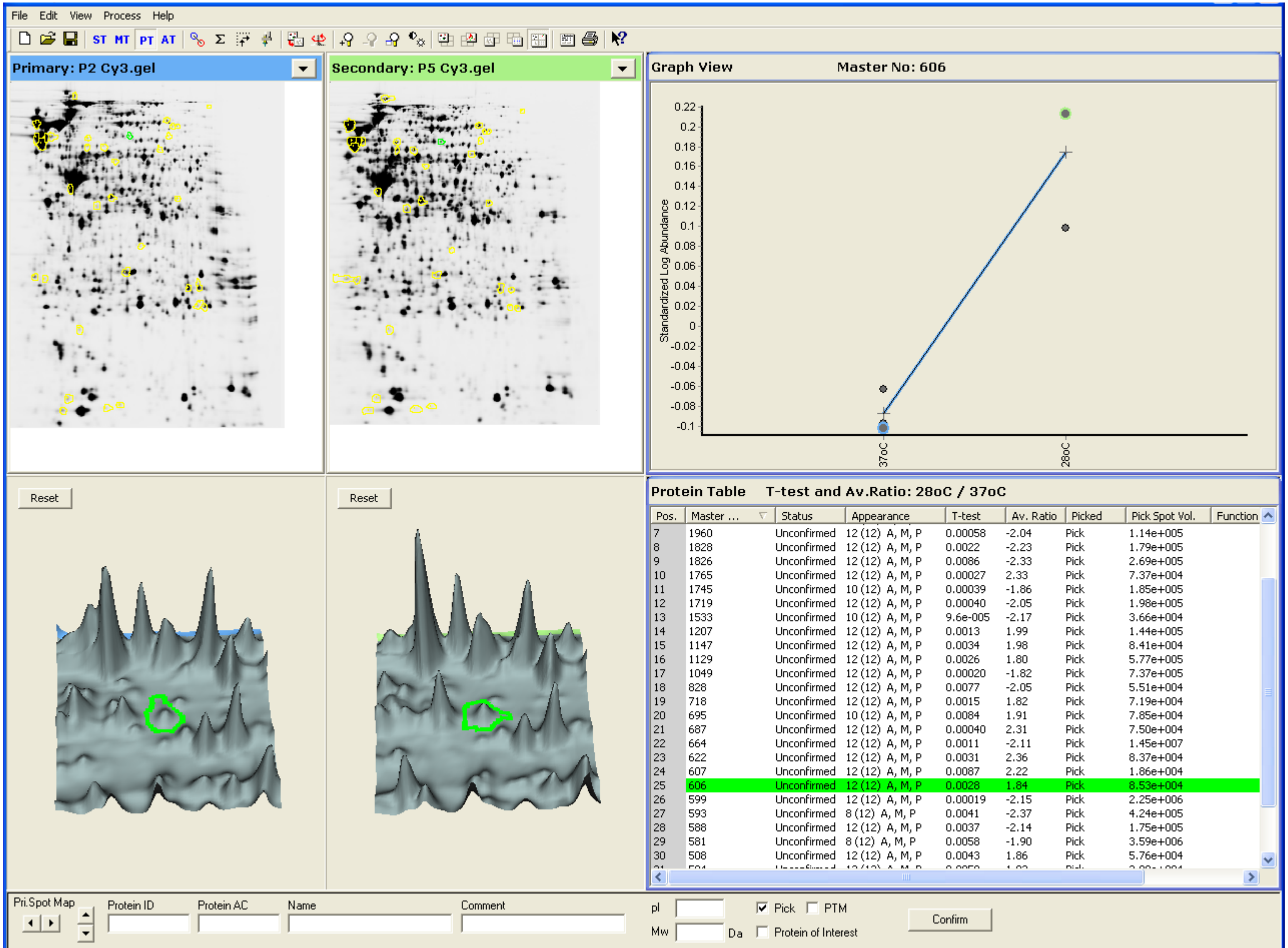

# DnaK (PAU_00453)

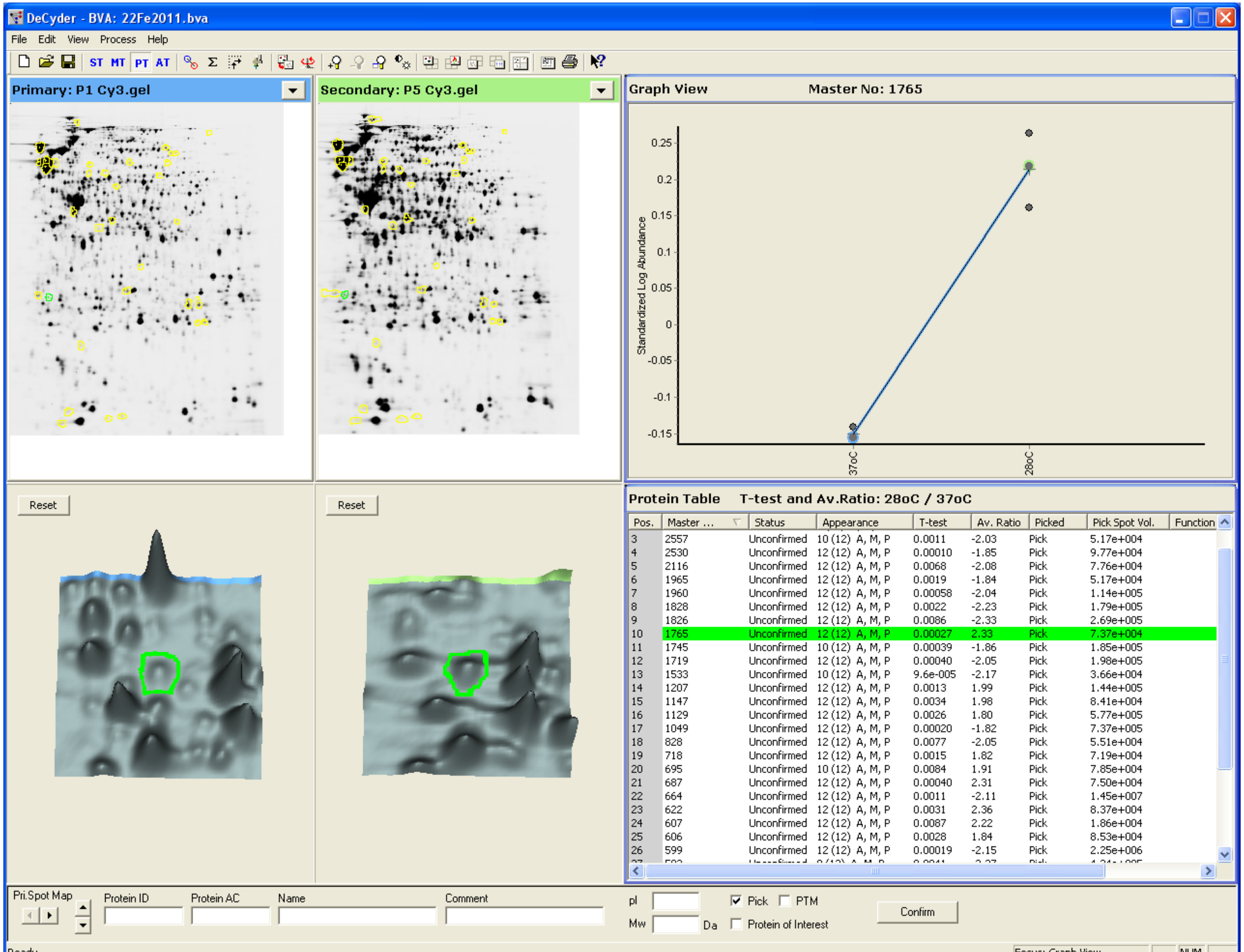

# AspC (PAU_02766)

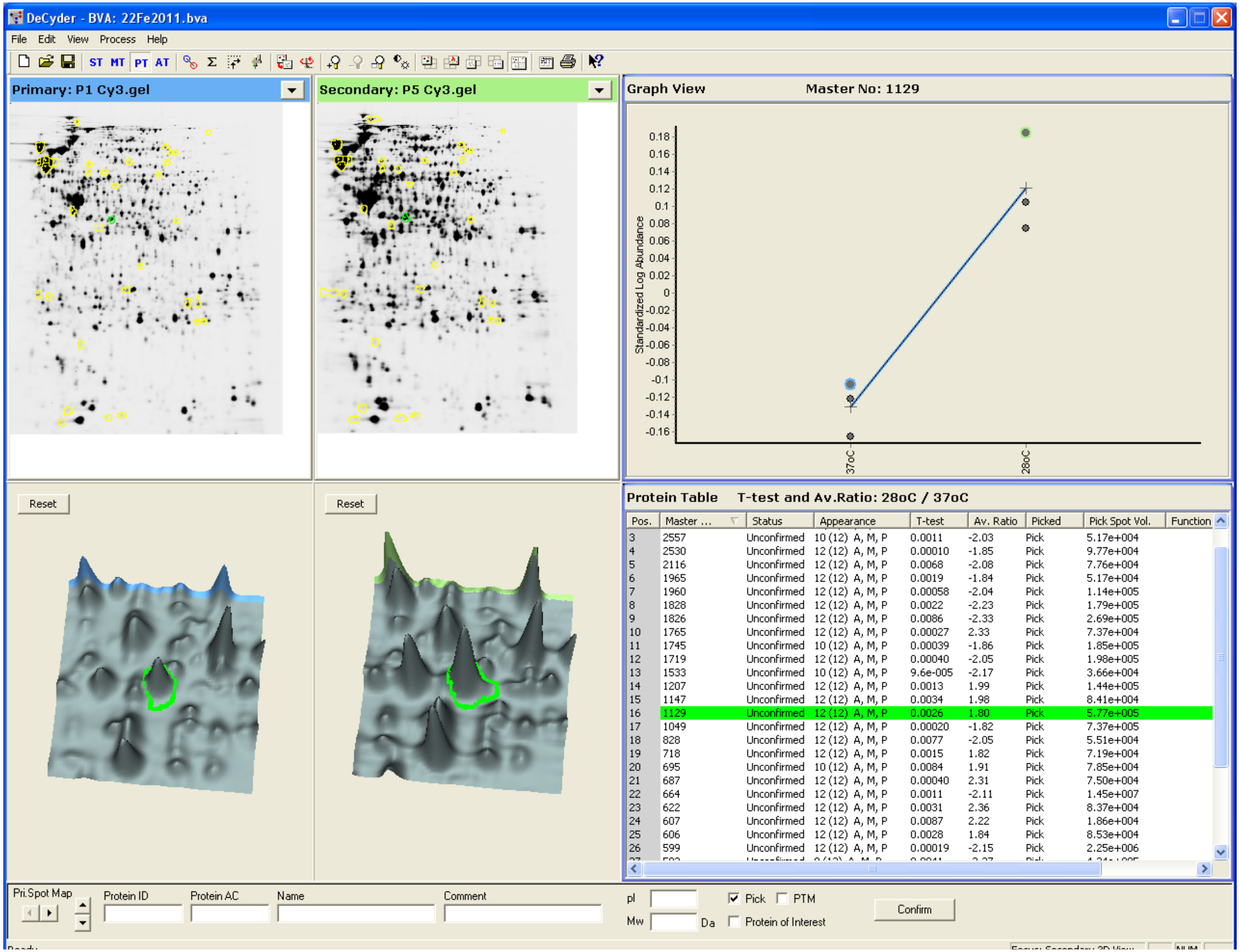

Supplement: S5 Data — (PDF) [file pone.0144937.s005.pdf]
