# Supplementary figures and images for: From Insect to Man: Photorhabdus Sheds Light on the Emergence of Human Pathogenicity
Source: PLoS One. 2015 Dec 17;10(12):e0144937. doi: 10.1371/journal.pone.0144937 (PMC4683029; doi:10.1371/journal.pone.0144937)

A

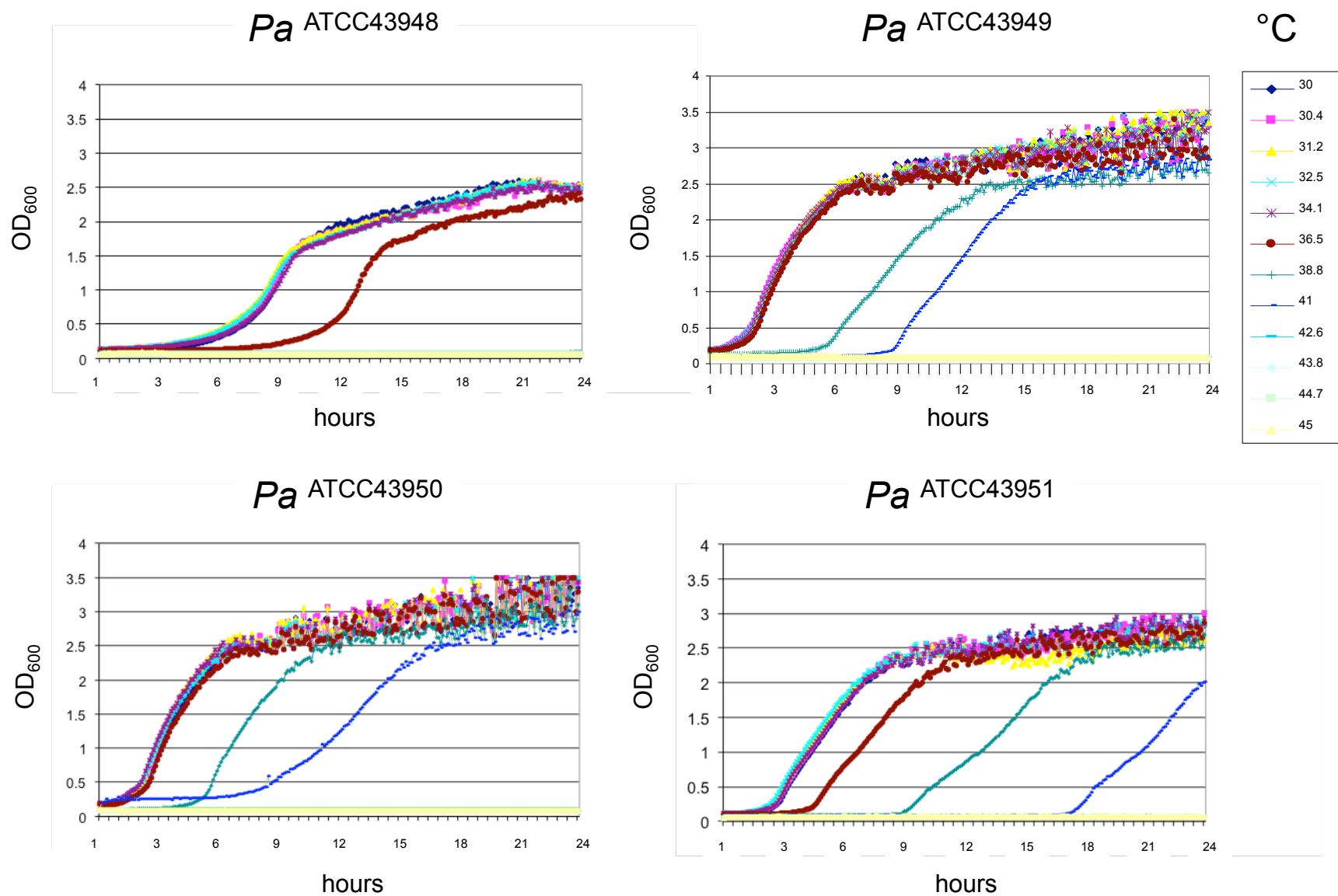

B

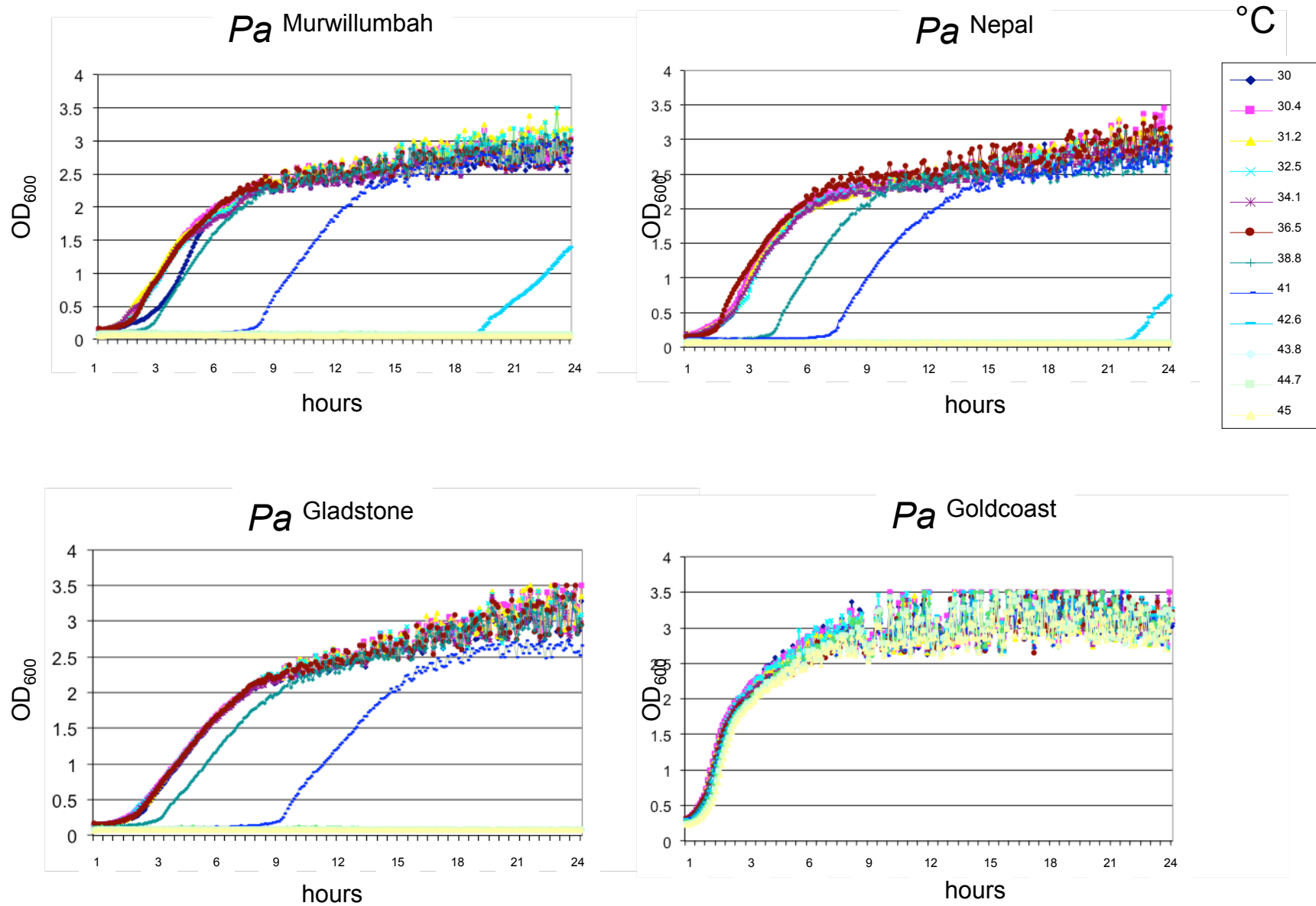

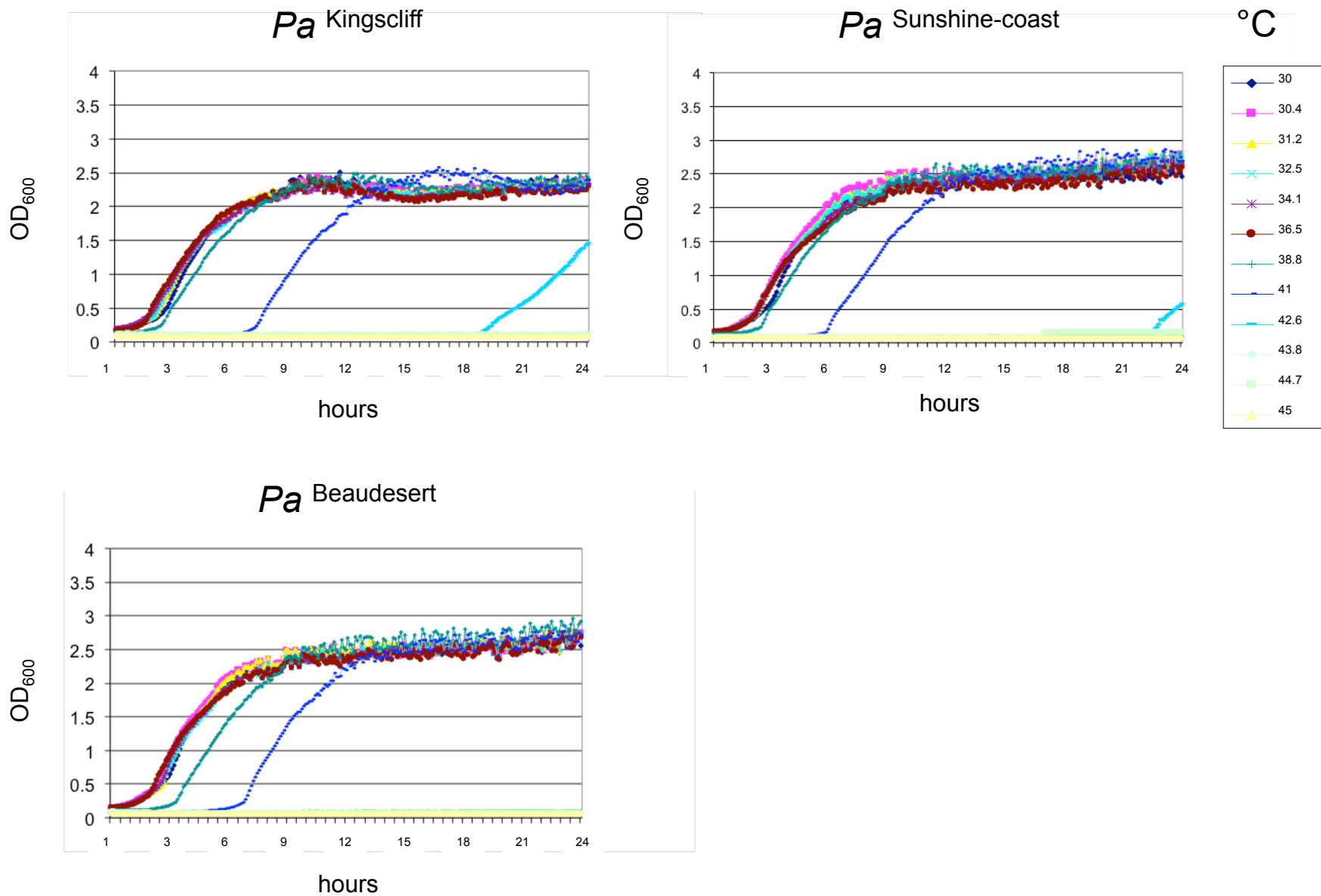

C

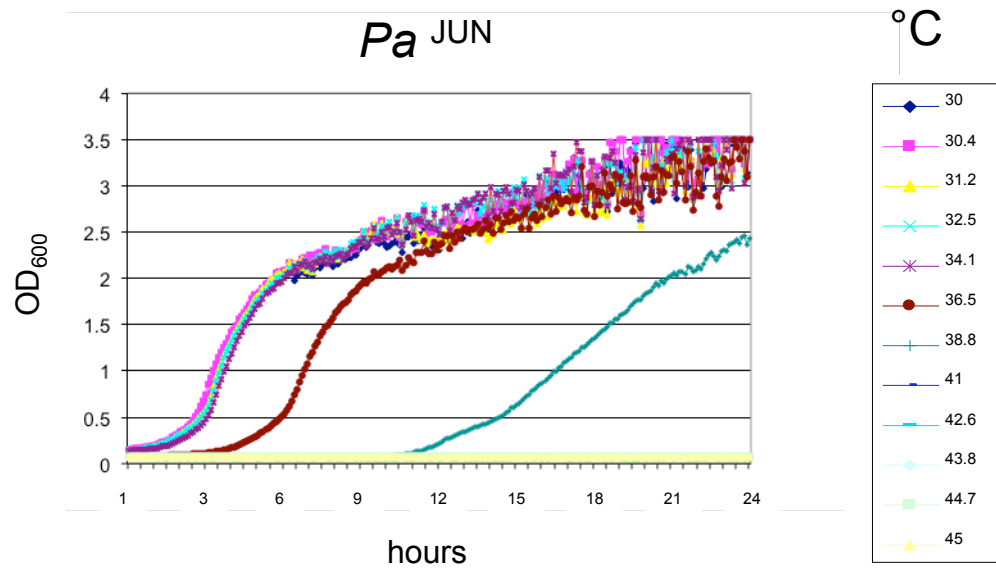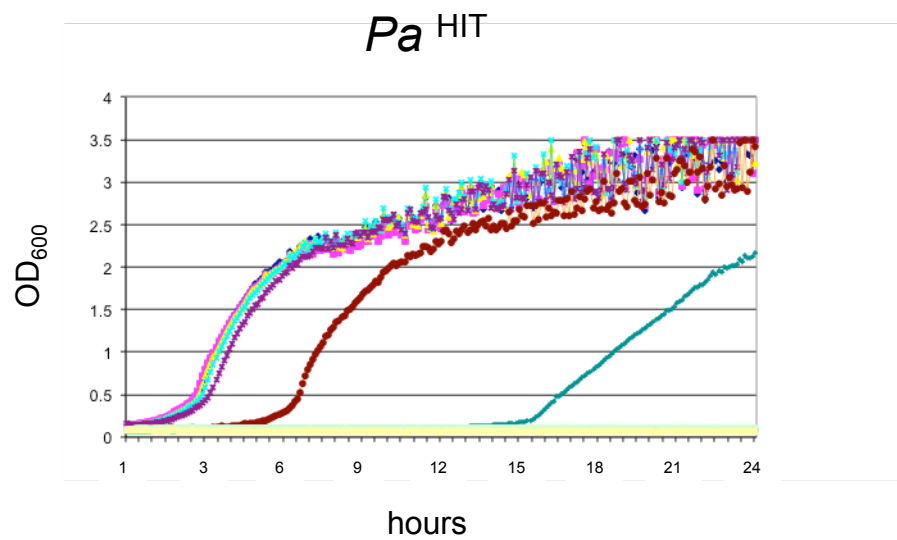

D

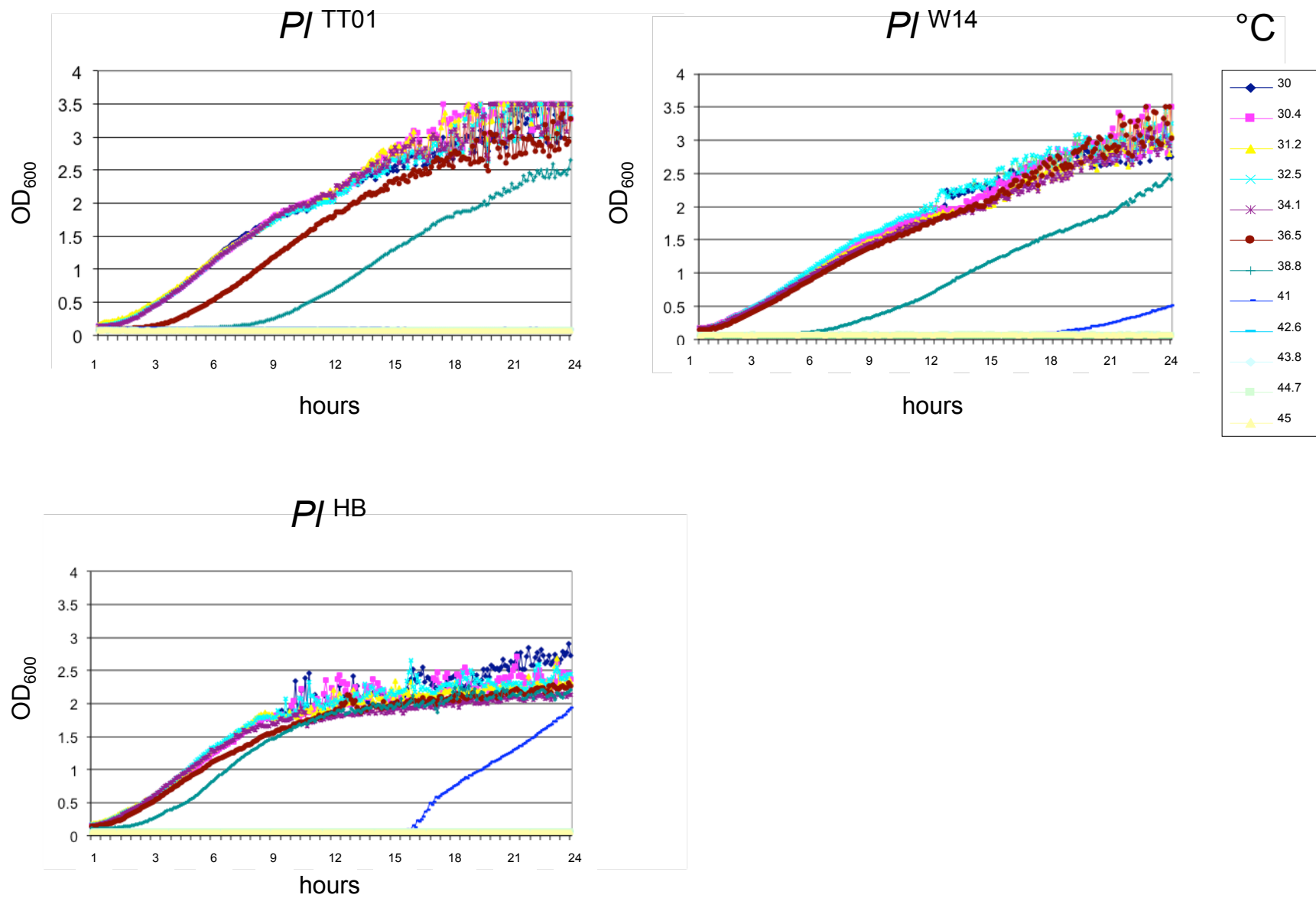

Supplement: S1 Fig — The growth dynamics of strains resuming aerated growth at 28°C in LB medium after a 4 h static exposure to a range of temperatures. Strains tested include clinical P. asymbiotica isolates from (A) USA, (B) Australia and Nepal, (C) non-clinical European P. asymbiotica isolates and (D) representative P. luminescens strains. Note there is a large variation in times taken for strains to exit lag-phase into exponential growth, but growth rates are comparable once exponential growth begins. The longer lag-phase observed at the threshold between permissive and non-permissive temperatures is therefore likely due to a proportion of the population dying due to temperature stress. (PDF) [file pone.0144937.s006.pdf]

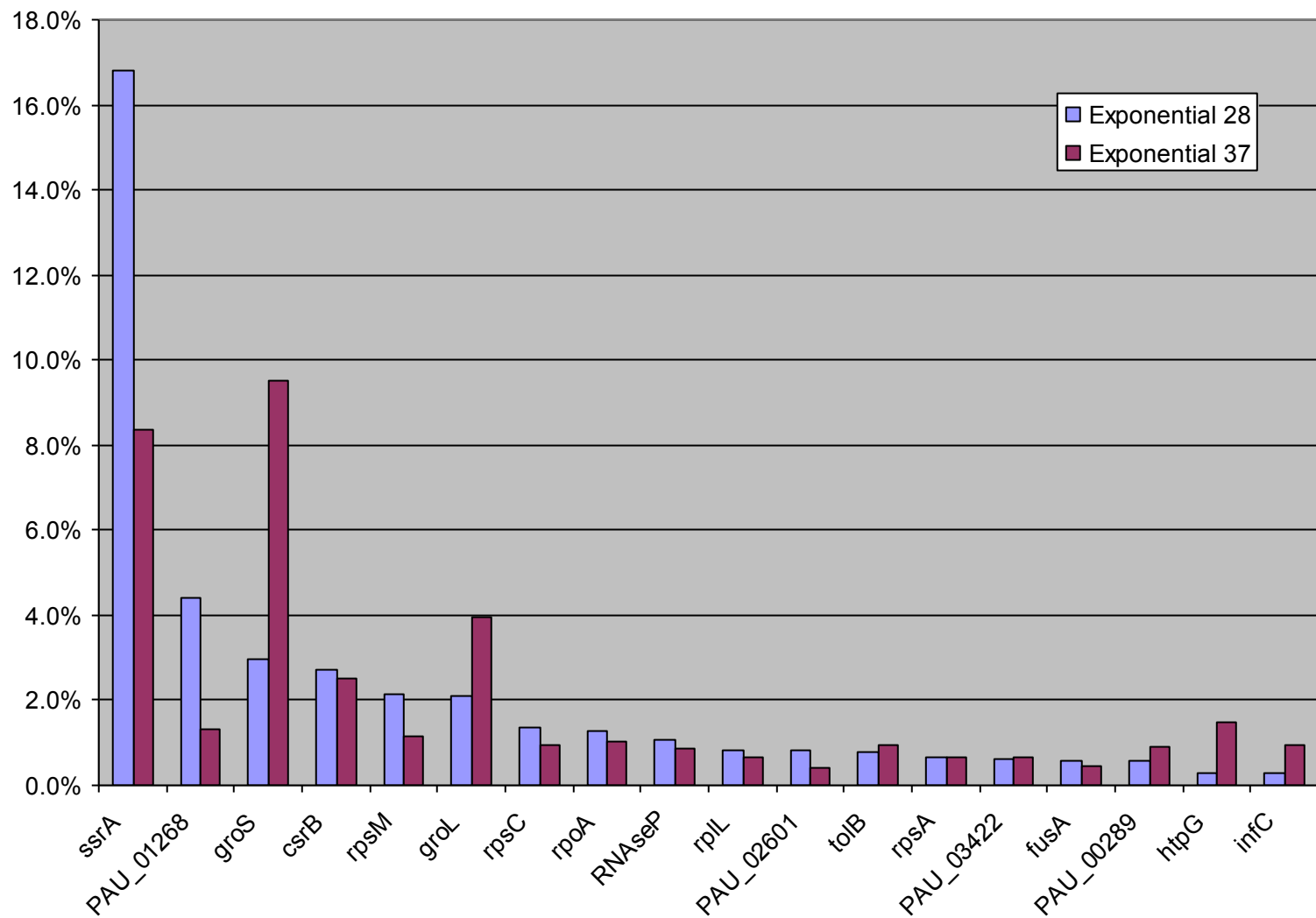

Supplement: S2 Fig — The graph shows the mapped base mean values as a percentage of total reads for various gene transcripts at the two temperatures. It should be noted that the majority of sRNAs are not annotated on the Pa ATCC43949 genome so would have been excluded from the DESEQ analysis of the RNA-seq data. (PDF) [file pone.0144937.s007.pdf]

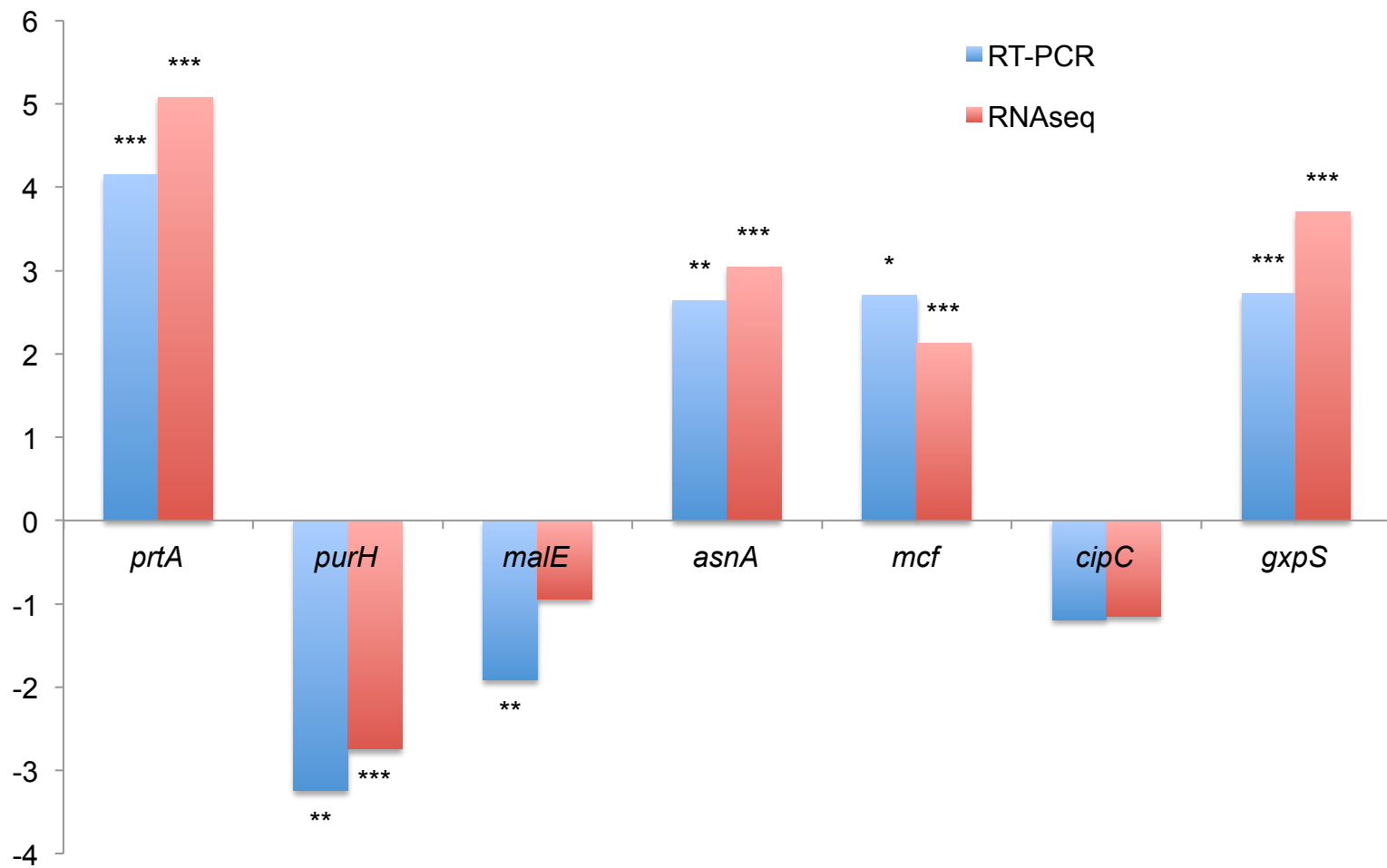

Supplement: S3 Fig — RNA-seq log2fold changes (y-axis) were compared to those measured by qRT-PCR. The symbols: [*], [**], [***] refers to P-value of <0.05, <0.01, <0.001 calculated using REST 2009 and DESeq for RT-PCR and RNA-seq respectively. RNA was extracted from cultures growing at exponential phase for 4 h in LB broth. Target genes were chosen to represent a range of classes and transcription levels. Primer sequences may be seen in S13 Table. (PDF) [file pone.0144937.s008.pdf]

A

28°C

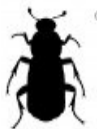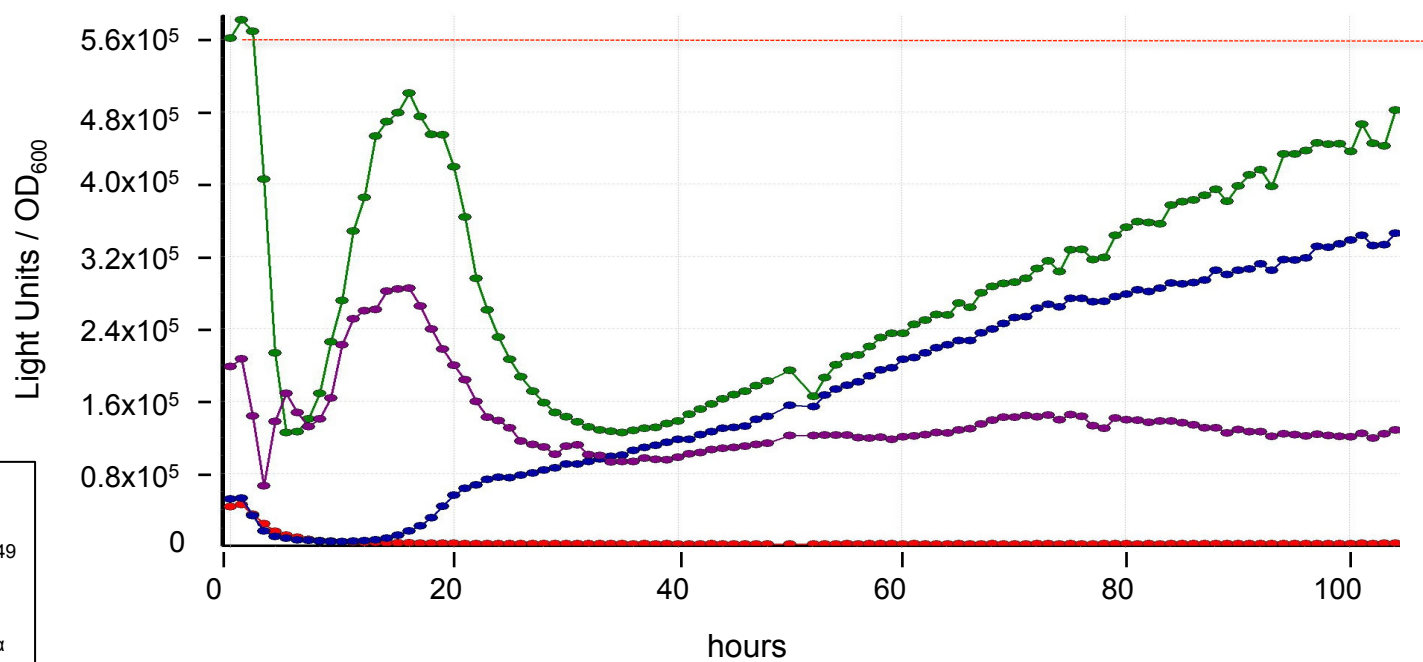

37°C

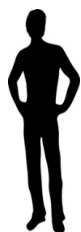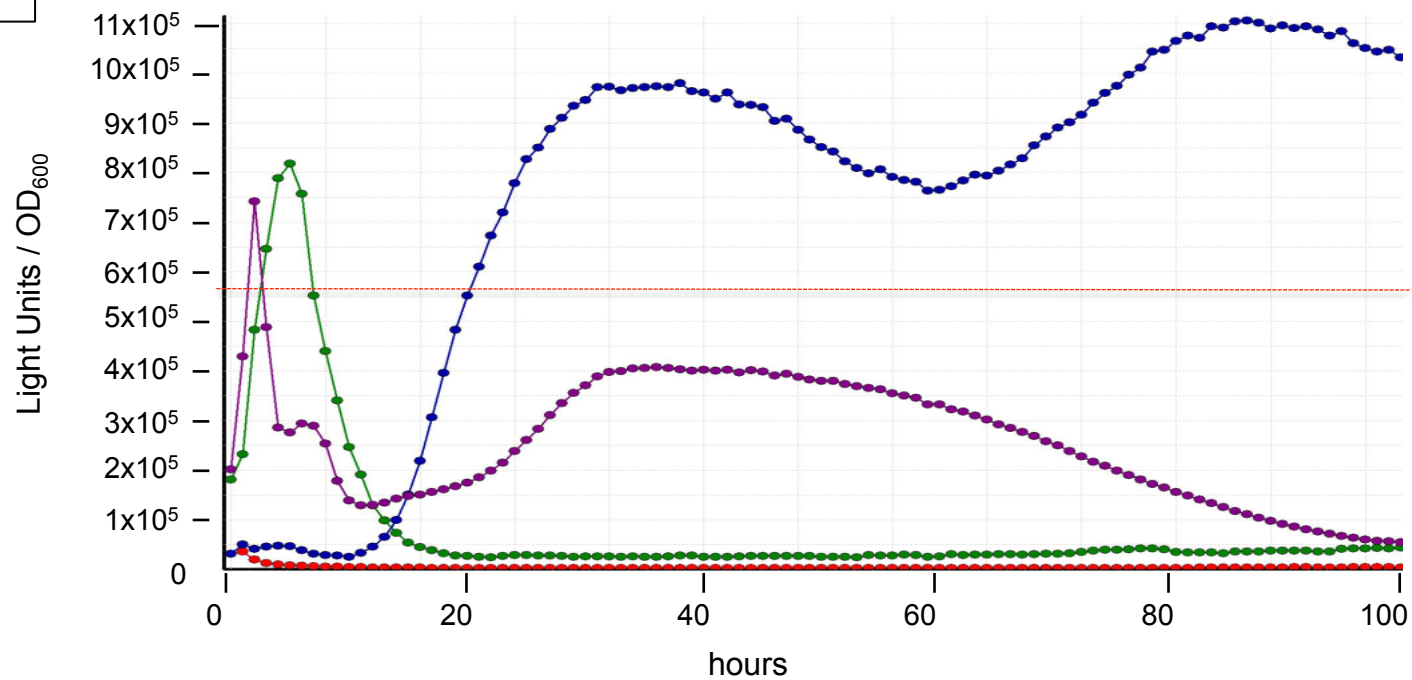

Supplement: S4 Fig — (A) Bioluminescence of Pa ATCC43949 (purple), Pa Kingscliff (blue), Pl TT01 (green) and E. coli DH5α negative control (red) growing at 28°C (top) and 37°C (bottom) in LB broth with aeration. Graphs show the relative mean light emission per cell (n = 3). All strains showed normal growth curves at 28°C and 37°C except Pl TT01 which only reached OD600 of 0.2 by 10 h at 37°C before declining. Red dotted line shows the equivalent emission level between the two graphs for comparison. (PDF) [file pone.0144937.s009.pdf]

28°C

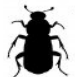

rep1

rep2

rep3

37°C

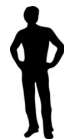

rep1

rep2

rep3

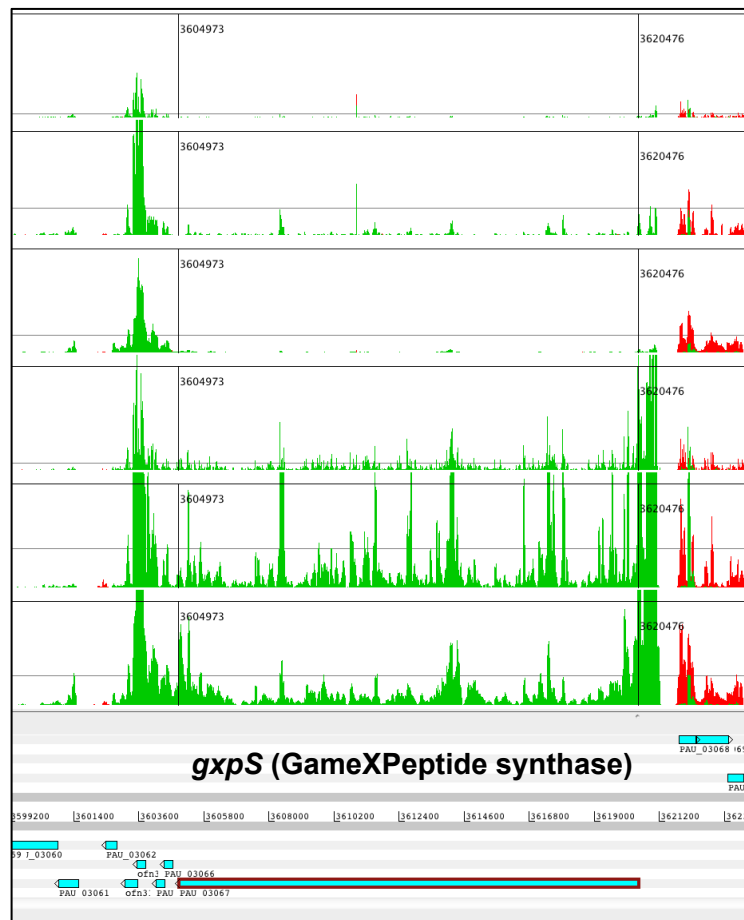

Supplement: S6 Fig — Increased transcription of this NRPS gene correlates with detection of increased levels of the final peptide in the supernatant. (PDF) [file pone.0144937.s011.pdf]

28°C

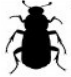

37°C

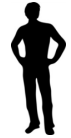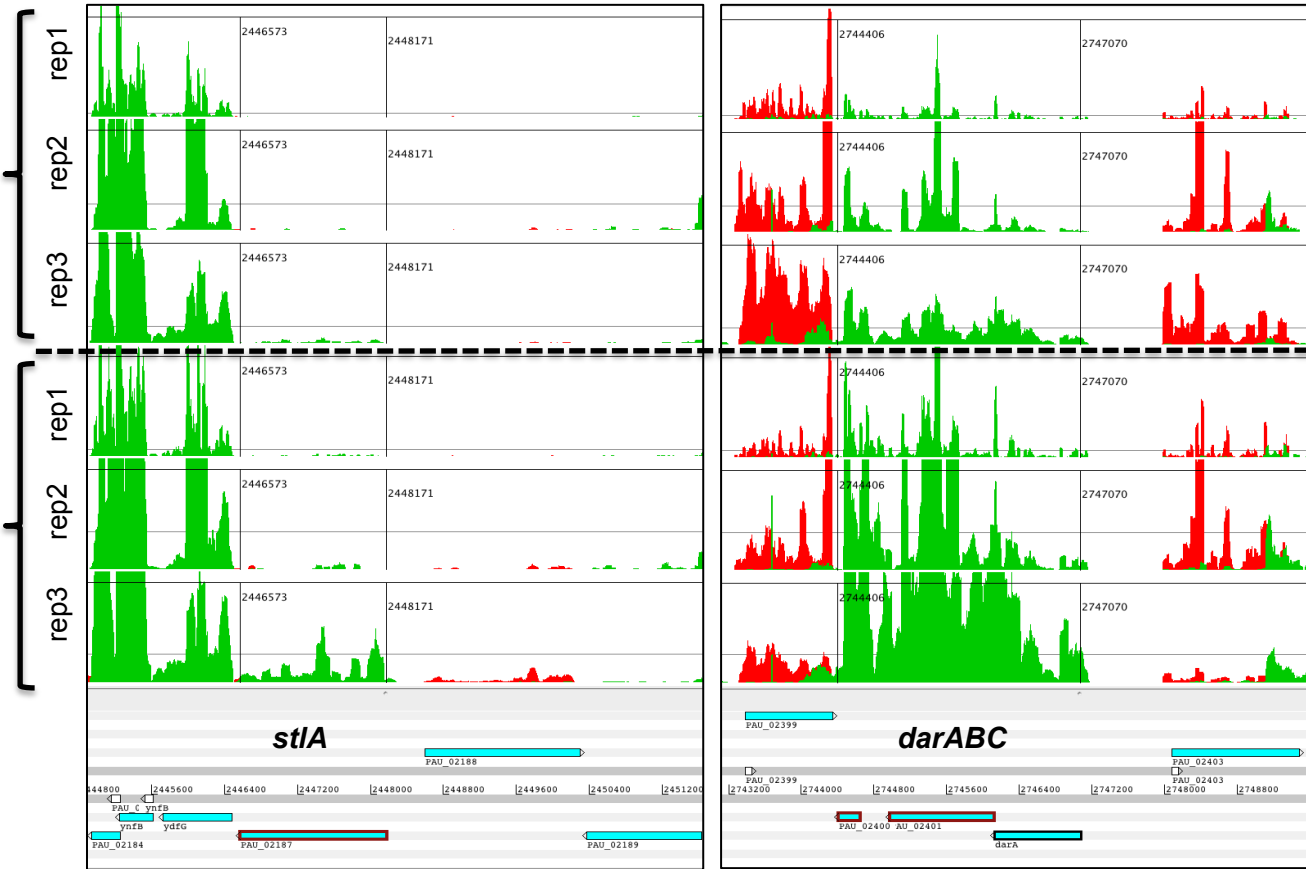

B

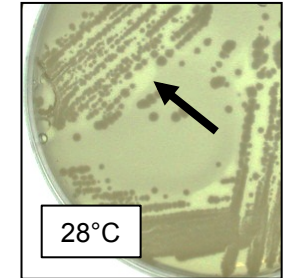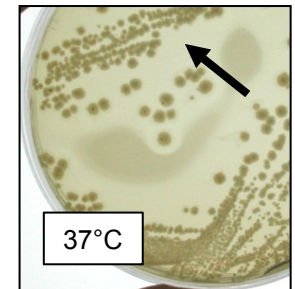

Staph. aureus  
MRSA cdc16  
overlay

Supplement: S7 Fig — (A) Artemis views of the RNA-seq reads of the three replicates mapped onto the Pa ATCC43949 operons responsible for IPS synthesis. Note stlA shows very little transcription during exponential growth although it is induced more highly at stationary phase (data not shown). Conversely the darABC operon transcription is inducted at 37°C during exponential growth. (B) Staphylococcus aureus MRSA (strain cdc16) overlaid onto Pa ATCC43949 grown on LB agar plates at 28°C and 37°C showing increased antibiotic production at 37°C. Arrows indicate zones of growth inhibition of the MRSA which is almost complete on the 37°C plate. (PDF) [file pone.0144937.s012.pdf]

A

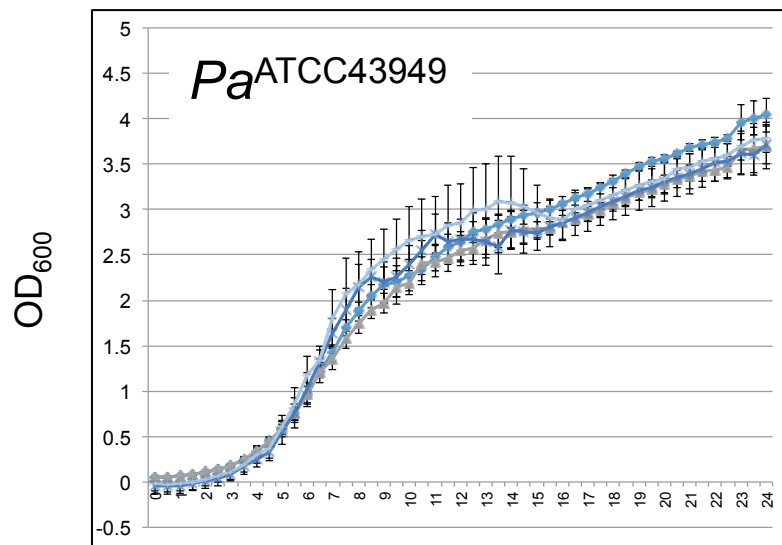

Time (h)

B

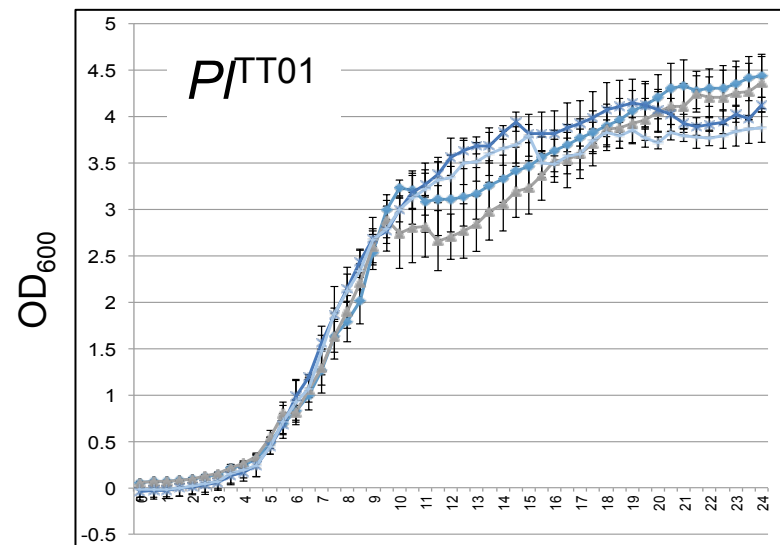

Time (h)

C

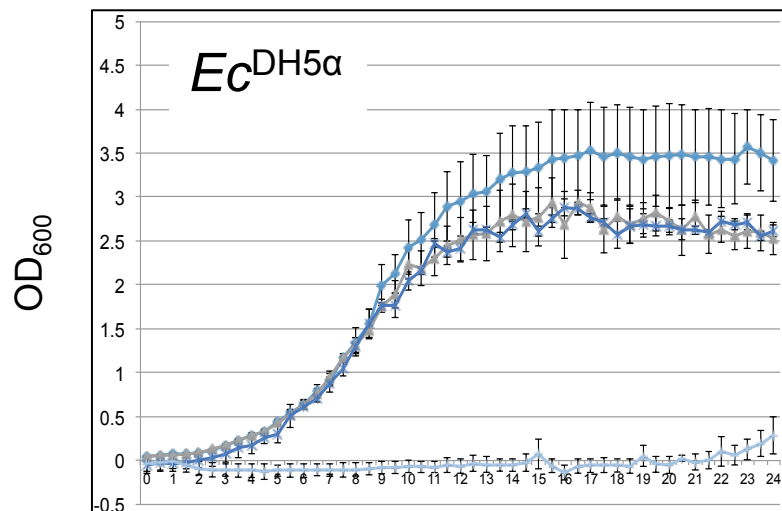

Time (h)

Key

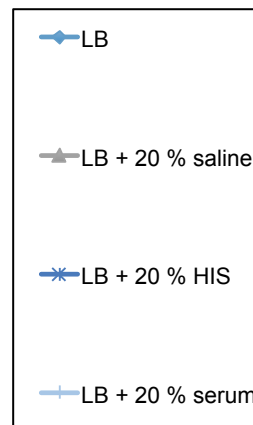

Supplement: S8 Fig — Growth of (A) Pa ATCC43949 and (B) Pl TT01 and (C) E. coli, Ec DH5α, at 28°C with aeration in LB and LB supplemented with 20% (v/v) human serum (type AB), Heat Inactivated human AB serum (HIS) and 0.9% saline. Results shown are the average of three independent experiments +/- standard error bars. (PDF) [file pone.0144937.s013.pdf]

A

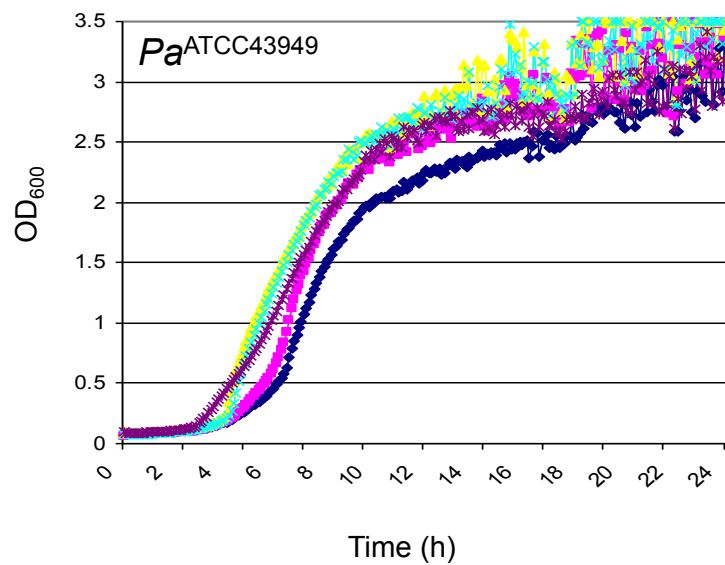

B

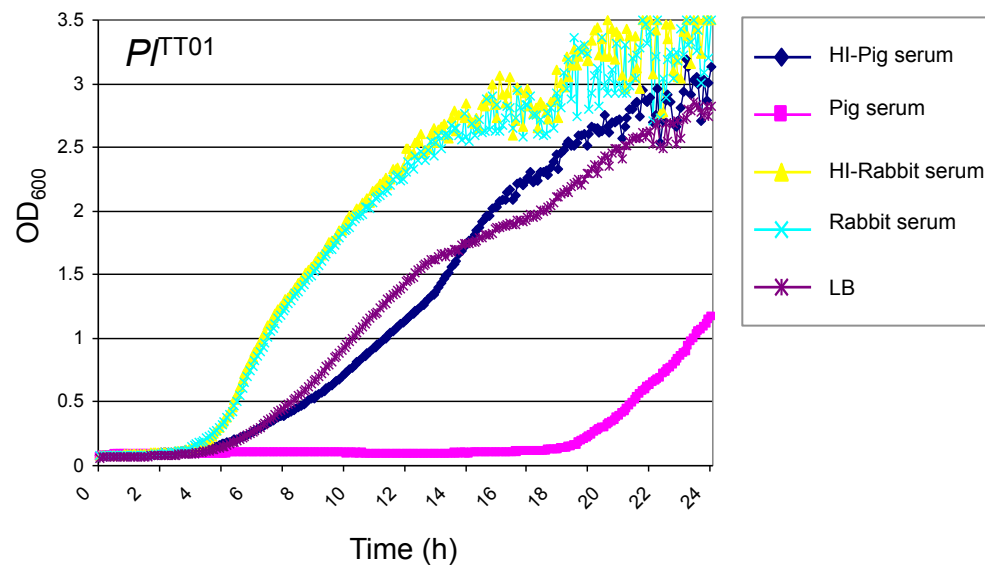

Supplement: S9 Fig — Growth of (A) Pa ATCC43949 and (B) Pl TT01 at 28°C with aeration in LB supplemented with 10% (v/v) serum from pigs and rabbits. Heat inactivated (HI) serum was also included. Note Pa ATCC43949 is resistant to both types while Pl TT01 shows complete resistance to rabbit but is strongly inhibited by the pig serum. (PDF) [file pone.0144937.s014.pdf]

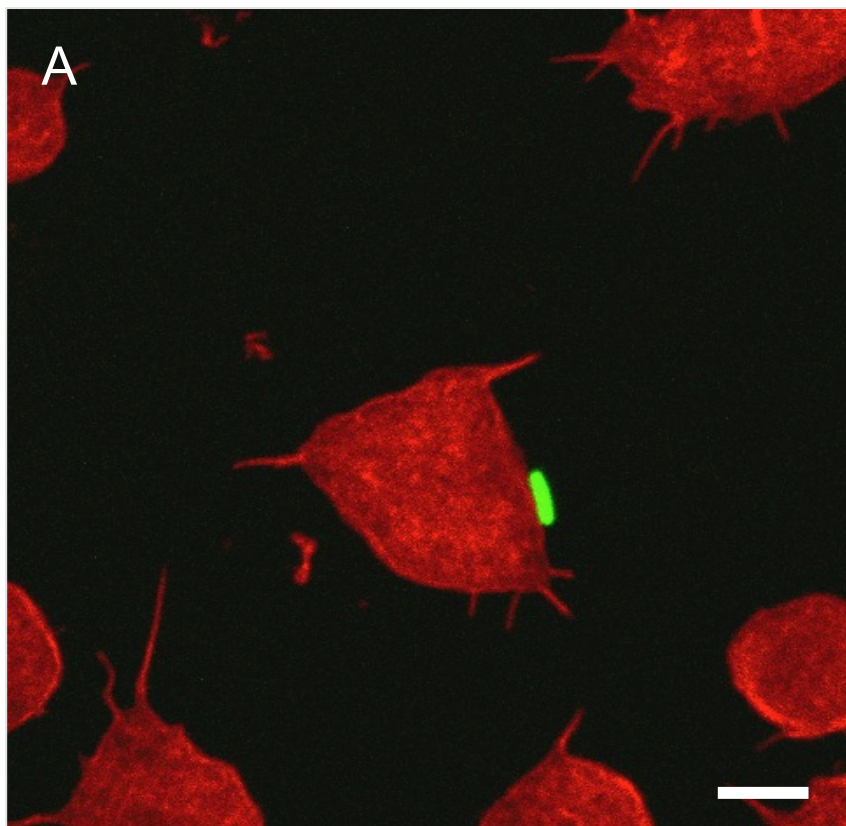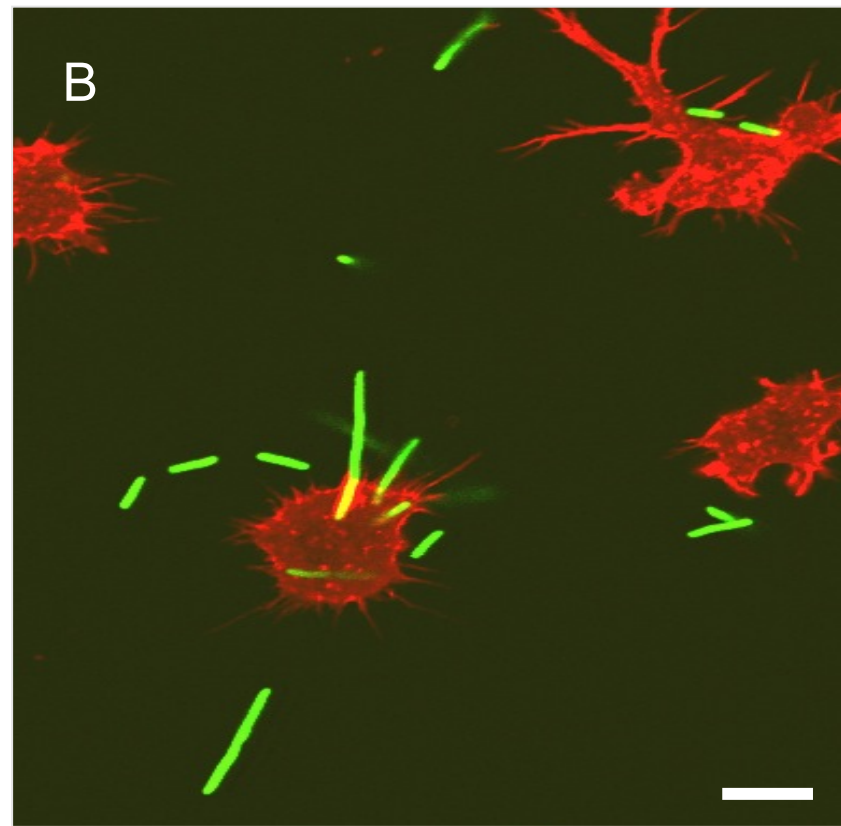

C

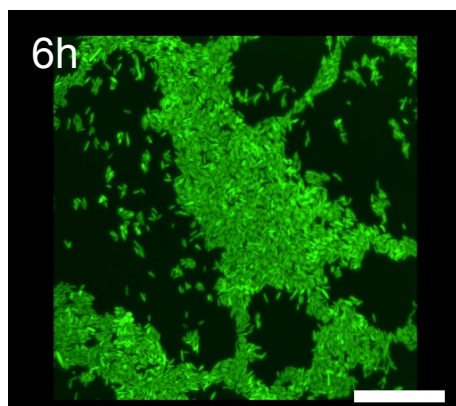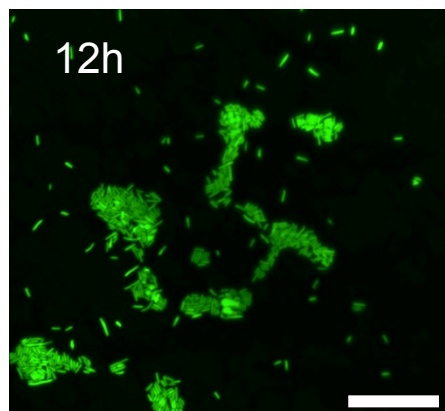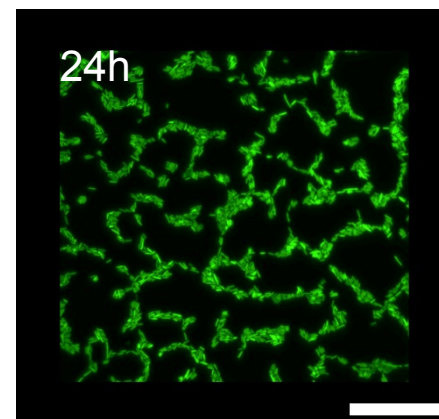

D

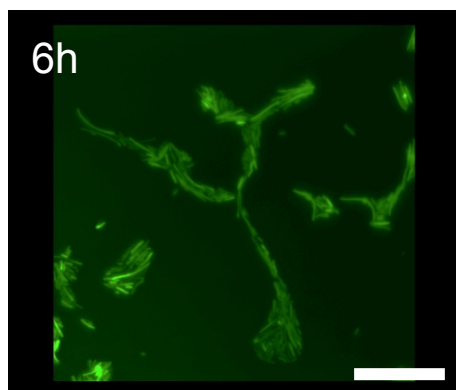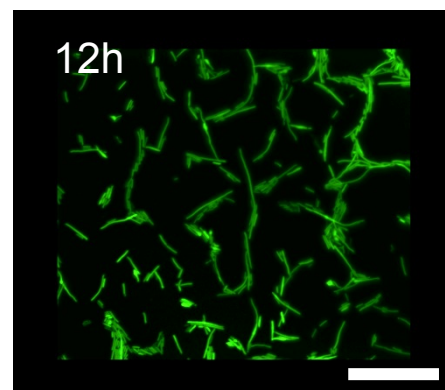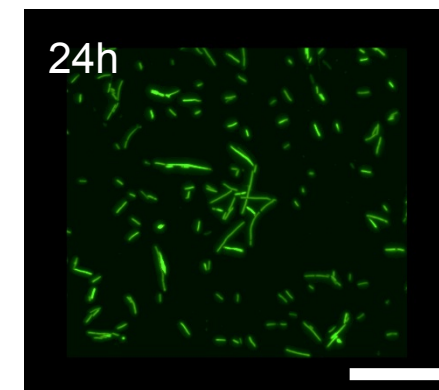

E

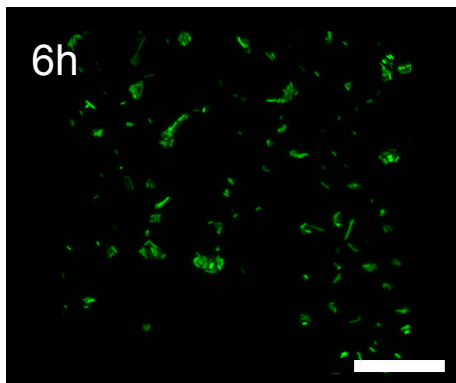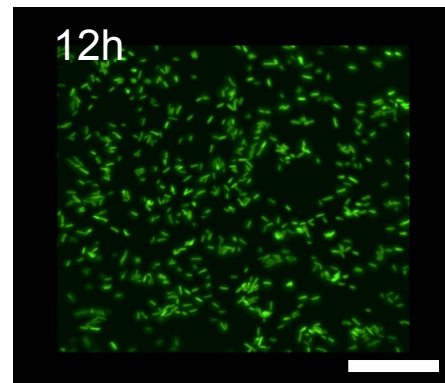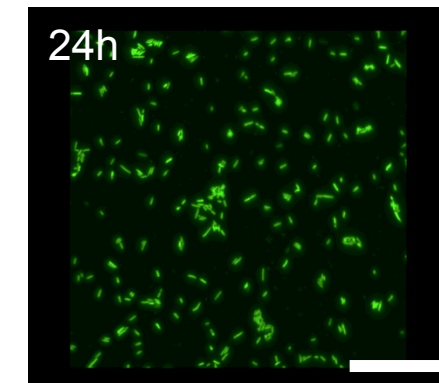

Supplement: S10 Fig — (A-B) Bacterial morphology and behaviour of GFP labelled Pa ATCC43949 when exposed to (A) Manduca sexta haemocytes (bled from a whole animal infection) at 28°C in normal air and (B) cultured J774.2 murine macrophage-like cells at 37°C in a 5% CO2 incubator. The host cell actin cytoskeleton is labelled with Phalloidin:TRITC stain in both cases and slides visualised on a confocal microscope. White bar represents 10μM. (C-E) Time courses showing representative Pa ATCC43949 cell morphology and early biofilm development in LB medium in static conditions on glass slides under different conditions, (C) at 28°C in normal air, (D) at 37°C in 5% C02 and (E) at 37°C in normal air. White bar represents 500μM. Note a combination of 37°C and 5% C02 induces filamentation as also seen in panel (B). (PDF) [file pone.0144937.s015.pdf]
