# Supplementary material for: From Insect to Man: Photorhabdus Sheds Light on the Emergence of Human Pathogenicity
Source: PLoS One. 2015 Dec 17;10(12):e0144937. doi: 10.1371/journal.pone.0144937 (PMC4683029; doi:10.1371/journal.pone.0144937)
Supplement: S5 Fig — Panels (A-C) show P. asymbiotica ATCC43949, (D-F) show P. asymbiotica Kingscliff and (G-H) show P. luminescens TT01. The strains were cultured on solid agar media under aerobic or microaerobic conditions at 28°C and 37°C. Various supplements were added to the plates as indicated. In the Oxidation/Fermentation (O/F) plates the bromothymol blue indicator change from blue to yellow indicates acid production from the supplemented carbon source. (PDF) [file pone.0144937.s010.pdf]

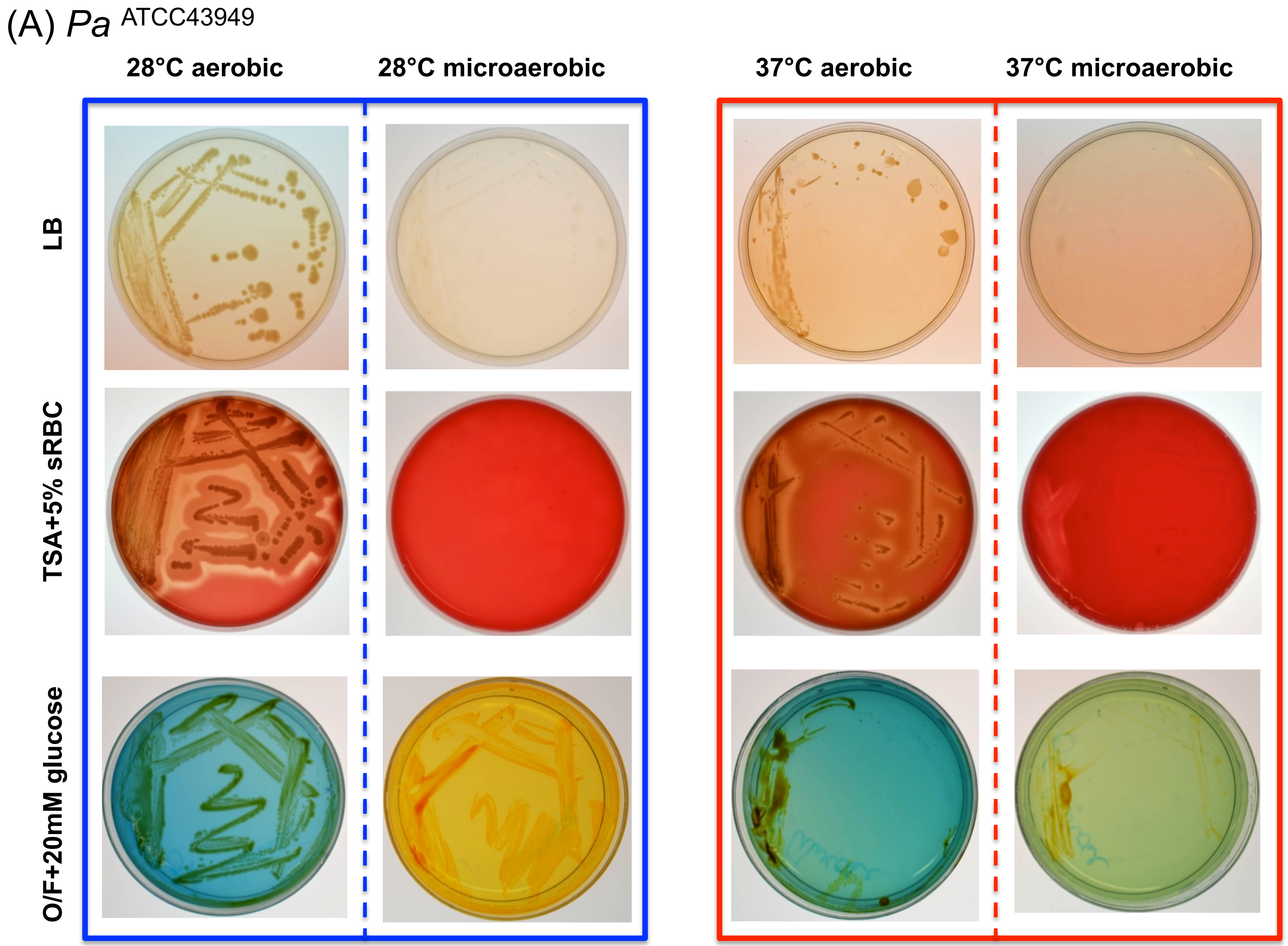

(A) Pa ATCC43949
28°C aerobic
28°C microaerobic
37°C aerobic
37°C microaerobic
LB
TSA+5% sRBC
O/F+20mM glucose

(B) *Pa* ATCC43949

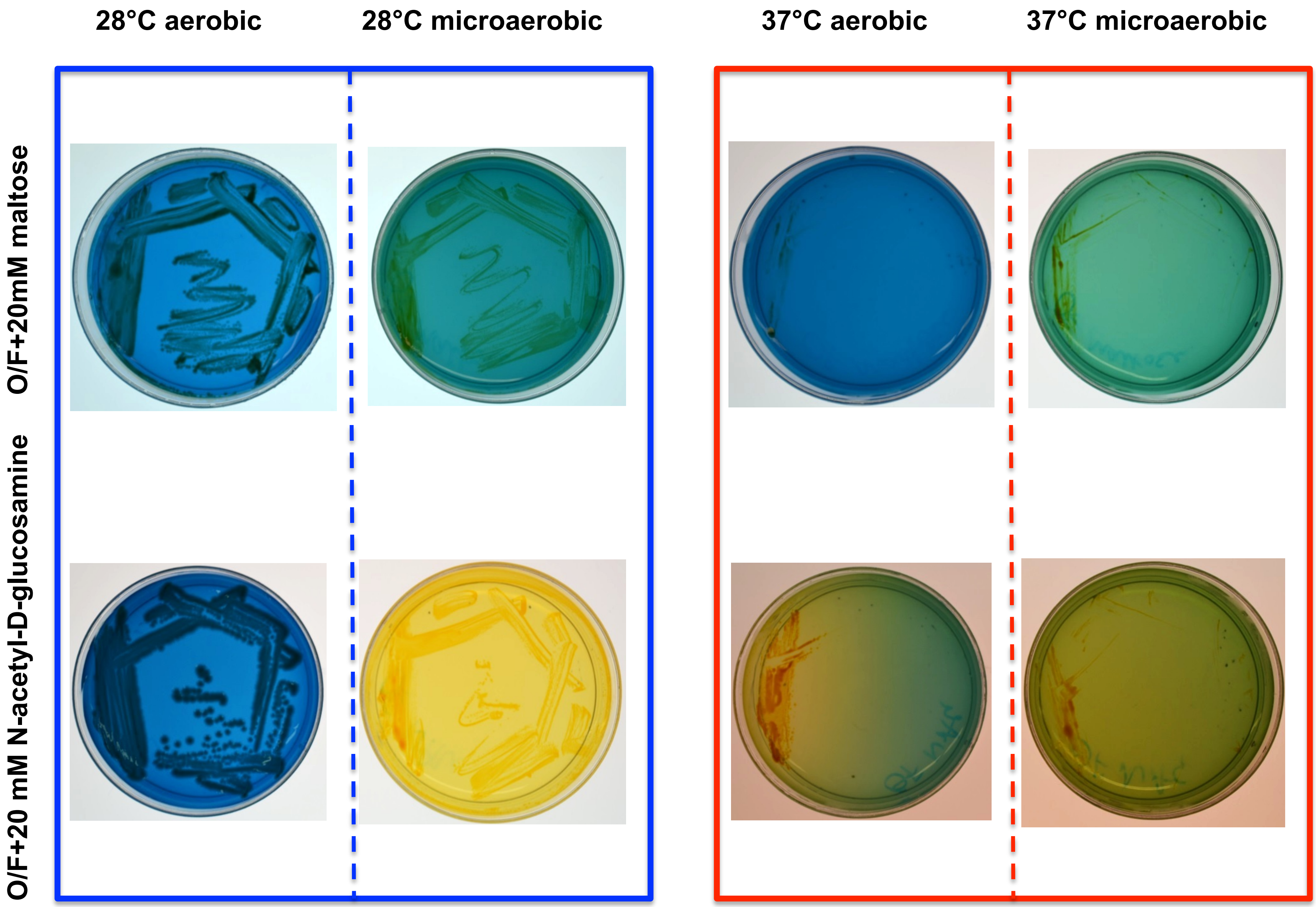

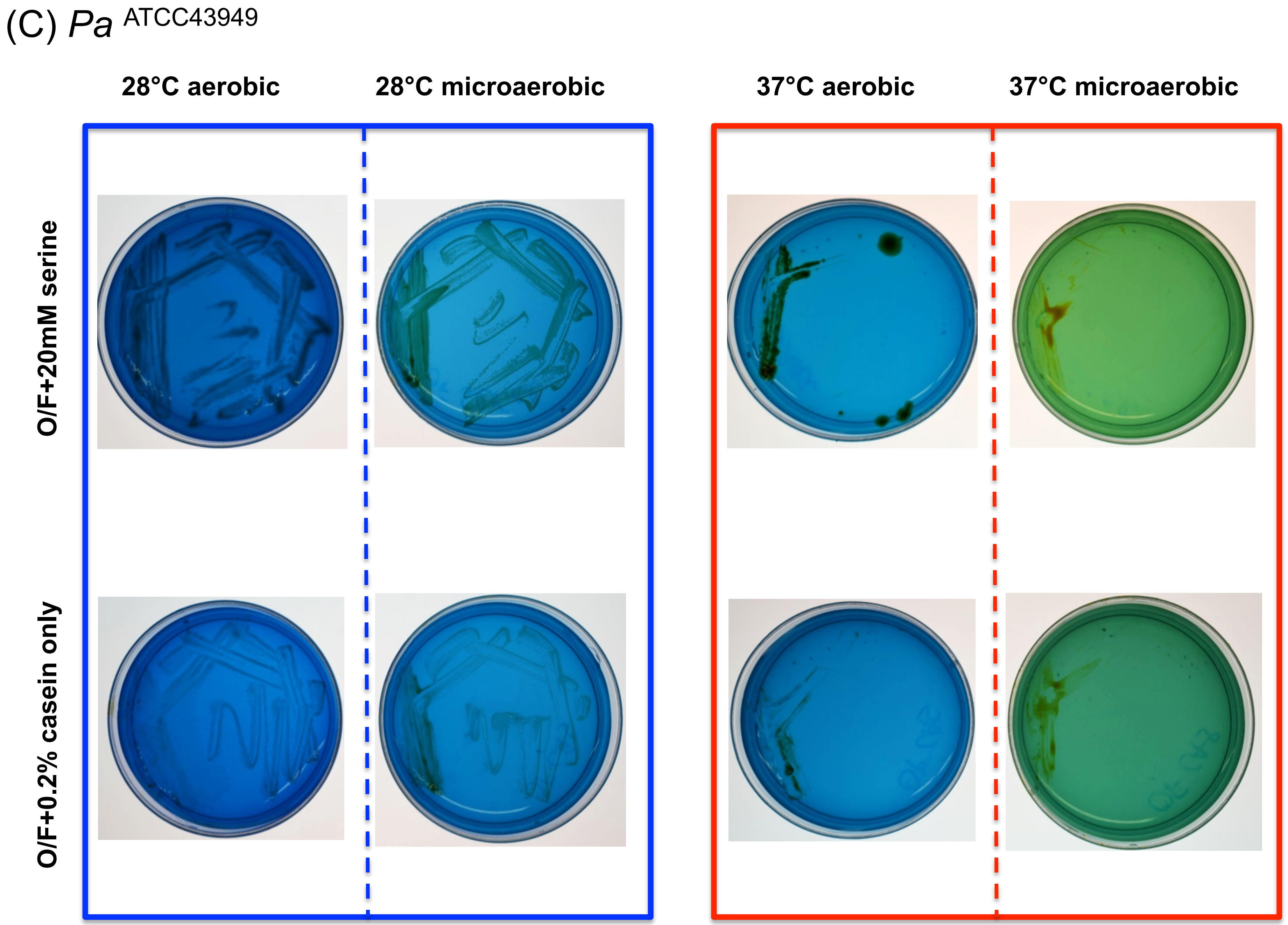
(C) Pa ATCC43949
28°C aerobic
28°C microaerobic
37°C aerobic
37°C microaerobic
O/F+20mM serine
O/F+0.2% casein only

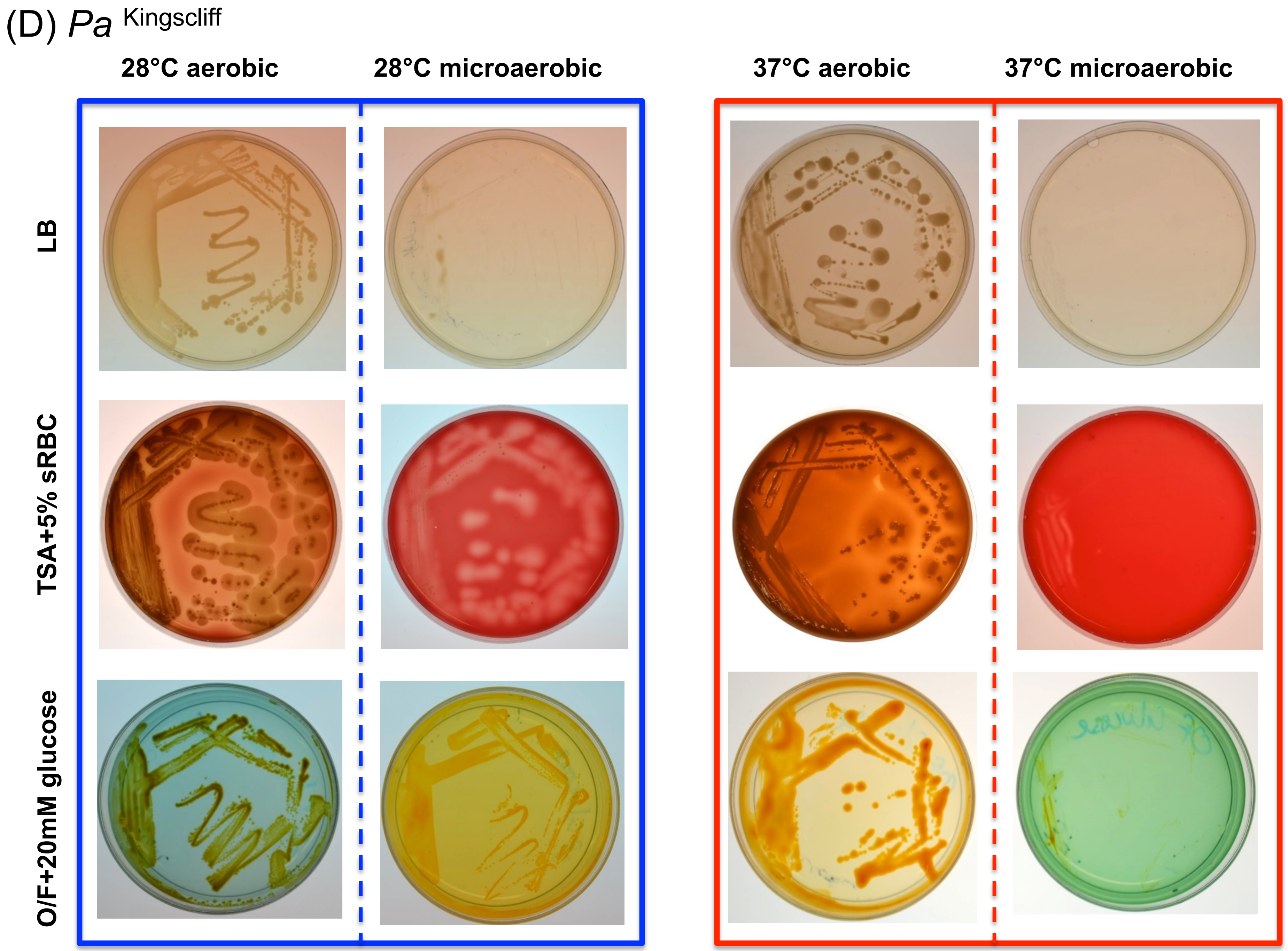

(D) Pa Kingscliff
28°C aerobic
28°C microaerobic
37°C aerobic
37°C microaerobic
LB
TSA+5% sRBC
O/F+20mM glucose

(E) *Pa* Kingscliff

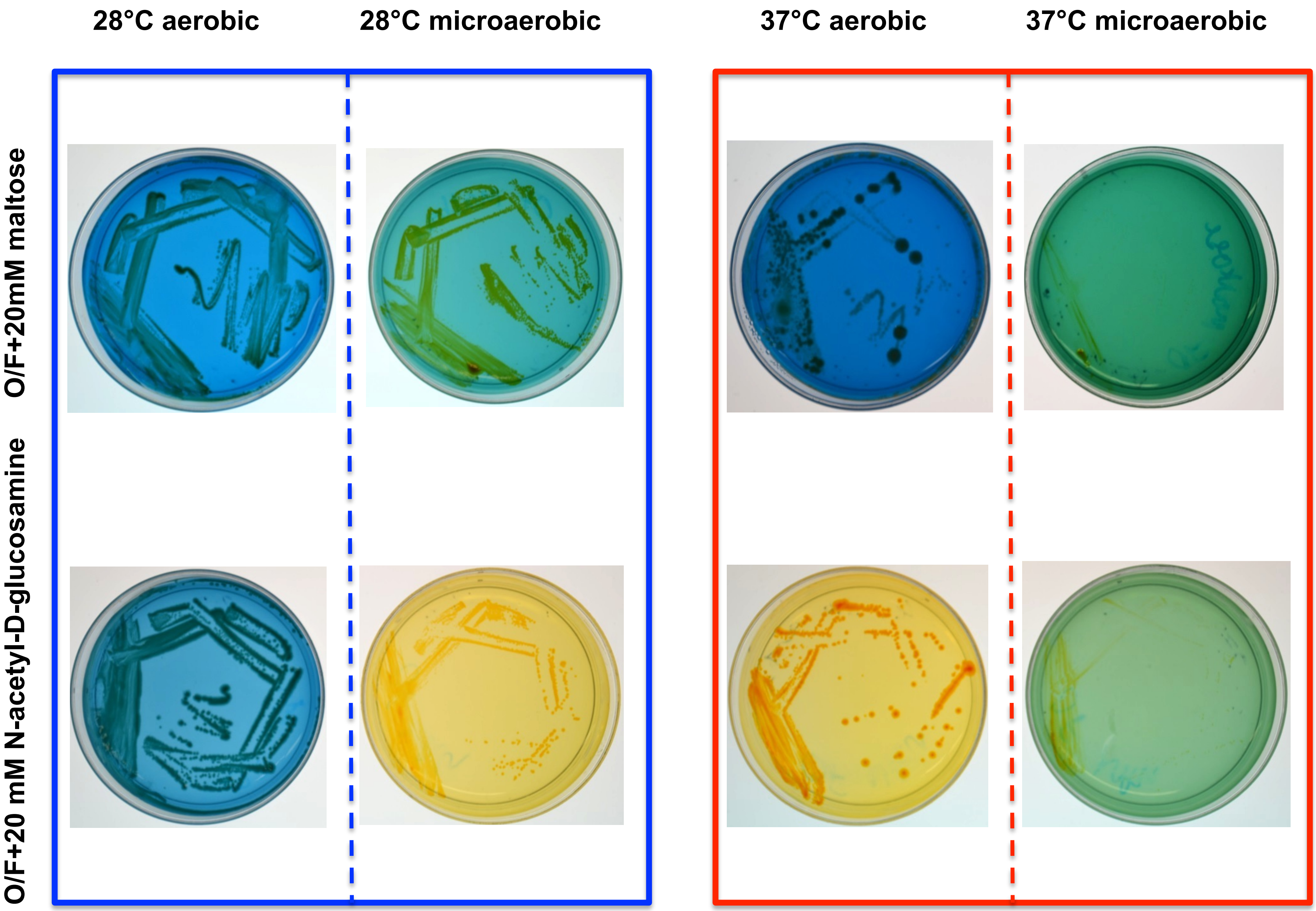

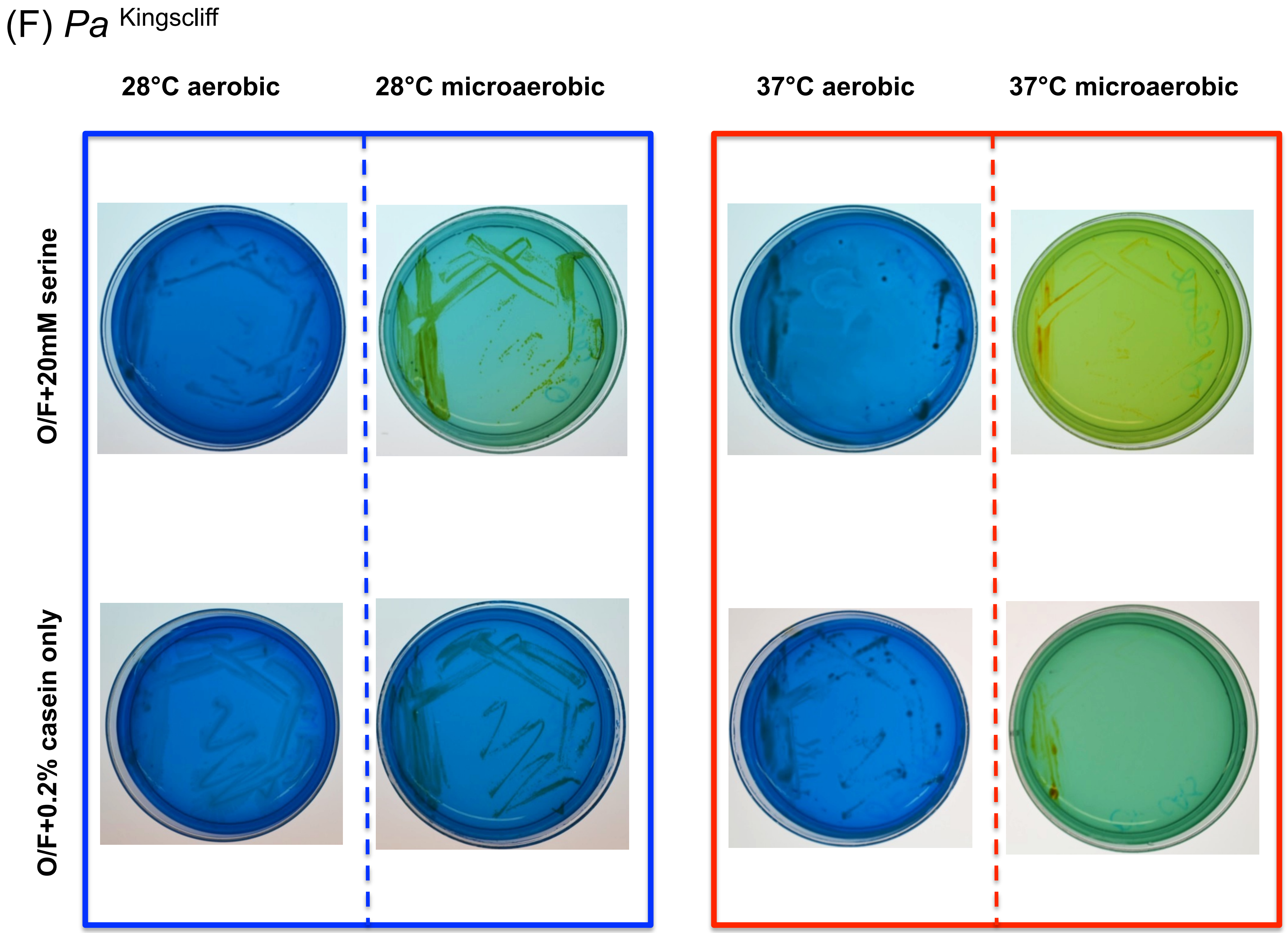
(F) Pa Kingscliff
28°C aerobic
28°C microaerobic
37°C aerobic
37°C microaerobic
O/F+20mM serine
O/F+0.2% casein only

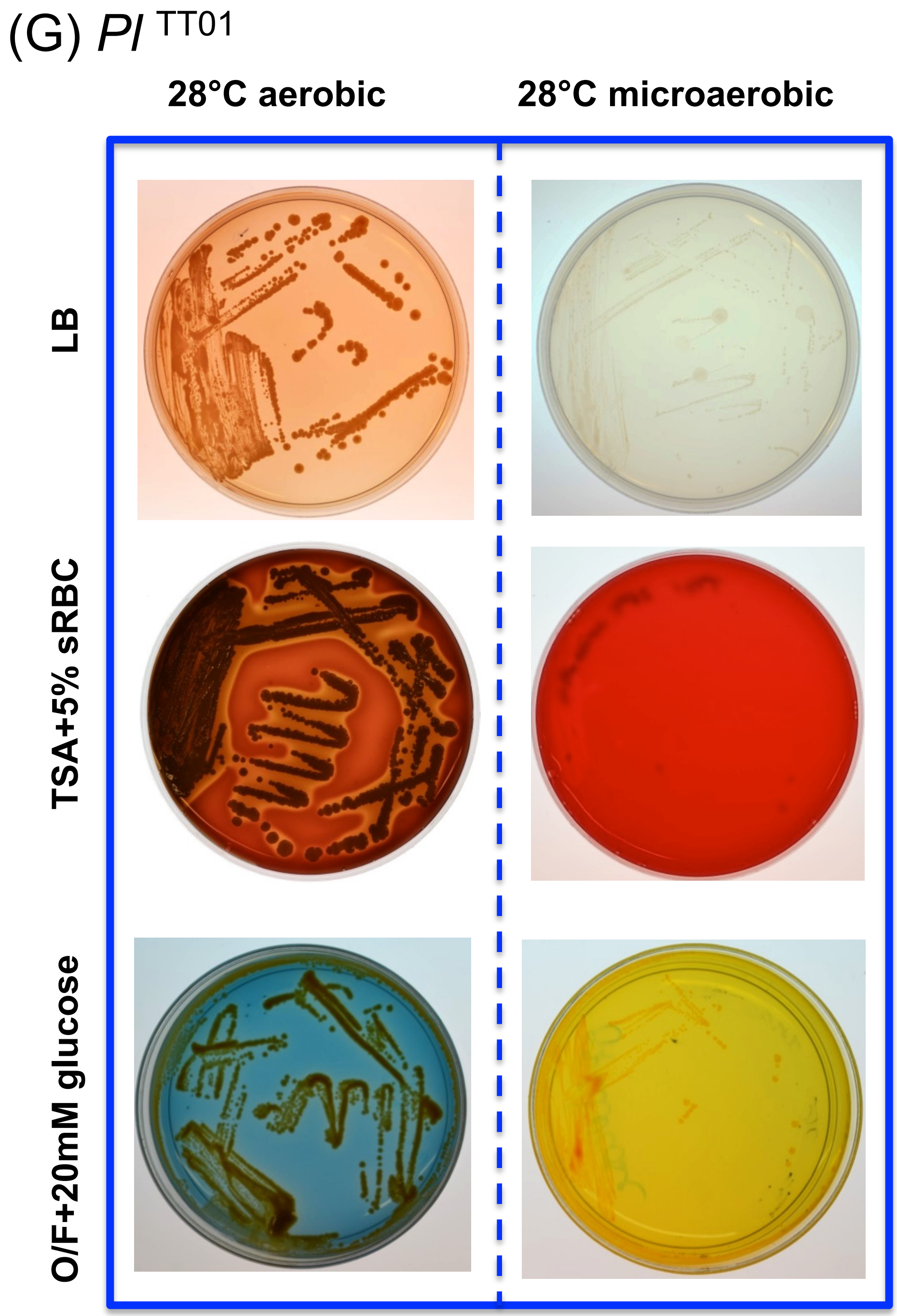

(G) Pl TT01
28°C aerobic
28°C microaerobic
LB
TSA+5% sRBC
O/F+20mM glucose

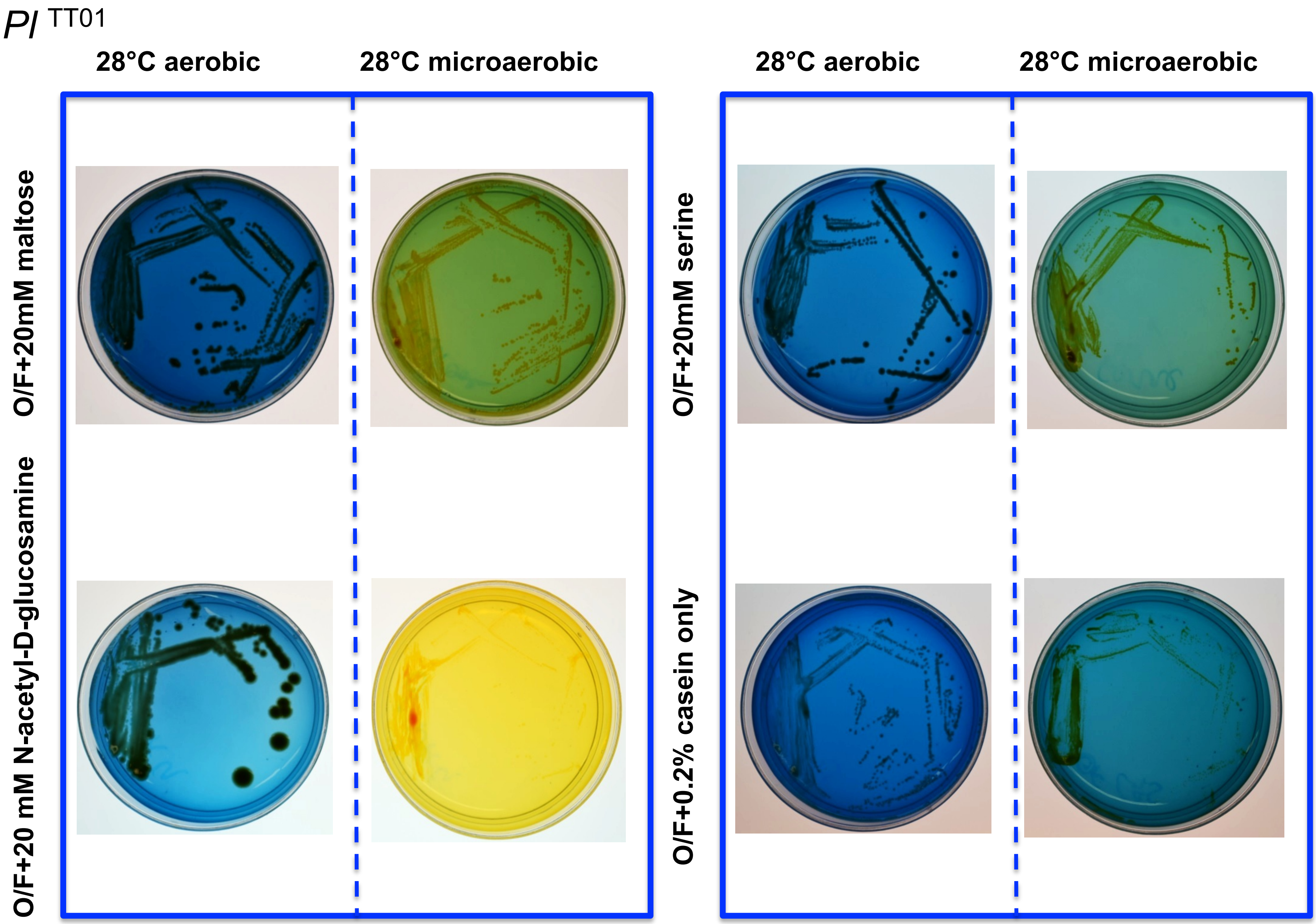

Pl TT01
28°C aerobic
28°C microaerobic
28°C aerobic
28°C microaerobic
O/F+20mM maltose
O/F+20 mM N-acetyl-D-glucosamine
O/F+20mM serine
O/F+0.2% casein only
