# Supplementary material for: From Insect to Man: Photorhabdus Sheds Light on the Emergence of Human Pathogenicity
Source: PLoS One. 2015 Dec 17;10(12):e0144937. doi: 10.1371/journal.pone.0144937 (PMC4683029; doi:10.1371/journal.pone.0144937)
Supplement: S1 Table — (DOCX) [file pone.0144937.s016.docx]

**Table S1. Transcripts more abundant at 37°C than at 28°C.** Summary of *P. asymbiotica*^ATCC43949^ genes showing higher transcription at 37°C compared to 28°C as deduced by DESeq analysis of triplicate RNA-seq experiments. Significant changes in mRNA level are defined by the cut off criteria used of >1.95 Log_2_fold change and a P-value of <0.1. The mean base mapping level (BM) is shown for each gene at the two temperatures. Locus tags in italics indicate genes with paralogues in *P. asymbiotica*^Kingscliff^ but not *P. luminescens*^TT01^.

| **Locus tag** | **BM 28°C** | **BM 37°C** | **Log_2_Fold** | **P-Value** | **Protein-function** |
| --- | --- | --- | --- | --- | --- |
| ***Peptide and amino acid metabolism*** | | | | | |
| PAU_00048 | 70.01 | 577.69 | 3.044 | 0.00007 | AsnA, asparagine synthetase |
| PAU_00605 | 53.49 | 1804.82 | 5.076 | <.00001 | PrtA secreted metalloprotease |
| PAU_00606 | 6.54 | 59.73 | 3.191 | 0.00471 | PrtA inhibitor |
| PAU_00607 | 7.47 | 59.83 | 3.001 | 0.00040 | PrtB, PrtA export ABC transporter |
| PAU_00608 | 11.03 | 54.18 | 2.297 | 0.00539 | PrtC, PrtA export ABC transporter |
| PAU_00753 | 28.95 | 119.19 | 2.041 | 0.09596 | Thermostable carboxypeptidase |
| PAU_02032 | 37.37 | 196.51 | 2.394 | 0.01866 | Solute binding protein OppA-like oligopeptide ABC transporter |
| PAU_02033 | 105.31 | 1040.82 | 3.304 | 0.02279 | Solute binding protein OppA-like oligopeptide ABC transporter |
| PAU_02203 | 149.58 | 944.51 | 2.658 | 0.00035 | D-amino-peptidase, release of an N-terminal D-amino acid from peptide). |
| PAU_02334 | 39.12 | 263.85 | 2.753 | 0.01863 | Enhancin-like, M60 peptidase domain |
| PAU_02337 | 19.12 | 117.13 | 2.614 | 0.00082 | Solute binding protein OppA-like oligopeptide ABC transporter |
| PAU_02342 | 3.97 | 51.50 | 3.698 | 0.00062 | Solute binding protein OppA-like oligopeptide ABC transporter |
| PAU_02393 | 10.85 | 122.64 | 3.498 | 0.03245 | AdoMet-synthetase, joins methionine and ATP in methionine cycle |
| PAU_02951 | 823.26 | 3473.70 | 2.077 | 0.07802 | AroG, enzyme in shikimate pathway for biosynthesis of phenylalanine, tyrosine, and tryptophan |
| PAU_03993 | 199.30 | 793.51 | 1.993 | 0.00747 | Solute binding protein predicted amino acid ABC transporter (cystine?) |
| ***Central metabolism and intermediate biochemistry*** | | | | | |
| PAU_00422 | 5.50 | 30.17 | 2.456 | 0.00742 | Putative alpha/beta hydrolase |
| *PAU_00750* | 120.81 | 580.12 | 2.263 | 0.00218 | PaaA-like, molybdopterin/thiamine biosynthesis |
| PAU_01924 | 14.32 | 92.18 | 2.686 | 0.00098 | Cyclopropane fatty acyl phospholipid synthase with AdoMet binding site, one-carbon group methyltransferase |
| PAU_02072 | 9.22 | 37.37 | 2.019 | 0.01895 | Putative epoxide hydrolase |
| PAU_02326 | 5.33 | 24.98 | 2.228 | 0.01415 | ACAD, acyl-CoA dehydrogenase, for initial step in each cycle of fatty acid β-oxidation |
| *PAU_02907* | 25.17 | 225.26 | 2.088 | 0.00555 | Putative NADP-dependent oxidoreductase |
| PAU_03622 | 12.70 | 59.15 | 2.018 | 0.01238 | Isopentenyl-diphosphate delta-isomerase |
| ***Nucleotide metabolism*** | | | | | |
| *PAU_00613* | 29.37 | 147.16 | 2.324 | 0.00266 | Predicted 5'-nucleotidase haloacid dehalogenase |
| PAU_00614 | 57.27 | 368.21 | 2.684 | 0.00040 | Predicted nucleotidyl-transferase |
| PAU_02369 | 5.32 | 30.61 | 2.523 | 0.00490 | Predicted uracil phosphoribosyl-transferase (UMP synthesis) |
| PAU_02388 | 20.35 | 80.96 | 1.992 | 0.04429 | Short-chain dehydrogenase |
| PAU_02389 | 28.43 | 181.57 | 2.674 | 0.00471 | Predicted NUDIX phosphohydrolase |
| PAU_02390 | 17.70 | 102.22 | 2.530 | 0.00599 | Weak NUDIX family similarity |
| PAU_02391 | 20.83 | 114.20 | 2.455 | 0.00244 | Hypothetical |
| PAU_02392 | 19.03 | 138.94 | 2.868 | 0.01745 | Putative pyridoxal phosphate-dependent aminotransferase |
| PAU_02394 | 18.98 | 138.37 | 2.865 | 0.00644 | Predicted carbamoyltransferase, pyrimidine biosynthesis |
| PAU_02396 | 93.69 | 481.98 | 2.363 | 0.00144 | Predicted myo-inositol-1-phosphate synthase |
| ***Putative virulence factors*** | | | | | |
| PAU_00419 | 13.77 | 54.74 | 1.990 | 0.03683 | Photopexin PhxA-like with fructose-binding lectin II (PA-IIL) domain |
| PAU_00820 | 694.76 | 4228.32 | 2.605 | 0.06986 | Carbonic anhydrase associated with intra-cellular pathogens |
| PAU_02143 | 35.45 | 267.82 | 2.917 | 0.07170 | Putative deoxyribonucleotide triphosphate pyrophosphatase, similar to C-terminus of SepC toxin |
| PAU_02406 | 39.42 | 206.49 | 2.388 | 0.01286 | Type I fimbrial subunit assembly |
| PAU_02408 | 2.63 | 11.99 | 2.186 | 0.04383 | PapC-like, P-pilus assembly protein porin |
| PAU_02531 | 22.37 | 185.97 | 3.055 | 0.09414 | Bacterial Ig-domain protein, putative Invasin/adhesin protein |
| PAU_02532 | 13.76 | 106.41 | 2.951 | 0.01185 | Hypothetical encoded in PAU_02531 operon |
| PAU_02533 | 21.92 | 236.70 | 3.432 | 0.05911 | Hypothetical encoded in PAU_02531 operon |
| PAU_02534 | 12.17 | 129.83 | 3.414 | 0.04572 | Hypothetical encoded in PAU_02531 operon |
| PAU_02535 | 169.17 | 2423.15 | 3.840 | 0.00601 | Hypothetical encoded in PAU_02531 operon |
| PAU_02536 | 97.54 | 1269.55 | 3.702 | 0.01279 | Hypothetical encoded in PAU_02531 operon |
| PAU_02537 | 70.80 | 315.95 | 2.157 | 0.00721 | Hypothetical encoded in PAU_02531 operon |
| PAU_02801 | 98.29 | 487.75 | 2.311 | 0.00196 | PVC unit 1 protein, Afp13-like |
| PAU_02802 | 66.81 | 277.48 | 2.054 | 0.01602 | PVC unit 1 protein, Afp14-like |
| PAU_02803 | 54.06 | 227.04 | 2.070 | 0.00546 | PVC unit 1 protein, Afp15-like |
| PAU_02804 | 43.31 | 227.71 | 2.394 | 0.07653 | PVC unit 1 protein, Afp16-like |
| PAU_02805 | 60.27 | 323.22 | 2.423 | 0.00140 | Putative PVC unit 1 effector protein |
| *PAU_02806* | 102.16 | 531.55 | 2.379 | 0.00128 | Putative PVC unit 1 effector protein, TccC toxin like |
| PAU_03369 | 29.28 | 128.10 | 2.129 | 0.00497 | Mcf1 pro-apoptosis multi-domain toxin |
| PAU_03717 | 12.56 | 49.55 | 1.979 | 0.01489 | PirB insecticidal toxin |
| PAU_03890 | 89.15 | 4791.40 | 5.748 | <.00001 | TAT secreted Intradiol dioxygenase, O_2_ dependent conversion of catecholate derivatives to TCA intermediates |
| ***Motility*** | | | | | |
| PAU_02610 | 32.02 | 308.27 | 3.267 | 0.01473 | FliD, flagellar hook-associated protein 2 |
| PAU_02642 | 45.67 | 178.18 | 1.964 | 0.00949 | FlgK flagellar hook-associated protein |
| PAU_02685 | 11.88 | 89.41 | 2.911 | 0.02217 | CheD, methyl accepting chemotaxis protein I (serine chemoreceptor) |
| ***Sensing and regulation*** | | | | | |
| *PAU_00212* | 456.49 | 2510.84 | 2.459 | 0.00096 | Putative HTH-type transcriptional regulator |
| PAU_00252 | 18.73 | 72.62 | 1.954 | 0.00947 | SdiA-like transcription regulator |
| PAU_01544 | 25.06 | 176.81 | 2.818 | 0.09978 | Putative HTH-type DNA binding protein |
| PAU_02297 | 92.10 | 556.23 | 2.594 | 0.02890 | AraC-family transcriptional regulator |
| *PAU_02251* | 3.69 | 34.81 | 3.236 | 0.02776 | LysR-family transcriptional regulator |
| *PAU_02908* | 16.09 | 76.78 | 2.254 | 0.00597 | YdhM-like protein with similarity to TetR-family regulators |
| ***Secondary metabolite production and putative quorum sensing*** | | | | | |
| PAU_01180 | 19.49 | 96.41 | 2.306 | 0.00354 | Fortimicin (a pseudo-disaccharide amino-glycoside) antibiotic synthesis-like protein, n-forimidoyltransferase |
| PAU_01181 | 40.74 | 400.86 | 3.298 | 0.00131 | AdoMet-dependent methyltransferase, potentially contributing to haem biosynthesis or fortimicin synthesis |
| PAU_01182 | 14.13 | 106.37 | 2.912 | 0.00035 | BioF-like, class II aminotransferase, used in fortimicin antibiotic synthesis |
| PAU_01183 | 44.07 | 320.69 | 2.863 | 0.00019 | PapB/chorismate mutase/prephenat de-hydrogenase-like, used in fortimicin antibiotic synthesis |
| PAU_01184 | 73.37 | 770.45 | 3.392 | 0.09504 | Putative *p*-aminobenzoic acid synthase-like, used in polyene macrolide antibiotic synthesis |
| PAU_01468 | 107.19 | 963.22 | 3.167 | 0.09406 | LsrG autoinducer-2 (AI-2) degradation protein |
| PAU_01469 | 245.07 | 1545.32 | 2.656 | 0.00068 | LsrF Autoinducer (AI-2) aldolase |
| *PAU_02219* | 79.90 | 348.85 | 2.126 | 0.03962 | SypC-like, NRPS acetyltransferase |
| *PAU_02220* | 71.90 | 308.77 | 2.102 | 0.00517 | SypC-like NRPS oxidoreductase |
| PAU_02513 | 35.30 | 147.95 | 1.974 | 0.01363 | LuxD, acyl transferase involved in luciferase substrate re-cycling |
| PAU_02514 | 64.88 | 258.55 | 1.994 | 0.01624 | LuxC, acyl-CoA reductase involved in luciferase substrate re-cycling |
| PAU_03067 | 5.82 | 75.74 | 3.701 | 0.00002 | GrpS, GameXPeptide NRPS |
| PAU_01720 | 44.93 | 845.44 | 4.234 | 0.00056 | IsnB Rhabduscin aglycon precursor synthesis |
| PAU_01721 | 52.26 | 443.96 | 3.086 | 0.01600 | IsnA Rhabduscin aglycon precursor synthesis |
| ***Cell wall and membrane*** | | | | | |
| PAU_00481 | 53.90 | 213.30 | 1.984 | 0.00782 | AttM-like Zn dependent hydrolase, potential beta-lactamase |
| PAU_01378 | 3.97 | 467.51 | 6.881 | 0.09669 | Putative metallo-beta-lactamase |
| ***Stress response*** | | | | | |
| PAU_01563 | 21.91 | 187.79 | 3.099 | 0.00009 | Putative stress responsive A/B barrel domain-containing protein |
| PAU_03190 | 164.54 | 1272.35 | 2.951 | 0.00064 | ClpB disaggregation chaperone |
| PAU_03384 | 1772.28 | 10814.7 | 2.609 | 0.00042 | HtpG (Hsp90) heat shock chaperone |
| ***Other transporters*** | | | | | |
| PAU_00162 | 45.67 | 184.31 | 2.013 | 0.00861 | PstS, phosphate-binding periplasmic protein, high affinity phosphate uptake ABC transporter |
| *PAU_02200* | 1.27 | 10.09 | 2.991 | 0.01624 | ABC transporter, ATP-binding protein |
| PAU_02694 | 6.96 | 28.82 | 2.049 | 0.02261 | MgtC-like, magnesium ion transport ATPase protein |
| ***Vitamin and co-factor synthesis*** | | | | | |
| PAU_00682 | 96.99 | 468.02 | 2.270 | 0.00224 | CysD, involved in cysteine biosynthesis |
| PAU_01543 | 180.41 | 782.51 | 2.116 | 0.00359 | Putative, menaquinone vitaminK2 biosynthesis |
| PAU_01601 | 10.76 | 144.53 | 3.747 | 0.04441 | CbiA, cobyrinic acid diamide synthase |
| PAU_01604 | 7.83 | 63.44 | 3.018 | 0.08993 | CbiD, cobalt-precorrin-6A synthase |
| PAU_01610 | 4.81 | 49.30 | 3.358 | 0.07808 | CbiJ, cobalt-precorrin- reductase |
| ***Information processing*** | | | | | |
| PAU_00759 | 41.87 | 267.55 | 2.675 | 0.00186 | LysS, lysine-tRNA ligase |
| PAU_02882 | 64.59 | 299.88 | 2.215 | 0.07964 | Putative aminoacyl-tRNA editing domain protein |
| ***Mobile elements and unknown function*** | | | | | |
| PAU_00254 | 4.54 | 23.84 | 2.392 | 0.01498 | PixA superfamily-like inclusion body protein |
| PAU_01142 | 3.47 | 22.02 | 2.666 | 0.04805 | Hypothetical protein |
| PAU_01811 | 8.29 | 48.63 | 2.552 | 0.06012 | Predicted Fe-S oxidoreductase protein |
| PAU_01858 | 1.74 | 11.08 | 2.669 | 0.01975 | Putative bacteriophage tail fibre protein |
| PAU_01891 | 9.82 | 47.98 | 2.288 | 0.00620 | Hypothetical protein |
| PAU_02670 | 15.35 | 120.78 | 2.975 | 0.00018 | Cupin-2 domain superfamily protein, includes enzymes and seed storage proteins |
| *PAU_02724* | 161.16 | 855.67 | 2.408 | 0.00107 | Putative membrane protein |
| PAU_02748 | 4.53 | 34.31 | 2.9216 | 0.03237 | Hypothetical secreted protein |
| PAU_02821 | 6.81 | 86.79 | 3.6717 | 0.05860 | Possible prolyl 4-hydroxylase |
| PAU_03812 | 2.06 | 16.16 | 2.9718 | 0.00829 | Hypothetical |
| PAU_03921 | 9.05 | 37.67 | 2.0577 | 0.01709 | Weak similarity with bacteriophage protein |
