# Supplementary material for: From Insect to Man: Photorhabdus Sheds Light on the Emergence of Human Pathogenicity
Source: PLoS One. 2015 Dec 17;10(12):e0144937. doi: 10.1371/journal.pone.0144937 (PMC4683029; doi:10.1371/journal.pone.0144937)
Supplement: S2 Table — (DOCX) [file pone.0144937.s017.docx]

**Table S2. Transcripts more abundant at 28°C than at 37°C.** Summary of *P. asymbiotica*^ATCC43949^ genes showing higher transcription at 28°C compared to 37°C as deduced by DESeq analysis of triplicate RNA-seq experiments. Significant changes in mRNA level are defined by the cut off criteria used of >1.95 Log_2_fold change and a P-value of <0.1. The mean base mapping level (BM) is shown for each gene at the two temperatures. Locus tags in italics indicate genes with paralogues in *P. asymbiotica*^Kingscliff^ but not *P. luminescens*^TT01^.

| **Locus tag** | **BM 28°C** | **BM 37°C** | **Log_2_Fold** | **P-Value** | **Protein-function** |
| --- | --- | --- | --- | --- | --- |
| ***Peptide and amino-acid metabolism*** | | | | | |
| PAU_01354 | 2392.87 | 593.87 | -2.0105 | 0.00689 | GlyA, serine hydroxyl-methyltransferase for inter-conversion of serine/glycine, important role in one-carbon pathways |
| PAU_02598 | 386.68 | 20.92 | -4.2085 | <.00001 | DsdA, serine dehydratase (deamination of serine to pyruvate+ammonia) |
| PAU_02599 | 439.73 | 19.20 | -4.5175 | <.00001 | DsdX, D-serine importer permease |
| ***Central metabolism and intermediate biochemistry*** | | | | | |
| PAU_01920 | 58.00 | 14.22 | -2.0282 | 0.01377 | PykF, pyruvate kinase (regulates glycolysis) |
| *PAU_03776* | 1825.42 | 76.04 | -4.5854 | 0.02438 | Predicted secreted short-chain dehydrogenase/reductase |
| ***Carbohydrate metabolism*** | | | | | |
| PAU_00364 | 85.83 | 13.60 | -2.6578 | 0.00104 | MalM, maltose regulon periplasmic protein |
| PAU_00365 | 98.46 | 15.05 | -2.7098 | 0.00080 | LamB, maltose-inducible maltoporin |
| PAU_00366 | 77.35 | 13.52 | -2.5160 | 0.00732 | MalK, maltose/ maltodextrin transporter ATP-binding protein |
| PAU_00376 | 56.95 | 8.16 | -2.8032 | 0.00101 | MalQ, 4-alpha-glucanotransferase |
| PAU_00377 | 101.99 | 15.35 | -2.7319 | 0.00076 | MalP, maltodextrin phosphorylase |
| PAU_01238 | 141.41 | 23.66 | -2.5792 | 0.00098 | Glycosyl hydrolase domain protein |
| ***Nucleotide metabolism*** | | | | | |
| PAU_00190 | 48.27 | 6.61 | -2.8685 | 0.00102 | Xanthine/uracil permease family protein |
| PAU_00405 | 796.78 | 128.67 | -2.6305 | 0.00050 | PurD, phosphor-ribosylglycinamide synthetase required for *de novo* purine biosynthesis |
| PAU_00406 | 457.43 | 68.43 | -2.7408 | 0.00029 | PurH, bifunctional purine biosynthesis protein |
| PAU_00996 | 72.06 | 14.48 | -2.3147 | 0.00362 | PurK, purine biosynthesis, phosphoribosyl-aminoimidazole carboxylase ATPase |
| PAU_00997 | 64.32 | 12.63 | -2.3483 | 0.00402 | PurE, purine biosynthesis, phosphoribosyl-aminoimidazole carboxylase subunit |
| PAU_01779 | 1152.58 | 219.19 | -2.3946 | 0.00301 | PurN, phosphoribosyl-glycinamide formyltransferase |
| PAU_01780 | 443.81 | 79.32 | -2.4842 | 0.00091 | PurM, phosphorribosyl-formylglycinamidine cyclo-ligase |
| PAU_01826 | 1007.91 | 259.76 | -1.9561 | 0.00725 | GuaB, inosine-5'-monophosphate dehydrogenase |
| PAU_01827 | 1609.40 | 348.54 | -2.2071 | 0.00253 | GuaA, glutamine-hydrolyzing GMP synthase |
| ***Putative virulence factors*** | | | | | |
| PAU_01534 | 292.01 | 64.52 | -2.1781 | 0.00388 | XaxA toxin |
| PAU_02310 | 69.96 | 17.33 | -2.0130 | 0.05912 | Putative hydrolase or acetytranferase |
| PAU_02313 | 35.07 | 8.16 | -2.1038 | 0.01758 | Probable type VI secretion protein |
| PAU_02314 | 26.65 | 6.37 | -2.0644 | 0.09329 | Hypothetical protein in putative type VI operon |
| PAU_02316 | 51.79 | 11.61 | -2.1574 | 0.00799 | Hypothetical lipoprotein in putative type VI operon |
| PAU_02317 | 67.22 | 16.20 | -2.0528 | 0.01039 | Hypothetical lipoprotein in putative type VI operon |
| PAU_02318 | 88.77 | 14.84 | -2.5808 | 0.00121 | Hypothetical lipoprotein in putative type VI operon |
| PAU_02319 | 133.01 | 20.94 | -2.6672 | 0.04125 | Hypothetical lipoprotein in putative type VI operon |
| PAU_02320 | 20.98 | 2.76 | -2.9243 | 0.01307 | Type VI secretion ImpA family protein |
| PAU_02321 | 311.31 | 73.65 | -2.0797 | 0.02976 | Type VI secretion protein |
| PAU_03902 | 55.78 | 11.21 | -2.3153 | 0.00449 | Colicin/pyocin immunity protein |
| PAU_03903 | 77.04 | 6.64 | -3.5357 | 0.00004 | Colicin/pyocin protein |
| PAU_03904 | 10.88 | 0.68 | -3.9943 | 0.00412 | Colicin/pyocin structural protein |
| ***Sensing and regulation*** | | | | | |
| PAU_00726 | 64.24 | 15.56 | -2.0456 | 0.03991 | MrfB/PapH-like, fimbrial regulatory protein |
| *PAU_00865* | 443.69 | 92.13 | -2.2677 | 0.00273 | PAS4/LuxR family transcriptional regulator |
| *PAU_00866* | 392.01 | 84.97 | -2.2059 | 0.00282 | PAS4/LuxR family transcriptional regulator |
| PAU_00867 | 1475.26 | 295.69 | -2.3188 | 0.00181 | PAS4/LuxR family transcriptional regulator |
| PAU_01443 | 1758.85 | 133.33 | -3.7216 | 0.00018 | TrbH domain containing protein, cojugative transfer protein family |
| *PAU_01444* | 771.57 | 83.20 | -3.2132 | 0.00003 | Putative aminoacyl tRNA synthetase anti-codon binding domain-like protein |
| PAU_01445 | 168.52 | 21.25 | -2.9876 | 0.00016 | TctD, two-component response regulator |
| PAU_01446 | 115.01 | 20.03 | -2.5215 | 0.00129 | TctE, two-component sensor protein |
| PAU_02587 | 84.18 | 5.13 | -4.0377 | 0.05515 | LuxR family transcriptional regulator |
| PAU_02588 | 179.49 | 3.26 | -5.7818 | 0.02607 | LuxR/PAS domain transcriptional regulator |
| PAU_03778 | 608.71 | 64.28 | -3.2433 | 0.00003 | LysR-type transcriptional regulator protein |
| *PAU_03919* | 3182.65 | 313.37 | -3.3443 | 0.03546 | Ner-like regulator (sugar fermentation stimulation protein B) |
| *PAU_03939* | 29.75 | 6.58 | -2.1772 | 0.01554 | FecI, RNA polymerase iron stimulated ECF sigma factor |
| ***Secondary metabolite production and putative quorum sensing*** | | | | | |
| *PAU_03374* | 20.65 | 4.36 | -2.2428 | 0.01757 | NRPS domain protein |
| *PAU_03375* | 53.02 | 2.28 | -4.5388 | 0.00379 | NRPS domain protein |
| *PAU_03767* | 2544.60 | 16.33 | -7.2841 | 0.02932 | Hypothetical protein in putative QS operon |
| *PAU_03768* | 1474.89 | 10.58 | -7.1226 | 0.02546 | AfsA family domain protein for A-factor biosynthesis, essential for streptomycin production and resistance in putative QS operon |
| *PAU_03769* | 1823.22 | 21.81 | -6.3853 | 0.00250 | Hypothetical protein in putative QS operon |
| *PAU_03770* | 1778.11 | 42.42 | -5.3895 | <.00001 | Hypothetical haloacid dehalogenase-like hydrolase in putative QS operon |
| *PAU_03771* | 1741.61 | 31.39 | -5.7941 | 0.00258 | Carboxy-muconolactone decarboxylase family protein in putative QS operon |
| *PAU_03772* | 461.19 | 11.42 | -5.3352 | 0.00015 | Probable acetyl-CoA acetyltransferase in putative QS operon |
| ***Iron acquisition*** | | | | | |
| PAU_03941 | 48.36 | 4.75 | -3.3491 | 0.00017 | FecA, iron(iii) dicitrate outer membrane transporter |
| PAU_03942 | 24.76 | 3.81 | -2.6984 | 0.00449 | FecB, iron(iii) dicitrate-binding periplasmic protein |
| PAU_03944 | 10.46 | 2.03 | -2.3647 | 0.03779 | FecD, iron(iii) dicitrate-transporter subunit |
| PAU_03945 | 15.55 | 1.73 | -3.1662 | 0.00357 | FecE, iron(iii) dicitrate-transporter ATP-binding subunit |
| ***Stress response*** | | | | | |
| PAU_02695 | 2030.66 | 274.28 | -2.8882 | 0.03444 | CspC-like, cold shock protein |
| PAU_04005 | 438.45 | 31.57 | -3.7959 | 0.04912 | BhsA-like *Xenorhabdus* multiple stress resistance protein |
| PAU_04022 | 199.33 | 6.51 | -4.9357 | <.00001 | CstA, carbon starvation membrane protein for peptide utilization during carbon starvation |
| ***Other transporters*** | | | | | |
| PAU_01440 | 299.02 | 10.10 | -4.8872 | <.00001 | TctA, tripartite tricarboxylate transporter |
| PAU_01441 | 489.02 | 10.91 | -5.4855 | <.00001 | TctB, tripartite tricarboxylate transporter |
| PAU_01442 | 2214.58 | 54.44 | -5.3462 | <.00001 | TctC, tripartite tricarboxylate transporter |
| PAU_02764 | 66.89 | 6.65 | -3.3312 | 0.00063 | OmpN, outer membrane porin protein |
| ***Information processing*** | | | | | |
| PAU_00707 | 85.98 | 20.67 | -2.0565 | 0.00987 | CRISPR associated protein, Cse1 family |
| PAU_00708 | 61.23 | 13.62 | -2.1686 | 0.00738 | CRISPR-associated protein, Cse2 family |
| PAU_00709 | 41.80 | 10.69 | -1.9670 | 0.01597 | CRISPR-associated protein, Cse4 family |
| ***Mobile elements and unknown function*** | | | | | |
| PAU_00934 | 69.60 | 13.45 | -2.3716 | 0.06580 | Predicted secreted hypothetical protein |
| *PAU_00995* | 49.74 | 11.28 | -2.1411 | 0.06104 | Hypothetical protein |
| PAU_01252 | 17.34 | 4.48 | -1.9527 | 0.05278 | Hypothetical protein |
| PAU_01647 | 239.43 | 24.55 | -3.2858 | 0.00003 | Putative phage tail fibre assembly protein |
| PAU_01648 | 57.71 | 5.75 | -3.3283 | 0.01205 | Phage tail fibre protein |
| PAU_01649 | 46.87 | 4.31 | -3.4419 | 0.00014 | Phage tail fibre protein |
| PAU_01650 | 29.04 | 3.86 | -2.9109 | 0.00212 | Phage tail fibre protein |
| PAU_01651 | 120.97 | 20.77 | -2.5419 | 0.00341 | Phage tail fibre protein |
| *PAU_03086* | 971.72 | 176.23 | -2.4631 | 0.00098 | Hypothetical protein |
| *PAU_03087* | 382.96 | 44.66 | -3.1002 | 0.00006 | Hypothetical protein |
| *PAU_03513* | 1053.05 | 72.22 | -3.8660 | 0.00492 | Predicted secreted lipoprotein |
| *PAU_03897* | 235.66 | 36.60 | -2.6869 | 0.00047 | Predicted secreted lipoprotein |
| *PAU_03898* | 685.28 | 137.02 | -2.3223 | 0.00158 | Hypothetical protein |
| *PAU_04004* | 411.69 | 88.84 | -2.2123 | 0.00273 | Hypothetical protein |
