# Supplementary material for: From Insect to Man: Photorhabdus Sheds Light on the Emergence of Human Pathogenicity
Source: PLoS One. 2015 Dec 17;10(12):e0144937. doi: 10.1371/journal.pone.0144937 (PMC4683029; doi:10.1371/journal.pone.0144937)
Supplement: S3 Table — (DOCX) [file pone.0144937.s018.docx]

**Table S3.** **Proteomic changes.** Significantly differentially expressed *P. asymbiotica* ^ATCC43939^ cellular proteins expressed in exponential growth phase at 28°C and 37°C in rich medium (LB). Negative protein fold changes, shown in green, are those more abundant at 28°C (MalE to AspC) and therefore more relevant to insect infection. Positive fold changes shown in red (DnaK to PAU_03286) are more abundant at 37°C and therefore more relevant to a human infection.

| **Gene** | **Protein** | **Fold change** | **Notes** |
| --- | --- | --- | --- |
| PAU_00367 | MalE | -3.31 | Maltose-binding periplasmic protein ABC transporter |
| PAU_00083 | PckA | -2.96 | Phosphoenolpyruvate carboxykinase |
| PAU_01826 | GuaB | -2.75 | Inosine-5'-monophosphate dehydrogenase |
| PAU_00406 | PurH | -2.65 | Bifunctional purine biosynthesis protein |
| PAU_01827 | GuaA | -2.55 | Glutamine-hydrolyzing GMP synthase |
| PAU_01242 | MetY | -2.55 | o-acetylhomoserine sulfhydrylase |
| PAU_02896 | SfcA | -2.36 | NAD-dependent malic enzyme |
| PAU_01159 | GcvT | -2.36 | Aminomethyltransferase of glycine cleavage system |
| PAU_01877 | ArnA | -2.34 | Bifunctional polymyxin resistance protein |
| PAU_01324 | PurL | -2.26 | Phospho-ribosylformylglycinamidine synthase. |
| PAU_03499 | PAU_03499 | -1.98 | Gamma-glutamyltranspeptidase. |
| PAU_00969 | VgrG | -1.86 | Type VI secretion system |
| PAU_01687 | PAU_01687 | -1.85 | Putative iron-regulated outer membrane protein |
| PAU_02443 | Zwf | -1.82 | Glucose-6-phosphate 1-dehydrogenase |
| PAU_02766 | AspC | -1.8 | Aspartate aminotransferase |
| PAU_00543 | DnaK | 1.90 | Hsp70, heat shock Hsp70, heat shock chaperone |
| PAU_03212 | RecA | 1.81 | Recombination protein |
| PAU_02173 | PAU_02173 | 1.86 | Conserved hypothetical protein |
| PAU_03263 | PAU_03263 | 2.05 | Conserved hypothetical protein |
| PAU_03757 | GroS | 2.14 | GroES 10 KDa chaperone |
| PAU_01940 | Gst | 2.19 | Glutathione S-transferase |
| PAU_02575 | LuxR | 2.33 | Transcriptional regulator |
| PAU_02834 | AnsB | 2.33 | Asparaginase II |
| PAU_03756 | GroL | 2.37 | 60 KDa chaperonin |
| PAU_03260 | GmhA | 2.37 | Phosphoheptose isomerase |
| PAU_01793 | Bcp | 2.43 | Bacterioferritin comigrate protein |
| PAU_03190 | ClpB | 3.17 | Disaggregation chaperone |
| PAU_01469 | LsrF | 3.35 | Autoinducer (AI-2) aldolase |
| PAU_01824 | CjrC | 3.70 | Similar to outer membrane siderophore receptor |
| PAU_03286 | PAU_03286 | 4.89 | Iron compound ABC transporter |
