# Supplementary material for: From Insect to Man: Photorhabdus Sheds Light on the Emergence of Human Pathogenicity
Source: PLoS One. 2015 Dec 17;10(12):e0144937. doi: 10.1371/journal.pone.0144937 (PMC4683029; doi:10.1371/journal.pone.0144937)
Supplement: S4 Table — (DOCX) [file pone.0144937.s019.docx]

**Table S4. Carbon source utilization enabling respiration for *P. asymbiotica* ^ATCC43949^ (*Pa*) and *P. luminescens* ^TT01^ (*Pl*) at 28°C and 37°C.** Data from Biolog plate PM01 in which the bacteria were grown in M9 supplemented with 0.05% (w/v) CAS amino acids (as a mixed nitrogen source) and reporter DyeA was used (Biolog). The addition of a vitamin mix was also necessary at 37°C. A binary classification is used where 1 is positive and zero is negative. A classification of 1 is given if the respiration curve of the test sample rises above the upper limit of that of the relevant control cell curve. Cells in yellow indicate that the bacteria could use the source for at least limited respiration to a level above the background level of the negative control well. Cells in green indicate were high levels of respiration were observed. Those in red show ability to use the carbon source in all cases.

| **Plate, well** | **Carbon source** | ***Pa* 28**°C | ***Pa* 37**°C | ***Pl* 28**°C |
| --- | --- | --- | --- | --- |
| PM01,A01 | Negative Control | 0 | 0 | 0 |
| PM01,A02 | L-Arabinose | 0 | 0 | 0 |
| PM01,A03 | N-Acetyl-D-Glucosamine | 1 | 1 | 1 |
| PM01,A04 | D-Saccharic Acid | 0 | 0 | 0 |
| PM01,A05 | Succinic Acid | 0 | 0 | 0 |
| PM01,A06 | D-Galactose | 0 | 0 | 0 |
| PM01,A07 | L-Aspartic Acid | 1 | 0 | 0 |
| PM01,A08 | L-Proline | 1 | 0 | 0 |
| PM01,A09 | D-Alanine | 0 | 0 | 0 |
| PM01,A10 | D-Trehalose | 1 | 0 | 0 |
| PM01,A11 | D-Mannose | 1 | 1 | 1 |
| PM01,A12 | Dulcitol | 0 | 0 | 0 |
| PM01,B01 | D-Serine | 0 | 0 | 0 |
| PM01,B02 | D-Sorbitol | 0 | 0 | 0 |
| PM01,B03 | Glycerol | 1 | 0 | 1 |
| PM01,B04 | L-Fucose | 0 | 0 | 0 |
| PM01,B05 | D-Glucuronic Acid | 1 | 0 | 1 |
| PM01,B06 | D-Gluconic Acid | 0 | 0 | 0 |
| PM01,B07 | D,L-a-Glycerol- Phosphate | 1 | 0 | 1 |
| PM01,B08 | D-Xylose | 0 | 0 | 0 |
| PM01,B09 | L-Lactic Acid | 0 | 0 | 0 |
| PM01,B10 | Formic Acid | 0 | 0 | 0 |
| PM01,B11 | D-Mannitol | 0 | 0 | 0 |
| PM01,B12 | L-Glutamic Acid | 1 | 0 | 0 |
| PM01,C01 | D-Glucose-6-Phosphate | 0 | 0 | 0 |
| PM01,C02 | D-Galactonic Acid-g-Lactone | 0 | 0 | 0 |
| PM01,C03 | D,L-Malic Acid | 1 | 0 | 0 |
| PM01,C04 | D-Ribose | 0 | 0 | 0 |
| PM01,C05 | Tween 20 | 1 | 0 | 0 |
| PM01,C06 | L-Rhamnose | 0 | 0 | 0 |
| PM01,C07 | D-Fructose | 1 | 0 | 1 |
| PM01,C08 | Acetic Acid | 0 | 0 | 0 |
| PM01,C09 | a-D-Glucose | 1 | 0 | 1 |
| PM01,C10 | Maltose | 1 | 0 | 1 |
| PM01,C11 | D-Melibiose | 0 | 0 | 0 |
| PM01,C12 | Thymidine | 0 | 0 | 0 |
| PM01,D01 | L-Asparagine | 1 | 0 | 1 |
| PM01,D02 | D-Aspartic Acid | 0 | 0 | 0 |
| PM01,D03 | D-Glucosaminic Acid | 0 | 0 | 0 |
| PM01,D04 | 1,2-Propanediol | 0 | 0 | 0 |
| PM01,D05 | Tween 40 | 0 | 0 | 0 |
| PM01,D06 | a-Keto-Glutaric Acid | 1 | 0 | 0 |
| PM01,D07 | a-Keto-Butyric Acid | 1 | 0 | 0 |
| PM01,D08 | a-Methyl-D-Galactoside | 0 | 0 | 0 |
| PM01,D09 | a-D-Lactose | 0 | 0 | 0 |
| PM01,D10 | Lactulose | 0 | 0 | 0 |
| PM01,D11 | Sucrose | 0 | 0 | 0 |
| PM01,D12 | Uridine | 1 | 1 | 1 |
| PM01,E01 | L-Glutamine | 1 | 0 | 1 |
| PM01,E02 | m-Tartaric Acid | 0 | 0 | 0 |
| PM01,E03 | D-Glucose-1-Phosphate | 0 | 0 | 0 |
| PM01,E04 | D-Fructose-6-Phosphate | 0 | 0 | 0 |
| PM01,E05 | Tween 80 | 1 | 0 | 1 |
| PM01,E06 | a-Hydroxy Glutaric Acid-g-Lactone | 0 | 0 | 0 |
| PM01,E07 | a-Hydroxy-Butyric Acid | 0 | 0 | 0 |
| PM01,E08 | b-Methyl-D-Glucoside | 0 | 0 | 0 |
| PM01,E09 | Adonitol | 0 | 0 | 0 |
| PM01,E10 | Maltotriose | 1 | 0 | 1 |
| PM01,E11 | 2`-Deoxy-Adenosine | 0 | 0 | 0 |
| PM01,E12 | Adenosine | 1 | 1 | 1 |
| PM01,F01 | Glycyl-L-Aspartic Acid | 0 | 0 | 0 |
| PM01,F02 | Citric Acid | 1 | 0 | 1 |
| PM01,F03 | m-Inositol | 1 | 0 | 1 |
| PM01,F04 | D-Threonine | 0 | 0 | 0 |
| PM01,F05 | Fumaric Acid | 1 | 0 | 1 |
| PM01,F06 | Bromo-Succinic Acid | 1 | 0 | 0 |
| PM01,F07 | Propionic Acid | 0 | 0 | 0 |
| PM01,F08 | Mucic Acid | 0 | 0 | 0 |
| PM01,F09 | Glycolic Acid | 0 | 0 | 0 |
| PM01,F10 | Glyoxylic Acid | 0 | 0 | 0 |
| PM01,F11 | D-Cellobiose | 0 | 0 | 0 |
| PM01,F12 | Inosine | 1 | 1 | 1 |
| PM01,G01 | Glycyl-L-Glutamic Acid | 0 | 0 | 0 |
| PM01,G02 | Tricarballylic Acid | 0 | 0 | 0 |
| PM01,G03 | L-Serine | 1 | 1 | 1 |
| PM01,G04 | L-Threonine | 1 | 0 | 1 |
| PM01,G05 | L-Alanine | 1 | 0 | 1 |
| PM01,G06 | L-Alanyl-Glycine | 1 | 0 | 1 |
| PM01,G07 | Acetoacetic Acid | 0 | 0 | 0 |
| PM01,G08 | N-Acetyl-b-D-Mannosamine | 0 | 0 | 0 |
| PM01,G09 | Mono Methyl Succinate | 0 | 0 | 0 |
| PM01,G10 | Methyl Pyruvate | 1 | 0 | 1 |
| PM01,G11 | D-Malic Acid | 0 | 0 | 0 |
| PM01,G12 | L-Malic Acid | 1 | 0 | 1 |
| PM01,H01 | Glycyl-L-Proline | 1 | 1 | 1 |
| PM01,H02 | p-Hydroxy-Phenylacetic Acid | 0 | 0 | 0 |
| PM01,H03 | m-Hydroxy-Phenylacetic Acid | 0 | 0 | 0 |
| PM01,H04 | Tyramine | 1 | 0 | 1 |
| PM01,H05 | D-Psicose | 0 | 0 | 0 |
| PM01,H06 | L-Lyxose | 0 | 0 | 0 |
| PM01,H07 | Glucuronamide | 0 | 0 | 0 |
| PM01,H08 | Pyruvic Acid | 1 | 0 | 1 |
| PM01,H09 | L-Galactonic Acid-g-Lactone | 0 | 0 | 0 |
| PM01,H10 | D-Galacturonic Acid | 0 | 0 | 0 |
| PM01,H11 | b-Phenylethylamine | 0 | 0 | 0 |
| PM01,H12 | Ethanolamine | 0 | 0 | 0 |
