# Supplementary material for: From Insect to Man: Photorhabdus Sheds Light on the Emergence of Human Pathogenicity
Source: PLoS One. 2015 Dec 17;10(12):e0144937. doi: 10.1371/journal.pone.0144937 (PMC4683029; doi:10.1371/journal.pone.0144937)
Supplement: S5 Table — (DOCX) [file pone.0144937.s020.docx]

**Table S5. Carbon source utilization enabling respiration for *P. asymbiotica* ^ATCC43949^ (*Pa*) and *P. luminescens* ^TT01^ (*Pl*) at 28°C and 37°C.** Data from Biolog plate PM02 in which the bacteria were grown in M9 supplemented with 0.05% (w/v) CAS amino acids (as a mixed nitrogen source) and reporter DyeA was used (Biolog). The addition of a vitamin mix was also necessary at 37°C. A binary classification is used where 1 is positive and zero is negative. A classification of 1 is given if the respiration curve of the test sample rises above the upper limit of that of the relevant control cell curve. Cells in yellow indicate that the bacteria could use the source for at least limited respiration to a level above the background level of the negative control well. Cells in green indicate were high levels of respiration were observed.

| **Plate, well** | **Carbon source** | ***Pa* 28°C** | ***Pa* 37°C** | ***Pl* 28°C** |
| --- | --- | --- | --- | --- |
| PM02A,A01 | Negative Control | 0 | 0 | 0 |
| PM02A,A02 | Chondroitin Sulfate C | 0 | 0 | 0 |
| PM02A,A03 | a-Cyclodextrin | 0 | 0 | 0 |
| PM02A,A04 | b-Cyclodextrin | 0 | 0 | 0 |
| PM02A,A05 | g-Cyclodextrin | 0 | 0 | 0 |
| PM02A,A06 | Dextrin | 1 | 0 | 1 |
| PM02A,A07 | Gelatin | 0 | 0 | 0 |
| PM02A,A08 | Glycogen | 0 | 0 | 0 |
| PM02A,A09 | Inulin | 0 | 0 | 0 |
| PM02A,A10 | Laminarin | 0 | 0 | 0 |
| PM02A,A11 | Mannan | 0 | 0 | 0 |
| PM02A,A12 | Pectin | 0 | 0 | 0 |
| PM02A,B01 | N-Acetyl-D-Galactosamine | 1 | 0 | 1 |
| PM02A,B02 | N-Acetyl-Neuraminic Acid | 0 | 0 | 0 |
| PM02A,B03 | b-D-Allose | 0 | 0 | 0 |
| PM02A,B04 | Amygdalin | 0 | 0 | 0 |
| PM02A,B05 | D-Arabinose | 0 | 0 | 0 |
| PM02A,B06 | D-Arabitol | 0 | 0 | 0 |
| PM02A,B07 | L-Arabitol | 0 | 0 | 0 |
| PM02A,B08 | Arbutin | 0 | 0 | 0 |
| PM02A,B09 | 2-Deoxy-D-Ribose | 0 | 0 | 0 |
| PM02A,B10 | i-Erythritol | 0 | 0 | 0 |
| PM02A,B11 | D-Fucose | 0 | 0 | 0 |
| PM02A,B12 | 3-0-b-D-Galactopyranosyl-D-Arabinose | 0 | 0 | 0 |
| PM02A,C01 | Gentiobiose | 0 | 0 | 0 |
| PM02A,C02 | L-Glucose | 0 | 0 | 0 |
| PM02A,C03 | D-Lactitol | 0 | 0 | 0 |
| PM02A,C04 | D-Melezitose | 0 | 0 | 0 |
| PM02A,C05 | Maltitol | 0 | 0 | 0 |
| PM02A,C06 | a-Methyl-D-Glucoside | 0 | 0 | 0 |
| PM02A,C07 | b-Methyl-D-Galactoside | 0 | 0 | 0 |
| PM02A,C08 | 3-O-Methyl-Glucose | 1 | 0 | 0 |
| PM02A,C09 | b-Methyl-D-Glucuronic Acid | 0 | 0 | 0 |
| PM02A,C10 | a-Methyl-D-Mannoside | 0 | 0 | 0 |
| PM02A,C11 | b-Methyl-D-Xyloside | 0 | 0 | 0 |
| PM02A,C12 | Palatinose | 0 | 0 | 0 |
| PM02A,D01 | D-Raffinose | 0 | 0 | 0 |
| PM02A,D02 | Salicin | 0 | 0 | 0 |
| PM02A,D03 | Sedoheptulosan | 0 | 0 | 0 |
| PM02A,D04 | L-Sorbose | 0 | 0 | 0 |
| PM02A,D05 | Stachyose | 0 | 0 | 0 |
| PM02A,D06 | D-Tagatose | 0 | 0 | 0 |
| PM02A,D07 | Turanose | 0 | 0 | 0 |
| PM02A,D08 | Xylitol | 0 | 0 | 1 |
| PM02A,D09 | N-Acetyl-D-Glucosaminitol | 0 | 0 | 0 |
| PM02A,D10 | g-Amino-Butyric Acid | 0 | 0 | 0 |
| PM02A,D11 | d-Amino-Valeric Acid | 0 | 0 | 0 |
| PM02A,D12 | Butyric Acid | 0 | 0 | 0 |
| PM02A,E01 | Capric Acid | 0 | 0 | 0 |
| PM02A,E02 | Caproic Acid | 0 | 0 | 0 |
| PM02A,E03 | Citraconic Acid | 0 | 0 | 0 |
| PM02A,E04 | D,L-Citramalic Acid | 0 | 0 | 0 |
| PM02A,E05 | D-Glucosamine | 0 | 0 | 0 |
| PM02A,E06 | 2-Hydroxy-Benzoic Acid | 0 | 0 | 0 |
| PM02A,E07 | 4-Hydroxy-Benzoic Acid | 0 | 0 | 0 |
| PM02A,E08 | b-Hydroxy-Butyric Acid | 0 | 0 | 0 |
| PM02A,E09 | g-Hydroxy-Butyric Acid | 0 | 0 | 0 |
| PM02A,E10 | a-Keto-Valeric Acid | 0 | 0 | 0 |
| PM02A,E11 | Itaconic Acid | 0 | 0 | 0 |
| PM02A,E12 | 5-Keto-D-Gluconic Acid | 0 | 0 | 0 |
| PM02A,F01 | D-Lactic Acid Methyl Ester | 0 | 0 | 0 |
| PM02A,F02 | Malonic Acid | 0 | 0 | 0 |
| PM02A,F03 | Melibionic Acid | 0 | 0 | 0 |
| PM02A,F04 | Oxalic Acid | 0 | 0 | 0 |
| PM02A,F05 | Oxalomalic Acid | 0 | 0 | 0 |
| PM02A,F06 | Quinic Acid | 0 | 0 | 0 |
| PM02A,F07 | D-Ribono-1,4-Lactone | 0 | 0 | 0 |
| PM02A,F08 | Sebacic Acid | 0 | 0 | 0 |
| PM02A,F09 | Sorbic Acid | 0 | 0 | 0 |
| PM02A,F10 | Succinamic Acid | 0 | 0 | 0 |
| PM02A,F11 | D-Tartaric Acid | 0 | 0 | 0 |
| PM02A,F12 | L-Tartaric Acid | 0 | 0 | 0 |
| PM02A,G01 | Acetamide | 0 | 0 | 0 |
| PM02A,G02 | L-Alaninamide | 0 | 0 | 0 |
| PM02A,G03 | N-Acetyl-L-Glutamic Acid | 0 | 0 | 0 |
| PM02A,G04 | L-Arginine | 0 | 0 | 0 |
| PM02A,G05 | Glycine | 0 | 0 | 0 |
| PM02A,G06 | L-Histidine | 1 | 0 | 0 |
| PM02A,G07 | L-Homoserine | 0 | 0 | 0 |
| PM02A,G08 | 4-Hydroxy-L-Proline (trans) | 1 | 0 | 0 |
| PM02A,G09 | L-Isoleucine | 0 | 0 | 0 |
| PM02A,G10 | L-Leucine | 0 | 0 | 0 |
| PM02A,G11 | L-Lysine | 0 | 0 | 0 |
| PM02A,G12 | L-Methionine | 0 | 0 | 0 |
| PM02A,H01 | L-Ornithine | 0 | 0 | 0 |
| PM02A,H02 | L-Phenylalanine | 0 | 0 | 0 |
| PM02A,H03 | L-Pyroglutamic Acid | 0 | 0 | 0 |
| PM02A,H04 | L-Valine | 0 | 0 | 0 |
| PM02A,H05 | D,L-Carnitine | 0 | 0 | 0 |
| PM02A,H06 | Butylamine (sec) | 0 | 0 | 0 |
| PM02A,H07 | D,L-Octopamine | 0 | 0 | 0 |
| PM02A,H08 | Putrescine | 0 | 0 | 0 |
| PM02A,H09 | Dihydroxy-Acetone | 0 | 0 | 0 |
| PM02A,H10 | 2,3-Butanediol | 0 | 0 | 0 |
| PM02A,H11 | 2,3-Butanone | 0 | 0 | 0 |
| PM02A,H12 | 3-Hydroxy-2-Butanone | 0 | 0 | 0 |
