# Supplementary material for: From Insect to Man: Photorhabdus Sheds Light on the Emergence of Human Pathogenicity
Source: PLoS One. 2015 Dec 17;10(12):e0144937. doi: 10.1371/journal.pone.0144937 (PMC4683029; doi:10.1371/journal.pone.0144937)
Supplement: S6 Table — (DOCX) [file pone.0144937.s021.docx]

**Table S6. Nitrogen source utilization enabling respiration for *P. asymbiotica* ^ATCC43949^ (*Pa*) and *P. luminescens* ^TT01^ (*Pl*) at 28°C and 37°C.** Data from Biolog plate PM03 in which the bacteria were grown in M9 supplemented with 20 mM mannose (as a carbon source) with 1x RPMI vitamins at 37°C. A binary classification is used where 1 is positive and zero is negative. A classification of 1 is given if the respiration curve of the test sample rises above the upper limit of that of the relevant control cell curve. Cells in yellow indicate that the bacteria could use the source for at least limited respiration to a level above the background level of the negative control well. Cells in green indicate were high levels of respiration were observed. Those in red show ability to use the nitrogen source in all cases.

| **Plate, well** | **Nitrogen source** | ***Pa* 28°C** | ***Pa* 37°C** | ***Pl* 28°C** |
| --- | --- | --- | --- | --- |
| PM03B,A01 | Negative Control | 0 | 0 | 0 |
| PM03B,A02 | Ammonia | 0 | 0 | 0 |
| PM03B,A03 | Nitrite | 0 | 0 | 0 |
| PM03B,A04 | Nitrate | 0 | 0 | 0 |
| PM03B,A05 | Urea | 0 | 0 | 0 |
| PM03B,A06 | Biuret | 0 | 0 | 0 |
| PM03B,A07 | L-Alanine | 0 | 0 | 0 |
| PM03B,A08 | L-Arginine | 0 | 0 | 0 |
| PM03B,A09 | L-Asparagine | 0 | 1 | 0 |
| PM03B,A10 | L-Aspartic Acid | 0 | 1 | 0 |
| PM03B,A11 | L-Cysteine | 0 | 0 | 0 |
| PM03B,A12 | L-Glutamic Acid | 1 | 1 | 1 |
| PM03B,B01 | L-Glutamine | 1 | 1 | 1 |
| PM03B,B02 | Glycine | 0 | 0 | 0 |
| PM03B,B03 | L-Histidine | 0 | 0 | 0 |
| PM03B,B04 | L-Isoleucine | 0 | 0 | 0 |
| PM03B,B05 | L-Leucine | 0 | 0 | 0 |
| PM03B,B06 | L-Lysine | 0 | 0 | 0 |
| PM03B,B07 | L-Methionine | 0 | 0 | 0 |
| PM03B,B08 | L-Phenylalanine | 0 | 0 | 0 |
| PM03B,B09 | L-Proline | 0 | 0 | 0 |
| PM03B,B10 | L-Serine | 1 | 0 | 0 |
| PM03B,B11 | L-Threonine | 0 | 0 | 0 |
| PM03B,B12 | L-Tryptophan | 1 | 0 | 0 |
| PM03B,C01 | L-Tyrosine | 1 | 1 | 1 |
| PM03B,C02 | L-Valine | 0 | 0 | 0 |
| PM03B,C03 | D-Alanine | 0 | 0 | 0 |
| PM03B,C04 | D-Asparagine | 0 | 0 | 0 |
| PM03B,C05 | D-Aspartic Acid | 0 | 0 | 0 |
| PM03B,C06 | D-Glutamic Acid | 0 | 0 | 0 |
| PM03B,C07 | D-Lysine | 0 | 0 | 0 |
| PM03B,C08 | D-Serine | 0 | 0 | 0 |
| PM03B,C09 | D-Valine | 0 | 0 | 0 |
| PM03B,C10 | L-Citrulline | 0 | 0 | 0 |
| PM03B,C11 | L-Homoserine | 0 | 0 | 0 |
| PM03B,C12 | L-Ornithine | 0 | 0 | 0 |
| PM03B,D01 | N-Acetyl-D,L-Glutamic Acid | 0 | 0 | 0 |
| PM03B,D02 | N-Phthaloyl-L-Glutamic Acid | 0 | 0 | 0 |
| PM03B,D03 | L-Pyroglutamic Acid | 0 | 0 | 0 |
| PM03B,D04 | Hydroxylamine | 0 | 0 | 0 |
| PM03B,D05 | Methylamine | 0 | 0 | 0 |
| PM03B,D06 | N-Amylamine | 0 | 0 | 0 |
| PM03B,D07 | N-Butylamine | 0 | 0 | 0 |
| PM03B,D08 | Ethylamine | 0 | 0 | 0 |
| PM03B,D09 | Ethanolamine | 0 | 0 | 0 |
| PM03B,D10 | Ethylenediamine | 0 | 0 | 0 |
| PM03B,D11 | Putrescine | 0 | 0 | 0 |
| PM03B,D12 | Agmatine | 0 | 0 | 0 |
| PM03B,E01 | Histamine | 0 | 0 | 0 |
| PM03B,E02 | b-Phenylethylamine | 0 | 0 | 0 |
| PM03B,E03 | Tyramine | 0 | 0 | 0 |
| PM03B,E04 | Acetamide | 0 | 0 | 0 |
| PM03B,E05 | Formamide | 0 | 0 | 0 |
| PM03B,E06 | Glucuronamide | 0 | 0 | 0 |
| PM03B,E07 | D,L-Lactamide | 0 | 0 | 0 |
| PM03B,E08 | D-Glucosamine | 0 | 0 | 0 |
| PM03B,E09 | D-Galactosamine | 0 | 0 | 0 |
| PM03B,E10 | D-Mannosamine | 0 | 0 | 0 |
| PM03B,E11 | N-Acetyl-D-Glucosamine | 1 | 0 | 0 |
| PM03B,E12 | N-Acetyl-D-Galactosamine | 0 | 0 | 0 |
| PM03B,F01 | N-Acetyl-D-Mannosamine | 0 | 0 | 0 |
| PM03B,F02 | Adenine | 0 | 0 | 0 |
| PM03B,F03 | Adenosine | 1 | 0 | 0 |
| PM03B,F04 | Cytidine | 1 | 0 | 0 |
| PM03B,F05 | Cytosine | 0 | 0 | 0 |
| PM03B,F06 | Guanine | 0 | 0 | 0 |
| PM03B,F07 | Guanosine | 0 | 0 | 0 |
| PM03B,F08 | Thymine | 0 | 0 | 0 |
| PM03B,F09 | Thymidine | 0 | 0 | 0 |
| PM03B,F10 | Uracil | 0 | 0 | 0 |
| PM03B,F11 | Uridine | 0 | 0 | 0 |
| PM03B,F12 | Inosine | 0 | 0 | 0 |
| PM03B,G01 | Xanthine | 1 | 1 | 1 |
| PM03B,G02 | Xanthosine | 0 | 0 | 0 |
| PM03B,G03 | Uric Acid | 0 | 0 | 0 |
| PM03B,G04 | Alloxan | 0 | 0 | 0 |
| PM03B,G05 | Allantoin | 0 | 0 | 0 |
| PM03B,G06 | Parabanic Acid | 0 | 0 | 0 |
| PM03B,G07 | D,L-a-Amino-N-Butyric Acid | 0 | 0 | 0 |
| PM03B,G08 | g-Amino-N-Butyric Acid | 0 | 0 | 0 |
| PM03B,G09 | e-Amino-N-Caproic Acid | 0 | 0 | 0 |
| PM03B,G10 | D,L-a-Amino-Caprylic Acid | 0 | 0 | 0 |
| PM03B,G11 | d-Amino-N-Valeric Acid | 0 | 0 | 0 |
| PM03B,G12 | a-Amino-N-Valeric Acid | 0 | 0 | 0 |
| PM03B,H01 | Ala-Asp | 0 | 0 | 0 |
| PM03B,H02 | Ala-Gln | 1 | 1 | 1 |
| PM03B,H03 | Ala-Glu | 1 | 0 | 0 |
| PM03B,H04 | Ala-Gly | 0 | 0 | 0 |
| PM03B,H05 | Ala-His | 1 | 0 | 0 |
| PM03B,H06 | Ala-Leu | 0 | 0 | 0 |
| PM03B,H07 | Ala-Thr | 0 | 0 | 0 |
| PM03B,H08 | Gly-Asn | 0 | 0 | 0 |
| PM03B,H09 | Gly-Gln | 0 | 0 | 0 |
| PM03B,H10 | Gly-Glu | 0 | 0 | 0 |
| PM03B,H11 | Gly-Met | 0 | 0 | 0 |
| PM03B,H12 | Met-Ala | 0 | 0 | 0 |
