# Supplementary material for: From Insect to Man: Photorhabdus Sheds Light on the Emergence of Human Pathogenicity
Source: PLoS One. 2015 Dec 17;10(12):e0144937. doi: 10.1371/journal.pone.0144937 (PMC4683029; doi:10.1371/journal.pone.0144937)
Supplement: S10 Table — (DOCX) [file pone.0144937.s025.docx]

**Table S10. Osmolyte effect on respiration for *P. asymbiotica* ^ATCC43949^ (*Pa*) and *P. luminescens* ^TT01^ (*Pl*) at 28°C and 37°C.** Data from Biolog plate PM09 in which the bacteria were grown in a Biolog-specific rich media. Extent of respiration achieved in each well is classified as a index between 0 and 5, where 0 is no respiration and 5 is full respiration relative to the A1 control well. Conditions that show a clear reduction in osmolyte tolerance for *Pa* at 37°C are highlighted in yellow.

| **Plate, well** | **Osmolyte** | ***Pa* 28°C** | ***Pa* 37°C** | ***Pl* 28°C** |
| --- | --- | --- | --- | --- |
| PM09,A01 | 1% NaCl | 5 | 5 | 5 |
| PM09,A02 | 2% NaCl | 5 | 3 | 4 |
| PM09,A03 | 3% NaCl | 4 | 0 | 3 |
| PM09,A04 | 4% NaCl | 3 | 0 | 1 |
| PM09,A05 | 5% NaCl | 0 | 0 | 0 |
| PM09,A06 | 5.5% NaCl | 0 | 0 | 0 |
| PM09,A07 | 6% NaCl | 0 | 0 | 0 |
| PM09,A08 | 6.5% NaCl | 0 | 0 | 0 |
| PM09,A09 | 7% NaCl | 0 | 0 | 0 |
| PM09,A10 | 8% NaCl | 0 | 0 | 0 |
| PM09,A11 | 9% NaCl | 0 | 0 | 0 |
| PM09,A12 | 10% NaCl | 0 | 0 | 0 |
| PM09,B01 | 6% NaCl | 0 | 0 | 0 |
| PM09,B02 | 6% NaCl + Betaine | 0 | 0 | 0 |
| PM09,B03 | 6% NaCl + N-N Dimethyl Glycine | 0 | 0 | 0 |
| PM09,B04 | 6% NaCl + Sarcosine | 0 | 0 | 0 |
| PM09,B05 | 6% NaCl + DimethylSulphonyl Propionate | 0 | 0 | 0 |
| PM09,B06 | 6% NaCl + MOPS | 0 | 0 | 0 |
| PM09,B07 | 6% NaCl + Ectoine | 0 | 0 | 0 |
| PM09,B08 | 6% NaCl + Choline | 0 | 0 | 0 |
| PM09,B09 | 6% NaCl + Phosphorylcholine | 0 | 0 | 0 |
| PM09,B10 | 6% NaCl + Creatine | 0 | 0 | 0 |
| PM09,B11 | 6% NaCl + Creatinine | 0 | 0 | 0 |
| PM09,B12 | 6% NaCl + L-Carnitine | 0 | 0 | 0 |
| PM09,C01 | 6% NaCl + KCl | 0 | 0 | 0 |
| PM09,C02 | 6% NaCl + L-Proline | 0 | 0 | 0 |
| PM09,C03 | 6% NaCl + N-Acetyl-L-Glutamine | 0 | 0 | 0 |
| PM09,C04 | 6% NaCl + ?-Glutamic Acid | 0 | 0 | 0 |
| PM09,C05 | 6% NaCl + ?–Amino-N-Butyric Acid | 0 | 0 | 0 |
| PM09,C06 | 6% NaCl + Glutathione | 0 | 0 | 0 |
| PM09,C07 | 6% NaCl + Glycerol | 0 | 0 | 0 |
| PM09,C08 | 6% NaCl + Trehalose | 0 | 0 | 0 |
| PM09,C09 | 6% NaCl + Trimethylamine-N-Oxide | 0 | 0 | 0 |
| PM09,C10 | 6% NaCl + Trimethylamine | 0 | 0 | 0 |
| PM09,C11 | 6% NaCl + Octopine | 0 | 0 | 0 |
| PM09,C12 | 6% NaCl + Trigonelline | 0 | 0 | 0 |
| PM09,D01 | 3% Potassium Chloride | 5 | 3 | 5 |
| PM09,D02 | 4% Potassium Chloride | 5 | 0 | 4 |
| PM09,D03 | 5% Potassium Chloride | 4 | 0 | 3 |
| PM09,D04 | 6% Potassium Chloride | 2 | 0 | 0 |
| PM09,D05 | 2% Sodium Sulfate | 5 | 1 | 5 |
| PM09,D06 | 3% Sodium Sulfate | 5 | 0 | 5 |
| PM09,D07 | 4% Sodium Sulfate | 5 | 0 | 4 |
| PM09,D08 | 5% Sodium Sulfate | 5 | 0 | 4 |
| PM09,D09 | 5% Ethylene Glycol | 5 | 2 | 5 |
| PM09,D10 | 10% Ethylene Glycol | 5 | 3 | 5 |
| PM09,D11 | 15% Ethylene Glycol | 5 | 4 | 5 |
| PM09,D12 | 20% Ethylene Glycol | 5 | 5 | 5 |
| PM09,E01 | 1% Sodium Formate | 5 | 0 | 3 |
| PM09,E02 | 2% Sodium Formate | 5 | 0 | 0 |
| PM09,E03 | 3% Sodium Formate | 0 | 0 | 0 |
| PM09,E04 | 4% Sodium Formate | 0 | 0 | 0 |
| PM09,E05 | 5% Sodium Formate | 0 | 0 | 0 |
| PM09,E06 | 6% Sodium Formate | 0 | 0 | 0 |
| PM09,E07 | 2% Urea | 5 | 0 | 4 |
| PM09,E08 | 3% Urea | 5 | 0 | 3 |
| PM09,E09 | 4% Urea | 0 | 0 | 1 |
| PM09,E10 | 5% Urea | 0 | 0 | 0 |
| PM09,E11 | 6% Urea | 0 | 0 | 0 |
| PM09,E12 | 7% Urea | 0 | 0 | 0 |
| PM09,F01 | 1% Sodium Lactate | 5 | 0 | 5 |
| PM09,F02 | 2% Sodium Lactate | 4 | 1 | 3 |
| PM09,F03 | 3% Sodium Lactate | 0 | 0 | 0 |
| PM09,F04 | 4% Sodium Lactate | 0 | 0 | 0 |
| PM09,F05 | 5% Sodium Lactate | 0 | 0 | 0 |
| PM09,F06 | 6% Sodium Lactate | 0 | 0 | 0 |
| PM09,F07 | 7% Sodium Lactate | 0 | 0 | 0 |
| PM09,F08 | 8% Sodium Lactate | 0 | 0 | 0 |
| PM09,F09 | 9% Sodium Lactate | 0 | 0 | 0 |
| PM09,F10 | 10% Sodium Lactate | 0 | 0 | 0 |
| PM09,F11 | 11% Sodium Lactate | 0 | 0 | 0 |
| PM09,F12 | 12% Sodium Lactate | 0 | 0 | 0 |
| PM09,G01 | 20mM Sodium Phosphate pH 7 | 5 | 4 | 5 |
| PM09,G02 | 50mM Sodium Phosphate pH 7 | 5 | 4 | 5 |
| PM09,G03 | 100mM Sodium Phosphate pH 7 | 5 | 4 | 5 |
| PM09,G04 | 200mM Sodium Phosphate pH 7 | 5 | 3 | 5 |
| PM09,G05 | 20mM Sodium Benzoate pH 5.2 | 3 | 0 | 2 |
| PM09,G06 | 50mM Sodium Benzoate pH 5.2 | 0 | 0 | 0 |
| PM09,G07 | 100mM Sodium Benzoate pH 5.2 | 0 | 0 | 0 |
| PM09,G08 | 200mM Sodium Benzoate pH 5.2 | 0 | 0 | 0 |
| PM09,G09 | 10mM Ammonium Sulfate pH 8 | 5 | 3 | 5 |
| PM09,G10 | 20mM Ammonium Sulfate pH 8 | 5 | 4 | 5 |
| PM09,G11 | 50mM Ammonium Sulfate pH 8 | 5 | 3 | 5 |
| PM09,G12 | 100mM Ammonium Sulfate pH 8 | 5 | 2 | 5 |
| PM09,H01 | 10mM Sodium Nitrate | 5 | 3 | 5 |
| PM09,H02 | 20mM Sodium Nitrate | 5 | 3 | 5 |
| PM09,H03 | 40mM Sodium Nitrate | 5 | 3 | 5 |
| PM09,H04 | 60mM Sodium Nitrate | 5 | 3 | 5 |
| PM09,H05 | 80mM Sodium Nitrate | 5 | 3 | 5 |
| PM09,H06 | 100mM Sodium Nitrate | 5 | 2 | 5 |
| PM09,H07 | 10mM Sodium Nitrite | 5 | 0 | 5 |
| PM09,H08 | 20mM Sodium Nitrite | 5 | 0 | 5 |
| PM09,H09 | 40mM Sodium Nitrite | 5 | 0 | 4 |
| PM09,H10 | 60mM Sodium Nitrite | 4 | 0 | 1 |
| PM09,H11 | 80mM Sodium Nitrite | 3 | 0 | 0 |
| PM09,H12 | 100mM Sodium Nitrite | 1 | 0 | 0 |
|  |  | 0 | 0 | 0 |
