# Supplementary material for: From Insect to Man: Photorhabdus Sheds Light on the Emergence of Human Pathogenicity
Source: PLoS One. 2015 Dec 17;10(12):e0144937. doi: 10.1371/journal.pone.0144937 (PMC4683029; doi:10.1371/journal.pone.0144937)
Supplement: S11 Table — (DOCX) [file pone.0144937.s026.docx]

**Table S11. pH effect on respiration for *P. asymbiotica* ^ATCC43949^ (*Pa*) and *P. luminescens* ^TT01^ (*Pl*) at 28°C and 37°C.** Data from Biolog plate PM10 in which the bacteria were grown in a Biolog-specific rich media. Extent of respiration achieved in each well is classified as a index between 0 and 5, where 0 is no respiration and 5 is full respiration relative to the A1 control well. Conditions that show a clear reduction in pH tolerance for *Pa* at 37°C are highlighted in yellow.

| **Plate, well** | **Osmolyte** | ***Pa* 28°C** | ***Pa* 37°C** | ***Pl* 28°C** |
| --- | --- | --- | --- | --- |
| PM10,A01 | pH 3.5 | 0 | 0 | 0 |
| PM10,A02 | pH 4 | 0 | 0 | 0 |
| PM10,A03 | pH 4.5 | 0 | 0 | 0 |
| PM10,A04 | pH 5 | 5 | 3 | 5 |
| PM10,A05 | pH 5.5 | 5 | 4 | 5 |
| PM10,A06 | pH 6 | 5 | 4 | 5 |
| PM10,A07 | pH 7 | 5 | 4 | 5 |
| PM10,A08 | pH 8 | 5 | 3 | 4 |
| PM10,A09 | pH 8.5 | 4 | 1 | 4 |
| PM10,A10 | pH 9 | 0 | 0 | 3 |
| PM10,A11 | pH 9.5 | 3 | 0 | 3 |
| PM10,A12 | pH 10 | 2 | 0 | 1 |
| PM10,B01 | pH 4.5 | 0 | 0 | 0 |
| PM10,B02 | pH 4.5 + L-Alanine | 0 | 0 | 0 |
| PM10,B03 | pH 4.5 + L-Arginine | 3 | 0 | 5 |
| PM10,B04 | pH 4.5 + L-Asparagine | 0 | 0 | 0 |
| PM10,B05 | pH 4.5 + L-Aspartic Acid | 2 | 0 | 2 |
| PM10,B06 | pH 4.5 + L-Glutamic Acid | 0 | 0 | 0 |
| PM10,B07 | pH 4.5 + L-Glutamine | 0 | 0 | 0 |
| PM10,B08 | pH 4.5 + Glycine | 5 | 1 | 4 |
| PM10,B09 | pH 4.5 + L-Histidine | 3 | 0 | 1 |
| PM10,B10 | pH 4.5 + L-Isoleucine | 0 | 0 | 0 |
| PM10,B11 | pH 4.5 + L-Leucine | 0 | 0 | 0 |
| PM10,B12 | pH 4.5 + L-Lysine | 0 | 0 | 0 |
| PM10,C01 | pH 4.5 + L-Methionine | 0 | 0 | 0 |
| PM10,C02 | pH 4.5 + L-Phenylalanine | 0 | 0 | 0 |
| PM10,C03 | pH 4.5 + L-Proline | 0 | 0 | 0 |
| PM10,C04 | pH 4.5 + L-Serine | 0 | 0 | 0 |
| PM10,C05 | pH 4.5 + L-Threonine | 0 | 0 | 0 |
| PM10,C06 | pH 4.5 + L-Tryptophan | 0 | 0 | 0 |
| PM10,C07 | pH 4.5 + L-Tyrosine | 5 | 4 | 5 |
| PM10,C08 | pH 4.5 + L-Valine | 0 | 0 | 0 |
| PM10,C09 | pH 4.5 + Hydroxy-L-Proline | 0 | 0 | 0 |
| PM10,C10 | pH 4.5 + L-Ornithine | 5 | 4 | 4 |
| PM10,C11 | pH 4.5 + L-Homoarginine | 0 | 0 | 3 |
| PM10,C12 | pH 4.5 + L-Homoserine | 0 | 0 | 0 |
| PM10,D01 | pH 4.5 + Anthranilic Acid | 0 | 0 | 0 |
| PM10,D02 | pH 4.5 + L-Norleucine | 0 | 0 | 0 |
| PM10,D03 | pH 4.5 + L-Norvaline | 0 | 0 | 0 |
| PM10,D04 | pH 4.5 + a- Amino-N-Butyric Acid | 0 | 0 | 0 |
| PM10,D05 | pH 4.5 + a-Amino Malonate | 0 | 0 | 0 |
| PM10,D06 | pH 4.5 + b-Hydroxy Glutamate | 5 | 5 | 4 |
| PM10,D07 | pH 4.5 + g-Hydroxy Glutamic Acid | 5 | 4 | 4 |
| PM10,D08 | pH 4.5 + 5-Hydroxy-L-Lysine | 5 | 5 | 4 |
| PM10,D09 | pH 4.5 + 5-Hydroxy-L-Tryptophan | 2 | 3 | 1 |
| PM10,D10 | pH 4.5 + D,L Diamino-Pimelic Acid | 0 | 0 | 0 |
| PM10,D11 | pH 4.5 + Trimethylamine-N-Oxide | 0 | 0 | 0 |
| PM10,D12 | pH 4.5 + Urea | 0 | 0 | 0 |
| PM10,E01 | pH 9.5 | 4 | 0 | 3 |
| PM10,E02 | pH 9.5 + L-Alanine | 4 | 0 | 3 |
| PM10,E03 | pH 9.5 + L-Arginine | 4 | 0 | 3 |
| PM10,E04 | pH 9.5 + L-Asparagine | 4 | 0 | 3 |
| PM10,E05 | pH 9.5 + L-Aspartic Acid | 5 | 0 | 4 |
| PM10,E06 | pH 9.5 + L-Glutamic Acid | 5 | 0 | 4 |
| PM10,E07 | pH 9.5 + L-Glutamine | 5 | 4 | 5 |
| PM10,E08 | pH 9.5 + Glycine | 4 | 0 | 4 |
| PM10,E09 | pH 9.5 + L-Histidine | 3 | 0 | 1 |
| PM10,E10 | pH 9.5 + L-Isoleucine | 0 | 0 | 0 |
| PM10,E11 | pH 9.5 + L-Leucine | 0 | 0 | 0 |
| PM10,E12 | pH 9.5 + L-Lysine | 1 | 0 | 0 |
| PM10,F01 | pH 9.5 + L-Methionine | 4 | 0 | 3 |
| PM10,F02 | pH 9.5 + L-Phenylalanine | 0 | 0 | 0 |
| PM10,F03 | pH 9.5 + L-Proline | 2 | 0 | 2 |
| PM10,F04 | pH 9.5 + L-Serine | 1 | 0 | 2 |
| PM10,F05 | pH 9.5 + L-Threonine | 4 | 0 | 3 |
| PM10,F06 | pH 9.5 + L-Tryptophan | 0 | 0 | 0 |
| PM10,F07 | pH 9.5 + L-Tyrosine | 3 | 3 | 2 |
| PM10,F08 | pH 9.5 + L-Valine | 3 | 0 | 3 |
| PM10,F09 | pH 9.5 + Hydroxy-L-Proline | 5 | 0 | 3 |
| PM10,F10 | pH 9.5 + L-Ornithine | 0 | 0 | 0 |
| PM10,F11 | pH 9.5 + L-Homoarginine | 5 | 2 | 4 |
| PM10,F12 | pH 9.5 + L-Homoserine | 3 | 0 | 2 |
| PM10,G01 | pH 9.5 + Anthranilic Acid | 3 | 0 | 3 |
| PM10,G02 | pH 9.5 + L-Norleucine | 0 | 0 | 0 |
| PM10,G03 | pH 9.5 + L-Norvaline | 5 | 0 | 4 |
| PM10,G04 | pH 9.5 + Agmatine | 5 | 0 | 4 |
| PM10,G05 | pH 9.5 + Cadaverine | 3 | 0 | 3 |
| PM10,G06 | pH 9.5 + Putrescine | 4 | 0 | 2 |
| PM10,G07 | pH 9.5 + Histamine | 0 | 0 | 3 |
| PM10,G08 | pH 9.5 + Phenylethylamine | 0 | 0 | 0 |
| PM10,G09 | pH 9.5 + Tyramine | 0 | 0 | 0 |
| PM10,G10 | pH 9.5 + Tryptamine | 0 | 0 | 0 |
| PM10,G11 | pH 9.5 + Trimethylamine-N-Oxide | 0 | 0 | 0 |
| PM10,G12 | pH 9.5 + Urea | 0 | 0 | 0 |
| PM10,H01 | X-Caprylate | 5 | 5 | 5 |
| PM10,H02 | X-α-D-Glucoside | 5 | 5 | 5 |
| PM10,H03 | X-β-D-Glucoside | 5 | 5 | 5 |
| PM10,H04 | X-α-D-Galactoside | 5 | 5 | 5 |
| PM10,H05 | X-β-D-Galactoside | 5 | 5 | 5 |
| PM10,H06 | X-α-D-Glucuronide | 5 | 4 | 5 |
| PM10,H07 | X-β-D-Glucuronide | 5 | 4 | 5 |
| PM10,H08 | X-β-D-Glucosaminide | 5 | 5 | 5 |
| PM10,H09 | X-β-D-Galactosaminide | 5 | 5 | 5 |
| PM10,H10 | X-α-D-Mannoside | 5 | 2 | 5 |
| PM10,H11 | X-PO4 | 5 | 4 | 5 |
| PM10,H12 | X-SO4 | 5 | 5 | 5 |
