# Supplementary material for: From Insect to Man: Photorhabdus Sheds Light on the Emergence of Human Pathogenicity
Source: PLoS One. 2015 Dec 17;10(12):e0144937. doi: 10.1371/journal.pone.0144937 (PMC4683029; doi:10.1371/journal.pone.0144937)
Supplement: S12 Table — (DOCX) [file pone.0144937.s027.docx]

**Table S9**: **Changes in the production of known secondary metabolites found in *P. asymbiotica* at 28°C and 37°C.** This analysis is based on LC-MS data derived from crude extracts of *P. asymbiotica* grown at different temperatures in the presence of Amberlite ^®^ XAD-16 adsorber resin. For the calculation of the relative changes in the production of the NPs the total ion counts were used (correlating with an increased production (green arrow) or a decreased production (red) at 37°C).

| **Rt [min]** | **[M+H]^+^** | **Factor (37°C/28°C)** |  | **Substance** | **Reference** |
| --- | --- | --- | --- | --- | --- |
| 9.2 | 255.11 | 1.4634 | 🡹 | isopropyl stilbene | {Fuchs, 2013 #6251} |
| 9.2 | 259.12 | 0.8088 | 🡻 | isopropyl stilbene derivative | {Fuchs, 2013 #6251} |
| 10.4 | 239.17 | 0.8474 | 🡻 | 1,3-cyclohexanedione-239 | {Fuchs, 2013 #6251} |
| 10.4 | 586.44 | 2.6033 | 🡹 | GameXPeptide A | {Bode, 2012 #6252} |
| 11.6 | 267.23 | 1.1545 | 🡹 | 1,3-cyclohexanedione-267 | {Fuchs, 2013 #6251} |
