# Supplementary material for: From Insect to Man: Photorhabdus Sheds Light on the Emergence of Human Pathogenicity
Source: PLoS One. 2015 Dec 17;10(12):e0144937. doi: 10.1371/journal.pone.0144937 (PMC4683029; doi:10.1371/journal.pone.0144937)
Supplement: S13 Table — (DOCX) [file pone.0144937.s028.docx]

**Table S10.** qRT-PCR primers used for data presented in Figure S5.

| **Gene** | **Sequence (5' - 3')** | **Tm** | **Product size** |
| --- | --- | --- | --- |
| rpoA for | AGAAGATGAGCGCCCTATTG | 59.43 | 114 |
| rpoA rev | TTGTCCAAATCGGTACGTTG | 59.44 | 114 |
| SRP for | TTCTCCCGCAACGCTAACT | 60.92 | 60 |
| SRP rev | CTTGCTATGGCTGCTTCCTT | 59.61 | 60 |
| prtA for | TACAACCCGTACCGATGACA | 59.84 | 105 |
| prtA rev | TCCCAAGCAGTGAACAACAG | 59.87 | 105 |
| purH for | ATGGATGGTCGCGTTAAGAC | 59.96 | 147 |
| purH rev | AACGGTCTGAGCAAATGGAT | 59.56 | 147 |
| malE for | AAGGTGAAACAGCGATGACC | 60.12 | 134 |
| malE rev | CGTCAATACACCGACAAACG | 60.03 | 134 |
| asnA for | CGCGCTATTGCTAAAGAGTTG | 60.18 | 114 |
| asnA rev | TGGTGTTGTCCAATCATCGT | 59.81 | 114 |
| mcf for | TGTTCGGTGGAGTTGTGGTA | 60.00 | 106 |
| mcf rev | ATCGGCTGCGAGTTAGAGAA | 60.12 | 106 |
| katE for | TGCTGACAACTGGAACCATC | 59.68 | 115 |
| katE rev | CTCACTGGCGATACGATCAA | 59.82 | 115 |
| PAU_02188 for | CCGTTAAATCCAAGGCTCAA | 60.07 | 115 |
| PAU_02188 rev | CAGCCAAGACCTTCTCTTGC | 60.13 | 115 |
| cipA for | CGATATGCACCCTTCTTTGG | 60.46 | 140 |
| cipA rev | AGCCGCCATCAAGGAGTAAT | 60.98 | 140 |
| cipB for | TGTCACCCAGTTCAGTCATTG | 59.58 | 120 |
| cipB rev | AAAGAGAAAGATAGAGCAGCACA | 57.61 | 120 |
| cipC for | ACATCCTTCTTCAAACGGTGA | 59.58 | 106 |
| cipC rev | TGCAGCTTGGACTGAACTTACT | 59.19 | 106 |
| csrB for | GACACAATGCATAACTGGATCG | 60.39 | 100 |
| csrB rev | GTTTGCTCCCTGCTCACTCC | 62.39 | 100 |
